# 20S proteasome-regulated proteostasis in ELVAs is critical for oocyte-to-embryo transition and female fertility

**DOI:** 10.1038/s44318-026-00813-0

**Published:** 2026-05-21

**Authors:** Yan Rong, Yingyan Chen, Huiwen Cao, Chengkan Liu, Haomang Xu, Xiaomei Tong, Anxuan Fang, Leilei Ai, Yucong Zhu, Yingyi Zhang, Peipei Ren, Xiaoxuan Li, Yufan Gao, Xiaolin Tian, Lugeng He, Songying Zhang, Chao Yu

**Affiliations:** 1 Assisted Reproduction Unit, Department of Obstetrics and Gynecology, Sir Run Run Shaw Hospital, Zhejiang University, School of Medicine; Zhejiang Key Laboratory of Precise Protection and Promotion of Fertility; Zhejiang Provincial Clinical Research Center for Reproductive Health and Disease, Hangzhou, China; 2 MOE Key Laboratory of Biosystems Homeostasis and Protection, College of Life Sciences, Zhejiang University, Hangzhou, China; 3 Technology Center for Protein Sciences, Tsinghua University, Beijing, China; 4 Department of Urology, the First Affiliated Hospital, Zhejiang University School of Medicine, Zhejiang University, Hangzhou, China

**Keywords:** Cell Cycle, Development, Post-translational Modifications & Proteolysis

## Abstract

Programmed degradation of maternal proteins is essential for the oocyte-to-embryo transition (OET). While pharmacological inhibition studies have established the importance of proteasomes in ovarian reserve maintenance, oocyte maturation and fertilization, the physiological impact of intrinsic proteasome insufficiency and underlying molecular mechanisms remain poorly understood. In mice, endolysosomal vesicular assemblies (ELVAs), specialized membraneless compartments composed of proteasomes, endolysosomes and autophagosomes, facilitate protein degradation during oocyte maturation and early embryogenesis. In this study, we generated mice with oocyte-specific deletion of the proteasomal core subunit *Psma7*, to investigate the physiological function of the 20S proteasome and its roles in ELVAs-mediated protein degradation. PSMA7-deficiency destabilized 20S proteasomes and disrupted translocation of ELVAs, leading to pronounced accumulation of ubiquitinated proteins in oocytes and zygotes. Consequently, maternal *Psma7* deletion resulted in female infertility, manifested by impaired oocyte maturation and developmental arrest at one- to two-cell stage. Furthermore, we observed reduced proteasome abundance and dysfunction of ELVAs in aged oocytes, providing a mechanistic explanation for the decline in developmental competence associated with oocyte aging. Taken together, our findings elucidate the critical function of proteasome-regulated proteostasis within ELVAs in maintaining oocyte quality during OET and reproductive aging.

## Synopsis

Proteasome activity has been implicated in the mammalian oocyte-to-embryo transition (OET). This study adopts oocyte-specific knockout of PSMA7, the α4 subunit of 20S proteasome, to investigate the role of proteasome in ELVAs formation, OET and reproductive aging, with implications for clinical diagnosis and treatment of poor oocyte quality.

- PSMA7-deletion destabilizes 20S proteasome components in oocytes and eggs.
- PSMA7-deletion disrupts the assembly and oocyte-cortex translocation of endolysosomal vesicular assemblies (ELVAs).
- PSMA7-deficiency leads to pronounced accumulation of ubiquitinated proteins in oocytes and zygotes.
- Maternal Psma7 deletion results in female infertility, manifested by impaired oocyte maturation and developmental arrest at the 1- to 2-cell stage.
- Reduced proteasome abundance and ELVAs dysfunction exist in aged mouse oocytes.

## Abstract (web summary)

Oocyte-specific knockout of proteasomal PSMA7 subunit causes accumulation of ubiquitinated proteins and impairs cortical translocations of endolysosomal vesicular assemblies (ELVAs).

## Graphical abstract

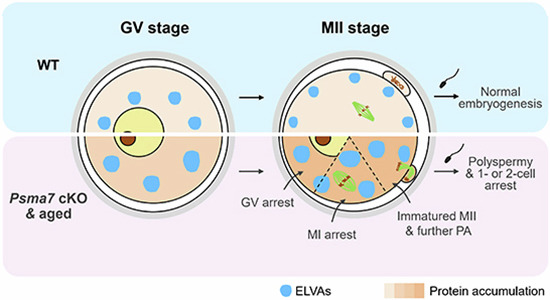

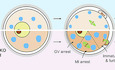

## Introduction

During the oocyte-to-embryo transition (OET), oocytes contribute to approximately half of the nuclear DNA and nearly all cytoplasmic components through a tightly orchestrated program encompassing oogenesis, meiotic maturation, fertilization and zygotic genome activation (ZGA) (Stitzel and Seydoux, 2007; Svoboda, 2018). As a hallmark of the OET, the regulated degradation of maternal factors (e.g., mRNAs and proteins), coupled with the de novo synthesis of zygotic factors, is essential for generating developmentally competent embryos (Bebbere et al, 2021; Li et al, 2010; Mitchell, 2022; Zhang and Smith, 2015), and its failure often leads to female infertility, miscarriage and birth defects (Sha et al, 2019; Toralova et al, 2020; Yu et al, 2016). Recent advances in single-cell transcriptome, translatome and proteome profiling have substantially elucidated gene expression regulation during OET, with extensive focus on mRNA decay (Hu et al, 2022; Jiang et al, 2023b, Sha et al, 2019; Sha et al, 2020; Svoboda et al, 2017; Zou et al, 2022). The abnormalities in proteostasis have also been discovered in mammalian aged oocytes including human samples (Galatidou et al, 2024; Harasimov et al, 2024). However, the molecular mechanisms involved in maternal protein clearance which requires more intricate spatiotemporal regulation, particularly their physiological significance in OET and oocyte aging remain poorly understood.

The ubiquitin-proteasome system (UPS) represents the major pathway for regulated protein degradation in eukaryotes, comprising E1, E2, E3 enzymes and the proteasome (Pohl and Dikic, 2019). Previous studies have demonstrated the oocyte-specific functions of multiple E3 ligases and their substrates in oocyte maturation of mammals. For instance, the SCF^β-TrCP^ complex degrades CPEB1 to mediate maternal mRNA translation (Sha et al, 2017); CRL4^DCAF1^-dependent degradation of PP2A is essential for meiotic progression (Yu et al, 2015); CRL4^DCAF13^ targets PTEN degradation to support meiotic resumption (Zhang et al, 2020). Moreover, declined proteasomal activity has been demonstrated to promote the accumulation of oxidative damage and leads to impaired oocyte quality (Galatidou et al, 2024; Mihalas et al, 2018; Sasaki et al, 2019). Pharmacological inhibition of proteasomal activity with ALLN or MG132 also disrupts the key events in oocyte maturation, such as the meiosis I-to-II transition in mouse oocytes depending on the degradation of securin and Cyclin B1 regulated by APC/C and the 26S proteasome (Huo et al, 2004; Shin et al, 2010; Terret et al, 2003). Therefore, investigating the dynamics and roles of proteasomes in oocyte maturation and aging is crucial for elucidating the mechanisms underlying maternal protein degradation, especially under physiological conditions.

Recent studies have shown that proteasomes are incorporated into a degradative super-organelle known as endolysosomal vesicular assemblies (ELVAs) in oocytes and early embryos (Zaffagnini et al, 2024). ELVAs also consist of endolysosomes and autophagosomes and are held together by RUFY1 to sequester detrimental protein aggregates (Satouh et al, 2025; Zaffagnini et al, 2024). During oocyte maturation, ELVAs translocate to the oocyte cortex and exhibit increased degradative activity (Zaffagnini et al, 2024). Both proteasomal and lysosomal activities are critical for protein degradation within ELVAs, as demonstrated by pharmacological inhibition using MG132 or BAFA1, which disrupt ELVAs assembly and distribution (Zaffagnini et al, 2024). These disruptions lead to abnormal spindle assembly during oocyte maturation and cause early embryonic arrest (Bowman et al, 1988; Klionsky et al, 2021; Zaffagnini et al, 2024; Zaffagnini et al, 2025). However, the specific contributions of proteasomes within ELVAs have not been investigated in vivo, largely due to the essential role of proteasomes in cell viability (Park et al, 2024).

The canonical 26S proteasome comprises a 20S core particle capped at one or both ends by 19S regulatory particles (Huang et al, 2016). The 20S core is composed of four stacked heteroheptameric rings (α–β–β–α), with the inner β-rings mediating proteolytic activity and the outer α-rings facilitating recognition and delivery of polyubiquitinated substrates (Zhang et al, 2019a). Deletion of the *Psma7* subunit in male germ cells leads to defects in proliferation and differentiation of spermatogonia (Fang et al, 2025). In this study, we identified that PSMA7 is notably abundant among 26S proteasome subunits in both human and mouse oocytes. To investigate the physiological function of proteasomes in ELVAs-mediated protein degradation during OET and reproductive aging, we generated oocyte-specific *Psma7* knockout mice (*Psma7*^*fl/fl*^*;Zp3-Cre*). PSMA7-deficiency destabilized 20S proteasome in oocytes, resulting in the accumulation of ubiquitinated proteins. Furthermore, ELVAs in PSMA7-deficient oocytes and zygotes exhibited abnormal enlargement and insufficient cortical relocation during oocyte maturation. Consequently, female mice lacking oocytic PSMA7 were infertile, displaying defects in meiotic maturation and embryonic arrest at the 1- or 2-cell stage. Notably, similar abnormalities in ELVAs assembly and translocation, along with decreased proteasome levels, were observed in aged oocytes, underscoring the critical role of proteasomes in maintaining oocyte quality during OET and reproductive aging.

## Results

### Decreased 20S proteasome abundance underlies age-related proteasomal dysfunction in oocytes

**Figure 1:**
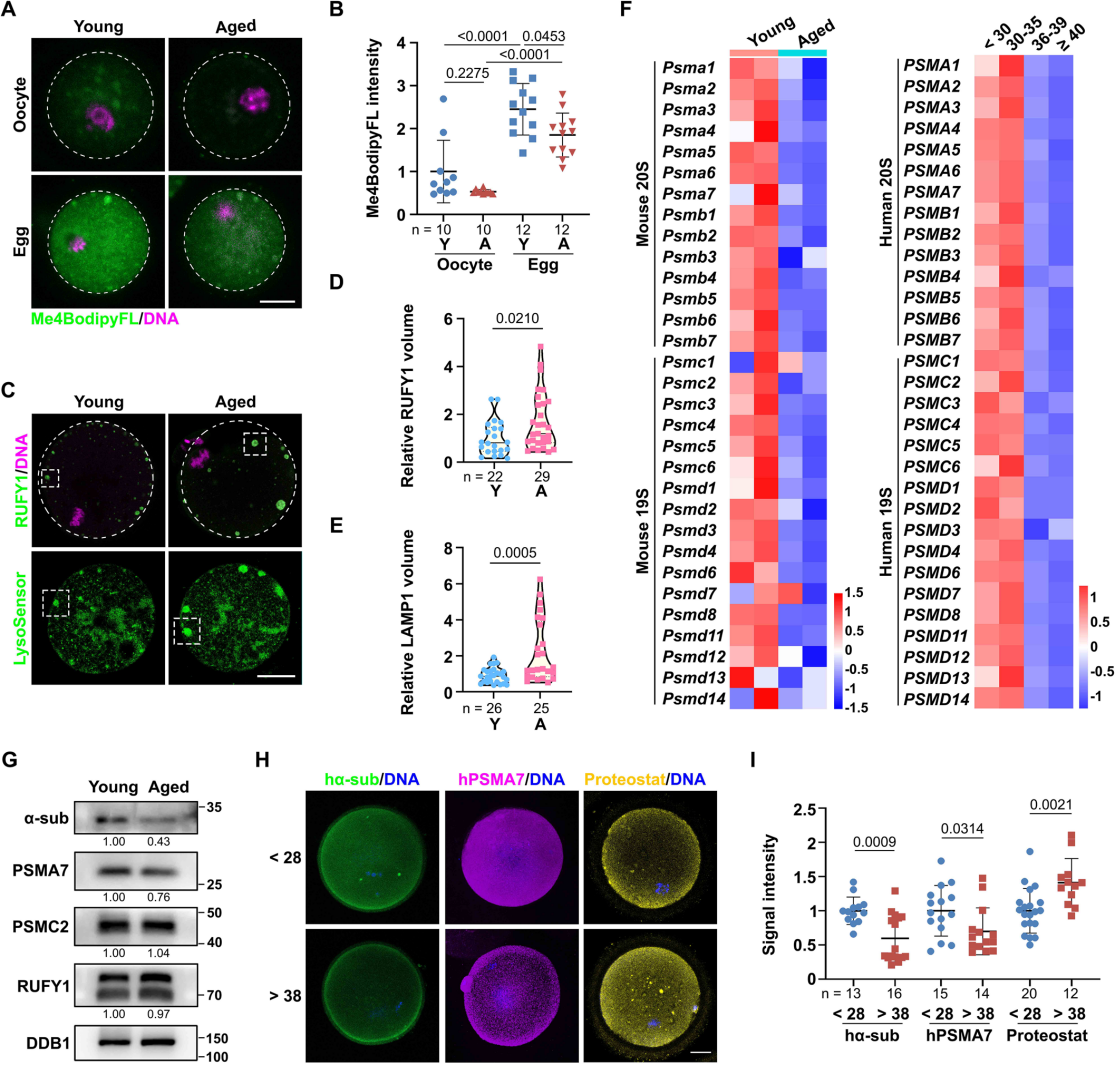
20S proteasome decline is associated with oocyte aging. (**A**, **B**) Confocal analysis with Me4BodipyFL probe (**A**) and quantification of Mean Fluorescence Intensity (MFI) (**B**) of GV oocytes and eggs derived from young and aged mice. Scale bar, 20 μm. Y young, A aged. Mean and SD are shown. *P* values: one-way ANOVA. (**C**) Confocal analysis of RUFY1 and LysoSensor signals in young and aged mouse eggs. Scale bar, 20 μm. (**D**, **E**) Quantification of the relative average volume of RUFY1 (**D**) and LAMP1 (**E**) puncta in young and aged mouse eggs by 3D models. *P* values: unpaired *t* test. (**F**) Heat map illustrating the differential expression profiles of proteasome subunits in mouse (left) and human (right) GV oocytes across distinct age groups. Data were reanalyzed from Wu et al, 2022. (**G**) Western blot analysis of indicated proteins in GV oocytes derived from young and aged female mice. DDB1 serves as the loading control. Quantification of the band intensities is shown below the blot. (**H**) Confocal analysis of human α-subunits (hα-sub), hPSMA7 and Proteostat signals in human eggs of indicated age groups. Scale bar, 20 μm. (**I**) Quantification of MFI in (**H**). Mean and SD are shown. *P* values: unpaired *t* test. For all statistical analysis, *n* indicates the number of oocytes analyzed. Source data are available online for this figure.

The proteasome is a primary machinery for targeted protein degradation, and its functional integrity is essential for maintaining proteostasis. Given that the loss of protein homeostasis is the common feature of cellular aging (Labbadia and Morimoto, 2015), we examined whether proteasomal activity of oocytes is altered during maternal aging using the cell-permeable fluorescent probe Me4BodipyFL. The initial assessment revealed that proteasomal activity in aged mouse GV oocytes was comparable to that in young GV oocytes (Fig. 1A,B), consistent with previous reports (Harasimov et al, 2024). However, while proteasomal activity increased during the transition to the MII phase, it remained relatively low in aged eggs (here referring to MII-stage oocytes) (Fig. 1A,B), suggesting an aging-related impairment in proteasome function. Given the role of proteasomes in protein-degrading ELVAs where they collaborate with lysosomes and other organelles to clear protein aggregates in oocytes (Zaffagnini et al, 2024), we investigated whether ELVAs formation is disrupted with aging. RUFY1 has been identified as a structural organizer of ELVAs, and we observed that RUFY1 and LysoSensor positive compartments tended to be larger in aged oocytes (Fig. 1C; Appendix Fig. S1A). To further confirm this enlargement, we applied the macro-based quantification of their volume on 3D models. Enlarged RUFY1-positive compartments were validated in aged oocytes (Fig. 1D), despite its unchanged protein levels (Fig. 1G) and relative distance from cortex (Appendix Fig. S1B). Similarly, the size of LAMP1-positive compartments was also increased in aged oocytes (Fig. 1E; Appendix Fig. S1C,D), consistent with aberrant ELVAs assembly during oocyte aging.

To assess whether proteasome abundance changes during oocyte aging, we re-analyzed transcriptome datasets derived from both young and aged oocytes of mouse and human (Wu et al, 2022; Zhang et al, 2023b). This revealed aging-related downregulation of transcripts encoding proteasomal subunits, especially those comprising the 20S core particle (Fig. 1F; Appendix Fig. S1E). Western blot analysis confirmed reduced protein levels of 20S α-subunits using the specific antibody (α-sub) recognizing α1, 2, 3, 5, 6 and 7, as well as the antibody against PSMA7 (α4) in aged mouse oocytes (Fig. 1G). Although transcription levels of 19S subunits also declined with maternal aging (Fig. 1F; Appendix Fig. S1E), the protein level of PSMC2 remained stable (Fig. 1G), suggesting that the loss of proteasomal activity in aged oocyte is likely attributed to reduced 20S proteasome abundance rather than 19S.

Notably, while human oocytes exhibit higher proteasomal activity at the GV stage than at MII, a pattern opposite to that in mice, proteasome function remains essential for maturation (Zaffagnini et al, 2025). Although human oocytes lack canonical ELVAs (Appendix Fig. S1F,G) and instead accumulate protein aggregates within enlarged lysosomes (Zaffagnini et al, 2025), comparative analysis revealed that oocytes from women over 38 years old contain more protein aggregates and lower levels of α-subunits and PSMA7 than those from women under 28 (Fig. 1H,I), mirroring observations in aged mouse oocytes (Fig. 1G). These findings support the conclusion that a decline in 20S proteasome abundance and consequent impairment of protein degradation represent potential mechanisms underlying oocyte aging.

### Oocyte-specific PSMA7 depletion impairs proteasome function

**Figure EV1:**
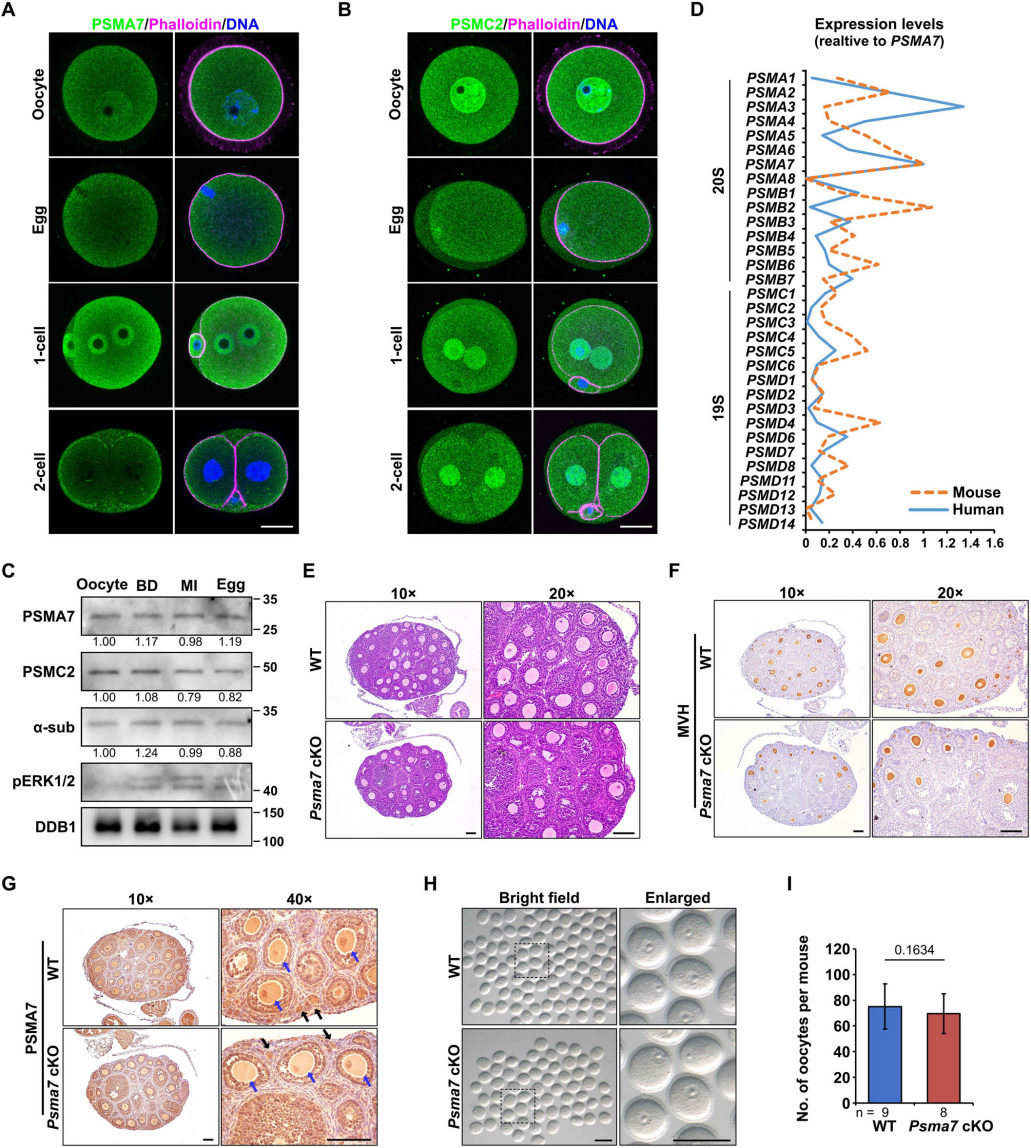
Histological analyses of ovaries and oocytes derived from WT and *Psma7* cKO females. (**A**, **B**) Immunofluorescent staining of PSMA7 (**A**) and PSMC2 (**B**) in mouse oocytes and early embryos. Scale bar, 20 μm. (**C**) Western blot analysis of PSMA7, PSMC2 and α-subunits levels during mouse oocyte maturation. DDB1 serves as the loading control. Total protein lysates from 50 oocytes were loaded in each lane. Quantification of the band intensities is shown below the blot. (**D**) The line chart illustrating the expression levels of proteasome subunits in mouse and human oocytes. Data were reanalyzed from Wu et al, 2022. (**E**) H&E staining of WT and *Psma7* cKO ovaries at PD21 (Postnatal Day 21). Scale bar, 100 μm. (**F**, **G**) IHC analysis of MVH (**F**) and PSMA7 (**G**) levels in WT and *Psma7* cKO ovaries at PD21. The black and blue arrows denote primordial and primary follicles, respectively. Scale bar, 100 μm. (**H**, **I**) Representative images of GV oocytes (**H**) and quantification of their number (**I**) collected from WT and *Psma7* cKO ovaries. BF brightfield. Scale bar, 100 μm. *n* indicates the number of female mice analyzed. Mean and SD are shown. *P* values: unpaired *t* test.

**Figure 2:**
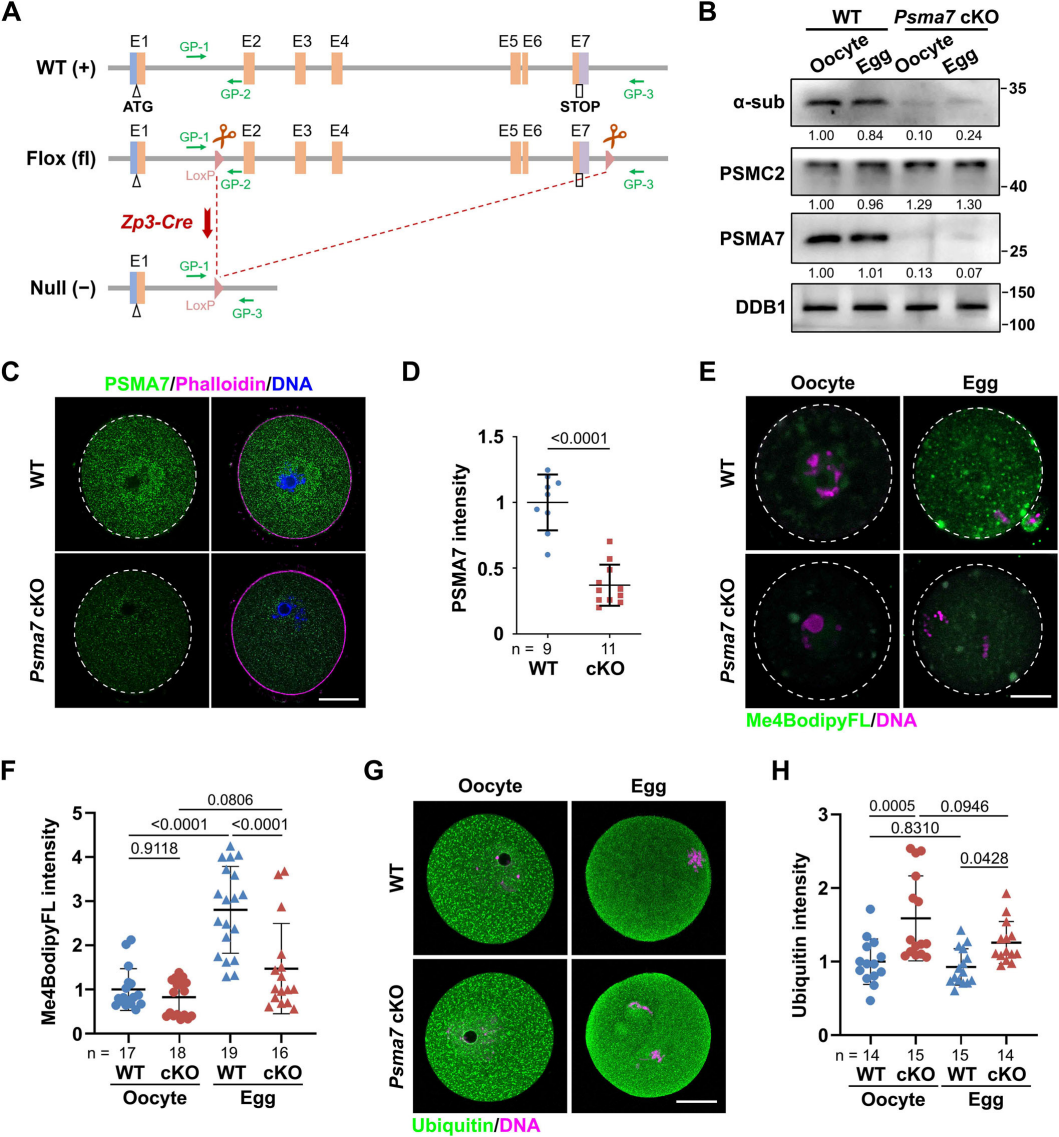
Proteasomal dysfunction and ubiquitinated protein accumulation in PSMA7-deficient oocytes. (**A**) Schematic diagram of the strategy for generating oocyte-specific *Psma7*-null allele. The genomic structure of mouse *Psma7* gene locus is shown, with exons (E1-E7), start codon, stop codon and LoxP sites indicated. Green arrows mark the positions of PCR primers used for genotyping. (**B**) Western blot analysis of indicated proteasome subunits in WT and *Psma7* cKO oocytes. (**C**, **D**) Immunofluorescent staining of PSMA7 (**C**) and quantification of MFI (**D**) in WT and *Psma7* cKO GV oocytes. Scale bar, 20 μm. Mean and SD are shown. *P* values: unpaired *t* test. (**E**–**H**) Confocal analysis of Me4BodipyFL (**E**) or ubiquitin signal (**G**) and quantification of the relevant MFI (**F**, **H**) in GV oocytes and eggs from WT and *Psma7* cKO females. Scale bars, 20 μm. Mean and SD are shown. *P* values: one-way ANOVA. For all statistical analysis, *n* indicates the number of oocytes analyzed. Source data are available online for this figure.

PSMA7, α-subunits and PSMC2 remain robustly expressed in oocytes and eggs, as well as in 1- and 2-cell embryos (Fig. EV1A–C), suggesting their functional importance during OET. Among the 26S proteasome subunits, PSMA7 (α4) is notably highly expressed in both human and mouse oocytes (Fig. EV1D). Given that the assembly of the α-ring precedes that of the β-ring during proteasome maturation (Rousseau and Bertolotti, 2018), we generated an oocyte specific *Psma7* conditional knockout mouse model *Psma7*^*fl/fl*^*;Zp3-Cre* (*Psma7* cKO), in which CRE recombinase is selectively expressed in oocytes from the primary follicle stage onward (Fig. 2A), to investigate the physiological role of 20S proteasomes in ELVAs dynamics and the maintenance of female fertility.

Ovarian morphology and follicular development in *Psma7* cKO females at postnatal day 21 (PD21) were comparable to those of WT mice (Fig. EV1E–G). Immunohistochemistry revealed robust PSMA7 expression in oocytes from the primordial follicle stage in WT females, whose level was significantly reduced in *Psma7* cKO oocytes beginning from the primary follicle stage (Fig. EV1G, blue arrows). Comparable numbers of morphologically normal oocytes were retrieved from both genotypes (Fig. EV1H,I). Western blot and immunofluorescent staining confirmed efficient depletion of PSMA7 and a concomitant decrease in α-subunit levels, whereas PSMC2 remained unchanged in *Psma7* cKO oocytes (Fig. 2B–D), indicating that PSMA7 is essential for the assembly of 20S, but not 19S, proteasomes. Proteasomal activity in *Psma7* cKO GV oocytes was reduced by roughly 20%, though not statistically significantly, but markedly impaired in eggs (Fig. 2E,F). Consistent with this, ubiquitinated proteins accumulated in both oocytes and eggs upon PSMA7 deletion (Fig. 2G,H), further confirming proteasomal dysfunction.

### PSMA7 deletion disrupts ELVAs translocation and protein aggregate clearance

**Figure 3:**
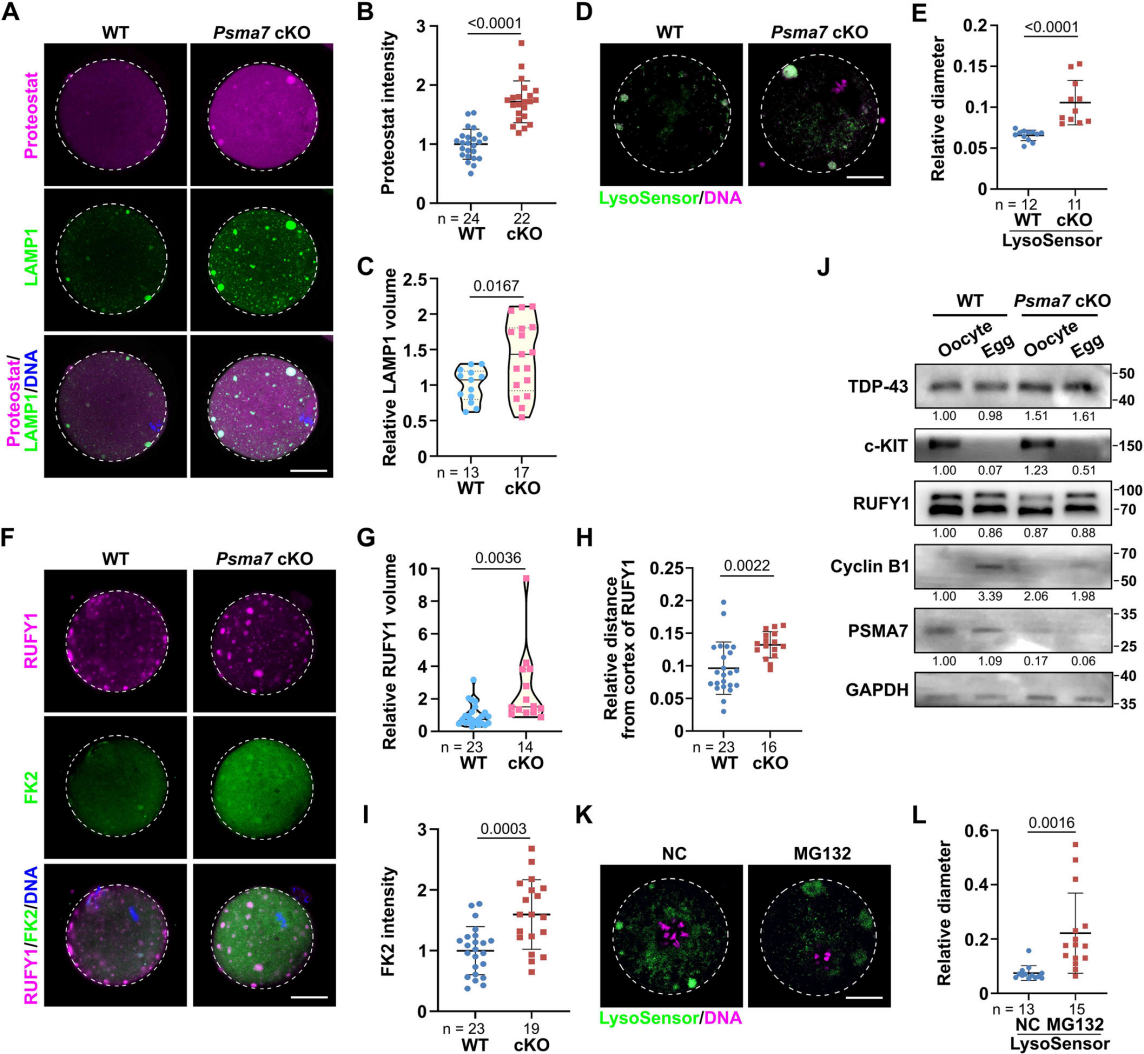
PSMA7 depletion disrupts the formation and translocation of ELVAs. (**A**) Confocal analysis of Proteostat and LAMP1 co-localization. Scale bar, 20 μm. (**B**) Quantification of the Proteostat signal MFI in WT and *Psma7* cKO eggs. Mean and SD are shown. *P* values: unpaired *t* test. (**C**) Quantification of the relative average volume of LAMP1 puncta in WT and *Psma7* eggs by 3D models. *P* values: unpaired *t* test. (**D**, **E**) Confocal analysis of LysoSensor (**D**) and quantification of the relative diameter of the compartments (**E**) in WT and *Psma7* cKO eggs. Scale bar, 20 μm. Mean and SD are shown. *P* values: unpaired *t* test. (**F**) Confocal analysis of RUFY1 and FK2. Scale bar, 20 μm. (**G**) Quantification of the relative average volume of RUFY1 puncta in WT and *Psma7* eggs by 3D models. *P* values: unpaired *t* test. (**H**, **I**) Quantification of the relative distance from cortex of RUFY1-positive compartments (**H**) and the FK2 signal MFI (**I**) in WT and *Psma7* cKO eggs. Mean and SD are shown. *P* values: unpaired *t* test. (**J**) Western blot analysis of indicated proteins in WT and *Psma7* cKO oocytes. GAPDH serves as the loading control. (**K**, **L**) Confocal analysis of LysoSensor signal (**K**) and quantification of the diameter of the compartments (**L**) in mouse MI oocytes treated with or without MG132 (20 μM) during maturation. NC negative control. Scale bar, 20 μm. Mean and SD are shown. *P* values: unpaired *t* test. For all statistical analysis, *n* indicates the number of oocytes analyzed. Source data are available online for this figure.

We next examined the effect of PSMA7 depletion on protein aggregate clearance and ELVAs integrity. Analysis of compartments co-stained for Proteostat and LAMP1 revealed more distinct and prominently localized protein aggregates within ELVAs in *Psma7* cKO oocytes compared to WT controls (Fig. 3A,B). Fluorescent staining of LysoSensor and RUFY1 further indicated the defects in ELVAs function (Fig. 3D–F). During oocyte maturation, ELVAs compartments are normally enlarged and translocated to the cortex to facilitate protein degradation; however, the enlargement of LAMP1 or RUFY1-positive compartments was particularly evident in *Psma7* cKO oocytes (Fig. 3C,G; Appendix Fig. S2A,B), and they failed to migrate to the cortex (Fig. 3H). The defects were further validated in WT and *Psma7* cKO oocytes overexpressing GFP-tagged RUFY1 (Appendix Fig. S1C,D). Additionally, the accumulation of ubiquitinated proteins labelled with FK2 antibody was also evident upon PSMA7 deletion (Fig. 3F,I), resembling the defects observed in aged oocytes. Notably, among ELVAs substrates (Zaffagnini et al, 2024), TDP-43 was upregulated in *Psma7* cKO oocytes (Fig. 3J). While c-KIT did not show marked accumulation (Fig. 3J), likely due to residual proteasomal function, its degradation was mediated through both proteasomal and lysosomal pathways (Appendix Fig. S2E).

Residual PSMA7 protein in *Psma7* cKO oocytes might result in incomplete decrease of 20S and proteasomal inhibition, we employed MG132 treatment as a complementary approach to further investigate the consequences of total proteasome inhibition on ELVAs formation. The inhibitory effect was confirmed by declined Me4BodipyFL signal intensity (Appendix Fig. S2F,G), and increased Proteostat intensity (Appendix Fig. S2H). Although the size of LAMP1-positive puncta remained unchanged (Appendix Fig. S2H), compartments harboring active lysosomes were significantly enlarged in MG132-treated oocytes (Fig. 3K,L), with a more pronounced effect than that observed in *Psma7* cKO oocytes (Fig. 3D,E). This difference may be attributed to the residual PSMA7 protein in the genetic model due to its extended half-life (Figs. 2B–D and EV1G) (Harasimov et al, 2024). Taken together, these results demonstrate that PSMA7-dependent proteasome function is essential for ELVAs activation, translocation and protein degradation during oocyte maturation.

### Impaired protein homeostasis in PSMA7-deficient oocytes

**Figure 4:**
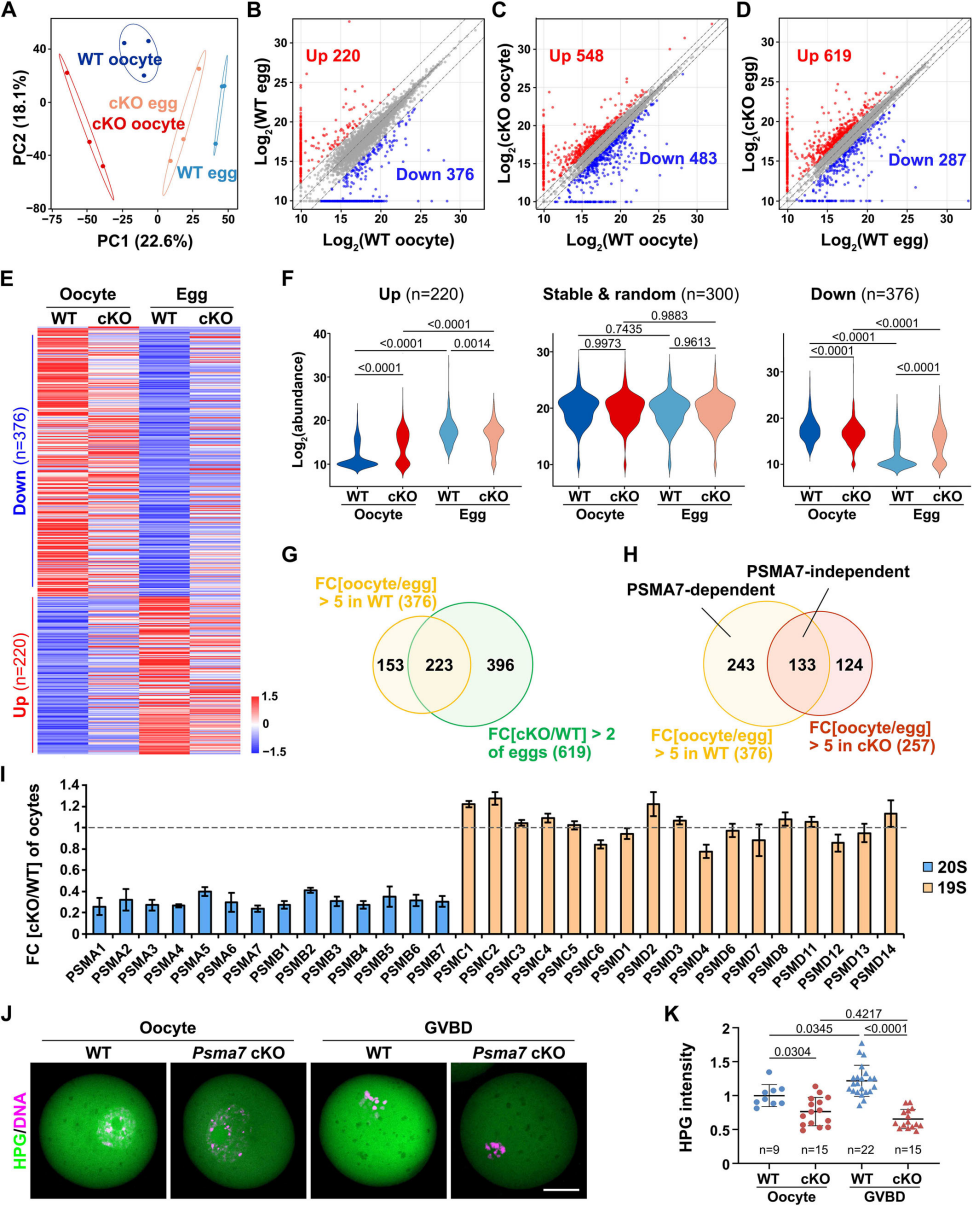
PSMA7-depletion disrupts protein degradation and translation in oocytes. (**A**) PCA analysis of proteomic data from GV oocytes and eggs obtained from WT and *Psma7* cKO females. (**B**–**D**) Scatter plots depicting fold changes in protein abundance. Comparisons are between: WT GV oocytes and eggs (**B**), WT and *Psma7* cKO GV oocytes (**C**), WT and *Psma7* cKO eggs (**D**). Proteins with > 5-fold (**B**) or > 2-fold (**C**, **D**) changes are highlighted in red (upregulated) or blue (downregulated). (**E**) Heat map of proteins up- and down-regulated as shown in (**B**). (**F**) Violin plots displaying fold changes of WT and *Psma7* cKO oocytes at GV or MII stage in the protein groups identified in panel B. The “Stable & random” group consists of 300 randomly selected proteins. *P* values: one-way ANOVA. (**G**) Venn diagram showing common proteins that are downregulated during the GV–MII transition in WT groups and are concurrently upregulated in *Psma7* cKO eggs. (**H**) Venn diagram showing common proteins that are downregulated during the GV–MII transition in both WT and *Psma7* cKO groups. (**I**) Fold changes of 20S and 19S subunits following PSMA7 depletion in GV oocytes. Analysis was conducted from three replicates of each genotype by proteomic data. SD is shown. (**J**, **K**) Confocal analysis of HPG signal (**J**) and quantification of MFI (**K**) in oocytes at indicated stages. Scale bar, 20 μm. *n* indicates the number of oocytes analyzed. Mean and SD are shown. *P* values: one-way ANOVA. Source data are available online for this figure.

Given that PSMA7 knockout disrupts both proteasome and ELVAs function, we sought to investigate how maternal PSMA7 deletion affects protein turnover during oocyte maturation. Fully-grown oocytes and ovulated eggs from WT and *Psma7* cKO mice were collected for micro-proteomic analysis, which is suitable for limited biological materials (Appendix Fig. S3A). The proteomic data from all groups exhibited high coverage and reproducibility across three biological replicates (Appendix Fig. S3B,C). Principal component analysis (PCA) clearly distinguished the control and experimental groups (Fig. 4A). In WT oocytes, 220 proteins were upregulated and 376 were downregulated by more than 5-fold during the GV to MII transition (Fig. 4B), reflecting extensive protein synthesis and degradation throughout maturation. Gene Ontology (GO) analysis indicated that upregulated proteins were enriched in pathways involved in meiosis progression, including cell cycle regulation, chromosome organization and spindle assembly, as well as histone modification (Appendix Fig. S3D). Downregulated proteins were associated with oocyte growth processes such as intermediate filament assembly, transcription, ribosome biogenesis, and phosphorylation (Appendix Fig. S3E). We validated several key regulators: ZAR1 and CPEB1 (translational control) (Rong et al, 2019; Sha et al, 2017), TPX2 (spindle assembly) (Chen et al, 2013; Helmke and Heald, 2014), BTG4 (maternal mRNA degradation) (Yu et al, 2016), and Cyclin B1 (cell cycle progression) (Yang et al, 2017). The observed protein dynamics during the GV to MII transition were consistent with their established roles (Appendix Fig. S1F), confirming the reliability of our dataset.

**Figure EV2:**
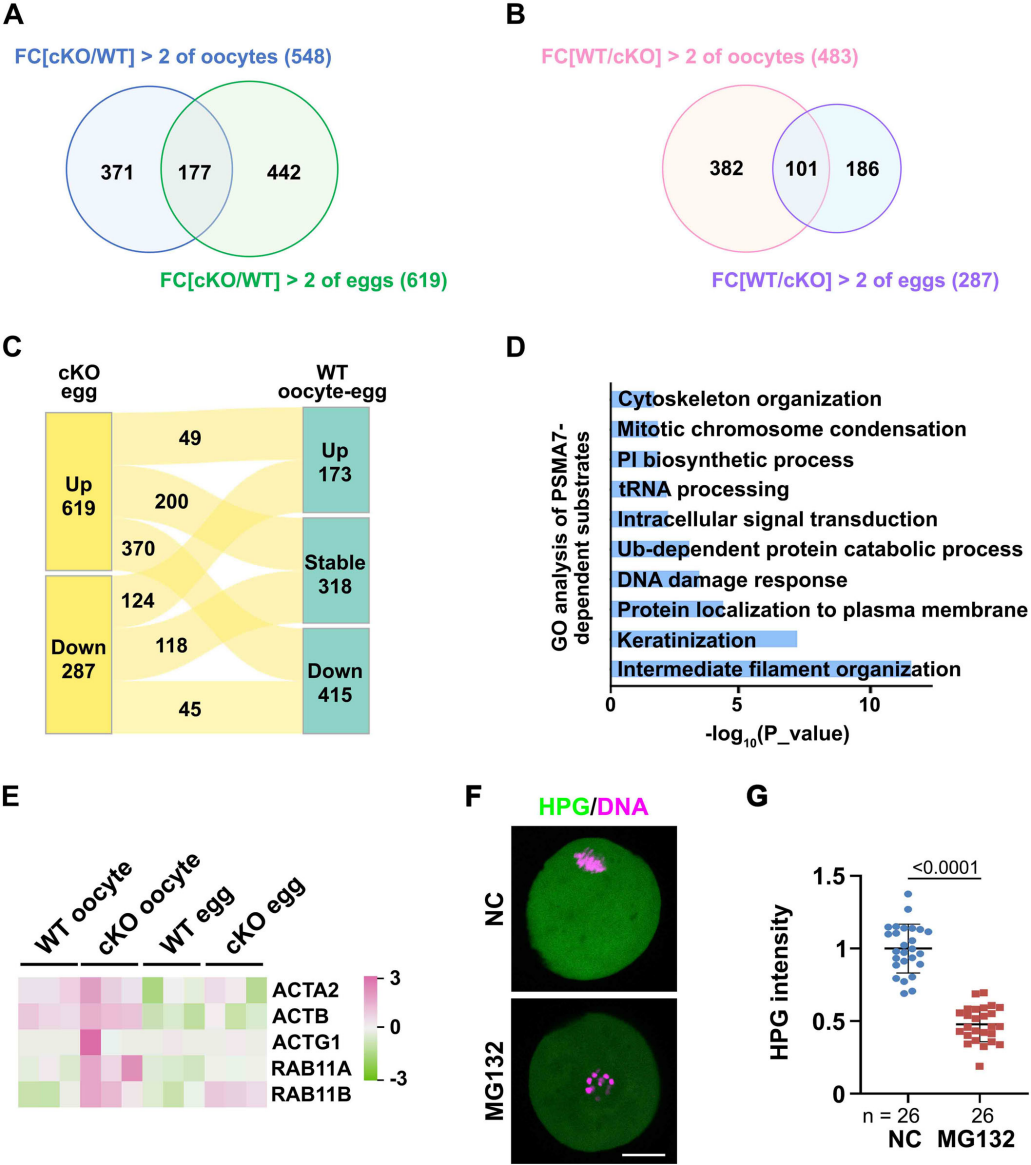
The dynamic proteomic landscape correlates with oocyte developmental competence and progression. (**A**, **B**) Venn diagram illustrating the overlap of significantly upregulated (**A**) or downregulated (**B**) proteins in both GV oocytes and eggs upon maternal PSMA7 depletion. (**C**) Sankey diagram showing how PSMA7 deficiency redirects the proteomic landscape, detailing shifts in up- and down-regulated proteins between WT and cKO eggs. (**D**) GO analysis of PSMA7-dependent substrates from Fig. 4H. Results are analyzed using the DAVID web server. (**E**) Heat map of actin-dependent relocation-related component levels in WT and *Psma7* cKO oocytes. (**F**) HPG fluorescent staining indicating protein synthesis activity in MI oocytes treated with or without MG132 treatment. Scale bar, 20 μm. (**G**) Quantification of HPG signal MFI in panel (**F**). *n* indicates number of oocytes. Mean and SD are shown. *P* values: unpaired *t* test.

After maternal *Psma7* deletion, 548 proteins were upregulated and 483 were downregulated (FC > 2) in GV oocytes (Fig. 4C), while 619 were upregulated and 287 were downregulated (FC > 2) in eggs (Fig. 4D). Notably, 32.3% (177/548) of proteins upregulated in oocytes remained elevated in eggs (Fig. EV2A), indicating persistent protein degradation defects throughout maturation. Furthermore, 35.2% (101/287) of downregulated proteins in eggs originated from reduced expression in oocytes (Fig. EV2B). Heat map and violin plot analyses revealed that PSMA7 deficiency not only impaired degradation of specific protein subsets, but also disrupted de novo synthesis of proteins required for oocyte maturation (Fig. 4E,F). Proteins that are typically stable from oocytes to eggs remained largely unaffected (Fig. 4F). Notably, accumulated proteins in *Psma7* cKO eggs primarily belonged to categories that are normally degraded or remain stable, whereas reduced proteins included those that are stable or upregulated during oocyte maturation (Fig. EV2C). Among the 376 proteins typically degraded during oocyte maturation, 223 (62.0%) accumulated in *Psma7* cKO eggs (Fig. 4G). The further overlap analysis with 257 proteins degraded in *Psma7* cKO oocytes during maturation identified 243 (64.6%) as PSMA7-dependent substrates (Fig. 4H). The remaining 133 (35.4%) were still efficiently degraded in the absence of PSMA7, indicating their turnover occurs through a PSMA7-independent pathway (Fig. 4H). The GO analysis revealed that PSMA7-dependent substrates were mainly associated with intermediate filament organization, keratinization, protein localization to plasma membrane, cytoskeleton organization and other processes (Fig. EV2D), suggesting that the structural support and stability of cells were severely affected. The cortical relocation of ELVAs depends on the actin/RAB11A axis (Zaffagnini et al, 2024). Notably, while the levels of multiple actin components remained unchanged, RAB11 components, which nucleate actin, were upregulated following PSMA7 deletion (Fig. EV2E), suggesting that elevated RAB11 may compensate for the impaired cortical translocation of overloaded ELVAs. Besides, consistent with western blot results (Fig. 2B), PSMA7-deletion led to pronounced downregulation of 20S proteasome subunits, with minimal effects on 19S components in oocytes (Fig. 4I).

Protein homeostasis is maintained through a balance of protein degradation and translation (Balch et al, 2008; Labbadia and Morimoto, 2015). Thus, we also examined translation activity to rule out the possibility that the accumulated proteins resulted from increased synthesis. We metabolically labeled newly synthesized proteins using a methionine analogue L-HPG. WT oocytes exhibited increased HPG incorporation after GVBD, indicating elevated translation upon meiotic resumption (Fig. 4J,K). In contrast, *Psma7* cKO oocytes showed significantly reduced HPG signals, particularly in GVBD groups (Fig. 4J,K). Impaired translation was also observed in MG132-treated oocytes (Fig. EV2F,G), further demonstrating that proteasome dysfunction attenuates global protein synthesis, rather than being an adaptive or indirect consequence. Taken together, PSMA7 deficiency disrupts protein homeostasis in oocytes by impairing both protein degradation and de novo protein synthesis.

### PSMA7-deletion has limited effects on the transcriptome

**Figure EV3:**
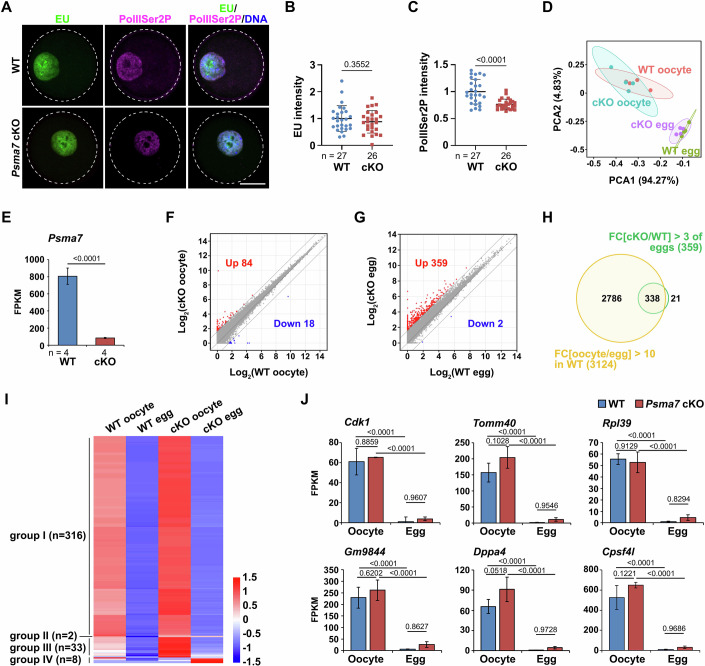
PSMA7 depletion has no significant effect on oocyte transcriptome. (**A**–**C**) Confocal analysis of EU and PolIISer2P signals (**A**), and quantification of relevant MFI (**B**, **C**) in WT and *Psma7* cKO GV oocytes. Scale bar, 20 μm. *n* indicates the number of oocytes analyzed. Mean and SD are shown. *P* values: unpaired *t* test. (**D**) PCA analysis of RNA-seq data from GV oocytes and eggs derived from WT and *Psma7* cKO mice. Results are analyzed using the SRplot web server. (**E**) RNA-seq results showing the relative expression levels of *Psma7* in GV oocytes of WT and *Psma7* cKO females. Analysis was conducted from four replicates of each genotype by RNA-seq. Mean and SD are shown. *P* values: unpaired *t* test. (**F**, **G**) Scatter plot depicting differentially expressed transcripts in *Psma7* cKO GV oocytes (**F**) and eggs (**G**) compared to WT controls. Transcripts with more than threefold change are highlighted in red (upregulated) or blue (downregulated). (**H**) Venn diagram displaying common transcripts that are downregulated during the GV–MII transition of WT groups and upregulated in *Psma7* cKO eggs. (**I**) Heat map displaying the expression of upregulated transcripts identified in panel (**G**) during the GV–MII transition in WT and *Psma7* cKO oocytes. Group I and III: transcripts upregulated in both GV oocytes and eggs after *Psma7* deletion, with Group III showing stronger upregulation in *Psma7* cKO GV oocytes. Group II: transcripts that should be downregulated during maturation remained stable after *Psma7* deletion. Group IV: genes upregulated in *Psma7* cKO eggs. Gene counts for each group are indicated. (**J**) RNA-seq results showing the relative expression levels of selected transcripts in GV oocytes and eggs of WT and *Psma7* cKO females. Four replicate results of each group by RNA-seq were used. Mean and SD are shown. *P* values: one-way ANOVA.

We evaluated global transcription activities in oocytes from PD13.5 WT and *Psma7* cKO mice—a timepoint characterized by the high global transcriptional activity—using 5’-ethynyl uridine (EU) incorporation and RNA polymerase II Ser2 phosphorylation (PolIISer2P) staining. While PolIISer2P signals were slightly decreased in *Psma7* cKO oocytes, EU intensity remained comparable to WT (Fig. EV3A–C). RNA-seq analysis of PD21 oocytes corroborated these findings. PCA clustering reflected differences in developmental stages rather than genotypes (Fig. EV3D). The expression of *Psma7* was indeed decreased in *Psma7* cKO oocytes (Fig. EV3E). However, only 84 transcripts were upregulated and 18 were downregulated (FC > 3) in *Psma7* cKO GV oocytes (Fig. EV3F). At the MII stage, 359 transcripts were upregulated and only 2 were downregulated in mutants (Fig. EV3G). Over 90% of upregulated transcripts in *Psma7* cKO eggs represented maternal mRNAs that normally undergo RNA decay during oocyte maturation (Fig. EV3H). Although these transcripts were slightly elevated in *Psma7* cKO eggs compared to WT, the difference was not statistically significant, and their abundance remained substantially lower than that in oocytes (Fig. EV3I,J), indicating that mRNA decay persists—though at a reduced efficiency—in the absence of PSMA7. Therefore, PSMA7 deficiency has limited impact on transcription and maternal mRNA clearance.

### PSMA7-deletion leads to defective oocyte maturation

**Figure 5:**
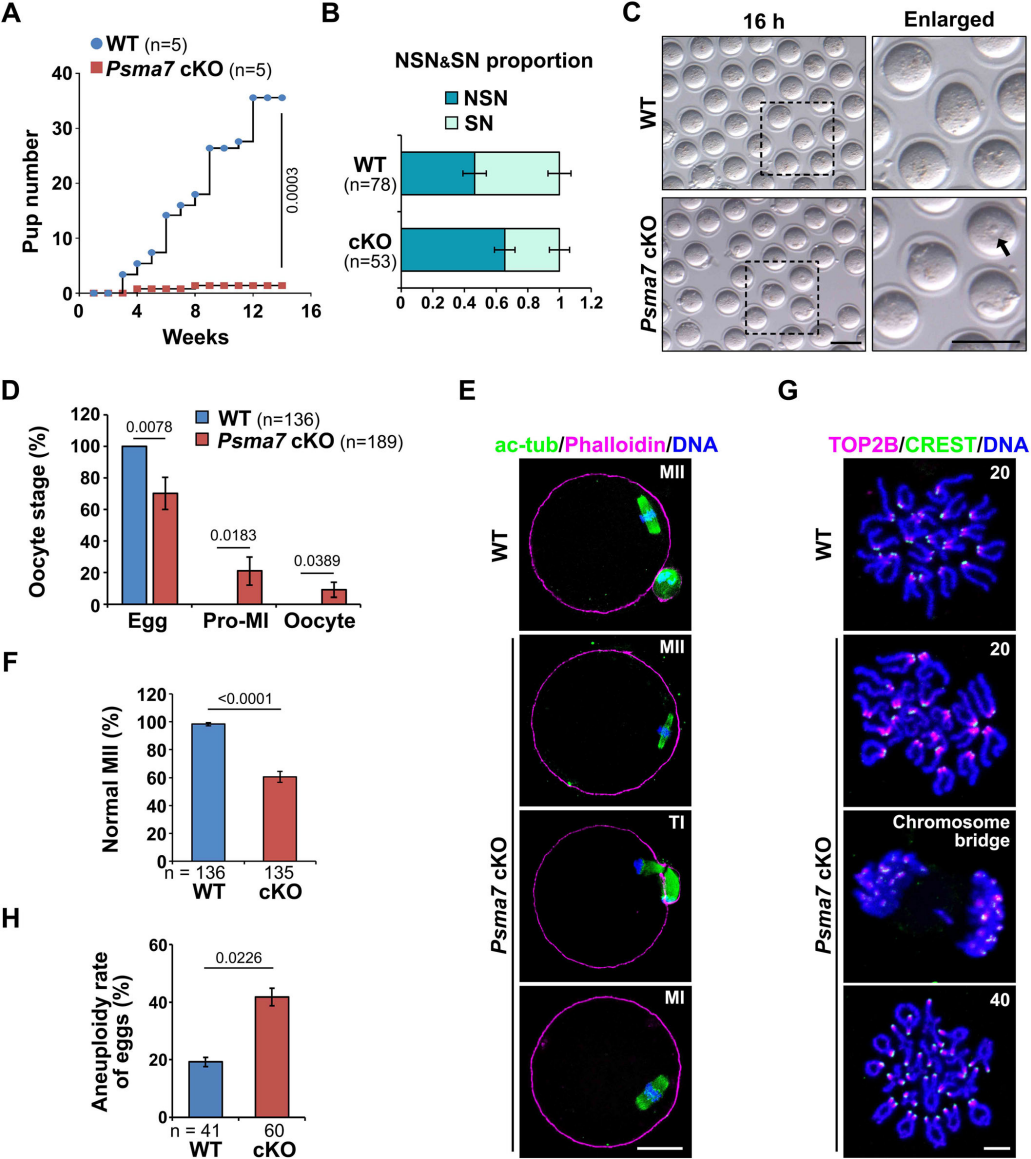
Impaired oocyte meiosis completion upon PSMA7 deletion. (**A**) Total number of pups per female mouse in WT and *Psma7* cKO groups. *n* indicates the number of mice analyzed. *P* values: unpaired *t* test. (**B**) Proportion of fully-grown oocytes with non-surrounded nucleolus (NSN) or surrounded nucleolus (SN) from PD21 WT and *Psma7* cKO females. SD is shown. (**C**) Representative images of oocytes collected from the oviducts of WT and *Psma7* cKO mice at 16 h post-hCG administration. The arrow indicates an oocyte arrested at the GV stage. Scale bars, 100 μm. (**D**) Developmental stage distribution of oocytes from WT and *Psma7* cKO mice. Mean and SD are shown. *P* values: unpaired *t* test. (**E**) Confocal analysis of acetyl-α-tubulin (ac-tub) shows spindle assembly and PB1 extrusion in WT and *Psma7* cKO oocytes. Scale bar, 20 μm. (**F**) Proportion of in vivo-matured eggs with normal spindle assembly in WT and *Psma7* cKO groups. Mean and SD are shown. *P* values: unpaired *t* test. (**G**) Immunofluorescent staining of TOP2B (chromosome arm marker) and CREST (centromere marker) on chromosome spreads of WT and *Psma7* cKO oocytes. The numbers of paired sister chromatids are indicated in the top right corner. Scale bar, 5 μm. (**H**) Aneuploidy rate in WT and *Psma7* cKO eggs. Mean and SD are shown. *P* values: unpaired *t* test. For panels (**D**, **F**, **H**), *n* indicates the number of oocytes analyzed. Source data are available online for this figure.

Adult *Psma7* cKO females exhibited severe subfertility over a 4-month fertility test, with only a minority producing one or two pups in isolated mating events (Fig. 5A). To investigate the underlying cause of this subfertility, we assessed the oocyte quality in *Psma7* cKO females. The proportion of non-surrounded nucleolus (NSN) oocytes to those with surrounded nucleolus (SN) was slightly elevated in *Psma7* cKO mice (PD21) compared to WT controls (Fig. 5B). Both in vivo and in vitro maturation assays revealed profound meiotic defects in *Psma7* cKO oocytes. After superovulation, only 70.2% of ovulated oocytes extruded the first polar body (PB1), while 20.9% failed to release PB1 and 8.9% even remained arrested at the GV stage (Fig. 5C,D). Immunofluorescent staining and confocal imaging confirmed that *Psma7* cKO oocytes were arrested at MI (Fig. 5E). Among those that extruded PB1, approximately 40% were arrested at telophase I (TI), displaying incomplete cytokinesis and chromosome bridges (Fig. 5E,F). In vitro maturation of *Psma7* cKO oocytes yielded similarly low rates of GVBD and PB1 extrusion (Appendix Fig. S4A,B), with developmental arrests at MI or TI (Appendix Fig. S4C,D). Notably, the rate of GVBD was more severely affected during in vitro maturation relative to that occurring in vivo (Fig. 5D; Appendix Fig. S4E).

Chromosome spreads stained for CREST and TOP2B showed that only 58.2% of *Psma7* cKO oocytes contained 20 normal pairs of sister chromatids, compared with 80.1% of WT oocytes (Fig. 5G,H). The rest *Psma7* cKO oocytes exhibited elevated aneuploidy rates, with chromosome bridges and unseparated chromosome masses (Fig. 5G,H), suggesting that PSMA7-deletion severely compromises chromosomal segregation and meiotic fidelity during oocyte maturation.

**Figure EV4:**
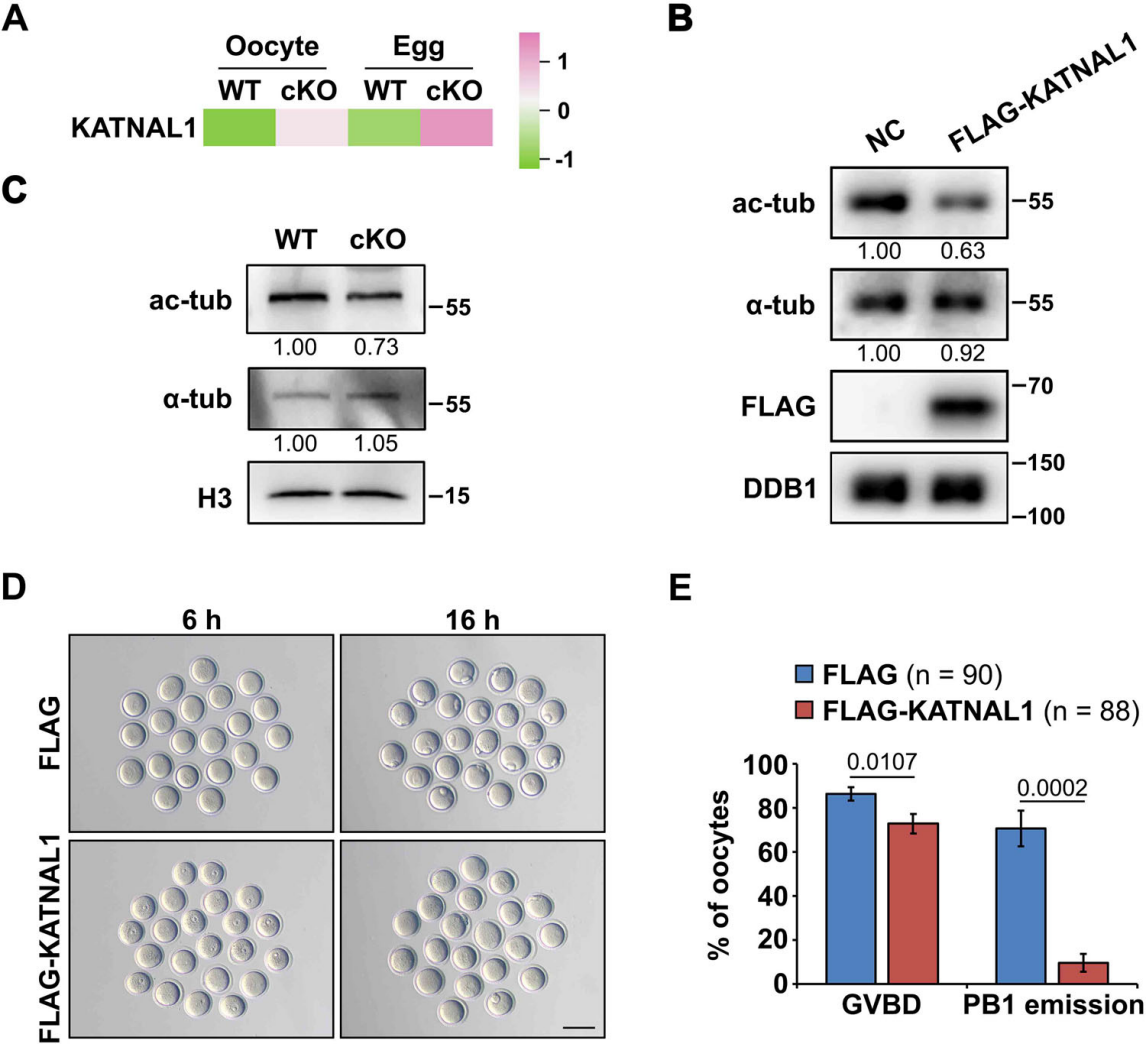
The accumulation of KATNAL1 leads to meiotic defects in *Psma7* cKO oocytes. (**A**) Heat map of KATNAL1 protein levels by proteomic analysis in WT and *Psma7* cKO oocytes. (**B**) Western blot analysis of acetylated α-tubulin (ac-tub) and total α-tubulin (α-tub) in control and overexpressing KATNAL1 eggs. (**C**) Western blot analysis of ac-tub and α-tub in WT and *Psma7* cKO eggs. DDB1 and H3 serve as the loading control. (**D**) Representative images of control and overexpressing KATNAL1 oocytes following in vitro culture. Scale bar, 100 μm. (**E**) Rates of GVBD and PB1 extrusion of oocytes cultured in vitro. n indicates the number of oocytes analyzed. Mean and SD are shown. *P* values: unpaired *t* test.

Proteomic analysis revealed the upregulation of Katanin p60 ATPase-containing subunit A-like 1 (KATNAL1) following PSMA7 depletion (Fig. EV4A). As a microtubule-severing protein essential for microtubule reorganization, KATNAL1 deficiency is known to cause spindle defects and delayed meiosis in oocytes (Gao et al, 2019; Lynn et al, 2021; Ververis et al, 2016). Consistent with this role, KATNAL1 overexpression specifically disrupted microtubule stability in oocytes, as evidenced by a decrease in acetylated-α-tubulin level (Ververis et al, 2016) (Fig. EV4B). Notably, this phenotype was recapitulated in *Psma7* cKO oocytes (Fig. EV4C), which consequently exhibited significantly reduced rates of GVBD and PB1 extrusion during in vitro culture (Fig. EV4D,E). In all, these findings suggest the accumulation of KATNAL1 likely contributes, at least in part, to the meiotic defects in *Psma7* cKO oocytes.

### Maternal PSMA7 deletion causes embryonic arrest at the 1- or 2-cell stage

**Figure 6:**
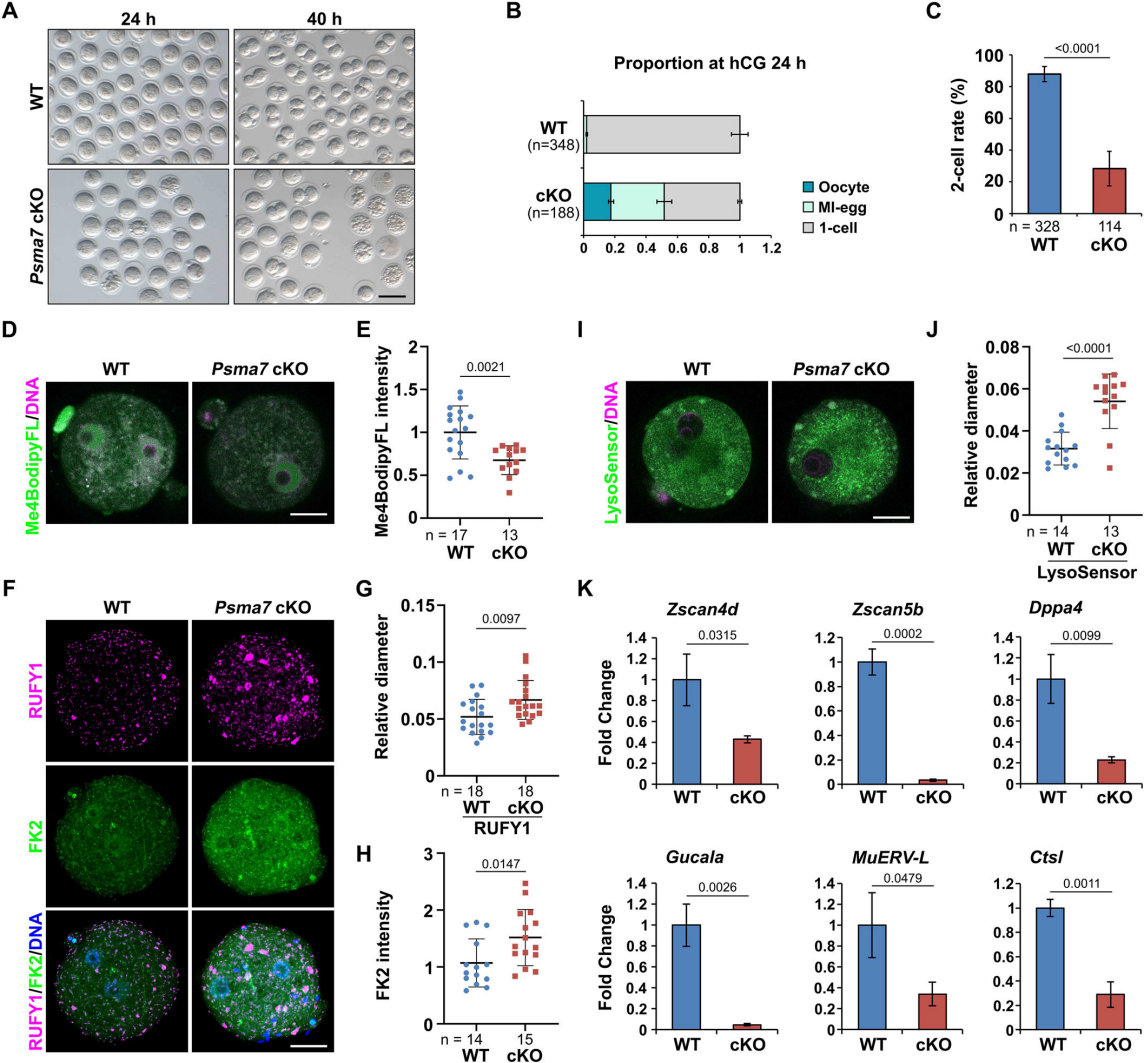
Maternal PSMA7 deletion results in embryonic developmental arrest at the 1-cell or 2-cell stage. (**A**) Representative images of embryos collected from WT and *Psma7* cKO mice at 24 h and 40 h post-hCG administration. Scale bar, 100 μm. (**B**) Developmental stage distribution of embryos collected from the oviducts of WT and *Psma7* cKO mice at 24 h post-hCG. SD is shown. (**C**) 2-cell rate of embryos from WT and *Psma7* cKO mice at 40 h post-hCG. Mean and SD are shown. *P* values: unpaired *t* test. (**D**, **E**) Confocal analysis of Me4BodipyFL signal (**D**) and quantification of the MFI (**E**) in zygotes from WT and *Psma7* cKO mice. Scale bar, 20 μm. Mean and SD are shown. *P* values: unpaired *t* test. (**F**) Immunofluorescent staining of RUFY1 and FK2 in WT and *Psma7* cKO zygotes. (**G**, **H**) Quantification of the relative diameter of RUFY1 compartments (**G**) and the MFI of FK2 signal (**H**) in (**F**). Mean and SD are shown. *P* values: unpaired *t* test. (**I**, **J**) Confocal analysis with LysoSensor probe (**I**) and quantification of the relative diameter of the compartments (**J**) in zygotes from WT and *Psma7* cKO females. Mean and SD are shown. *P* values: unpaired *t* test. (**K**) Relative expression levels of indicated ZGA genes in 2-cell stage embryos from WT and *Psma7* cKO females. Data are shown as mean of three independent trials with three biological replicates. Mean and SD are shown. *P* values: unpaired *t* test. For all statistical analysis, *n* indicates the number of eggs or embryos analyzed. Source data are available online for this figure.

We next asked whether the observed protein homeostasis defects in oocytes affect early embryonic development post-fertilization. After mating superovulated *Psma7* cKO females with WT males, embryos were collected at 24 h (1-cell stage) or 40 h (2-cell stage) post hCG administration. Although approximately half of *Psma7* cKO oocytes were fertilized and formed zygotes, only 28.3% developed to the 2-cell stage (Fig. 6A–C). In vitro fertilization experiments corroborated these results, with *Psma7*^*♀–/♂+*^ embryos consistently arresting at the 1- or 2-cell stage (Appendix Fig. S5A). In addition, *Psma7*^*♀–/♂+*^ zygotes showed reduced PSMA7 levels and proteasomal activity (Fig. 6D,E; Appendix Fig. S5B,C), accompanied by accumulation of ubiquitinated proteins (FK2 signal) and protein aggregates (Proteostat signal) (Fig. 6F,H; Appendix Fig. S5D,E). ELVAs indicators—RUFY1, LysoSensor and LAMP1—formed abnormally large puncta that failed to translocate to the cortex (Fig. 6F,G,I,J; Appendix Fig. S5D,F), mirroring defects observed in *Psma7* cKO oocytes.

**Figure EV5:**
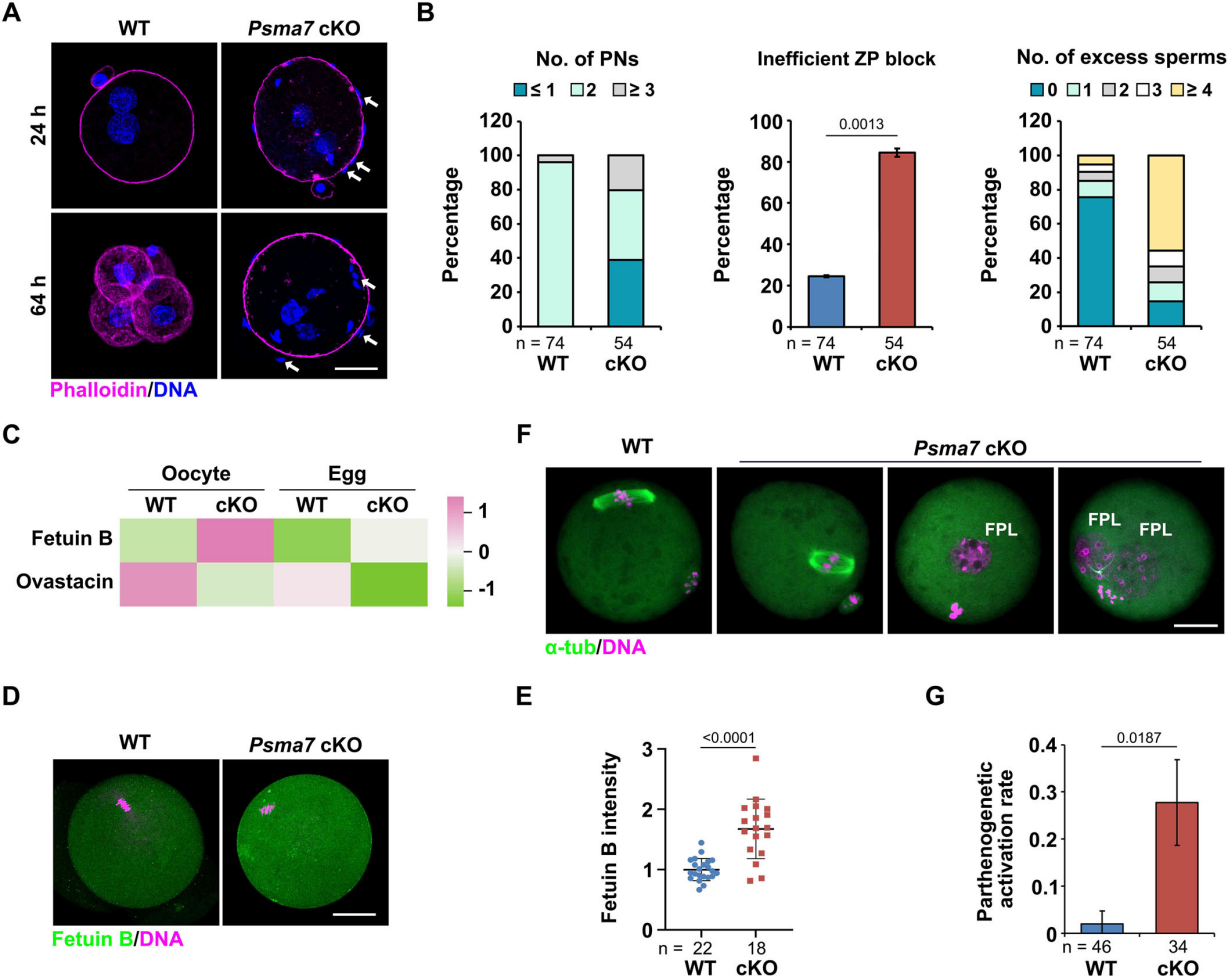
Maternal PSMA7 deficiency leads to polyspermy and parthenogenetic activation in oocytes. (**A**) Confocal analysis of embryos following IVF at 24 h and 64 h post-hCG. The arrows indicate excess sperms adhering to the cortex. (**B**) Quantification of pronuclear count, inefficient zona pellucida block rate, and excess sperm count in zygotes 8 h post-IVF. Mean and SD are shown in the middle image. *P* values: unpaired *t* test. (**C**) Heat map of Fetuin B and Ovastacin protein levels in WT and *Psma7* cKO oocytes. (**D**, **E**) Confocal analysis of Fetuin B signal (**D**) and quantification of the MFI (**E**) in WT and *Psma7* cKO eggs. Scale bar, 20 μm. Mean and SD are shown. *P* values: unpaired *t* test. (**F**) Fluorescence images showing normal and female pronucleus-like (FPL) structures in WT and *Psma7* cKO eggs after 48 h of in vitro culture. Scale bar, 20 μm. (**G**) Parthenogenetic activation rates in WT and *Psma7* cKO eggs in (**F**). Mean and SD are shown. *P* values: unpaired *t* test.

Following IVF, extra sperm persisted in *Psma7*^*♀–/♂+*^ embryos at both 24 h and 64 h post-hCG (Fig. EV5A; Appendix Fig. S5A). At 24 h, only 40.7% of *Psma7*^*♀–/♂+*^ zygotes formed two normal pronuclei, whereas 38.9% exhibited pronuclear deficiency, and 20.4% showed polyspermy (≥ 3 pronuclei); this was associated with increased zona pellucida penetration rate and elevated cortical sperm counts (Fig. EV5B). Consistent with these fertilization defects, maternal PSMA7 depletion led to an accumulation of Fetuin B, which is a key inhibitor of the zona pellucida hardening protease Ovastacin (Dietzel et al, 2013), and a decline in Ovastacin levels (Fig. EV5C–E). This molecular dysregulation underlies the defective polyspermy block. Furthermore, after extended culture, 27.7% of *Psma7* cKO eggs spontaneously formed female pronucleus-like (FPL) structures (Fig. EV5F,G), indicating loss of cytostatic factor activity and impaired MII arrest maintenance. Consequently, all *Psma7*^*♀–/♂+*^ embryos arrested before the 2-cell stage. The arrest coincided with defective ZGA, as evidenced by the downregulation of totipotency markers (*Zscan4d*, *Zscan5b*, *MuERV-L*) and early zygotic genes (*Dppa4*, *Gucala* and *Ctsl*) (Fig. 6K) (Chen et al, 2021; Macfarlan et al, 2012; Sharma et al, 2024; Wei et al, 2024; Zhang et al, 2019b).

In summary, abnormalities in meiotic progression during oocyte maturation and insufficient ZGA in early embryonic development, stemming from defective protein degradation, collectively account for the subfertility of oocyte-specific *Psma7*-deleted females.

## Discussion

Here, we present a detailed analysis of the physiological function of 20S proteasome in regulating ELVAs and female fertility, using an oocyte-specific PSMA7 knockout mouse model. As a core subunit of the long-lived proteasome complex (Harasimov et al, 2024), PSMA7 is highly abundant in oocytes, starting from the primordial follicle stage. Notably, even after *Psma7* knockout induced by Zp3-Cre at the primary follicle stage, residual PSMA7 protein persists throughout oocyte maturation and remains detectable in fertilized eggs. This retention underscores the exceptional stability of PSMA7 and implies the functional significance of proteasome-mediated degradation. Such partial loss is physiologically relevant, as poor-quality oocytes in clinical settings often exhibit reduced, rather than completely absent, proteasomal activity. Previous studies have largely relied on proteasomal inhibitors (Harasimov et al, 2024; Mailhes et al, 2002; Zaffagnini et al, 2024), which completely suppress proteasomal activity and block oocyte maturation, thereby limiting physiological relevance. Our model thus offers a more nuanced tool to investigate how a partial decline in proteasomal activity affects oocyte competence and subsequent embryonic development.

**Figure 7:**
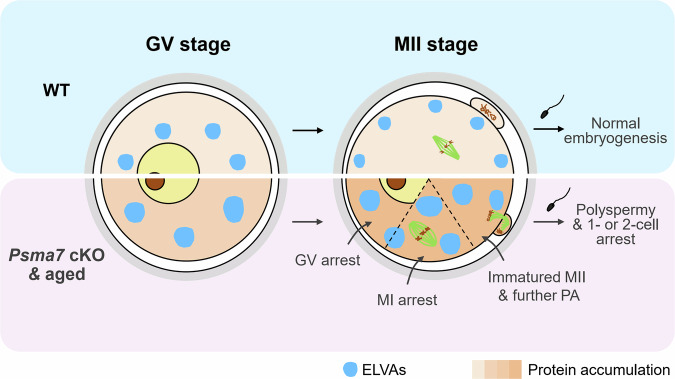
A diagram illustrating the function of 20S proteasome in oocyte maturation, OET and aging. In aged oocytes, decreased expression of 20S proteasome subunits coincides with the accumulation of protein aggregates. This proteasomal decline is recapitulated in *Psma7*^*fl/fl*^*;Zp3-Cre* females, where PSMA7 deficiency leads to reduced 20S proteasome abundance and impaired ELVAs translocation. Consequently, protein degradation and translation during OET are compromised, resulting in nearly complete infertility. Oocyte maturation is severely impaired, characterized by abnormal spindles, misaligned chromosomes, and polyspermy or partial parthenogenetic activation. Early embryos subsequently arrest predominantly at the 1- or 2-cell stage, primarily due to ZGA failure. Taken together, PSMA7 is essential for 20S proteasome assembly and ELVAs translocation in mouse oocytes, mediating maternal protein degradation during maturation to support post-fertilization embryonic development.

Despite the presence of residual maternal PSMA7, *Psma7*^*fl/fl*^*;Zp3-Cre* female mice exhibit near-complete infertility. Their oocytes and embryos display multi-stage developmental defects, including meiotic abnormalities such as aberrant spindle assembly and incomplete chromosome segregation during meiosis I, polyspermy, spontaneous parthenogenetic activation and post-fertilization arrest at the 1- to 2-cell stage. These defects originate from PSMA7 deficiency-induced disassembly of the 20S proteasome, which disrupts protein degradation in both maturing oocytes and zygotes (Fig. 7). Specifically, PSMA7-deficient oocytes display enlarged ELVAs that fail to translocate to the cortex, accompanied by heightened lysosomal activity, accumulation of protein aggregates and increased protein ubiquitination. Notably, oocytes from aged mice and humans also show enlarged ELVAs, suggesting that declining proteasomal activity may represent a critical mechanism underlying mammalian oocyte aging. Slightly different from PSMA7 deficiency, advanced age has a relatively minor effect on ELVAs migration to the oocyte cortex, which may be due to the fact that the reduction in PSMA7 and 20S proteasome levels in aged oocytes is less pronounced than that observed in PSMA7 knockout oocytes (Figs. 1G and 2B). More severe phenotypes might become apparent at older ages.

As long-lived and non-proliferative cells, oocytes face unique challenges in maintaining proteostasis, since they cannot dilute protein aggregates through cell division (Hipp et al, 2019; Sala and Morimoto, 2022). ELVAs, specialized degradative super-organelles, are known to sequester detrimental protein aggregates in immature oocytes and facilitate their degradation during maturation, thereby ensuring the high quality of ooplasm sufficient to support embryonic development (Charalambous et al, 2023; Zaffagnini et al, 2024). Under normal conditions, proteasomal activity gradually increases during oocyte maturation, coordinated with ELVAs activation. Maternal PSMA7 deficiency disrupts this process, leading to reduced proteasomal activity and accumulated protein aggregates, especially at the MII stage, consistent with the previous report that proteasomes are critical for ELVAs-mediated protein degradation (Zaffagnini et al, 2024). Further analysis reveals that although ELVAs assembly, marked by RUFY1-positive compartments, is unchanged, their puncta become enlarged and fail to fully translocate to the cortex upon PSMA7 deletion. Importantly, decreased proteasomal activity triggers compensatory lysosomal overactivation, as confirmed both *in Psma7* cKO oocytes and in MG132-treated samples.

Degradation of maternal factors is crucial for OET during early embryogenesis (Yu et al, 2016). Understanding the patterns, regulation and physiological significance of maternal protein degradation has long been a challenge in reproductive biology. Proteasome subunits are highly abundant in oocytes and are essential for proteostasis through their assembly into ELVAs. In the absence of PSMA7, protein levels of 20S subunits are significantly reduced, while 19S subunits remain largely unaffected, indicating that PSMA7 deficiency primarily disrupts 20S proteasome integrity. Leveraging advanced microscale proteomics, we obtained high-quality proteomic profiles with minimal input. Our analysis reveals that PSMA7 deficiency results in progressive protein accumulation as oocytes mature.

Oocyte maturation involves extensive protein synthesis and degradation (Zaffagnini et al, 2025). Our proteomic data indicate that proteins upregulated during normal maturation are mainly associated with cell cycle regulation, chromosome organization and segregation, spindle assembly and epigenetic reprogramming. Downregulated proteins are linked to transcription, ribosome biogenesis, phosphorylation and intermediate filament assembly. Especially, intermediate filament organization-related Keratins are PSMA7-dependent substrates, indicating that compromised structural support and stability of cells may impair actin/RAB11A-dependent ELVAs relocation in PSMA7 deficiency oocytes. Furthermore, global translational activity is impaired following both PSMA7 depletion and MG132 treatment, suggesting that defective protein degradation adversely affects de novo protein synthesis. Interestingly, these substantial proteomic alternations have minimal impact transcriptional activity and the transcriptome in oocytes. Given that fully-grown oocytes are transcriptionally quiescent (Hamazaki et al, 2021; Jiang et al, 2023a), it is not surprising that protein-level changes in PSMA7-deficient oocytes have limited effects on the transcriptome. However, ZGA is significantly impaired in embryos derived from *Psma7* cKO oocytes, likely due to defective degradation of maternal mRNAs and proteins in nascent zygotes.

While ELVAs capture aggregated proteins in immature oocytes and promote their degradation during maturation, proteostasis is already disrupted in *Psma7* cKO GV oocytes. PSMA7 and PSMC2 show prominent nuclear localization (Figs. 2C and EV1A,B), in contrast to aggregated proteins. Moreover, the cytoplasmic distribution of PSMA7 and other 20S subunits does not completely overlap with ELVAs localization. Thus, although proteasome activity is higher within ELVAs than that in other regions (Zaffagnini et al, 2024), our data do not exclude the possibility of ELVAs-independent functions of the 20S proteasome in oocytes.

Female reproductive aging has emerged as a pressing public health concern, particularly as women worldwide delay childbearing (Benzies, 2008; Carolan and Frankowska, 2011). Age-related decline in oocyte quality adversely affects ovarian reserve and comprises early embryonic developmental competence, resulting in elevated rates of miscarriage, birth defects, and infertility (MacLennan et al, 2015; Mihalas et al, 2024). Our results demonstrate that oocytes with deficient proteasomal activity exhibit maturation defects or severe developmental abnormalities. Importantly, poor-quality oocytes, particularly those from aged females, frequently display decreased proteasomal activity, although the underlying mechanisms remain poorly understood. We reveal the reduction of proteasome levels and activity in aged oocytes, associated with accumulation of ubiquitinated proteins and expansion of lysosomal puncta. The supplementation of PSMA7 or 20S strategy is theoretically feasible. However, the decline of oocyte quality in advanced age is a multi-factorial, multi-level, and progressive systemic collapse process, involving decreased transcriptional level, aberrant epigenomics, organelle dysfunction, metabolic decline and impaired protein degradation mentioned here. This perspective underscores the importance of preventive rather than restorative interventions for age-related fertility decline. The design and application of 20S small molecule activators represents a promising rescue direction. These findings establish a direct link between impaired proteasome function and age-related decline in oocyte and embryo quality. In summary, elucidating the roles of proteasomes in oocytes and early embryos may provide critical insights for diagnosis and treatment of clinical conditions such as oocyte maturation arrest, poor oocyte quality and recurrent early embryonic arrest.

## Methods

**Table:** Reagents and tools table

Reagent/resource	Reference or source	Identifier or catalog number
**Experimental models**		
C57BL/6J (*M.* *musculus*)	Jackson Lab	B6.129P2Gpr37tm1Dgen/J
**Recombinant DNA**		
**Antibodies**		
Anti-PSMA7	Abcam	ab133502
Anti-PSMC2	Proteintech	14905-1-AP
Anti-20S Proteasome α1, 2, 3, 5, 6, & 7-Subunits (α-sub)	Merck	ST1049
Anti-LAMP1	Cell Signaling Abcam	99437 ab24170
Anti-RUFY1	Proteintech	13498-1-AP
Anti-Ubiquitin conjugates (FK2)	Ubiquigent	68-0121-500
Anti-Acetyl-α-tubulin	Cell Signaling	5335
Anti-FITC-α-tubulin	Sigma	F2168
Anti-TOP2B	Abcam	ab109524
Anti-CREST	Fitzgerald Industries International	70R-21494
Anti-PolIISer2P	Abcam	ab5095
Anti-Ubiquitin	Cell Signaling	3936
Anti-MVH	Abcam	ab13840
Anti-cyclin B1	Cell Signaling	4138
Anti-Fetuin B	Proteintech	18052-1-AP
Anti-pERK1/2	Cell Signaling	9101
Anti-DDB1	Epitomics	3821-1
Anti-GAPDH	Proteintech	60004-1-Ig
Goat Anti-rabbit IgG H&L (HRP)	Abcam	Ab6721
Rabbit Anti-mouse IgG H&L (HRP)	Abcam	Ab6728
Goat anti-Mouse IgG (H + L), Alexa Fluor Plus 488	Thermo Scientific	A11001
Goat anti-Rabbit IgG (H + L), Alexa Fluor Plus 568	Thermo Scientific	A11011
**Oligonucleotides and other sequence-based reagents**		
RT-PCR primers	This study	Table EV2
**Chemicals, enzymes and other reagents**		
Me4BodipyFL-Ahx3Leu3VS	Bio-Techne	I–190
LysoSensor Green DND-189	Meilunstar	MB6043
Proteostat	Enzo	ENZ-51035-K100
MG132	MerckMillipore	474790
BAFA1	Abcam	ab120497
HPG Alexa Fluor^®^ Protein Synthesis Assay Kits	Invitrogen	C10428
Click-iT^®^ RNA Imaging Kits	Invitrogen	C10329
**Software**		
**Other**		

### Mice

Mice (C57BL/6) carrying the floxed allele of *Psma7* (*Psma7*^*fl*^) were generated via CRISPR-Cas9 technique by GemPharmatech Co., Ltd and were published previously (Fang et al, 2025). To achieve oocyte-specific knockout, *Psma7*^*fl/fl*^ mice were crossed with *Zp3-Cre* transgenic mice (Jiao et al, 2025; Liu et al, 2019; Wu et al, 2025; Yu et al, 2017; Yu et al, 2013). This breeding strategy induced a large fragment deletion of *Psma7* gene in oocytes at the primary follicle stage. Female C57BL/6 mice aged 10–12 months were used as a natural aging model. All mice were housed under SPF conditions in a controlled environment maintained at 20–22 °C, with a 12-h light/dark cycle, 50–70% humidity, and ad libitum access to food and water. Animal care and experimental procedures were conducted in accordance with the guidelines approved by the Laboratory Animal Welfare and Ethics Committee of Zhejiang University (NO. ZJU20220410). No blinding was performed in this study.

### Mouse oocyte collection and in vivo maturation (IVM)

Female mice aged 21–23 days were intraperitoneally injected with 5 IU of PMSG and humanely euthanized 44 h post-injection. Germinal vesicle (GV) stage oocytes were harvested in M2 medium (Sigma-Aldrich, M7167) and subsequently cultured in microdroplets of M16 medium (Sigma-Aldrich, M7292) under mineral oil (Sigma-Aldrich, M5310) at 37 °C in 5% CO_2_ atmosphere. The germinal vesicle breakdown (GVBD) rate and the first polar body (PB1) extrusion rate were evaluated at 4 h or 16 h after in vitro culture. Where indicated, 2.5 μM milrinone was added into the culture medium to prevent spontaneous GVBD.

### Human oocyte collection

All metaphase II (MII)-stage human eggs used in this study were generously donated by volunteers undergoing oocyte retrieval for IVF/ICSI treatment at the Center of Reproductive Medicine, Sir Run Run Shaw Hospital. Ovarian stimulation was performed using GnRH analogues in combination with recombinant follicle-stimulating hormone (FSH). Follicular puncture was carried out 36 h after hCG administration. A total of 32 participants were recruited, stratified into two age groups (< 28 years and >38 years). All donors were fully informed of the study purpose prior to participation. Oocytes were randomly selected from the donated pool without affecting clinical outcomes. Participant confidentiality and data security were strictly maintained throughout the study. The research protocol was reviewed and approved by the Ethics Committee of Sir Run Run Shaw Hospital.

### Superovulation and fertilization

Female mice aged 21–23 days were intraperitoneally injected with 5 IU PMSG, followed by injection of 5 IU hCG 44 h later. Eggs were collected from the oviducts 16 h post-hCG injection in M2 medium and treated with 0.3% hyaluronidase to remove cumulus cells. For preimplantation embryo collection, superovulated females were mated with 10–12-week-old wild-type (WT) male mice. Successful mating was confirmed by the presence of a vaginal plug. Embryos at 1-cell, 2-cell and 4-cell stages were isolated from the oviducts at 24, 40, and 64 h post-hCG injection, respectively.

### In vitro fertilization (IVF) and embryo culture

On the day of IVF, caudal epididymides and vas deferens were dissected from WT male mice and placed in HTF medium to facilitate sperm capacitation. Cumulus-oocyte complexes (COCs) were collected 16 h post-hCG administration from superovulated WT and *Psma7*^*fl/fl*^*;Zp3-Cre* female mice and incubated in HTF medium. After 1 h of pre-incubation, capacitated spermatozoa were introduced to the COCs. Following 6 h of co-incubation, presumptive zygotes were washed and transferred to KSOM medium (Aibei, M1435) for subsequent embryo culture at 37 °C in 5% CO_2_ atmosphere.

### Histological analysis

Ovarian tissues were dissected and fixed overnight with 4% PFA in PBS, then processed through standard dehydration, paraffin embedding, and serial sectioning at 5 μm thickness. Sections were stained with hematoxylin and eosin (H&E) for morphological analysis. Immunohistochemistry (IHC) was performed following established protocols (Yu et al, 2013). The antibodies applied are listed in Table EV1.

### Western blot

For protein analysis, oocytes were lysed in SDS sample buffer and denatured at 95 °C for 10 min. Protein lysates equivalent to 100 oocytes per lane were separated by SDS-PAGE using a Mini-PROTEAN Tetra Cell System (Bio-Rad) and transferred to PVDF membranes (Millipore, Billerica, MA, USA) following the manufacturer’s instructions. Immunoblotting was performed according to established procedures (Cao et al, 2025). Protein levels of targets and loading controls were compared through statistical analysis on the band intensities with ImageJ. The primary and secondary antibodies used are listed in Table EV1.

### Fluorescent staining

For immunofluorescent staining, oocytes and zygotes were fixed with 4% PFA in PBS for 30 min, permeabilized with 0.3% Triton X-100 in PBS for 20 min, and blocked with 1% BSA in PBS to minimize nonspecific binding. Samples were incubated with primary antibodies for 1 h, followed by incubation with Alexa Fluor 594- or 488-conjugated secondary antibodies containing DAPI for 30 min. The antibodies used are listed in Table EV1. For Me4BodipyFL-Ahx3Leu3VS (Bio-techne, I–190) labelling, oocytes were incubated with 2 μM fluorescent probe for 30–60 min at 37 °C before imaging. For LysoSensor^TM^ Green DND-189 (MeilunBio, MB6043) labelling, oocytes were incubated with 1 μM fluorescent probe for at 30–60 min before imaging. All images were acquired on a LSM800 confocal microscope (Zeiss) with single optical sections taken at the equatorial plane. Each experiment was repeated at least three times and representative results are shown in the article.

### Quantitative analysis of relative diameter, cortical distance and volume of ELVAs

As mentioned above, the maximum cross-section of each oocyte was selected for image acquisition to ensure consistency in section or focal plane selection. The diameter of each ELVA with the maximum cross-section was measured and divided by the diameter of the corresponding oocyte to obtain the relative diameter of that ELVA. The average relative diameter was then calculated for each oocyte, and statistical comparisons were performed across more than ten oocytes. The distance from the center of each ELVA to the oocyte cortex was measured and divided by the oocyte radius to obtain the relative cortical distance of that ELVA. The average relative distance from cortex of all ELVAs was calculated for each individual oocyte, followed by statistical analysis and inter-group comparison across multiple oocytes. For 3D models, oocytes were placed in droplets on a glass-bottom 35 mm dish covered with mineral oil and imaged with a confocal microscope. Z-stack was taken encompassing the entire oocyte with 31 slices. ELVA volume was quantified with the reported custom-made FIJI macro (Zaffagnini et al, 2024), available in Zenodo: 10.5281/zenodo.10446149. The top ten largest ELVAs by volume were quantified and averaged to represent the single value per oocyte, followed by statistical analysis and inter-group comparison across multiple oocytes.

### In vitro mRNA synthesis and microinjection

Following linearization of the plasmids with appropriate restriction enzymes, 5’-capped mRNAs were synthesized using the mMESSAGE mMACHINE^TM^ SP6 Transcription Kit (Invitrogen, AM1340), followed by polyadenylation with the Poly (A) Tailing Kit (Invitrogen, AM1350). The synthesized mRNAs were then purified by lithium chloride precipitation and resuspended in nuclease-free water. Oocytes were collected in M2 medium supplemented with 2.5 μM milrinone to inhibit spontaneous GVBD. Approximately 10 pl of the synthetic mRNAs (∼500 g/ml) were microinjected into the ooplasm using an Eppendorf TransferMan NK2 micromanipulator.

### Detection of transcription and translation activity

Oocytes were cultured in M16 medium containing 1 mM 5-ethynyl uridine (EU) and 50 μM homopropargylglycine (HPG) for 1 h to label newly synthesized RNA and proteins, respectively. After incubation, samples were fixed in 4% PFA for 30 min. EU incorporation was detected using the Click-iT® RNA Alexa Fluor® 488 Imaging Kit (Life Technologies), and HPG incorporation was visualized with the Click-iT® HPG Alexa Fluor® Protein Synthesis Assay Kit (Life Technologies), according to the manufacturer’s instructions.

### Oocyte micro-proteomics

After removal of the zona pellucida, 130 oocytes per sample were washed three times in ice-cold PBS and stored at −80 °C. As reported (Zhang et al, 2023a), oocytes were lysed in a buffer containing 20 mM Tris–HCl (pH 8.5), 1% sodium deoxycholate, 10 mM TCEP, and 40 mM 2-chloroacetamide, boiled for 5 min, and sonicated to denature proteins and shear genomic DNA. Proteins were digested with trypsin and Lys-C at a 1:50 (w/w) ratio, followed by desalting using SDB-RPS StageTips. Peptides were dried in a SpeedVac concentrator, reconstituted in 0.1% formic acid, and analyzed by LC-MS/MS on a Thermo Scientific Orbitrap Ascend Tribrid mass spectrometer. Data were processed with Spectronaut software and searched against the UniProt Mouse database.

### RNA-seq library preparation and gene expression analysis

As previously described (Rong et al, 2019), total RNA was extracted from ten embryos per sample collected from WT and *Psma7*^*fl/fl*^*;Zp3-Cre* female mice. Each sample was lysed in 4 μL of lysis buffer containing 0.2% Triton X-100, RNase inhibitor, dNTPs, oligo-dT primers, and ERCC mRNA spike-in mix. cDNA was synthesized following the Smart-seq2 protocol. Gene expression levels were quantified in fragments per kilobase of exon per million mapped fragments (FPKM) using Cufflinks (v2.2.1). For downstream analyses, FPKM values below 1 were set to 1.

### Real-time quantitative PCR (RT-qPCR)

RT-qPCR analysis was performed using a modified RNA Smart-seq protocol for low-input samples. In brief, five embryos isolated from WT or *Psma7*^*fl/fl*^*; Zp3-Cre* female mice were lysed in 2 μL of lysis buffer containing 0.2% Triton X-100, 2 IU/μl RNase inhibitor, dNTPs and oligo-dT primers. Reverse transcription was carried out using SuperScript III reverse transcriptase (Invitrogen). The resulting cDNA was diluted and amplified with Power SYBR Green PCR Master Mix (Applied Biosystems) on an ABI 7500 Real-Time PCR system (Applied Biosystems). Relative mRNA expression was calculated using the 2^-ΔΔCT^ method, with normalization to *Gapdh* mRNA. The primer sequences are listed in Table EV2.

### Statistical analysis

Quantitative data were presented as mean ± SD. All experiments were repeated in at least three independent biological replicates, with one representative dataset presented. Comparisons between two groups were evaluated using two-tailed unpaired Student’s *t* tests. For experiments involving more than two groups, one-way ANOVA was used. The exact *P* values were provided in the figures.

### Ethics statements

This study was approved by the Ethics Committee of Sir Run Run Shaw Hospital, Zhejiang University School of Medicine (No. SRRSH20220461). Animal experiments were approved by the Laboratory Animal Welfare and Ethics Committee of Zhejiang University (NO. ZJU20220410). It is confirmed that this study meets the ethical guidelines outlined in the journal’s Author Guidelines, including adherence to the legal requirements of the study country.

## Supplementary information

Appendix

Table EV1

Table EV2

Peer Review File

Source data Fig. 1

Source data Fig. 2

Source data Fig. 3

Source data Fig. 4

Source data Fig. 5

Source data Fig. 6

Expanded View Figures

## Data Availability

RNA-seq data were deposited in the NCBI Gene Expression Omnibus database with the accession codes GSE293047 (https://www.ncbi.nlm.nih.gov/geo/query/acc.cgi). Micro-proteomic data were deposited in the IProX proteome database with the accession codes PXD062494 (https://proteomecentral.proteomexchange.org/ui?pxid=PXD062494). The source data of this paper are collected in the following database record: biostudies:S-SCDT-10_1038-S44318-026-00813-0.
