# Supplementary material for: 20S proteasome-regulated proteostasis in ELVAs is critical for oocyte-to-embryo transition and female fertility
Source: EMBO J. 2026 May 21;45(14):4887–909. doi: 10.1038/s44318-026-00813-0 (PMC13373198; doi:10.1038/s44318-026-00813-0)
Supplement: Supplementary file 1 — Appendix [file 44318_2026_813_MOESM1_ESM.pdf]

APPENDIX

20S Proteasome-Regulated Proteostasis in ELVAs is Critical for Oocyte-to-Embryo Transition and Female Fertility

Table Of Contents

Appendix Figure S1	Page 2
Appendix Figure S2	Page 3
Appendix Figure S3	Page 4
Appendix Figure S4	Page 5
Appendix Figure S5	Page 6

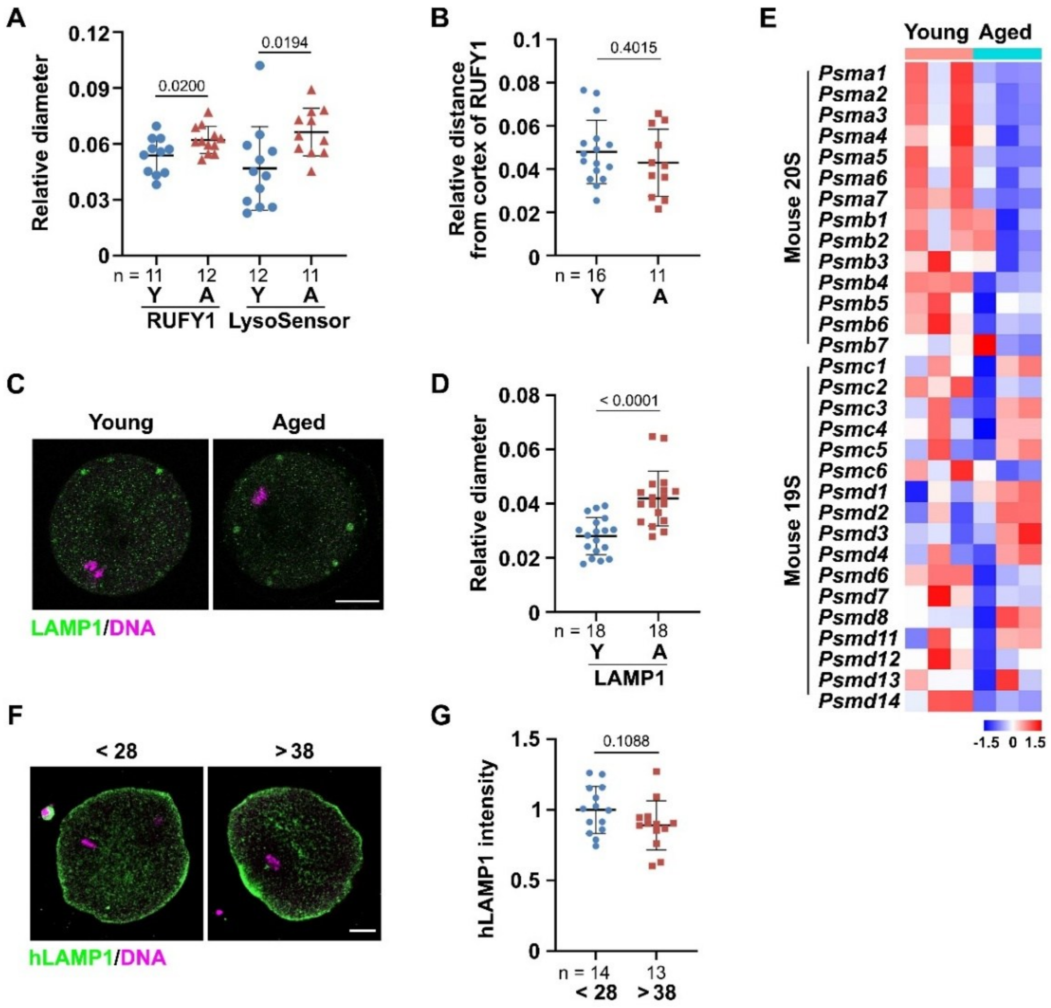

Appendix Figure S1. Enlarged ELVA compartments exist in aged mouse eggs.

(A) Quantification of the relative diameter of indicated RUFY1- and LysoSensor-compartments (normalized to the diameter of eggs) in young and aged mouse eggs of Fig. 1C. Mean and SD are shown. p values: unpaired t test. **(B)** Quantification of the relative cortical distance of RUFY1-positive compartments (normalized to the diameter of eggs) in young and aged mouse eggs. Mean and SD are shown. p values: unpaired t test. **(C)** Immunofluorescent staining of LAMP1 in young and aged mouse eggs. Scale bar, 20 µm. **(D)** Quantification of the relative diameter of LAMP1-positive compartments in panel **(C)**. Mean and SD are shown. p values: unpaired t test. **(E)** Heat map illustrating the differential expression profiles of proteasome subunits in young and aged mouse GV oocytes. Data were reanalyzed from Zhang et al., *Nature Aging*, 2023 (PMID: 37845508). **(F)** Confocal analysis of hLAMP1 signal in human eggs of indicated age groups. Scale bar, 20 µm. **(G)** Quantification of MFI in panel **(F)**. Mean and SD are shown. p values: unpaired t test. For all statistical analysis, n indicates the number of oocytes analyzed.

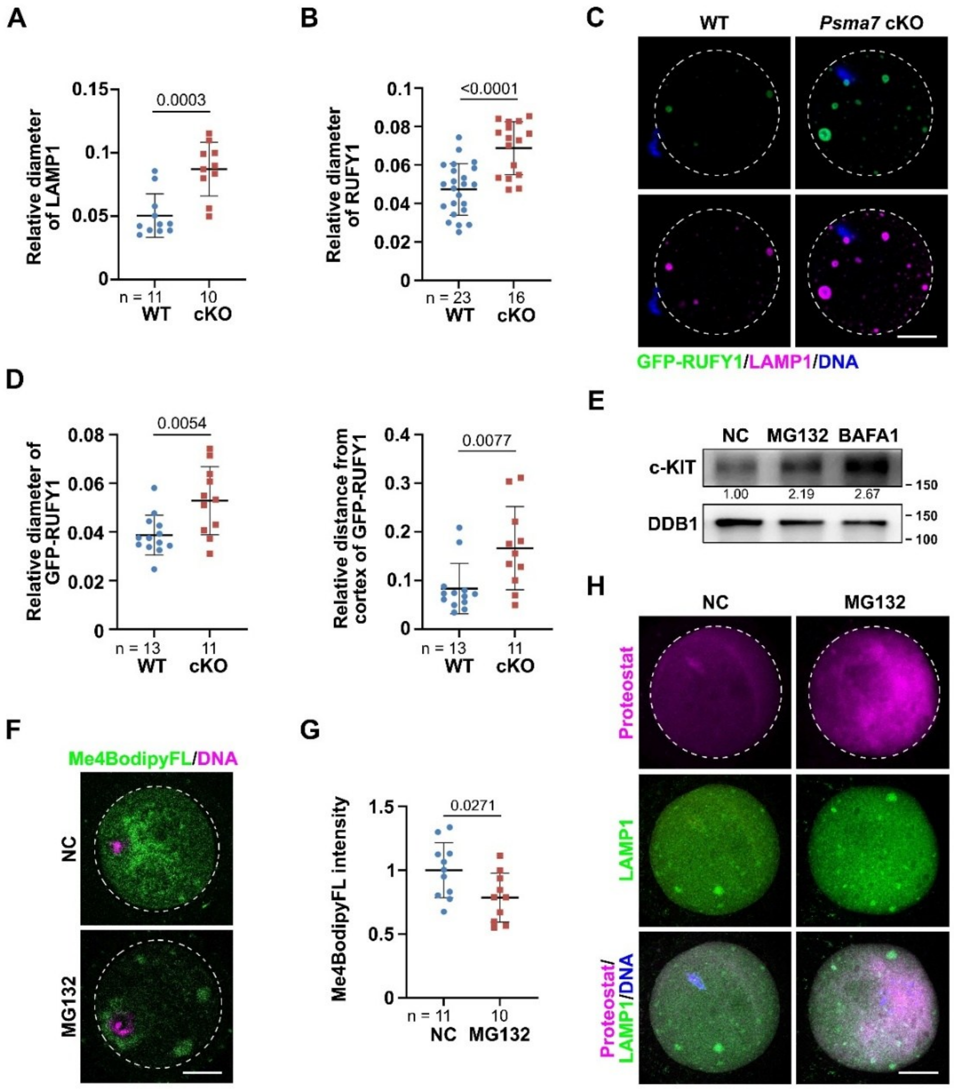

Appendix Figure S2. Inhibition of proteasome activity leads to impaired ELVAs function in oocytes.

(A-B) Quantification of the relative diameter of LAMP1- **(A)** and RUFY1- **(B)** positive compartments in WT and *Psma7* cKO eggs. Mean and SD are shown. p values: unpaired t test. **(C)** Confocal analysis of GFP-RUFY1 and endogenous LAMP1 signals in WT and *Psma7* cKO eggs. Scale bar, 20 μm. **(D)** Quantification of the diameter of RUFY1-positive compartments (left) and their relative distance from cortex (right) of panel **(C)**. Mean and SD are shown. p values: unpaired t test. **(E)** Western blot analysis of c-KIT in MI oocytes treated with vehicle, MG132 (20 μM) or BAFA1 (0.25 μM) since the GV stage. DDB1 serves as the loading control. **(F)** Confocal analysis of Me4BodipyFL signal in MI oocytes with or without MG132 treatment during maturation. NC, Negative Control. Scale bar, 20 μm. **(G)** Quantification of Me4BodipyFL signal MFI in panel **(F)**. Mean and SD are shown. p values: unpaired t test. **(H)** Confocal analysis of Proteostat and LAMP1 signals in MI oocytes with or without MG132 treatment during maturation. Scale bar, 20 μm. For all statistical analysis, n indicates the number of oocytes analyzed.

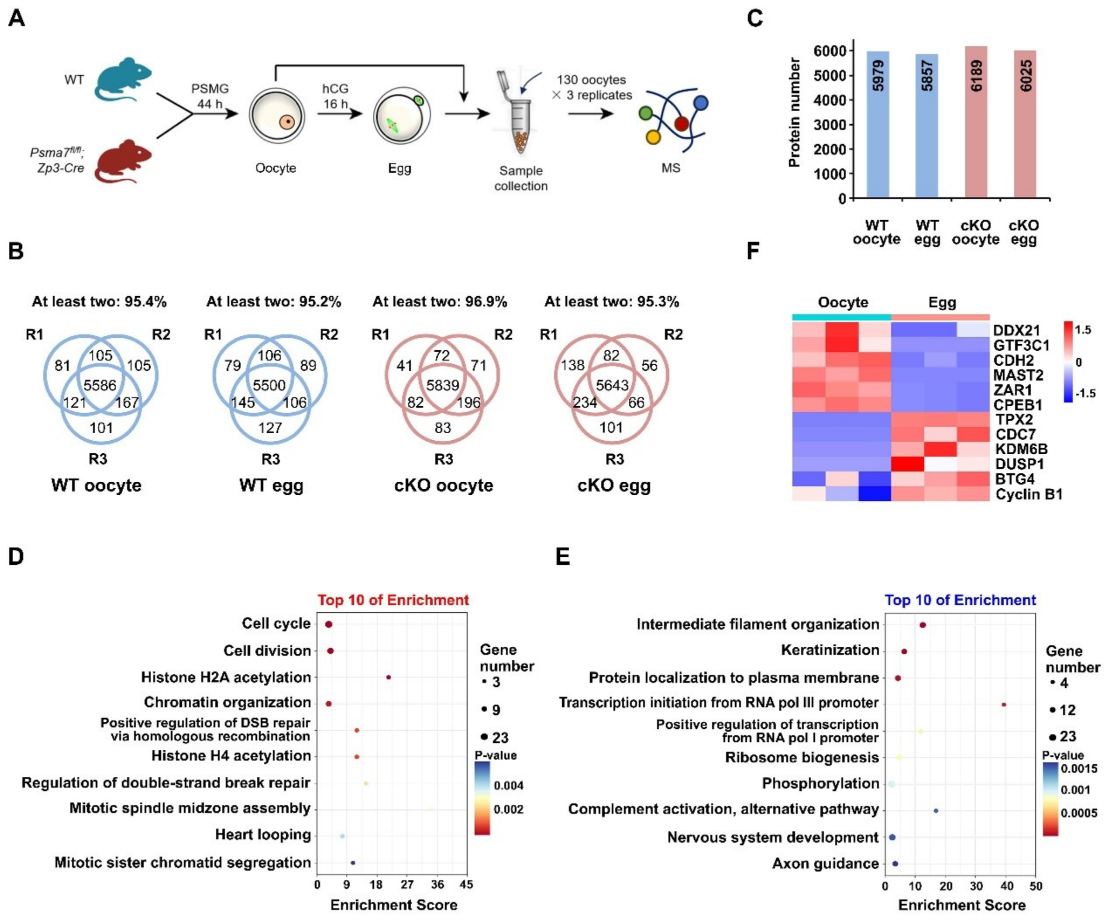

Appendix Figure S3. The micro-proteomics analysis.

(A) Workflow for MS sample collection. **(B)** Venn diagrams showing the overlap of identified proteins across three biological replicates in each group. **(C)** Number of consistently detected proteins in each group. **(D-E)** GO analysis of proteins significantly up-regulated **(D)** or down-regulated **(E)** during WT oocyte maturation. Results are analyzed using the DAVID web server. **(F)** Heat map showing stage-specific changes in indicated proteins levels.

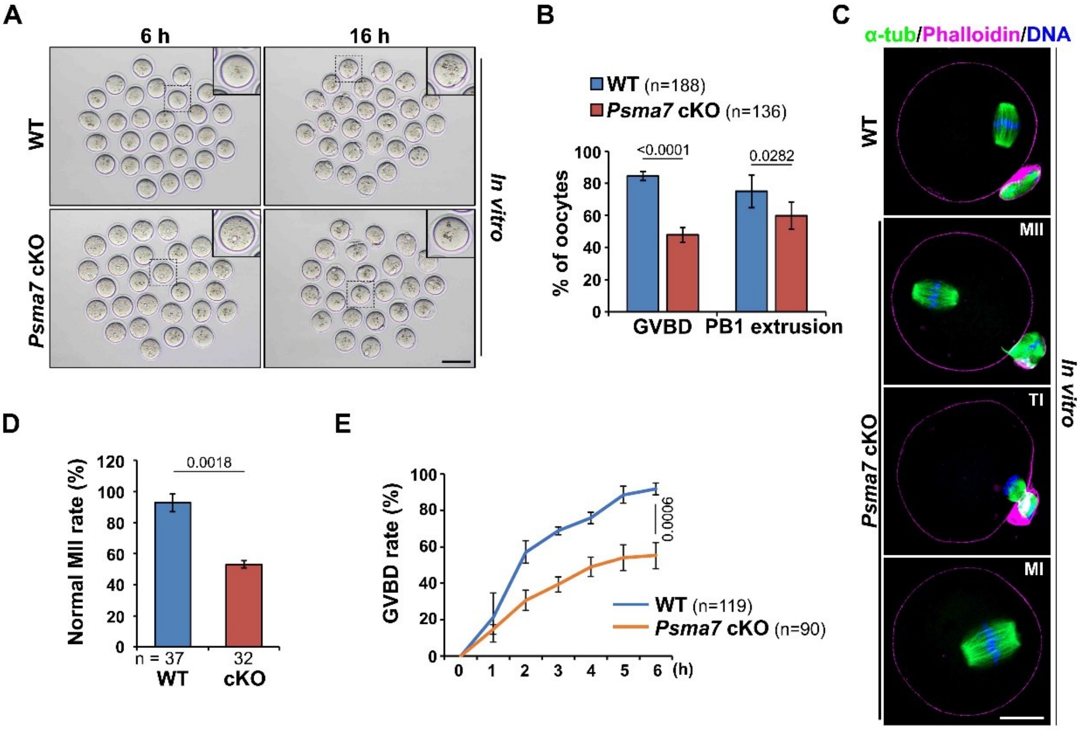

Appendix Figure S4. PSMA7 deletion impairs oocyte maturation *in vitro*.

(A) Representative images of WT and *Psma7* cKO oocytes following *in vitro* culture. Scale bar, 100 µm. **(B)** Rates of germinal vesicle breakdown (GVBD) and first polar body (PB1) extrusion in oocytes cultured *in vitro*. Fully grown oocytes were collected from PMSG-primed (44 h) mice of the indicated genotypes. Mean and SD are shown. p values: unpaired t test. **(C)** Confocal analysis of acetyl-α-tubulin (ac-tub) indicating spindle assembly and PB1 extrusion in WT and *Psma7* cKO oocytes at 16 h after *in vitro* culture. Developmental arrest stages are indicated in the upper right corner. Scale bar, 20 µm. **(D)** Proportion of *in vitro*-matured eggs with normal spindle assembly in WT and *Psma7* cKO groups. Mean and SD are shown. p values: unpaired t test. **(E)** Comparison of GVBD kinetics between WT and *Psma7* cKO oocytes cultured in *vitro*. Mean and SD are shown. p values: unpaired t test. For all statistical analysis, n indicates the number of oocytes analyzed.

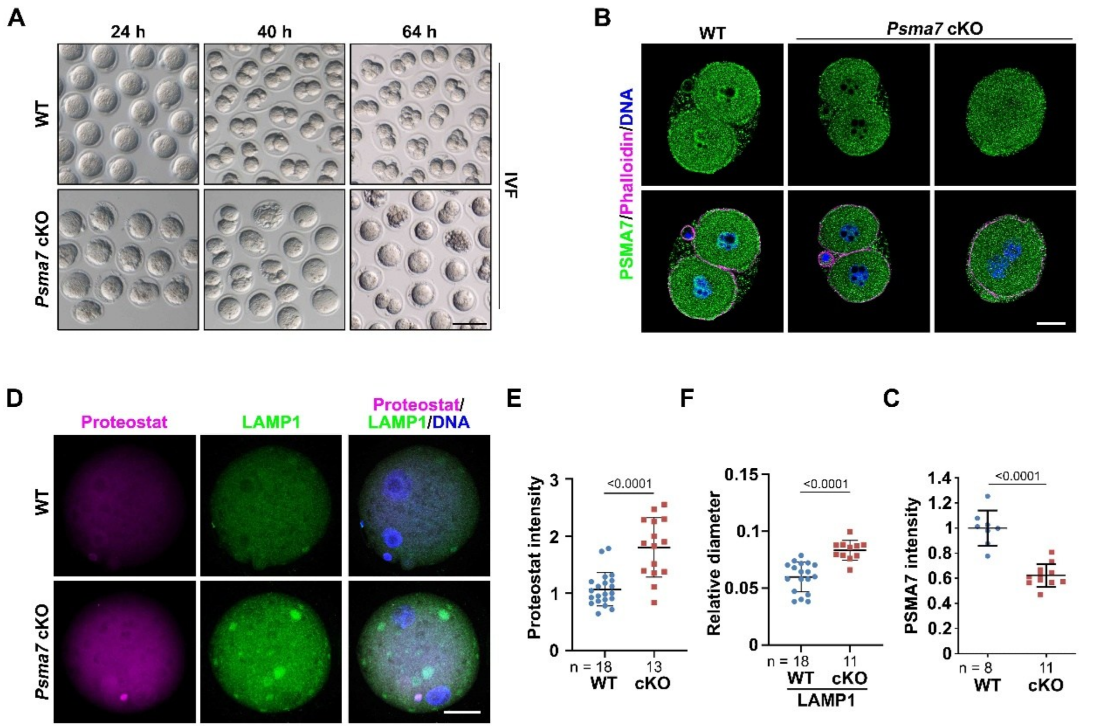

Appendix Figure S5. Maternal PSMA7 deletion impairs early embryonic development *in vitro*.

(A) Representative images of embryos at indicated time points post-hCG injection. Scale bar, 20 μm. **(B)** Immunofluorescent staining of PSMA7 in embryos derived from WT and *Psma7* cKO females at the presumptive 2-cell stage. Scale bar, 20 μm. **(C)** Quantification of the MFI of PSMA7 signal in panel **(B)**. Mean and SD are shown. p values: unpaired t test. **(D)** Confocal analysis of Proteostat and LAMP1 signals in zygotes from WT and *Psma7* cKO females. Scale bar, 20 μm. **(E-F)** Quantification of the MFI of Proteostat signal **(E)** and the relative diameter of LAMP1-positive compartment **(F)** in panel **(D)**. Mean and SD are shown. p values: unpaired t test.
