# Supplementary material for: 20S proteasome-regulated proteostasis in ELVAs is critical for oocyte-to-embryo transition and female fertility
Source: EMBO J. 2026 May 21;45(14):4887–909. doi: 10.1038/s44318-026-00813-0 (PMC13373198; doi:10.1038/s44318-026-00813-0)
Supplement: Supplementary file 2 — Table EV1 [file 44318_2026_813_MOESM2_ESM.docx]

**Table EV1. Antibody information**

| **Antibody name** | **Manufacture**  **(catalogue number)** | **Applications**  **(working dilution)** |
| --- | --- | --- |
| Anti-PSMA7 | Abcam (ab133502) | IF (1:200); IHC (1:200); WB (1:500) |
| Anti-PSMC2 | Proteintech (14905-1-AP) | IF (1:200); WB (1:500) |
| Anti-20S Proteasome α1, 2, 3, 5, 6, & 7-Subunits (α-sub) | Merck (ST1049) | IF (1:200); WB (1:500) |
| Anti-LAMP1 | Cell Signaling (99437)  Abcam (ab24170) | IF (1:200)  WB (1:1000) |
| Anti-RUFY1 | Proteintech (13498-1-AP) | IF (1:200); WB (1:1000) |
| Anti-Ubiquitin conjugates (FK2) | Ubiquigent (68-0121-500) | IF (1:200) |
| Anti-Acetyl-α-tubulin | Cell Signaling (5335) | IF (1:200) |
| Anti-FITC-α-tubulin | Sigma (F2168) | IF (1:200) |
| Anti-TOP2B | Abcam (ab109524) | IF (1:200) |
| Anti-CREST | Fitzgerald Industries International (70R-21494) | IF (1:100) |
| Anti-PolIISer2P | Abcam (ab5095) | IF (1:40000) |
| Anti-Ubiquitin | Cell Signaling (3936) | IF (1:200) |
| Anti-MVH | Abcam (ab13840) | IHC (1:200) |
| Anti-cyclin B1 | Cell Signaling (4138) | WB (1:1000) |
| Anti-Fetuin B | Proteintech (18052-1-AP) | IF (1:200) |
| Anti-pERK1/2 | Cell Signaling (9101) | WB (1:100) |
| Anti-DDB1 | Epitomics (3821-1) | WB (1:10000) |
| Anti-GAPDH | Proteintech (60004-1-Ig) | WB (1:5000) |
| Goat Anti-rabbit IgG H&L (HRP) | Abcam (Ab6721) | WB (1:5000); IHC (1:200) |
| Rabbit Anti-mouse IgG H&L (HRP) | Abcam (Ab6728) | WB (1:5000); IHC (1:200) |
| Goat anti-Mouse IgG (H+L), Alexa Fluor Plus 488 | Thermo Scientific (A11001) | IF (1:200) |
| Goat anti-Rabbit IgG (H+L), Alexa Fluor Plus 568 | Thermo Scientific (A11011) | IF (1:200) |
