# Supplementary material for: 20S proteasome-regulated proteostasis in ELVAs is critical for oocyte-to-embryo transition and female fertility
Source: EMBO J. 2026 May 21;45(14):4887–909. doi: 10.1038/s44318-026-00813-0 (PMC13373198; doi:10.1038/s44318-026-00813-0)
Supplement: Supplementary file 3 — Table EV2 [file 44318_2026_813_MOESM3_ESM.docx]

**Table EV2. Primer sequences**

| **Primer name** | **Target** | **Application** | **Sequences (5′-3′)** |
| --- | --- | --- | --- |
| *Psma7* GP-1 | *Psma7* | Genotyping of *Psma7* allele:  WT-326 bp  Flox-431 bp  KO-578 bp | 5′- CTGCTGCATCCAGCTAGCTT -3′ |
| *Psma7* GP-2 |  |  | 5′- TGGTTCCTGAGTCTTCATTGG -3′ |
| *Psma7* GP-3 |  |  | 5′- TATGGGAGATGGAGGTTTTCC -3′ |
| *Zp3-Cre*-F | *Zp3-Cre* | Genotyping of *Zp3-Cre* | 5′- AAGAACCTGATGGACATGTTCAG -3′ |
| *Zp3-Cre*-R |  |  | 5′- CTGATCCTGGCAATTTCGG -3′ |
| *Zscan4d*-F | *Zscan4d* | RT-PCR | 5′- CAATGCAAGGACAAGAAGCTCTCTT -3′ |
| *Zscan4d*-R |  |  | 5′- CTGGCATCAAGAGGGAATTGAAA -3′ |
| *Zscan5b*-F | *Zscan5b* | RT-PCR | 5′- ATGGGCAATACAGAAGATGGGC -3′ |
| *Zscan5b*-R |  |  | 5′- GGTCAAACCGGGACTTGTAAA -3′ |
| *Dppa4*-F | *Dppa4* | RT-PCR | 5′- AGTCAACCTAGCACGGCTC -3′ |
| *Dppa4*-R |  |  | 5′- TCCTGGCGTCTCAGTGTCT -3′ |
| *Gucala*-F | *Gucala* | RT-PCR | 5′- ATCTAGGGCATCCGTCTCTT -3′ |
| *Gucala* -R |  |  | 5′- GATGGGCACTCCGTCATAAA -3′ |
| *MuERV-L*-F | *MuERV-L* | RT-PCR | 5′- ATCTCCTGGCACCTGGTATG -3′ |
| *MuERV-L*-R |  |  | 5′- AGAAGAAGGCATTTGCCAGA -3′ |
| *Ctsl*-F | *Ctsl* | RT-PCR | 5′- CAAGCCATCCGTCTCTCCAGTTC -3′ |
| *Ctsl*-R |  |  | 5′- TGGCAAGTCCACAGTGGTTGTCC -3′ |
