# Supplementary material for: 20S proteasome-regulated proteostasis in ELVAs is critical for oocyte-to-embryo transition and female fertility
Source: EMBO J. 2026 May 21;45(14):4887–909. doi: 10.1038/s44318-026-00813-0 (PMC13373198; doi:10.1038/s44318-026-00813-0)
Supplement: Supplementary file 5 — Source data Fig. 1 [file 44318_2026_813_MOESM5_ESM.zip › Figure 1/1G/1G.pptx]

## Slide 1
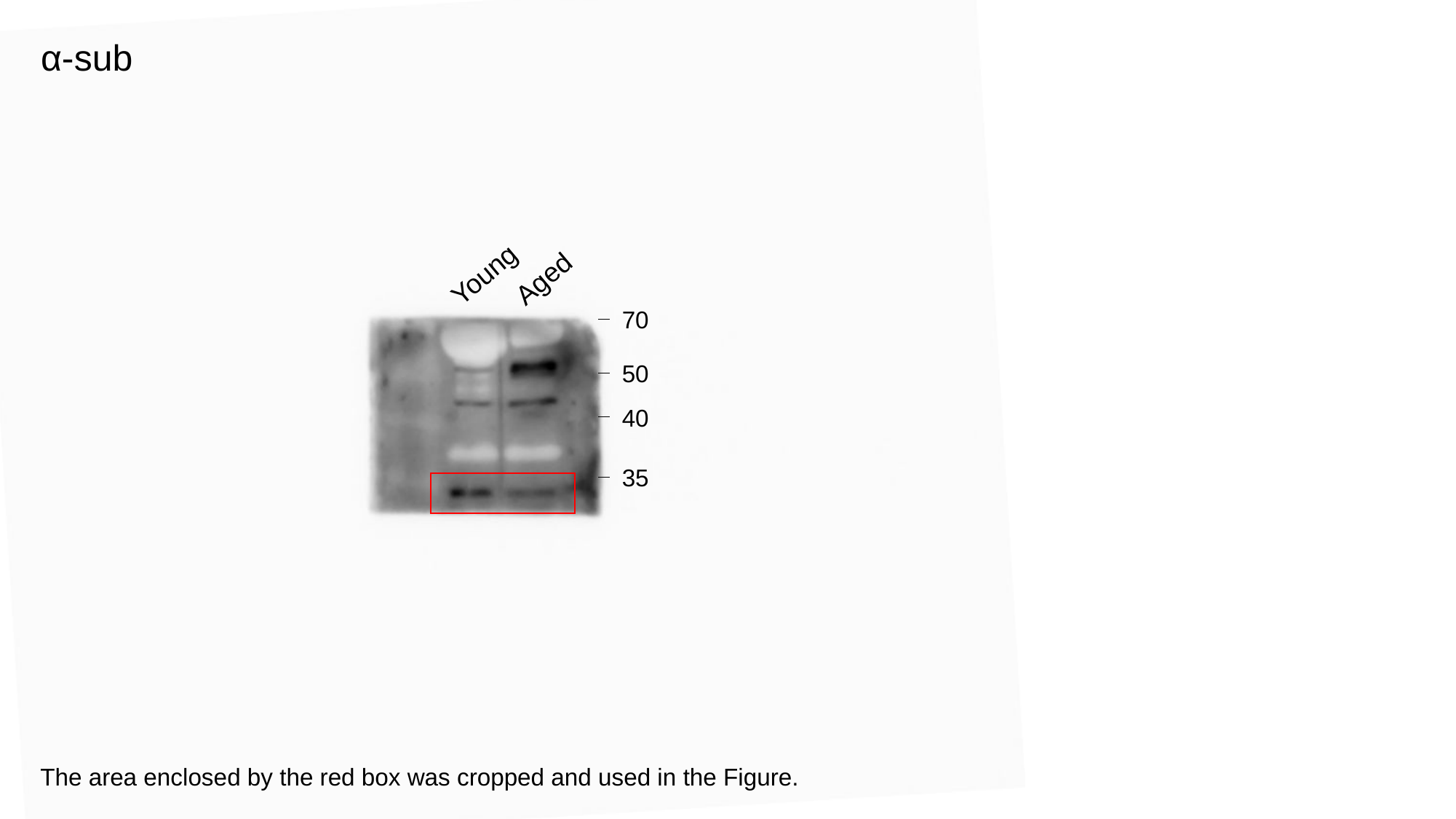

α-sub
Young
Aged
70
50
40
35
The area enclosed by the red box was cropped and used in the Figure.

## Slide 2
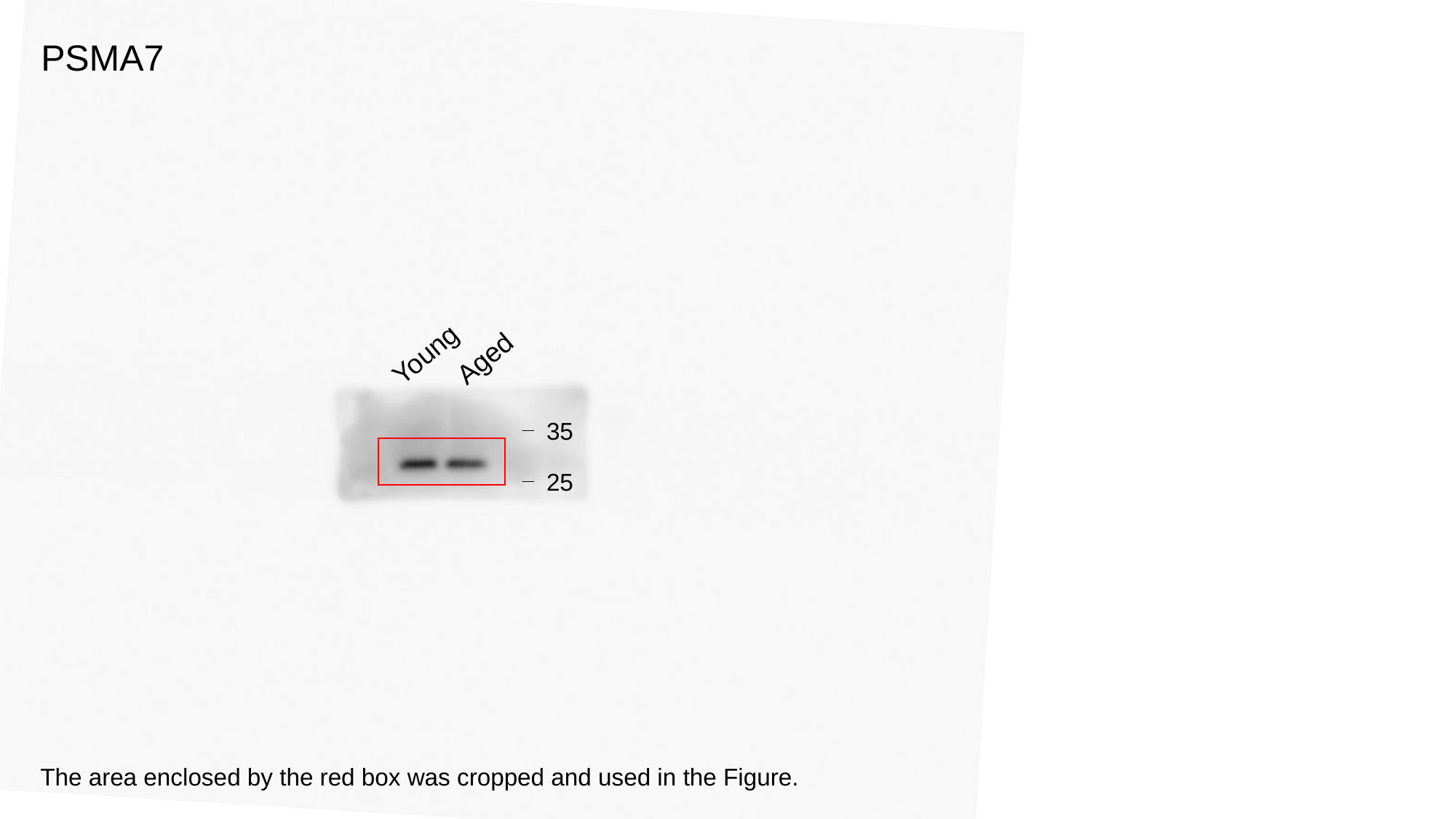

PSMA7
Young
Aged
35
25
The area enclosed by the red box was cropped and used in the Figure.

## Slide 3
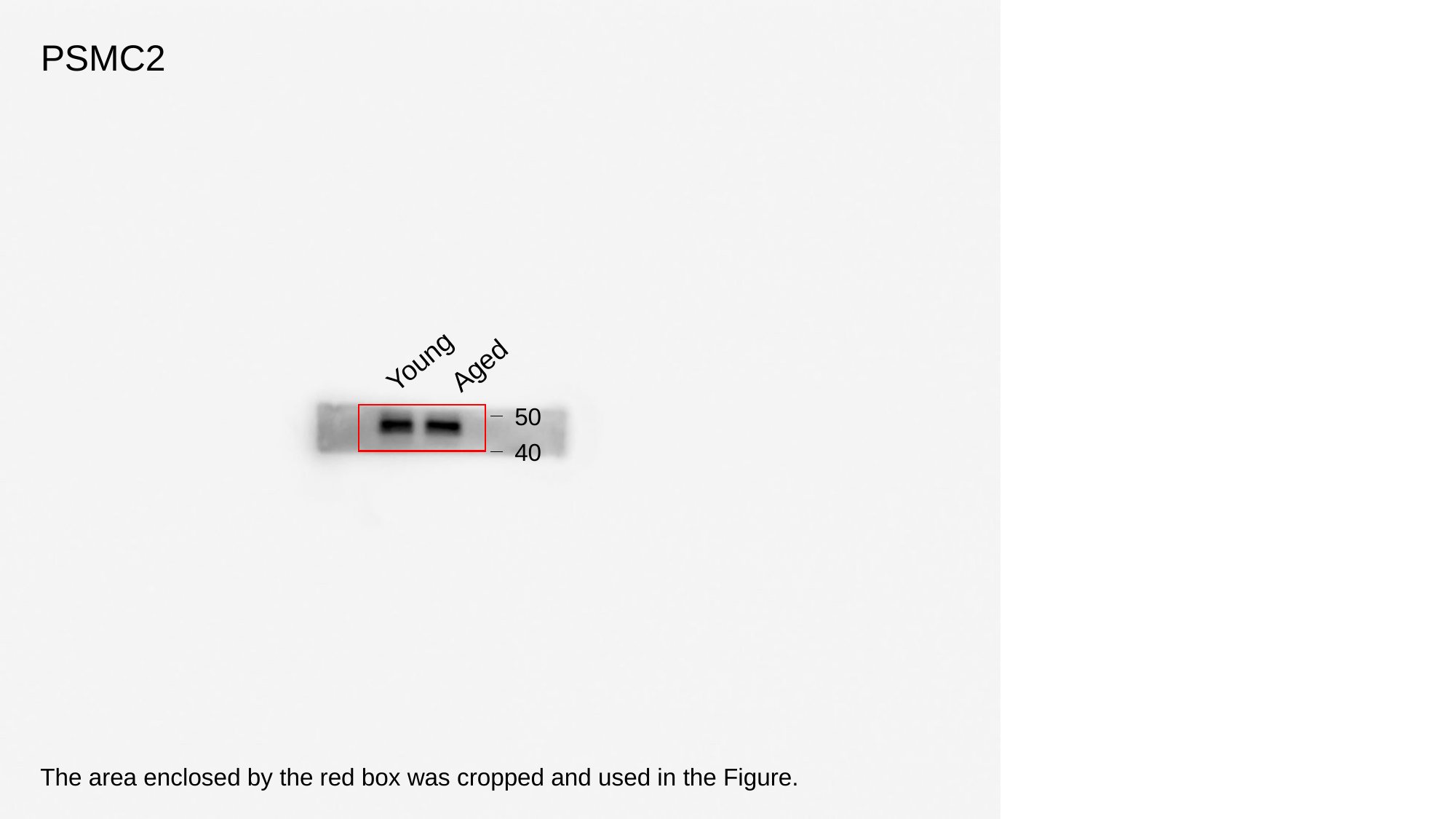

PSMC2
Young
Aged
50
40
The area enclosed by the red box was cropped and used in the Figure.

## Slide 4
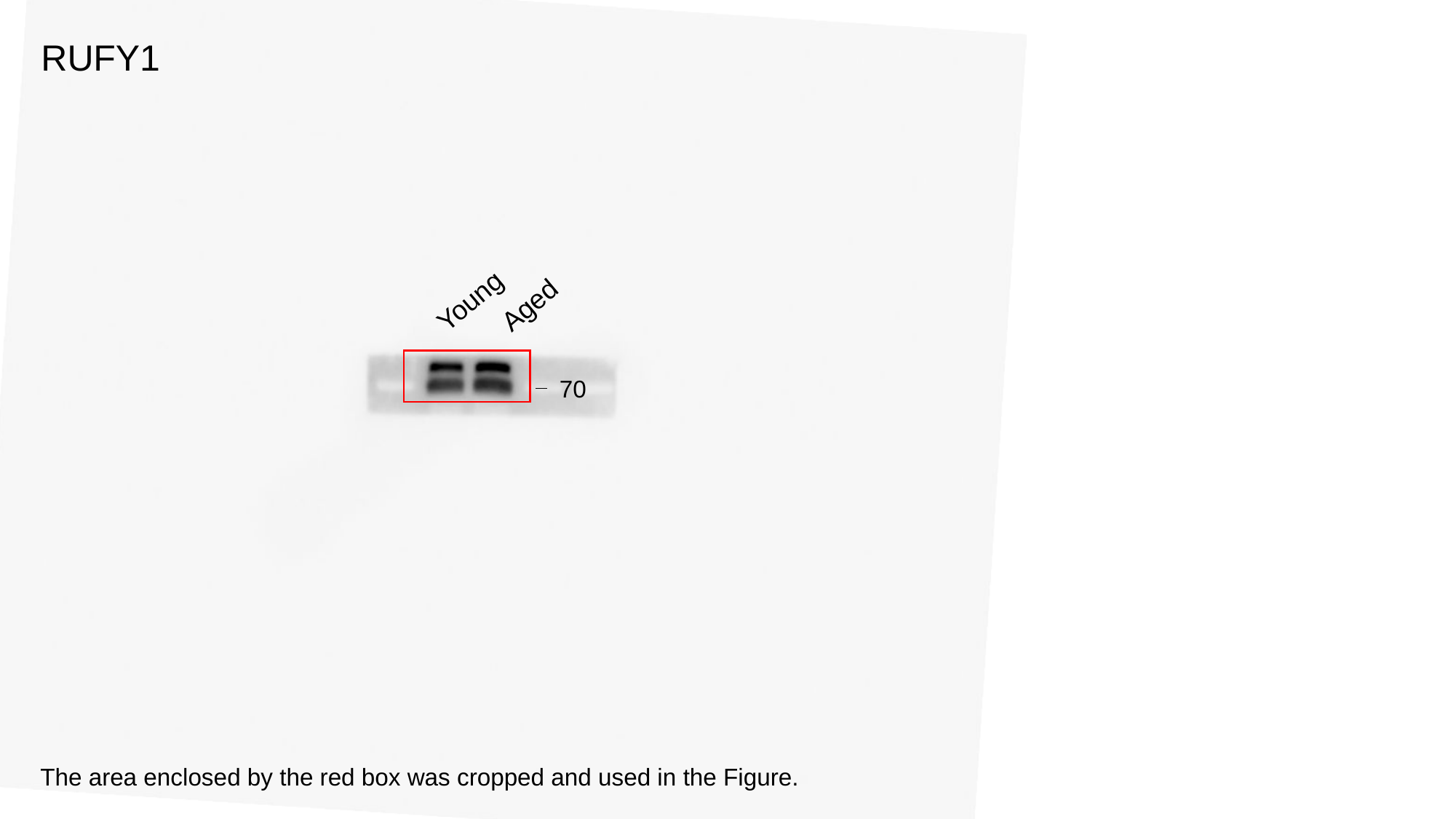

RUFY1
Young
Aged
70
The area enclosed by the red box was cropped and used in the Figure.

## Slide 5
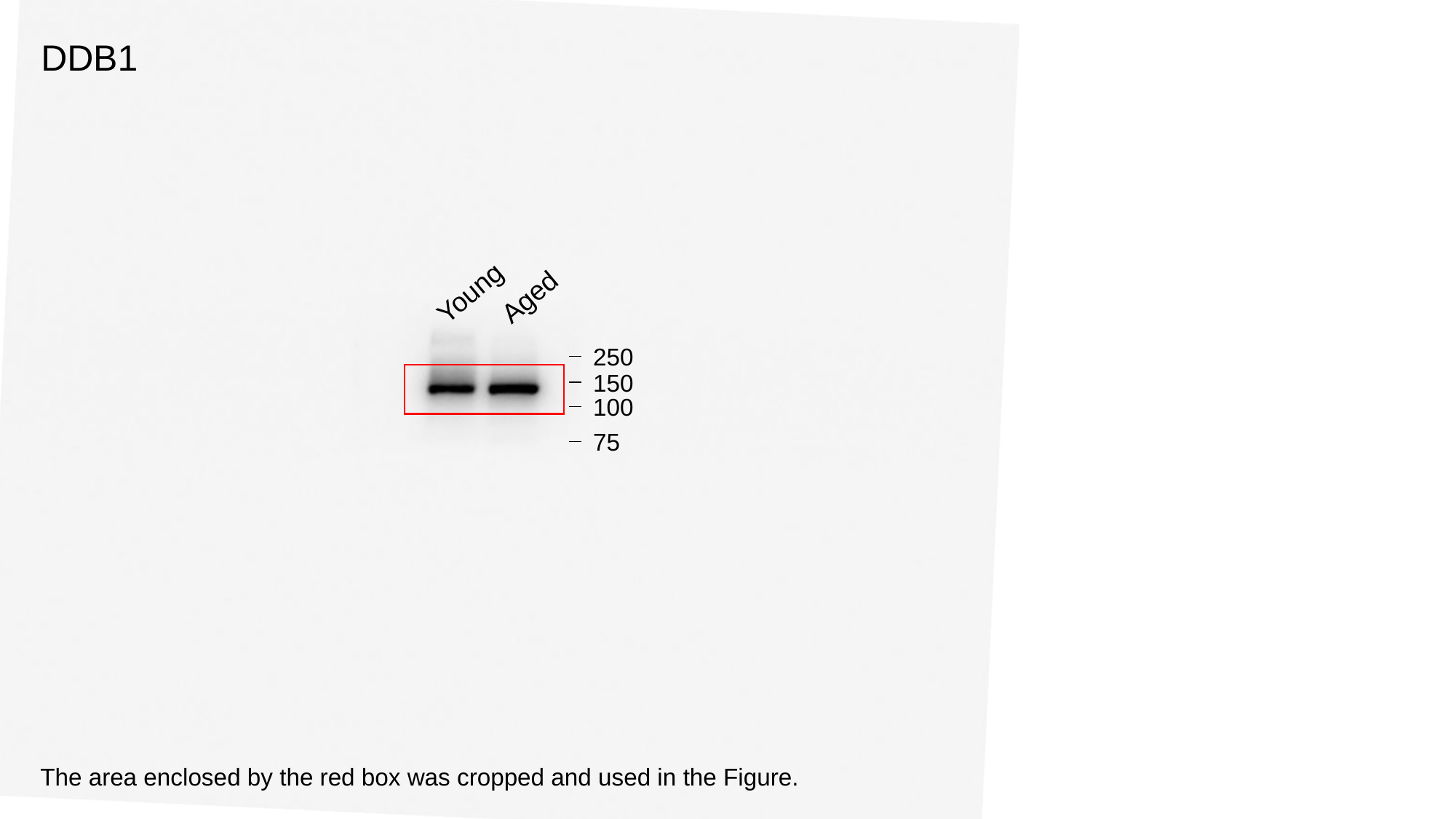

DDB1
Young
Aged
250
150
100
75
The area enclosed by the red box was cropped and used in the Figure.
