# Supplementary figures and images for: 20S proteasome-regulated proteostasis in ELVAs is critical for oocyte-to-embryo transition and female fertility
Source: EMBO J. 2026 May 21;45(14):4887–909. doi: 10.1038/s44318-026-00813-0 (PMC13373198; doi:10.1038/s44318-026-00813-0)

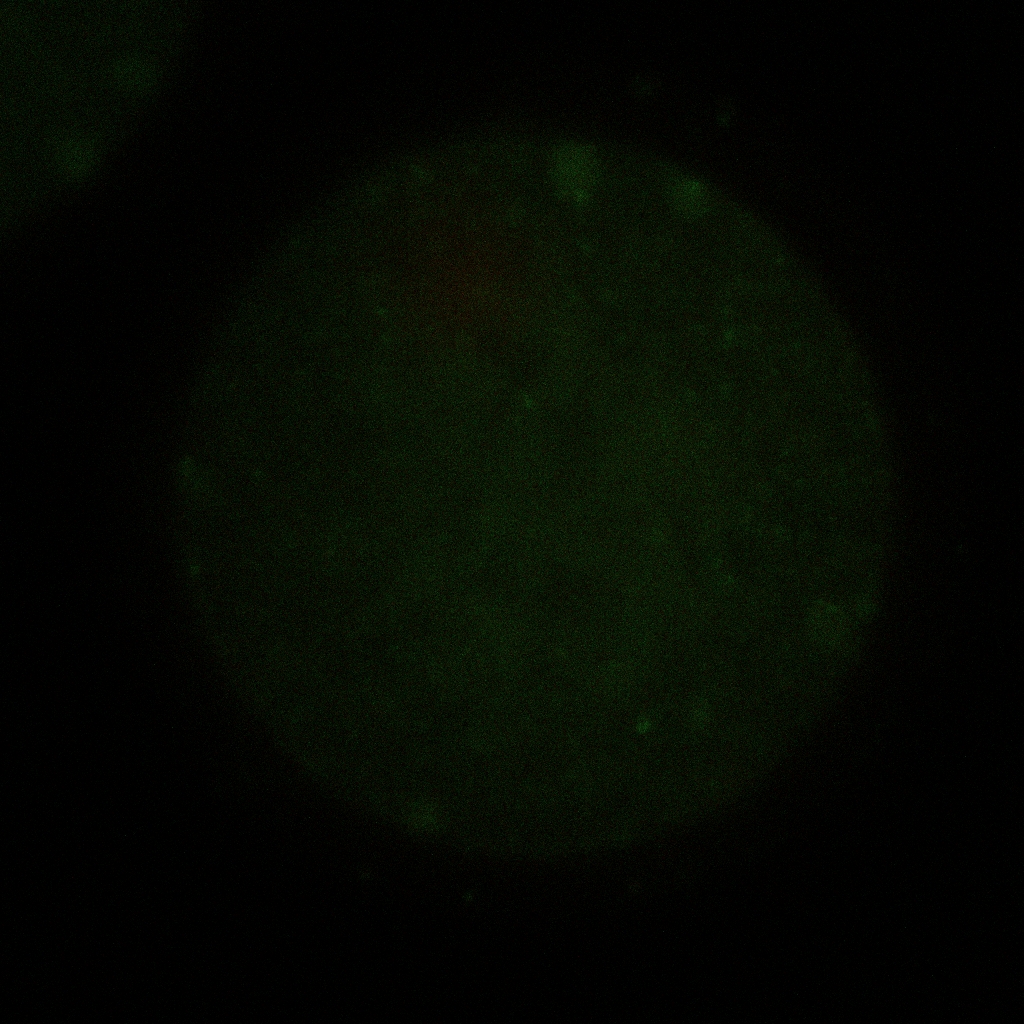

Supplement: Supplementary file 5 — Source data Fig. 1 [file 44318_2026_813_MOESM5_ESM.zip › Figure 1/1A/aged egg.jpg]

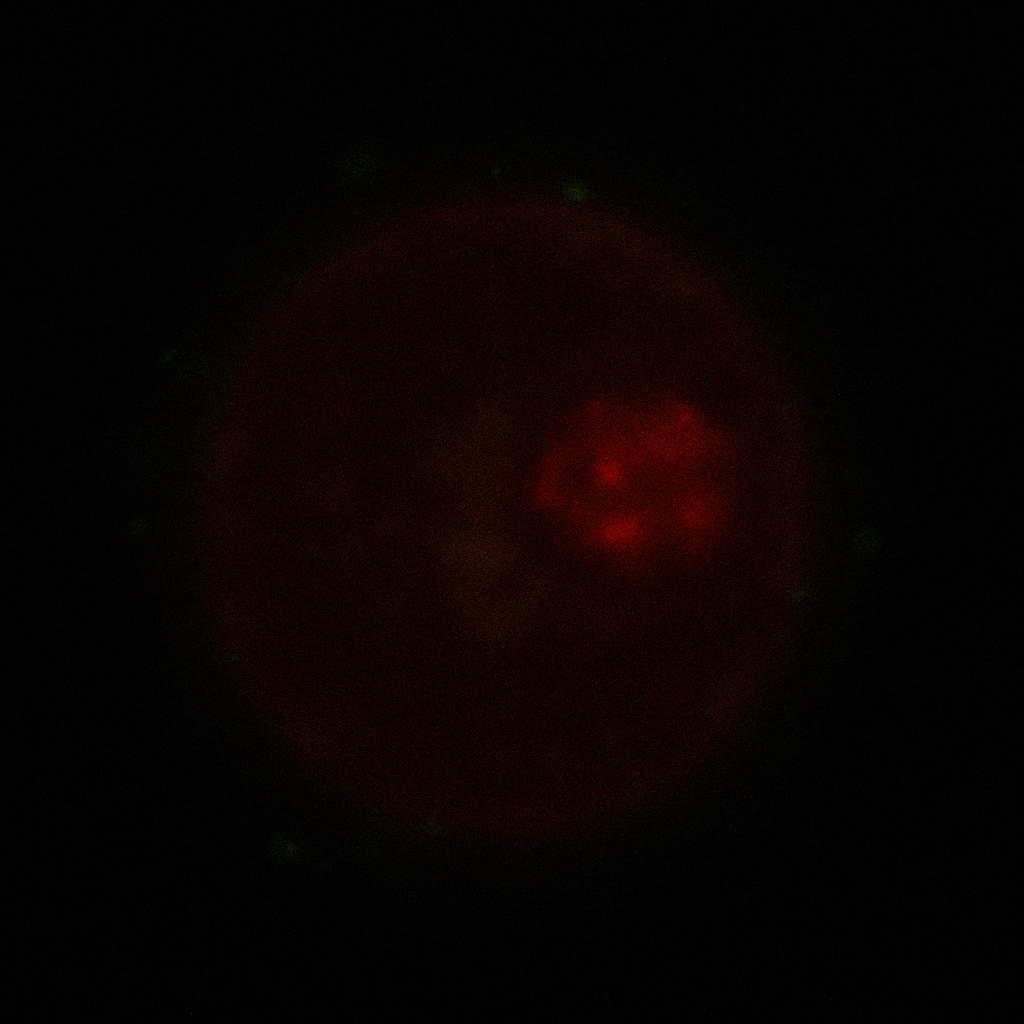

Supplement: Supplementary file 5 — Source data Fig. 1 [file 44318_2026_813_MOESM5_ESM.zip › Figure 1/1A/aged oocyte.jpg]

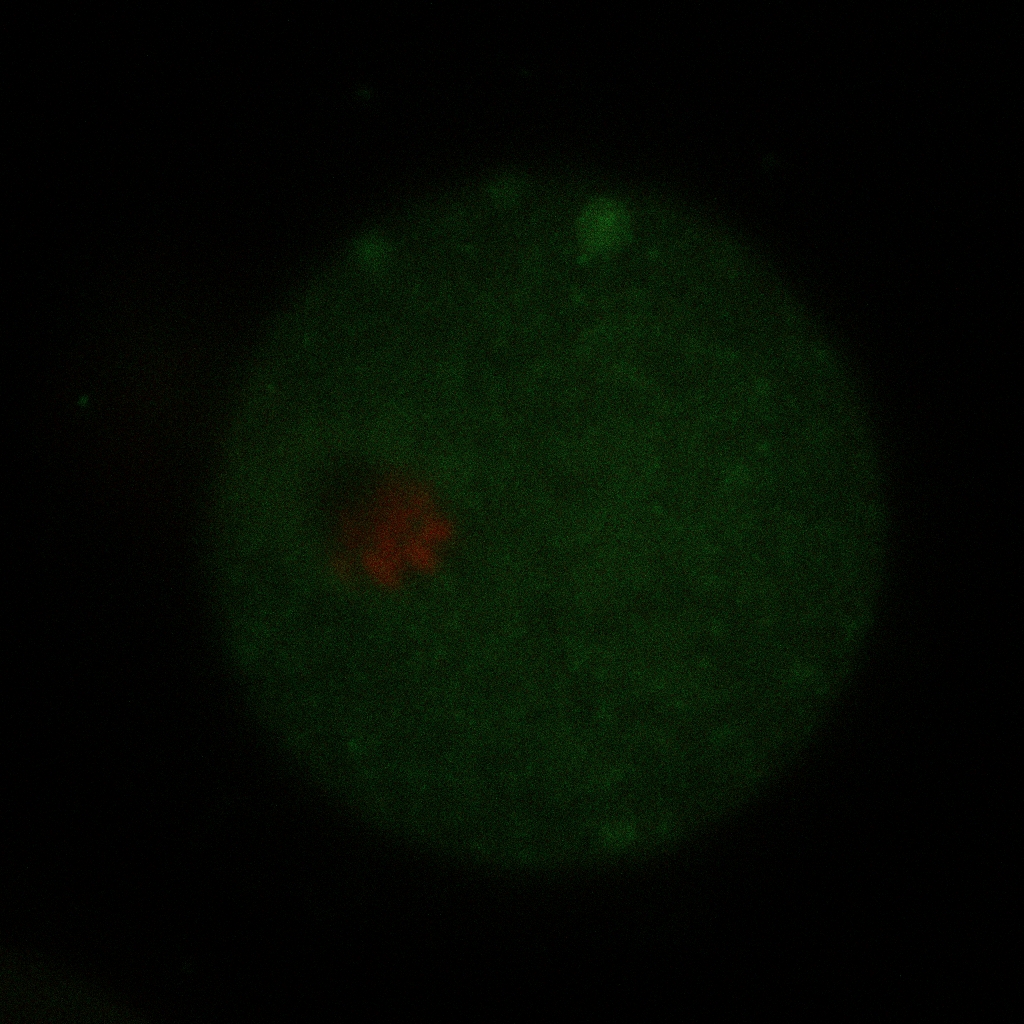

Supplement: Supplementary file 5 — Source data Fig. 1 [file 44318_2026_813_MOESM5_ESM.zip › Figure 1/1A/young egg.jpg]

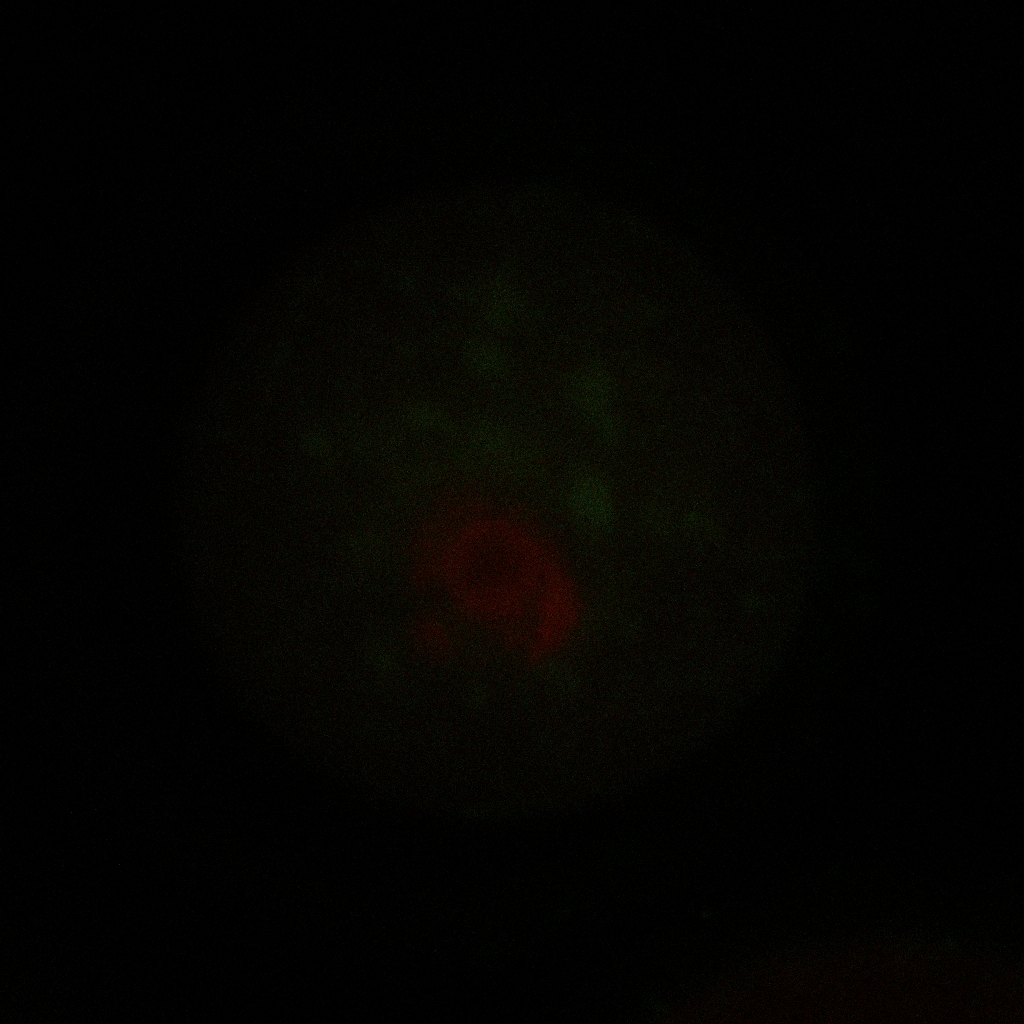

Supplement: Supplementary file 5 — Source data Fig. 1 [file 44318_2026_813_MOESM5_ESM.zip › Figure 1/1A/young oocyte.jpg]

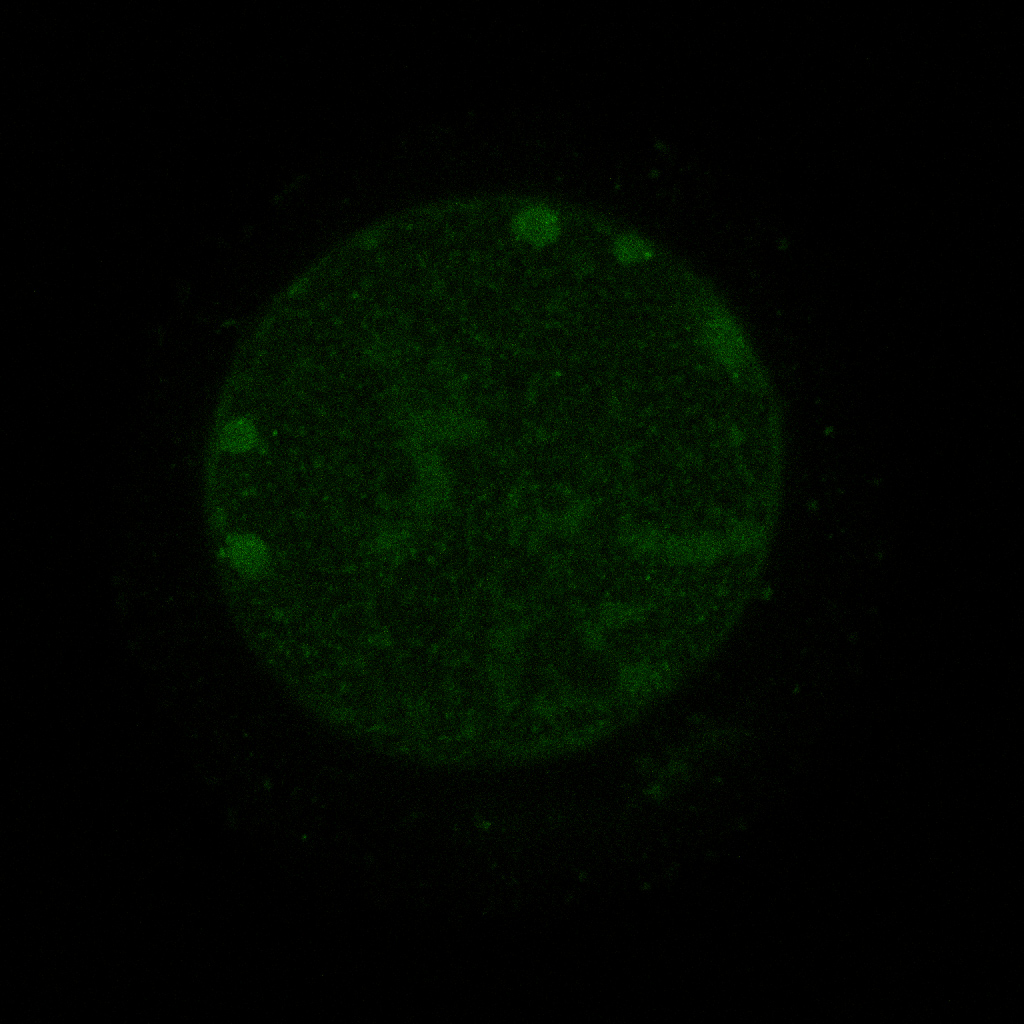

Supplement: Supplementary file 5 — Source data Fig. 1 [file 44318_2026_813_MOESM5_ESM.zip › Figure 1/1C/lysosensor-aged.jpg]

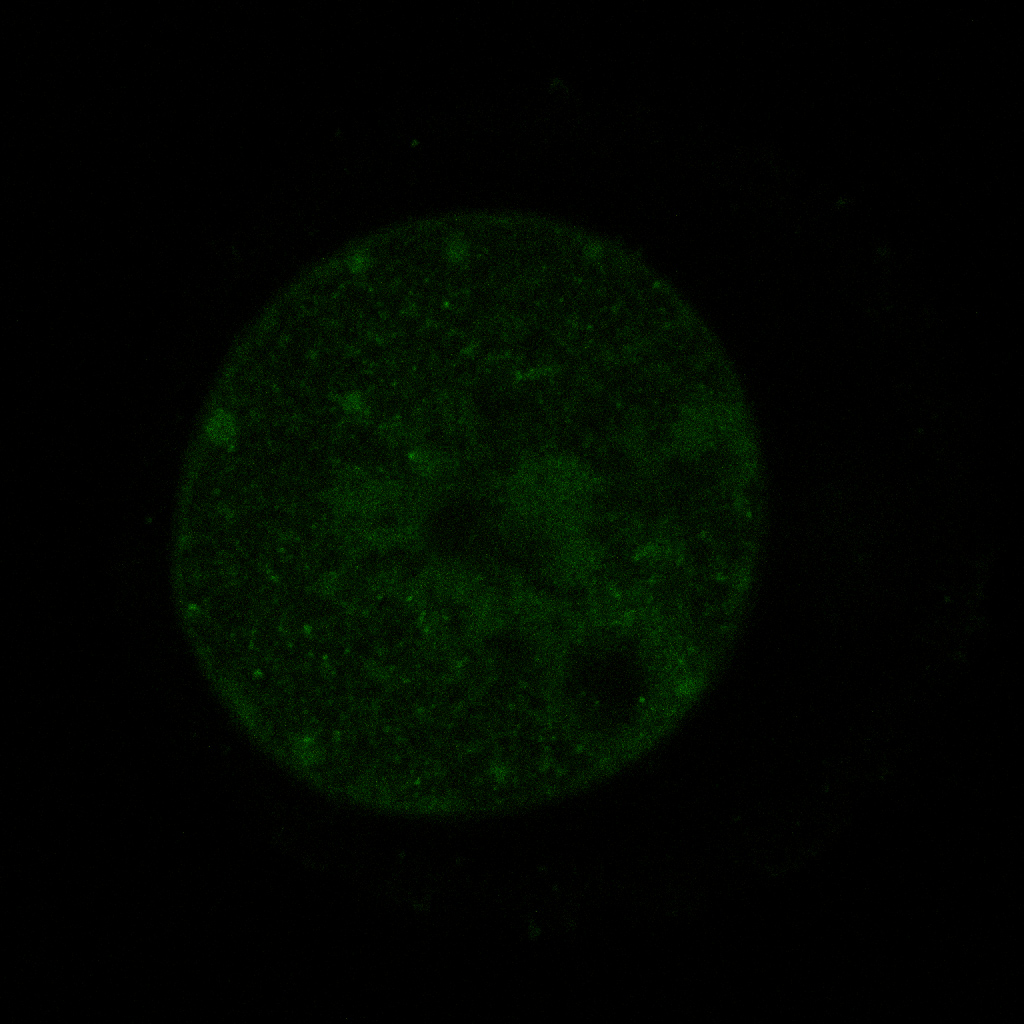

Supplement: Supplementary file 5 — Source data Fig. 1 [file 44318_2026_813_MOESM5_ESM.zip › Figure 1/1C/lysosensor-young.jpg]

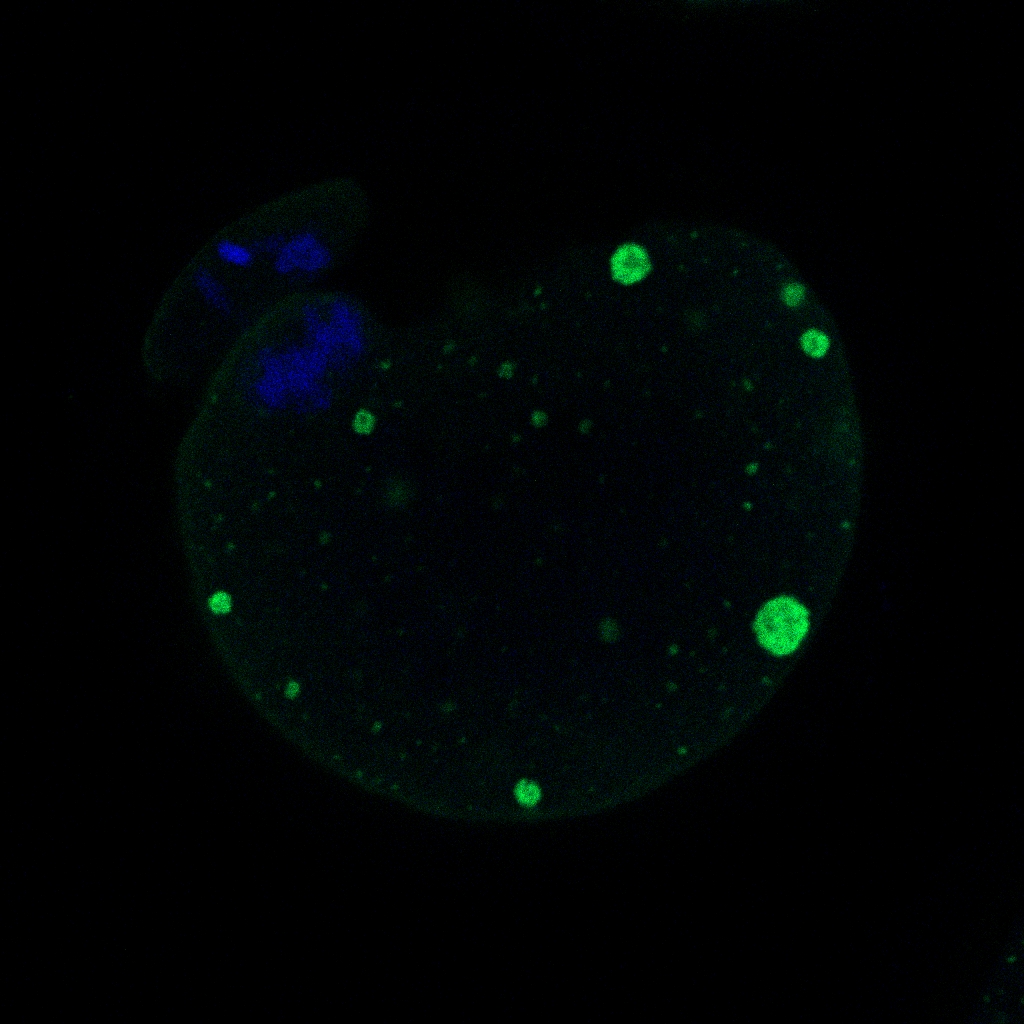

Supplement: Supplementary file 5 — Source data Fig. 1 [file 44318_2026_813_MOESM5_ESM.zip › Figure 1/1C/RUFY1-aged.jpg]

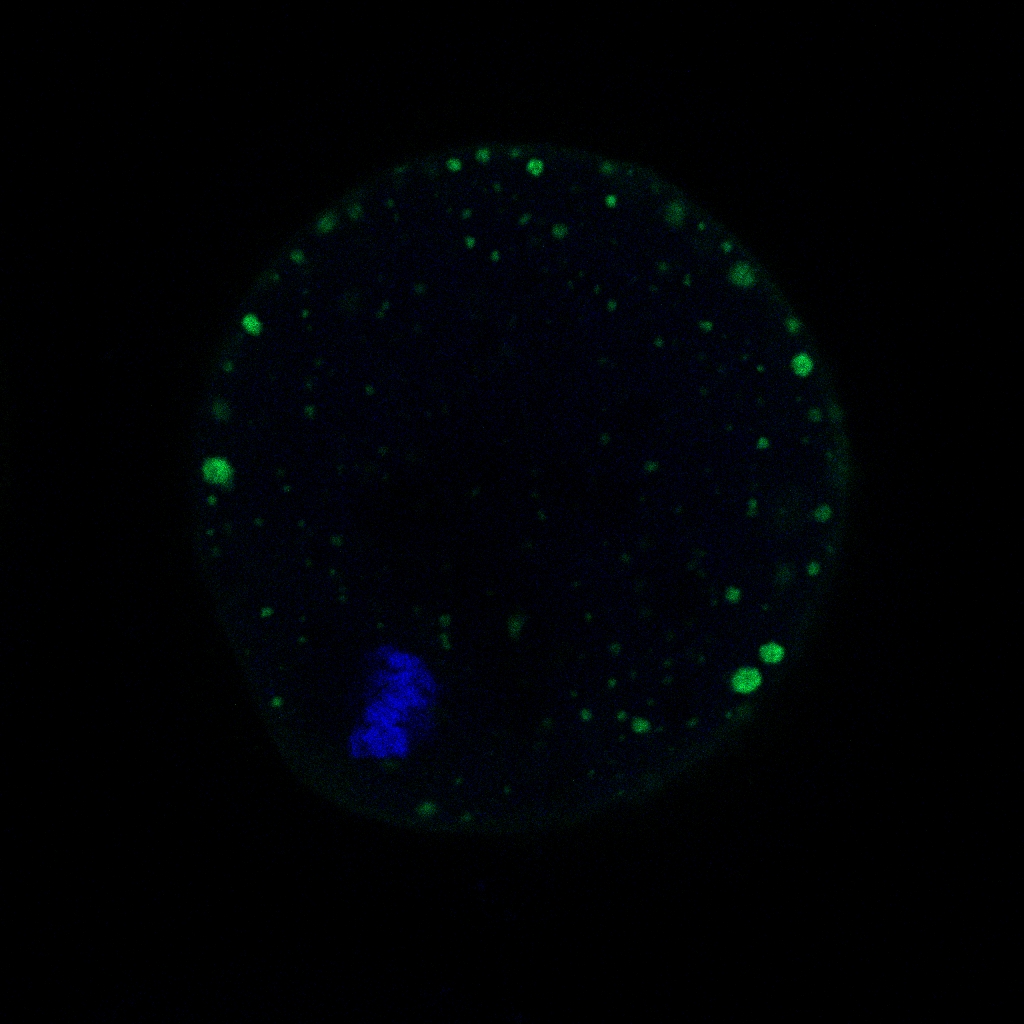

Supplement: Supplementary file 5 — Source data Fig. 1 [file 44318_2026_813_MOESM5_ESM.zip › Figure 1/1C/RUFY1-young.jpg]

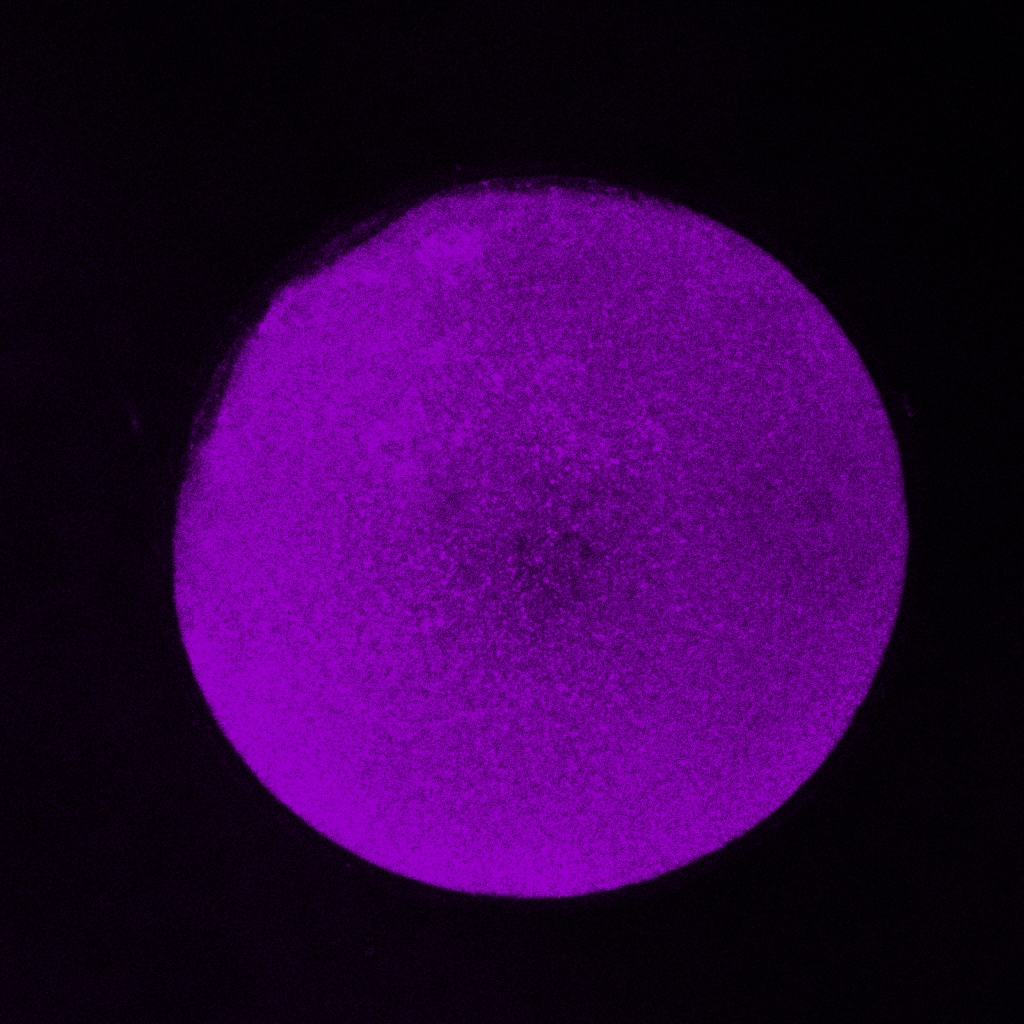

Supplement: Supplementary file 5 — Source data Fig. 1 [file 44318_2026_813_MOESM5_ESM.zip › Figure 1/1H/hPSMA7 28.jpg]

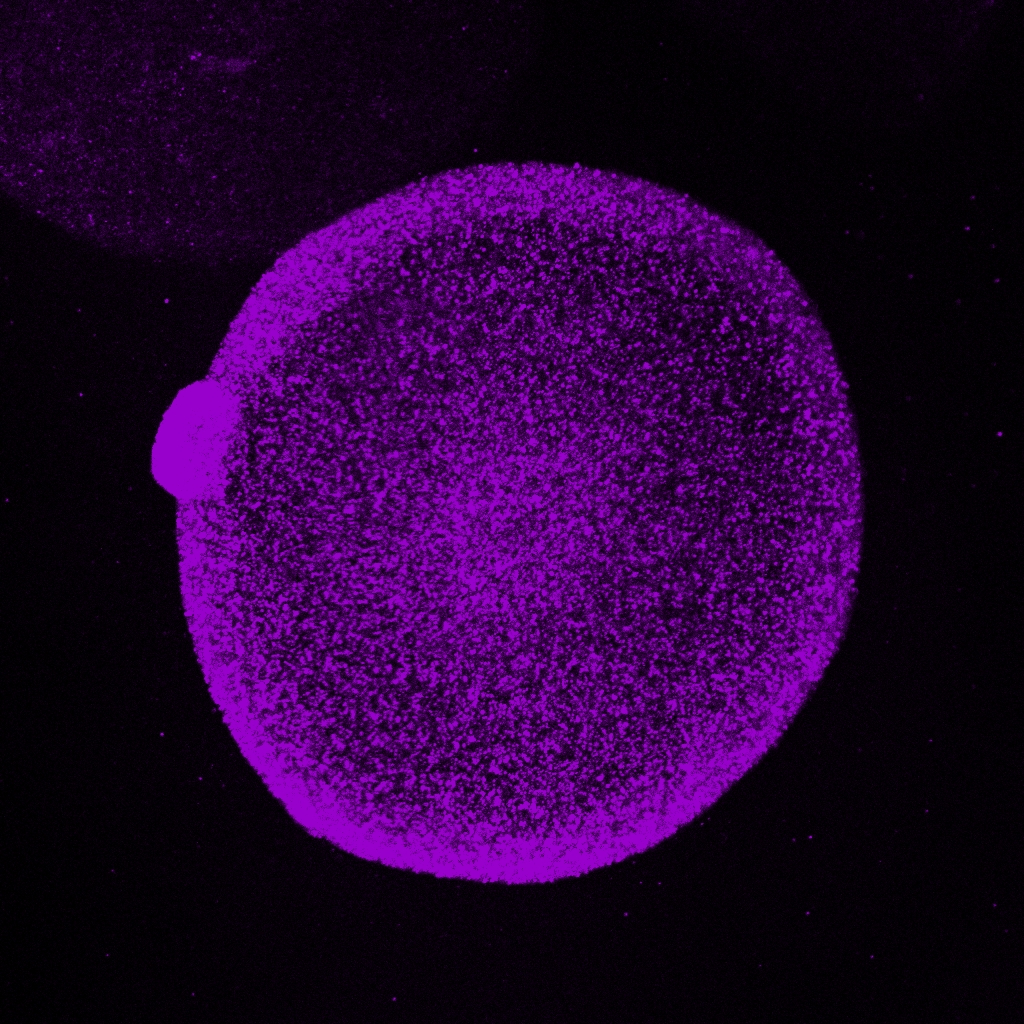

Supplement: Supplementary file 5 — Source data Fig. 1 [file 44318_2026_813_MOESM5_ESM.zip › Figure 1/1H/hPSMA7 38.jpg]

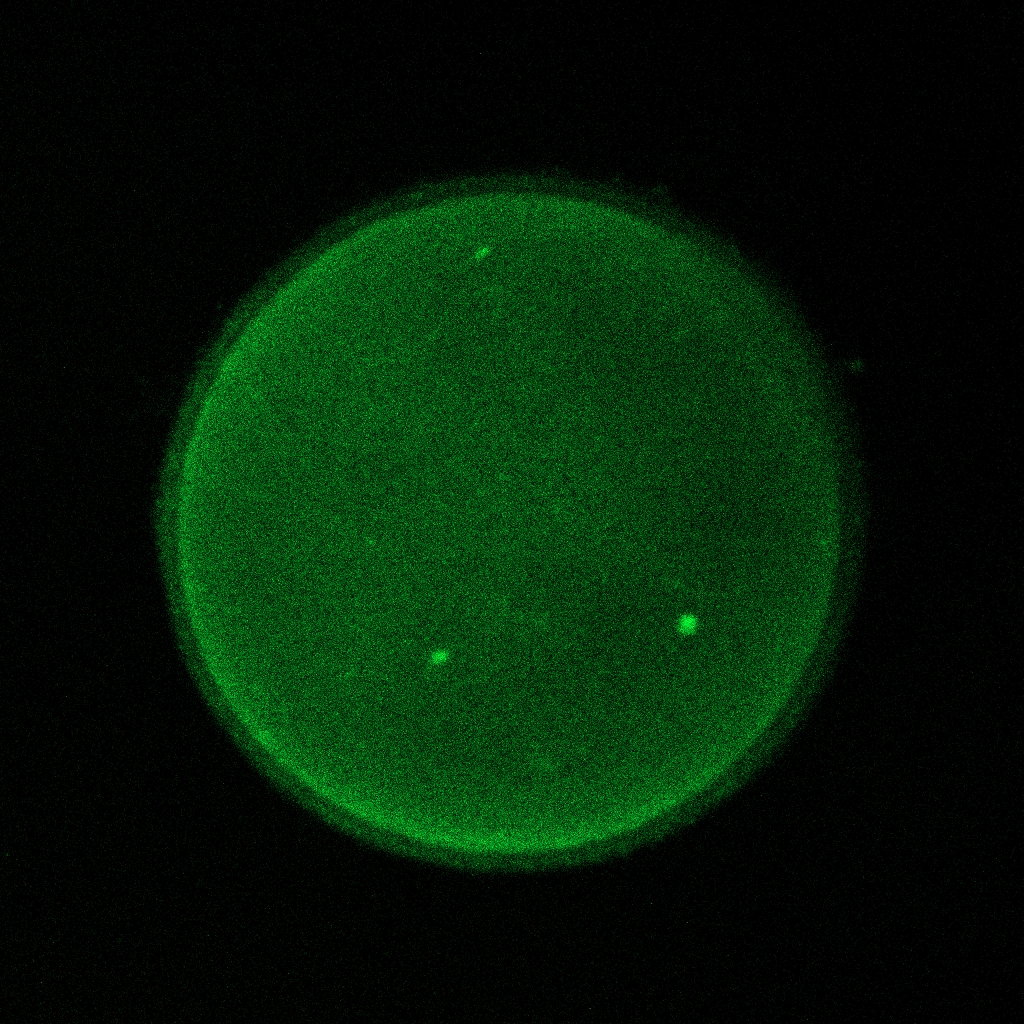

Supplement: Supplementary file 5 — Source data Fig. 1 [file 44318_2026_813_MOESM5_ESM.zip › Figure 1/1H/hα-sub 28.jpg]

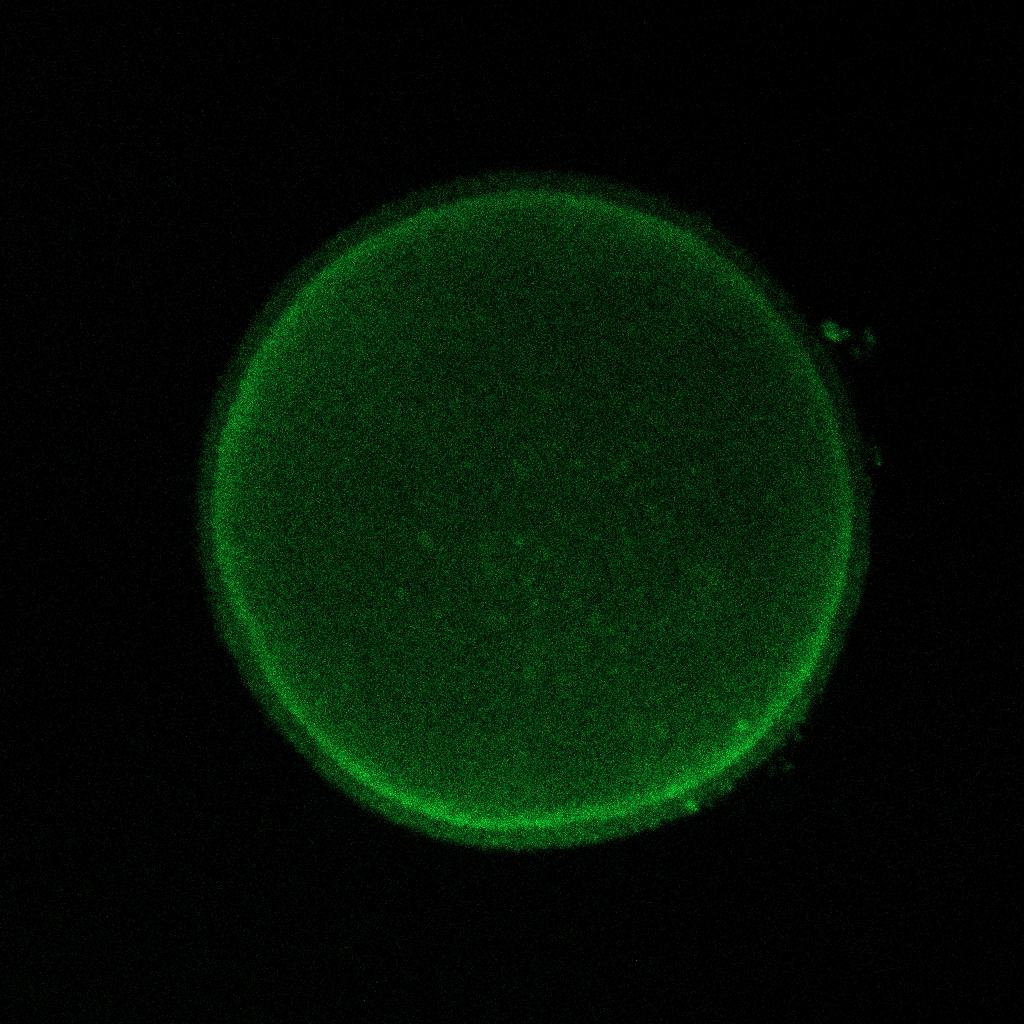

Supplement: Supplementary file 5 — Source data Fig. 1 [file 44318_2026_813_MOESM5_ESM.zip › Figure 1/1H/hα-sub 38.jpg]

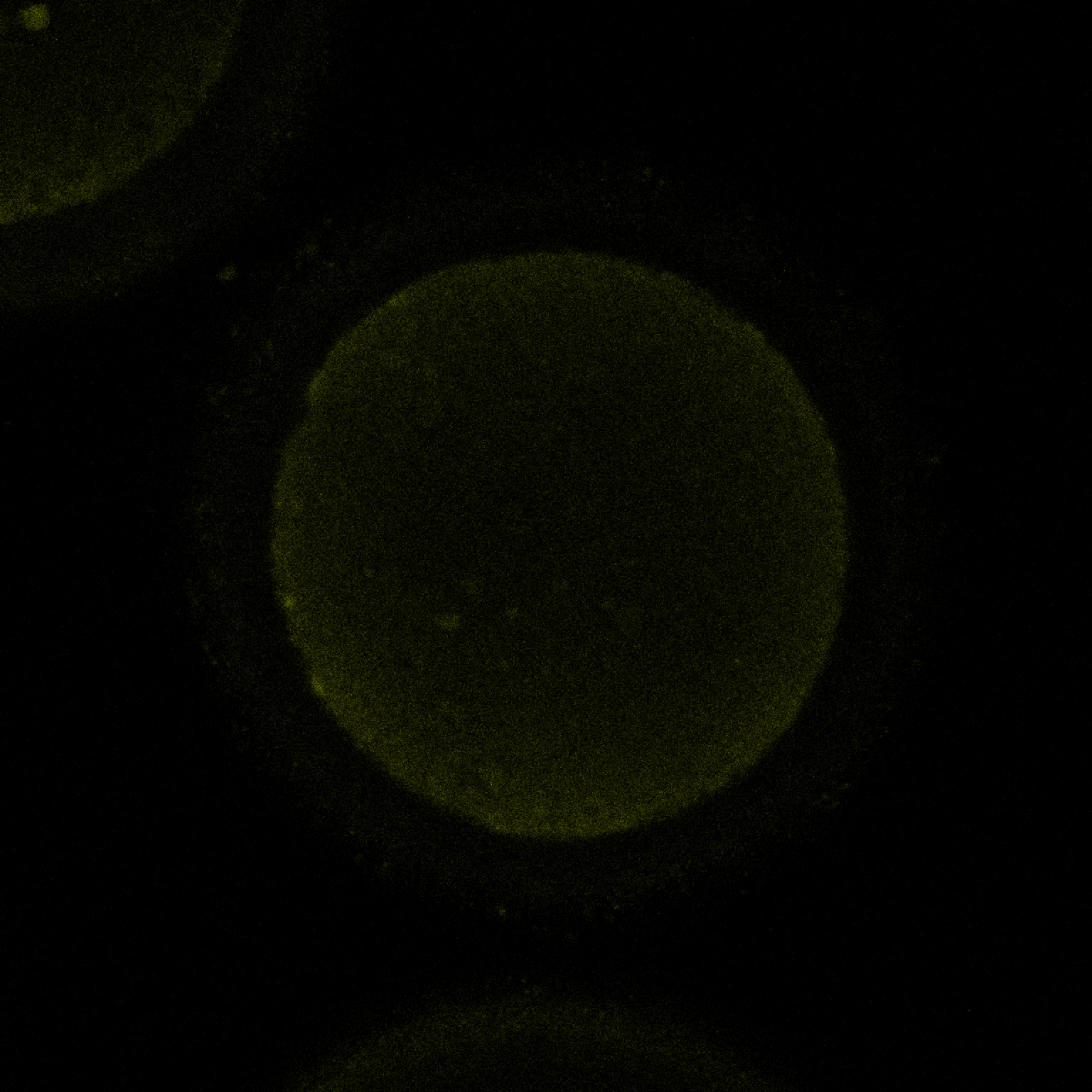

Supplement: Supplementary file 5 — Source data Fig. 1 [file 44318_2026_813_MOESM5_ESM.zip › Figure 1/1H/Proteostat 28.jpg]

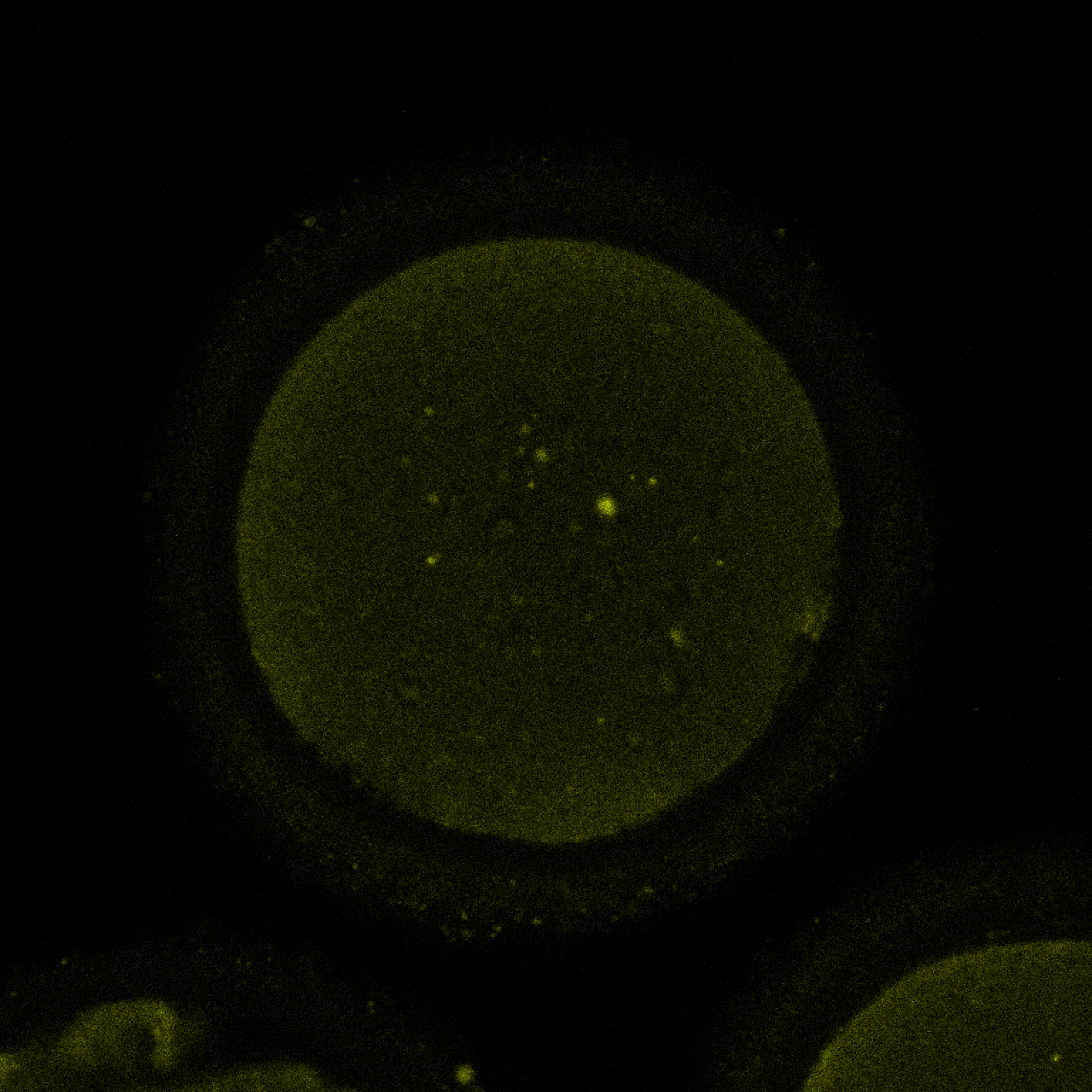

Supplement: Supplementary file 5 — Source data Fig. 1 [file 44318_2026_813_MOESM5_ESM.zip › Figure 1/1H/Proteostat 38.jpg]

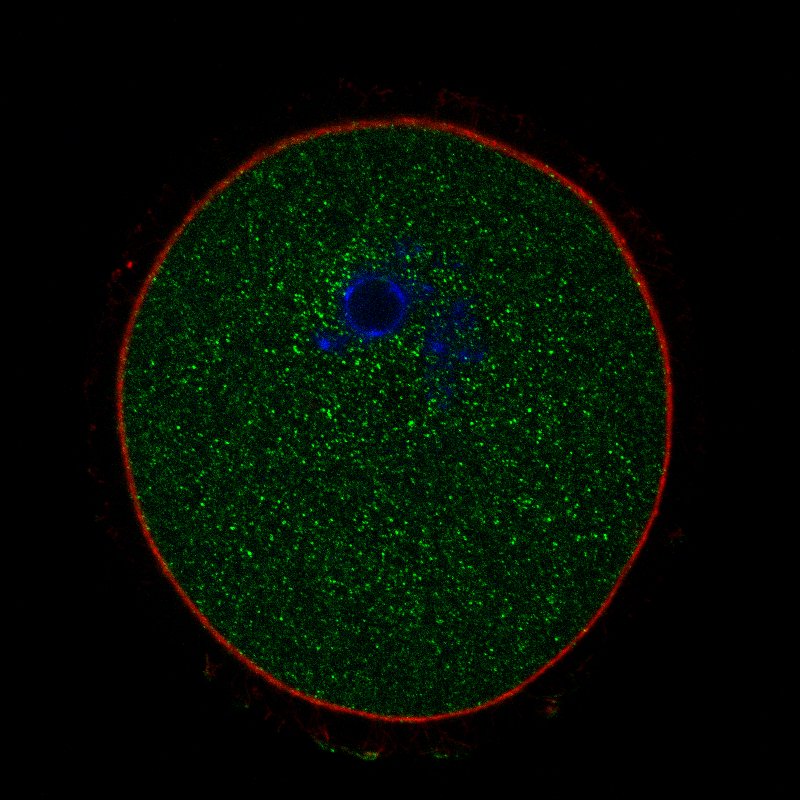

Supplement: Supplementary file 6 — Source data Fig. 2 [file 44318_2026_813_MOESM6_ESM.zip › Figure 2/2C/cKO.jpg]

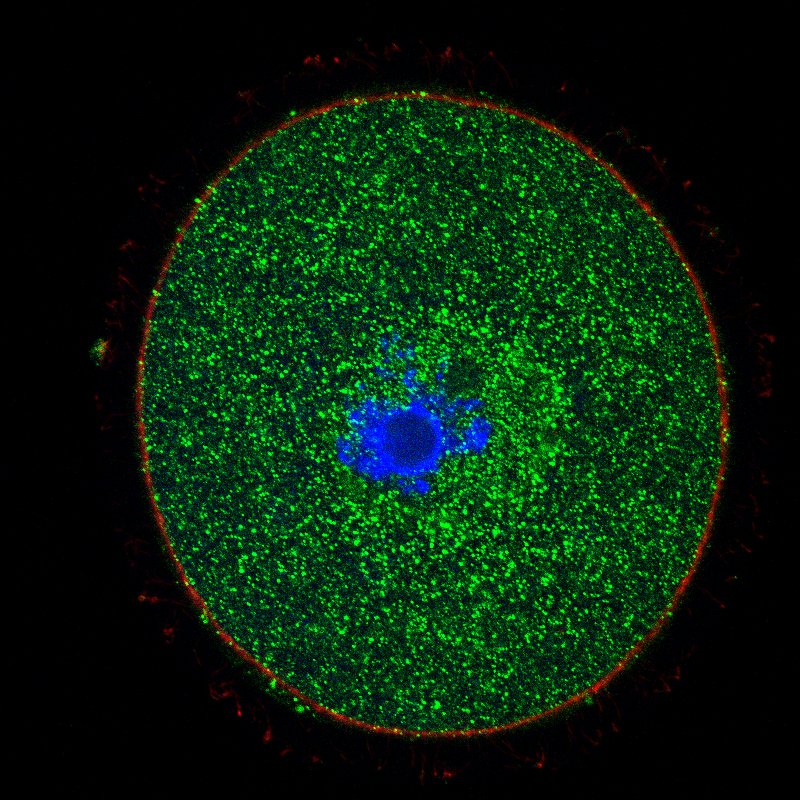

Supplement: Supplementary file 6 — Source data Fig. 2 [file 44318_2026_813_MOESM6_ESM.zip › Figure 2/2C/WT.jpg]

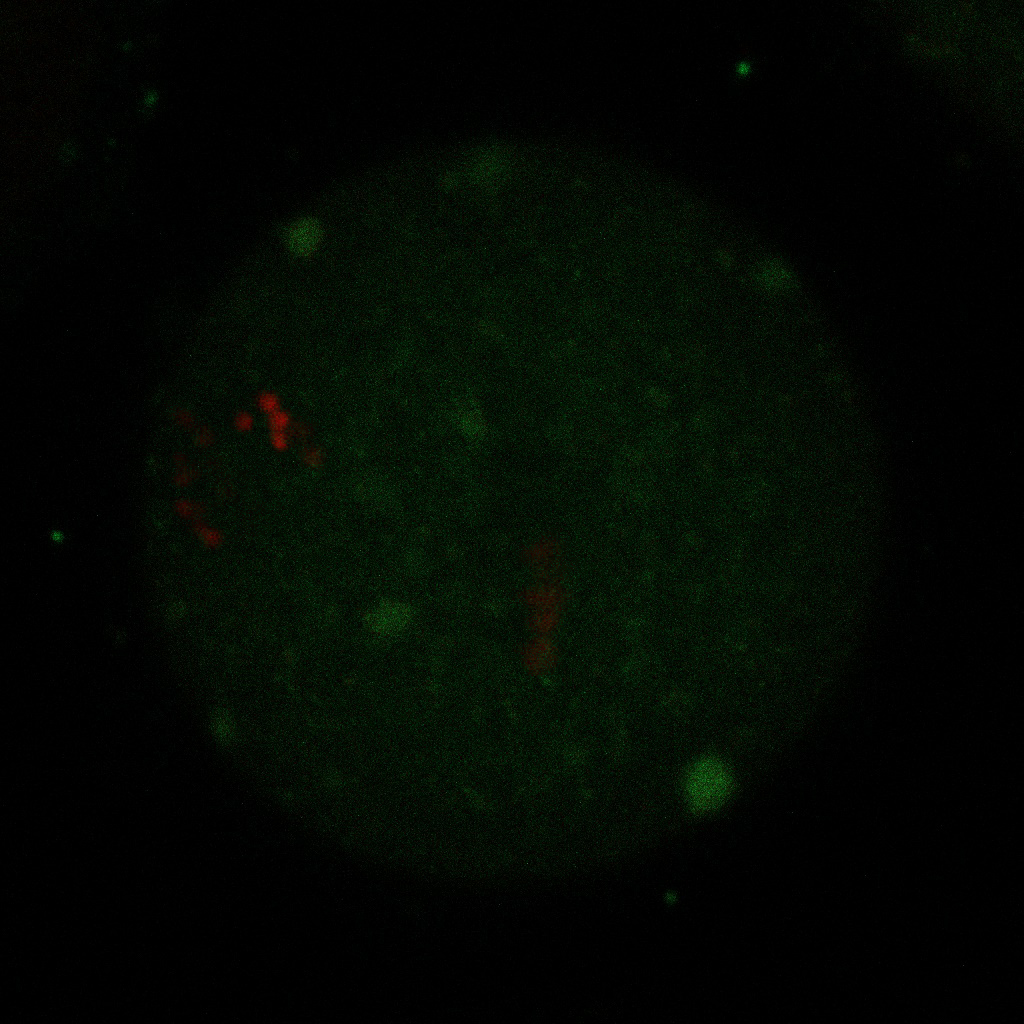

Supplement: Supplementary file 6 — Source data Fig. 2 [file 44318_2026_813_MOESM6_ESM.zip › Figure 2/2E/cKO egg.jpg]

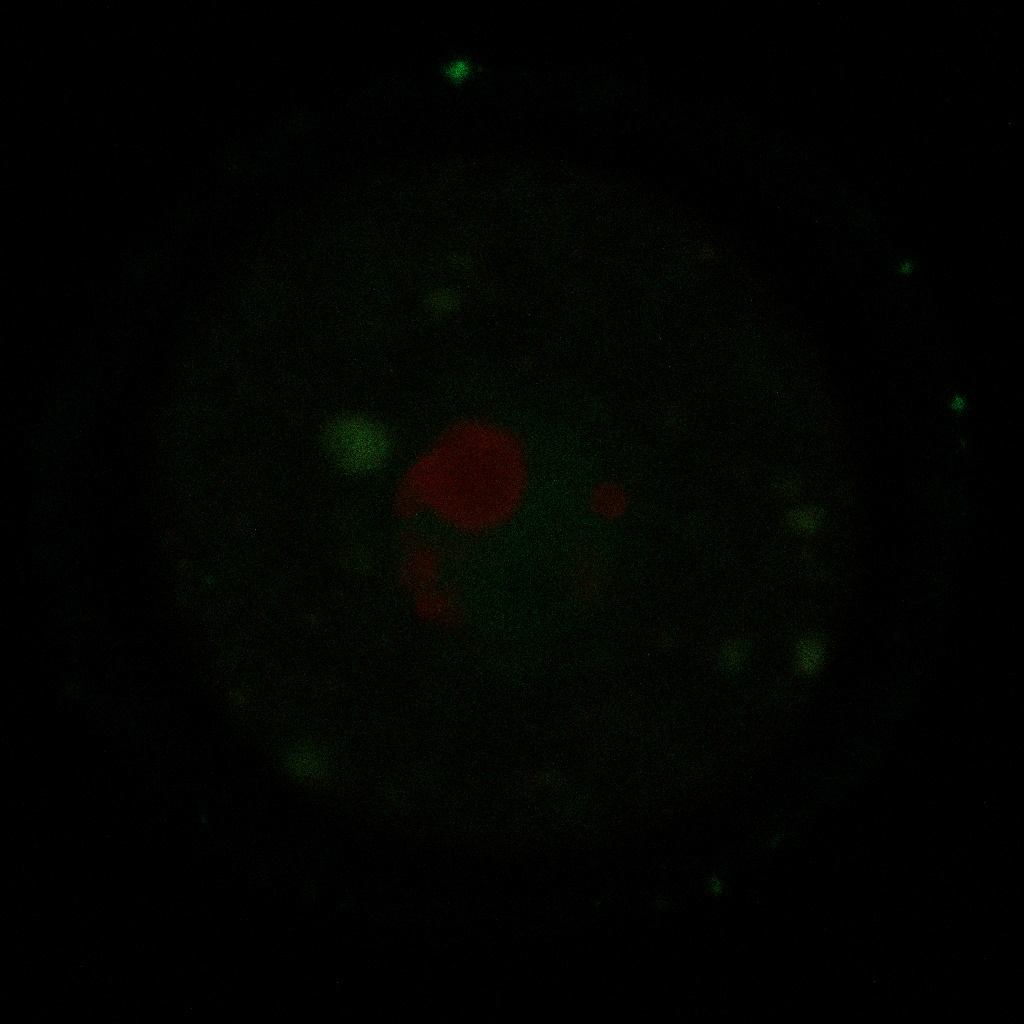

Supplement: Supplementary file 6 — Source data Fig. 2 [file 44318_2026_813_MOESM6_ESM.zip › Figure 2/2E/cKO oocyte.jpg]

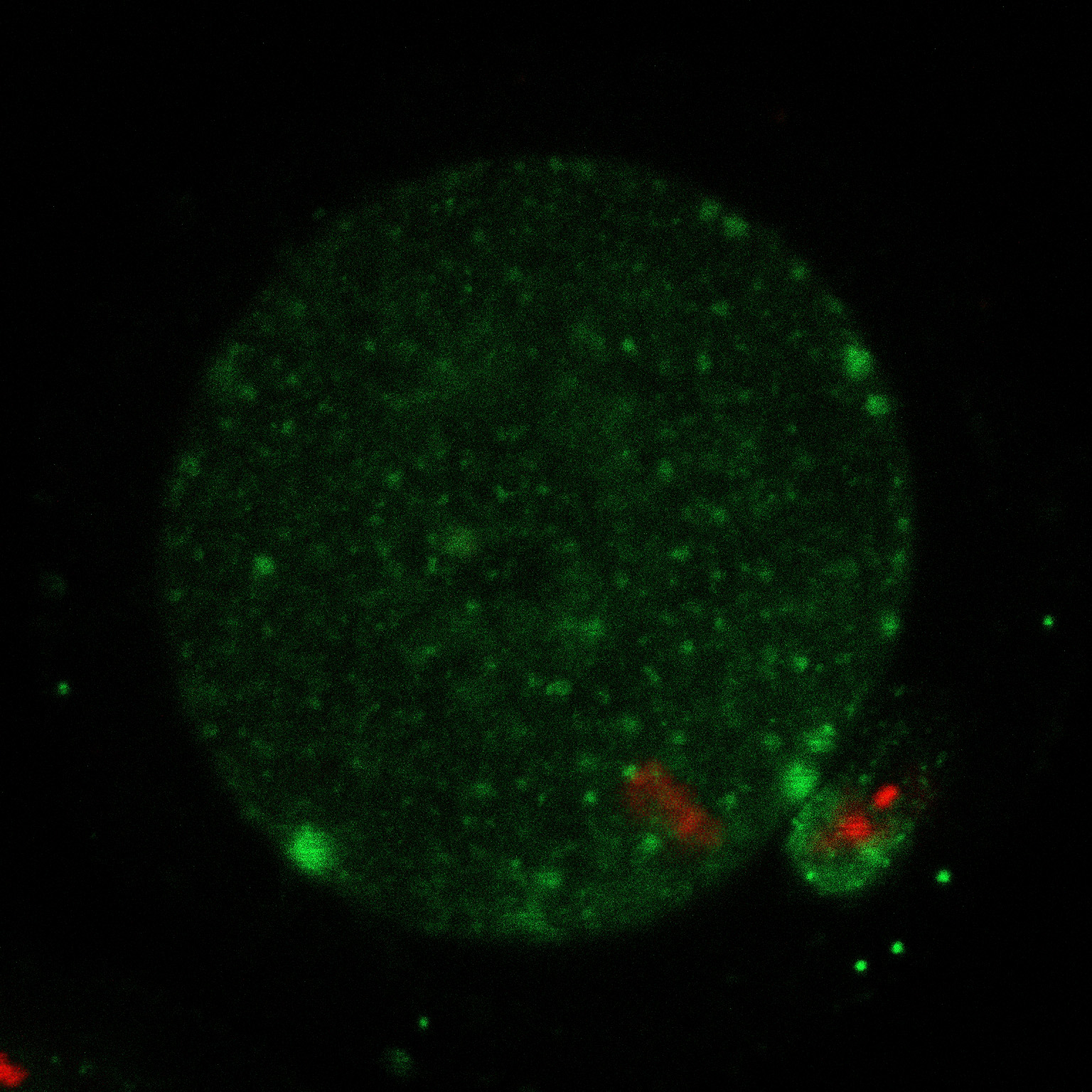

Supplement: Supplementary file 6 — Source data Fig. 2 [file 44318_2026_813_MOESM6_ESM.zip › Figure 2/2E/WT egg.jpg]

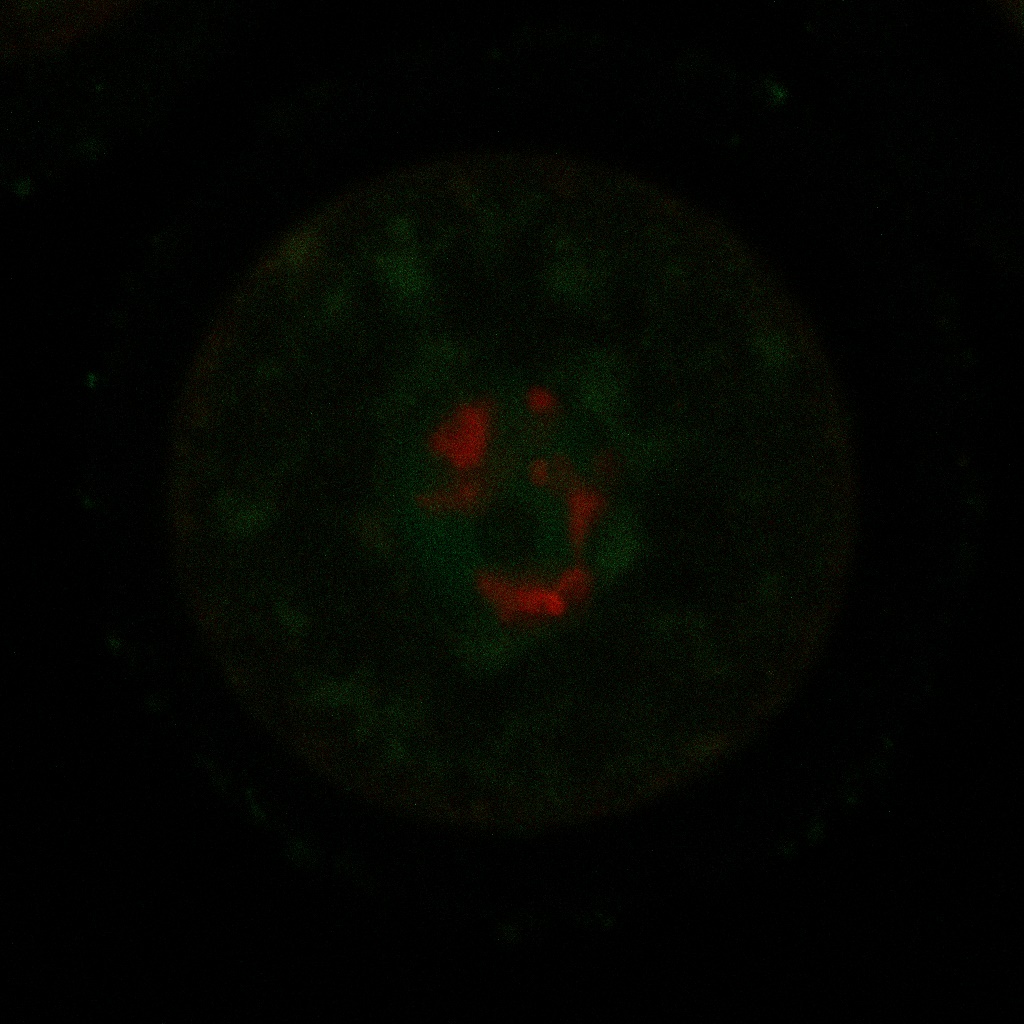

Supplement: Supplementary file 6 — Source data Fig. 2 [file 44318_2026_813_MOESM6_ESM.zip › Figure 2/2E/WT oocyte.jpg]

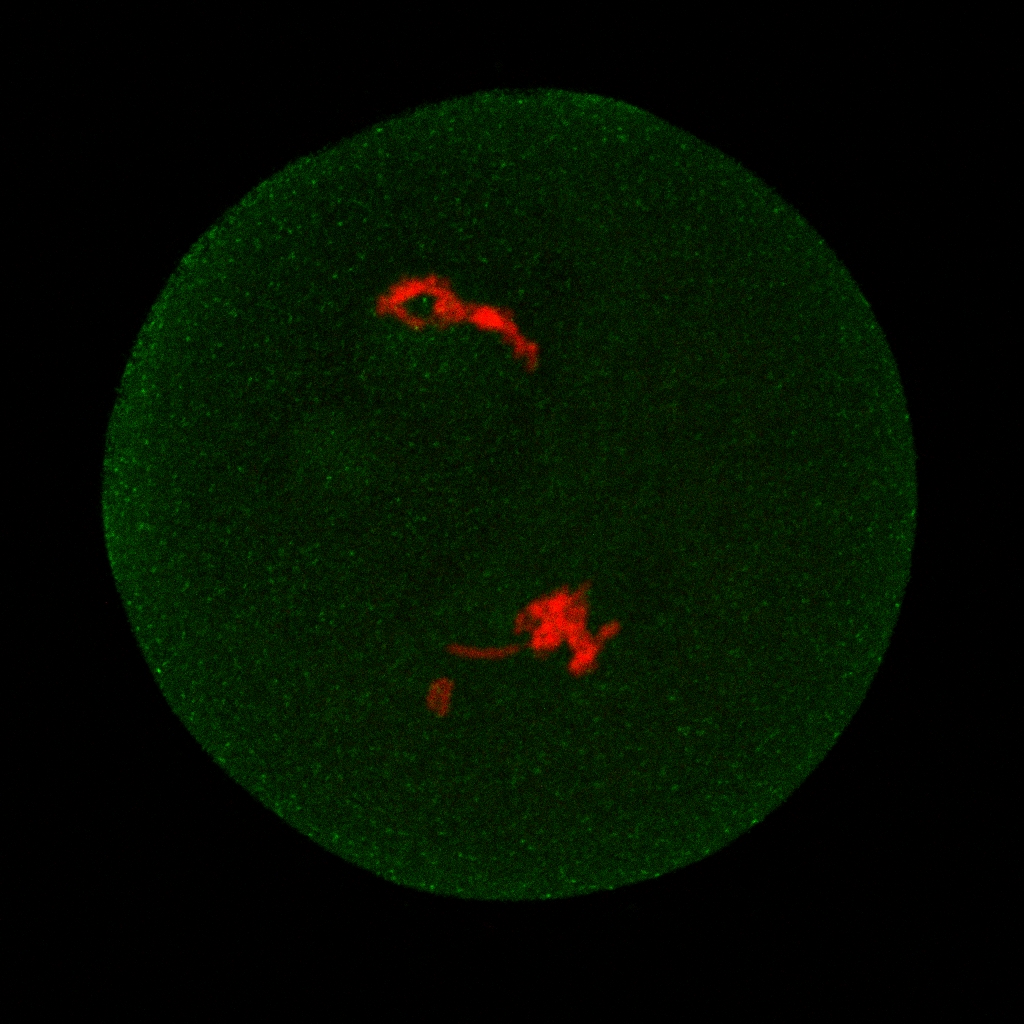

Supplement: Supplementary file 6 — Source data Fig. 2 [file 44318_2026_813_MOESM6_ESM.zip › Figure 2/2G/cKO egg.jpg]

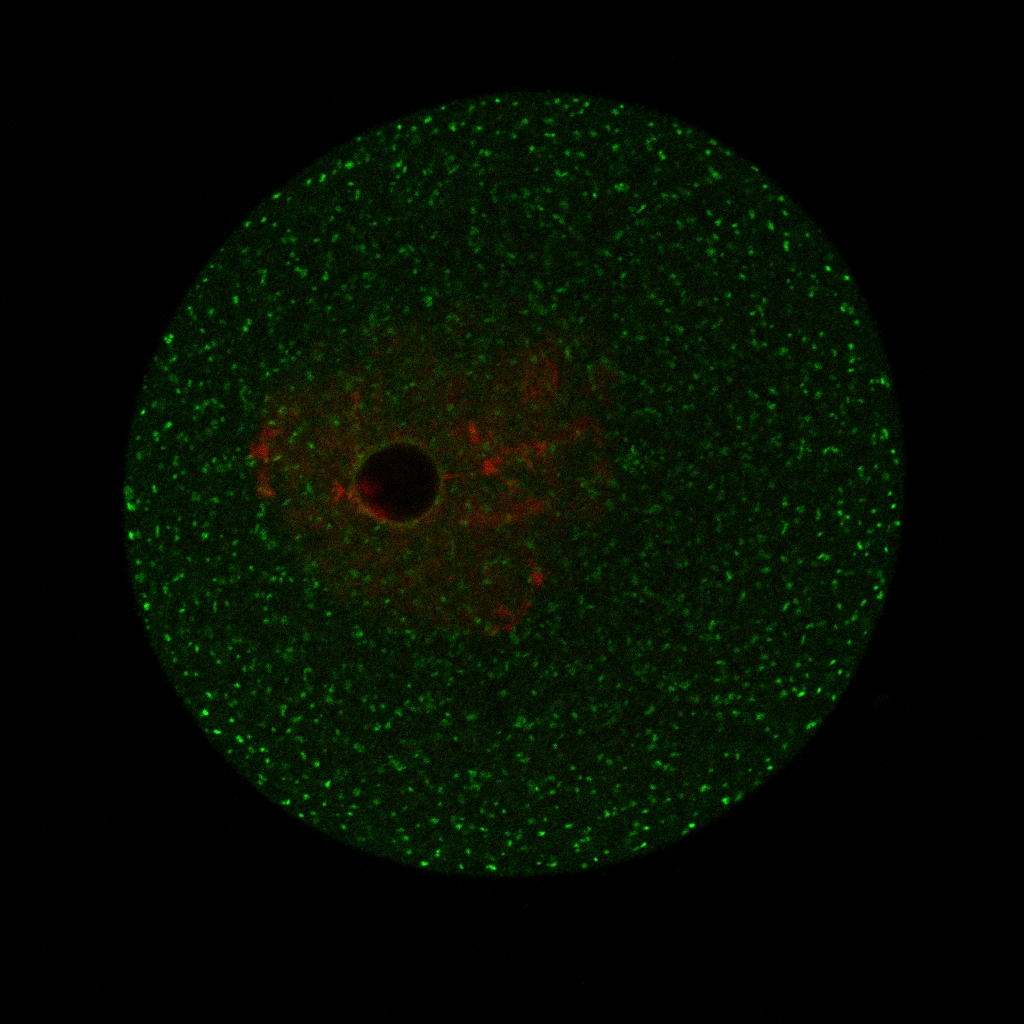

Supplement: Supplementary file 6 — Source data Fig. 2 [file 44318_2026_813_MOESM6_ESM.zip › Figure 2/2G/cKO oocyte.jpg]

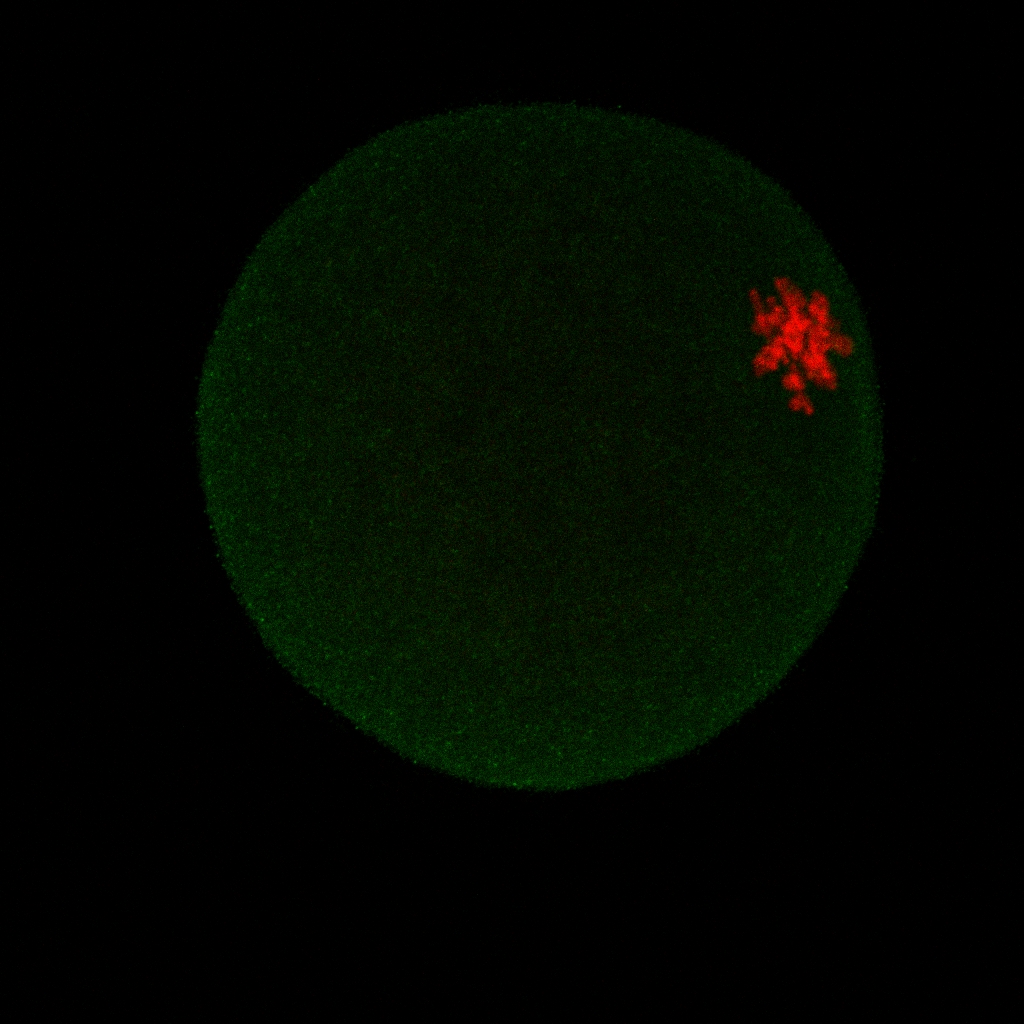

Supplement: Supplementary file 6 — Source data Fig. 2 [file 44318_2026_813_MOESM6_ESM.zip › Figure 2/2G/WT egg.jpg]

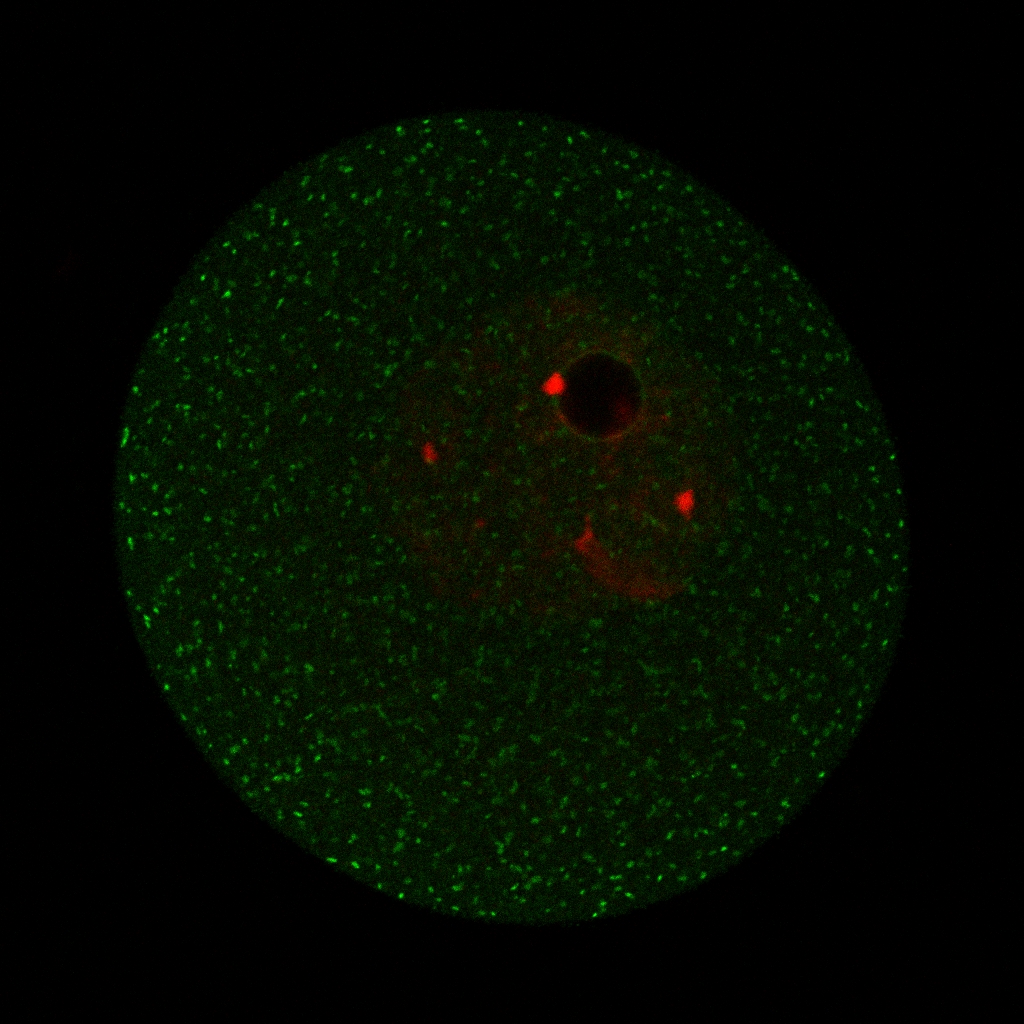

Supplement: Supplementary file 6 — Source data Fig. 2 [file 44318_2026_813_MOESM6_ESM.zip › Figure 2/2G/WT oocyte.jpg]

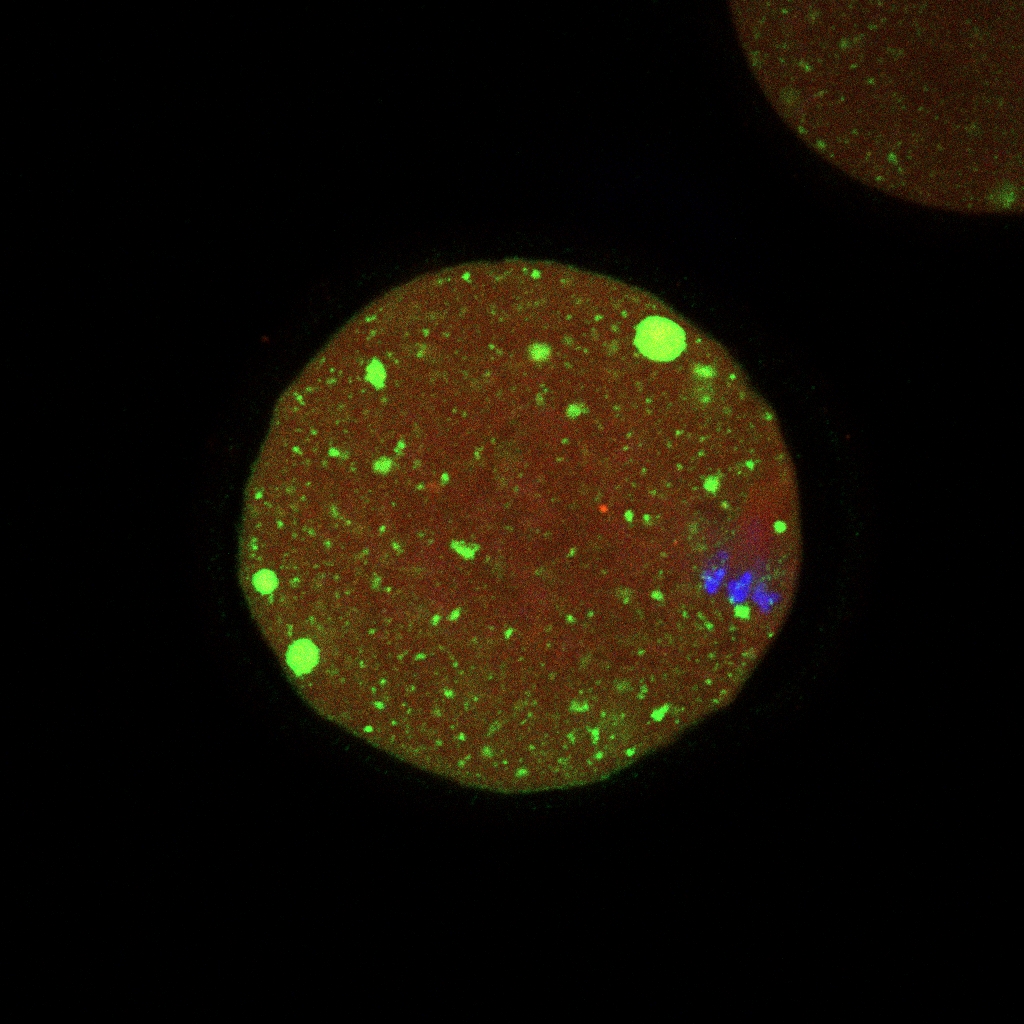

Supplement: Supplementary file 7 — Source data Fig. 3 [file 44318_2026_813_MOESM7_ESM.zip › Figure 3/3A/cKO.jpg]

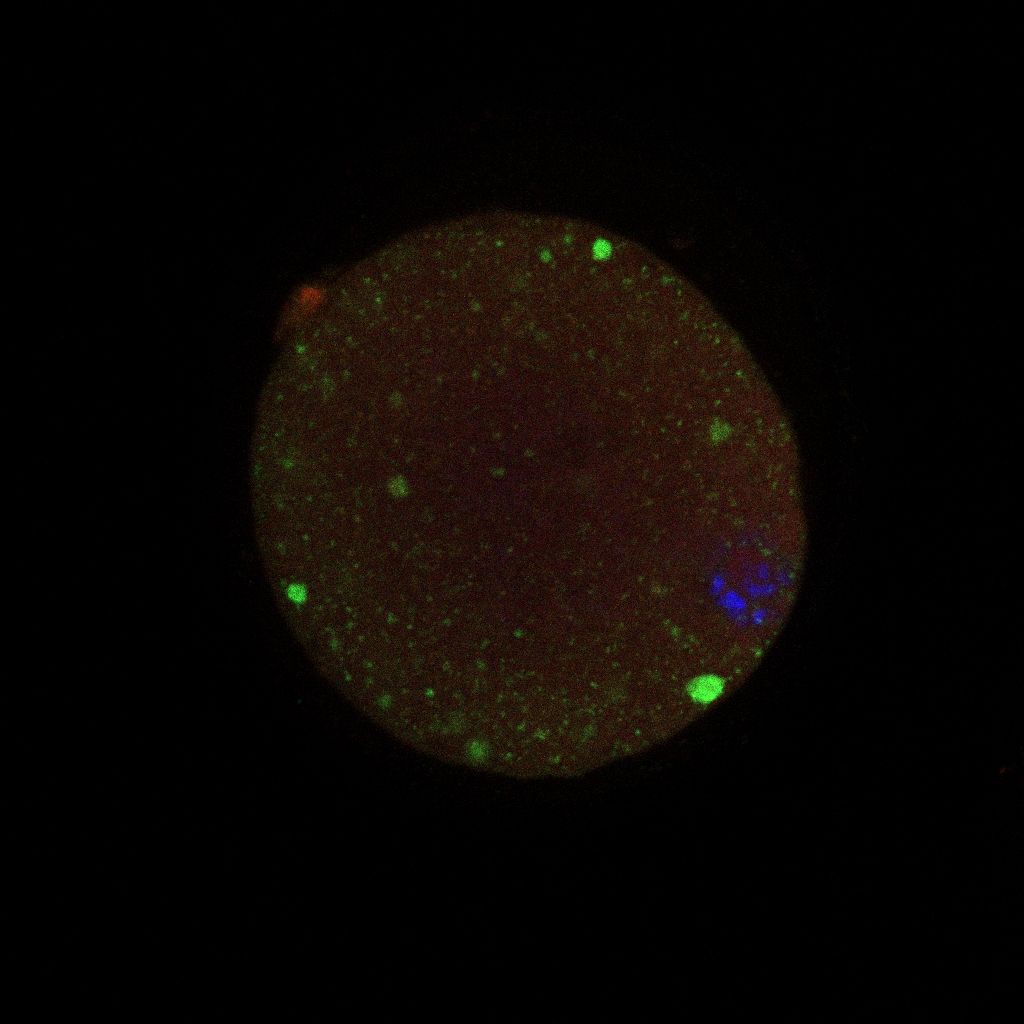

Supplement: Supplementary file 7 — Source data Fig. 3 [file 44318_2026_813_MOESM7_ESM.zip › Figure 3/3A/WT.jpg]

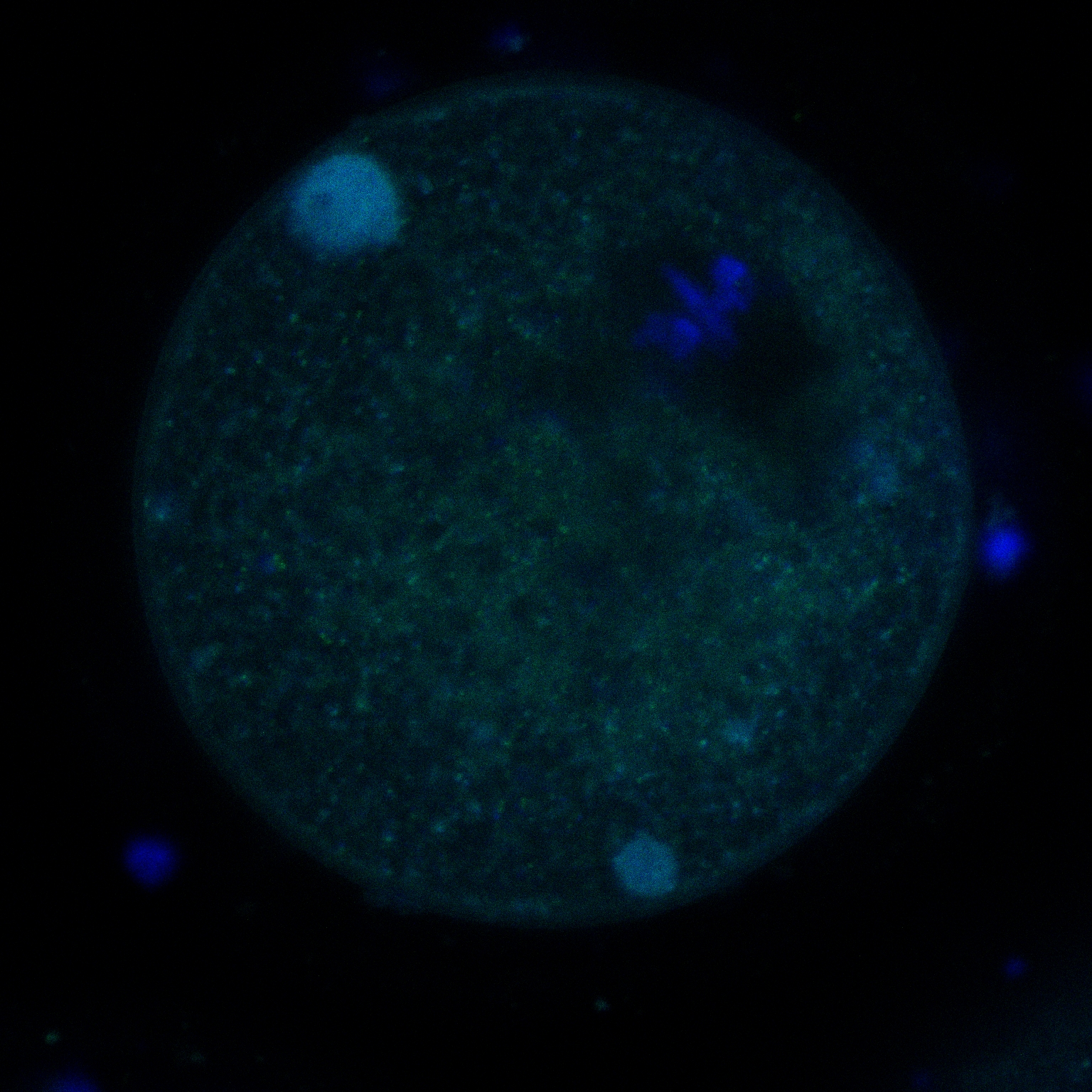

Supplement: Supplementary file 7 — Source data Fig. 3 [file 44318_2026_813_MOESM7_ESM.zip › Figure 3/3D/cKO.jpg]

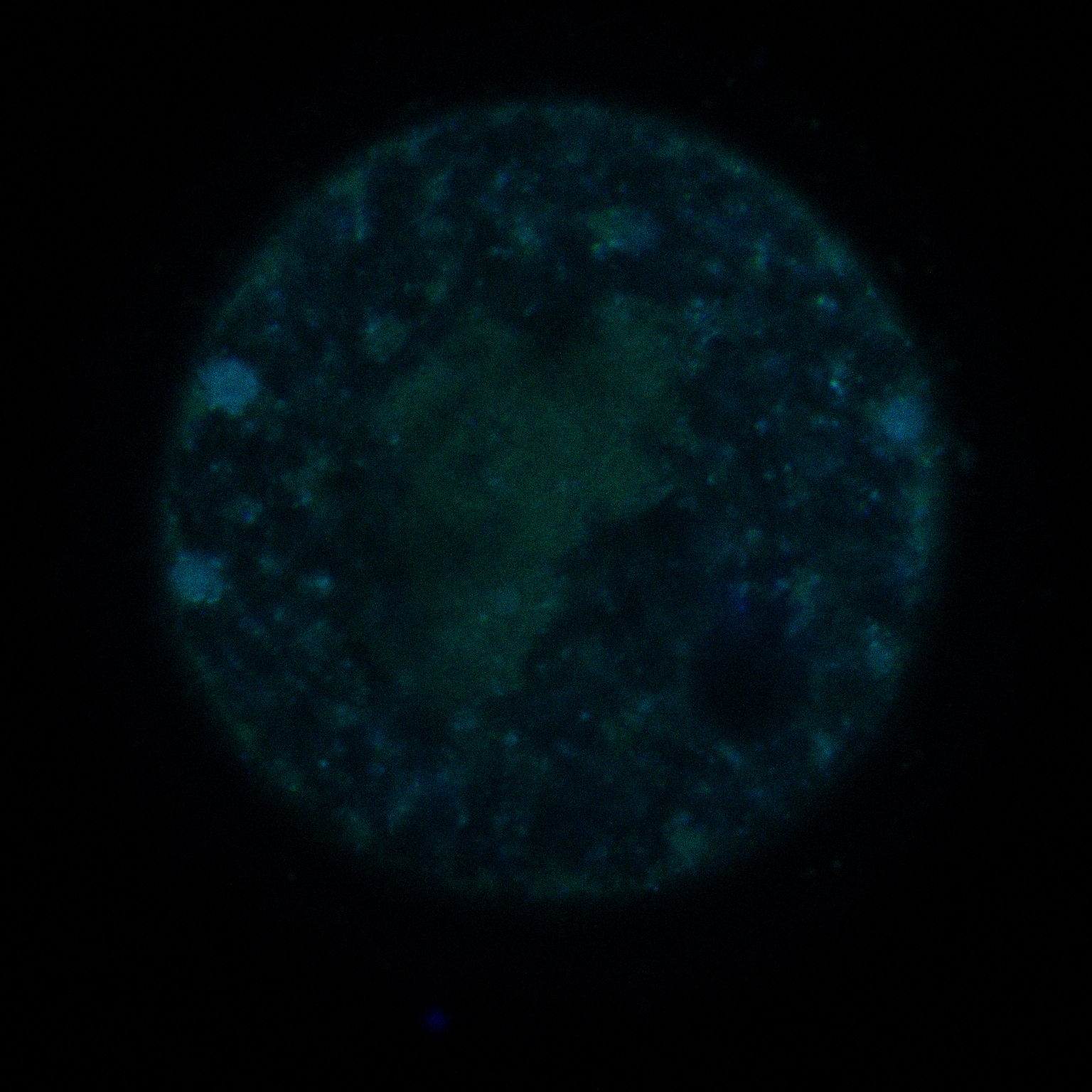

Supplement: Supplementary file 7 — Source data Fig. 3 [file 44318_2026_813_MOESM7_ESM.zip › Figure 3/3D/WT.jpg]

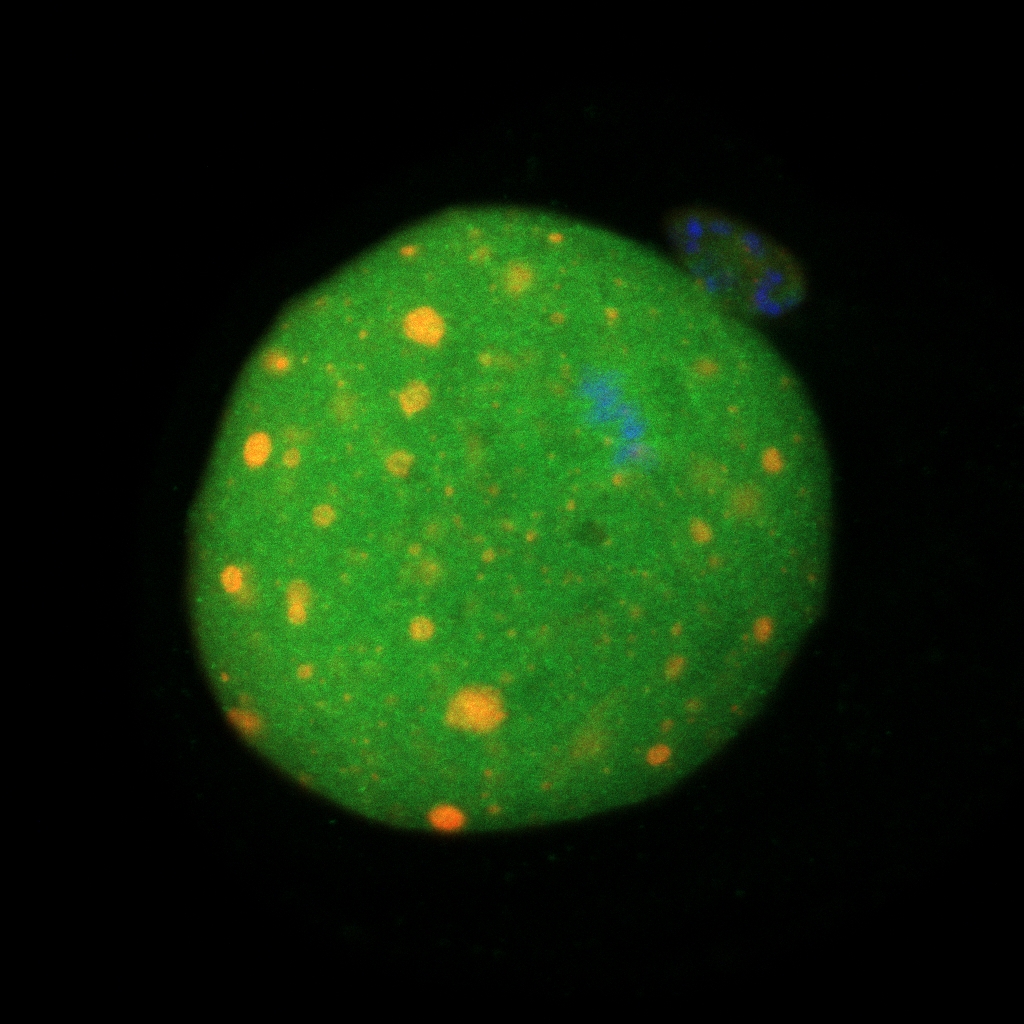

Supplement: Supplementary file 7 — Source data Fig. 3 [file 44318_2026_813_MOESM7_ESM.zip › Figure 3/3F/cKO.jpg]

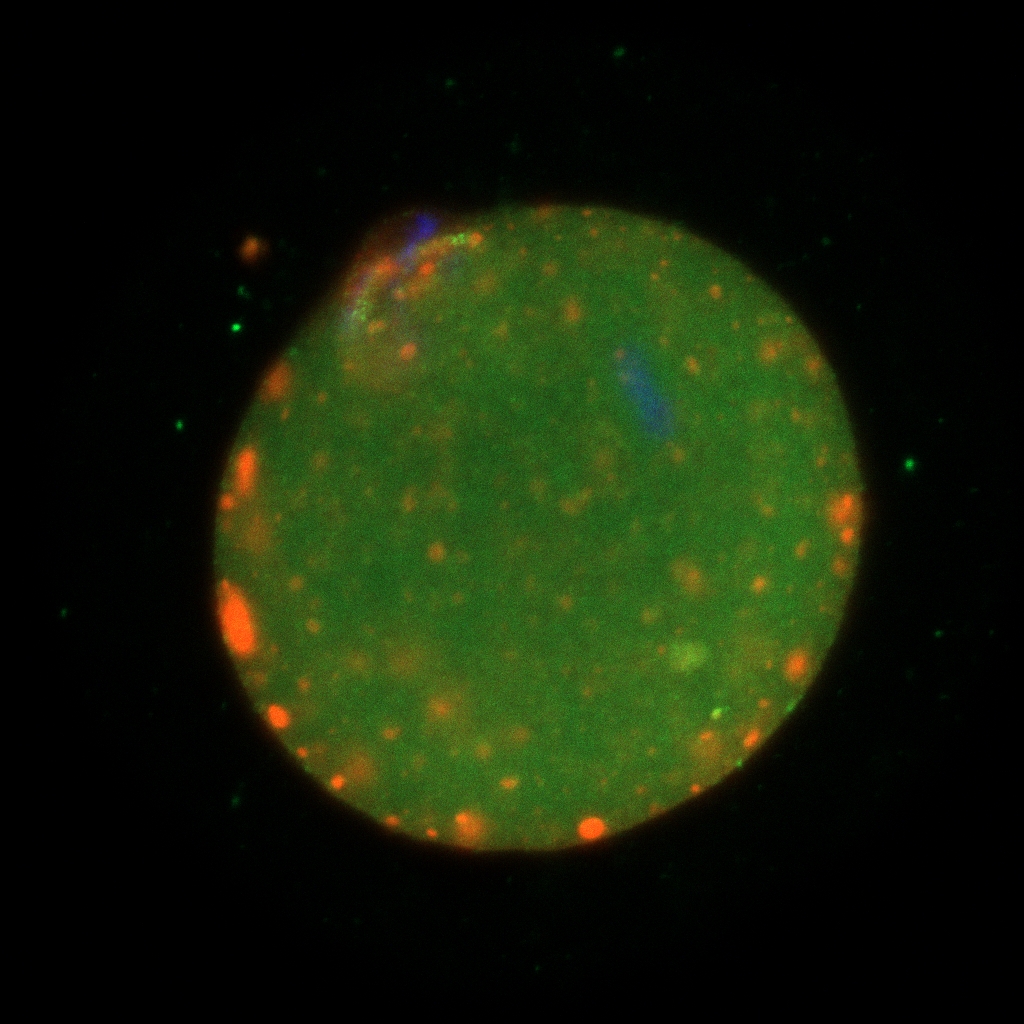

Supplement: Supplementary file 7 — Source data Fig. 3 [file 44318_2026_813_MOESM7_ESM.zip › Figure 3/3F/WT.jpg]

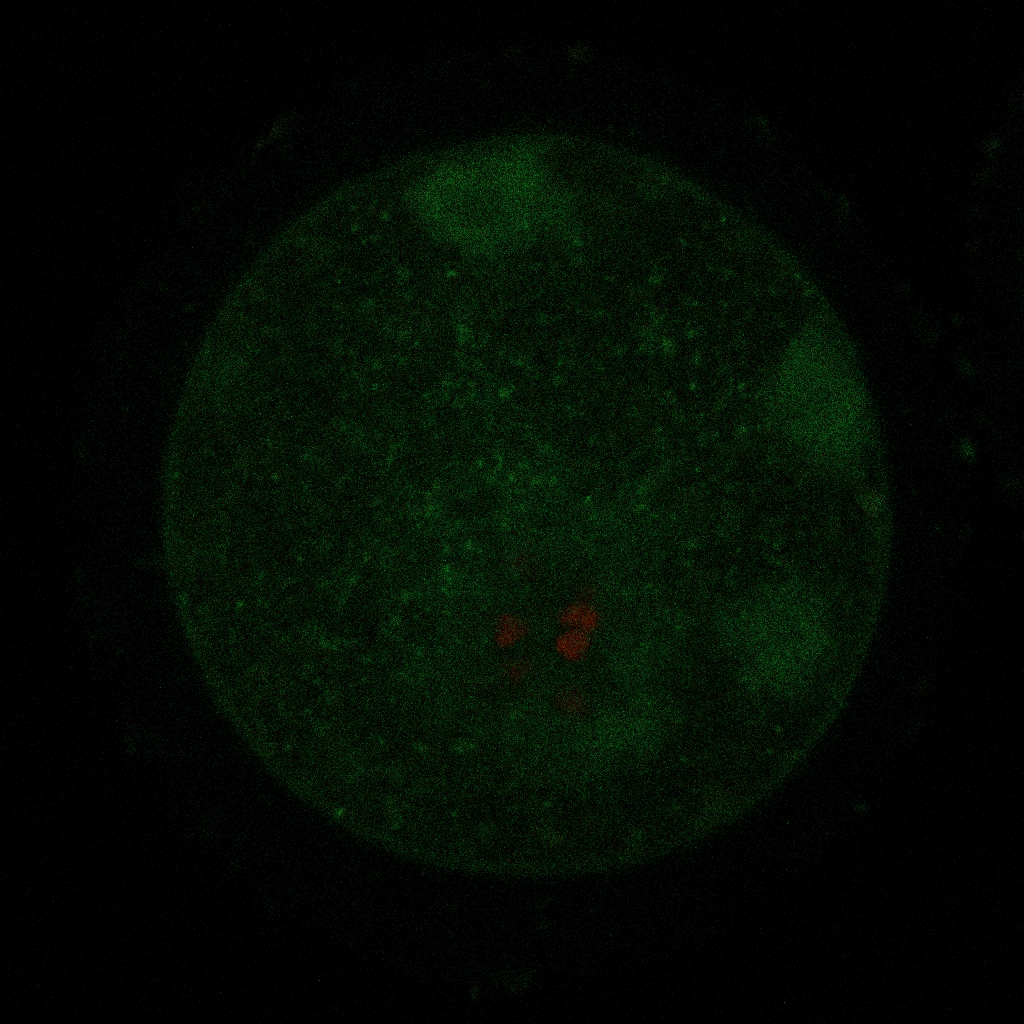

Supplement: Supplementary file 7 — Source data Fig. 3 [file 44318_2026_813_MOESM7_ESM.zip › Figure 3/3K/MG132.jpg]

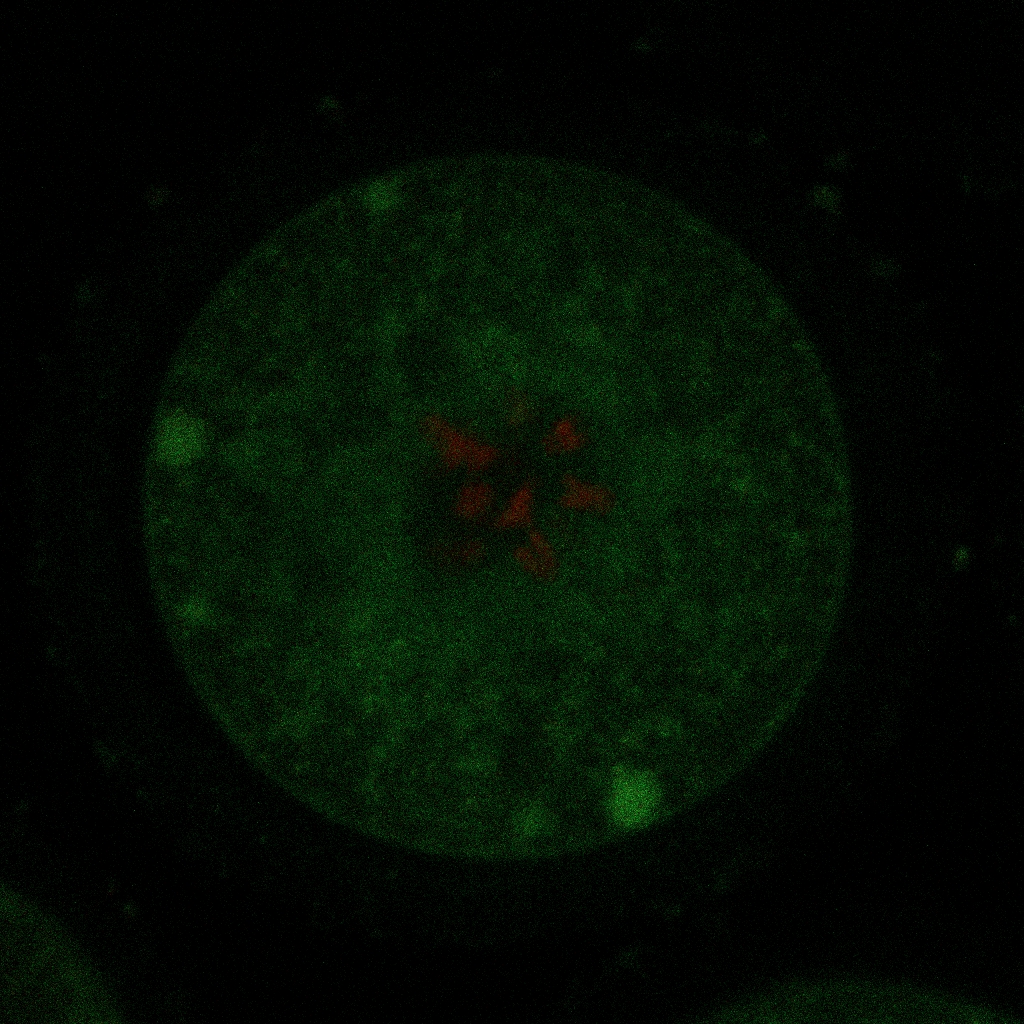

Supplement: Supplementary file 7 — Source data Fig. 3 [file 44318_2026_813_MOESM7_ESM.zip › Figure 3/3K/NC.jpg]

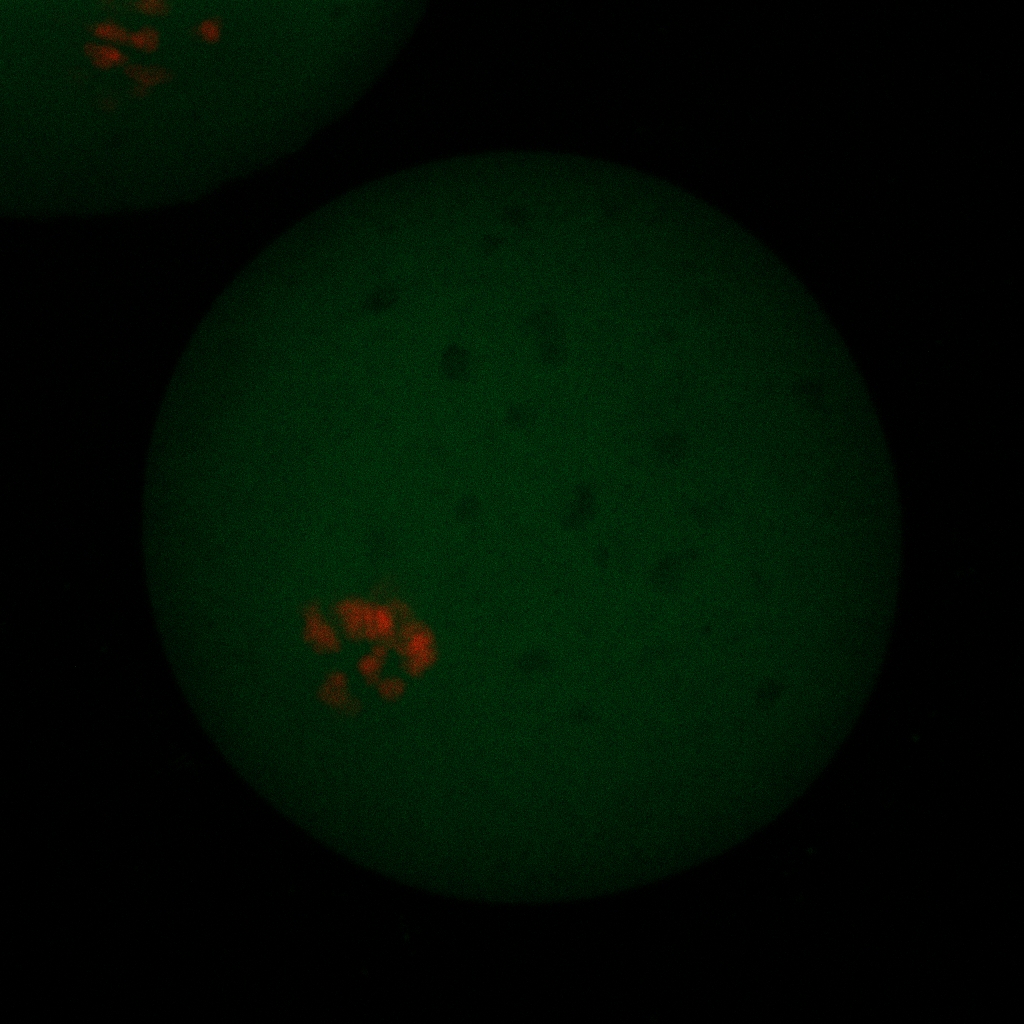

Supplement: Supplementary file 8 — Source data Fig. 4 [file 44318_2026_813_MOESM8_ESM.zip › Figure 4/4J/cKO GVBD.jpg]

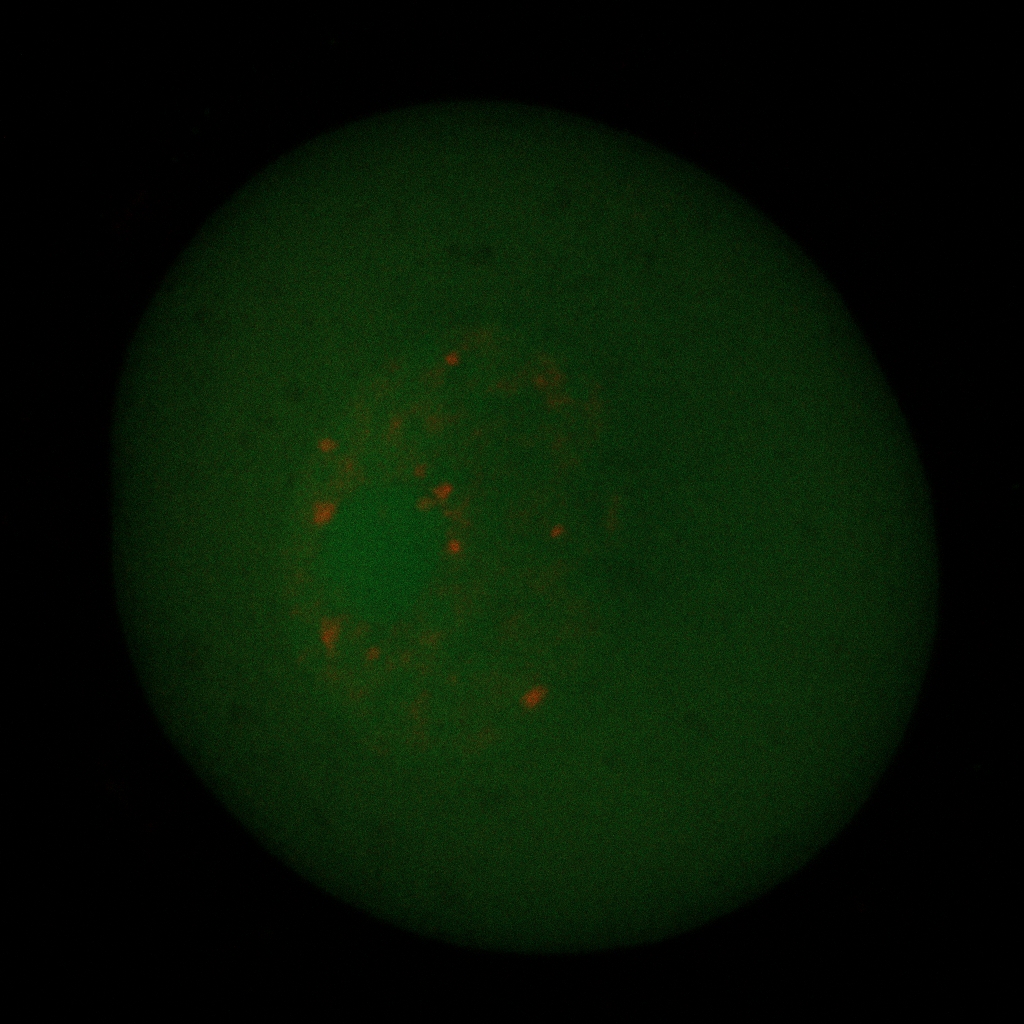

Supplement: Supplementary file 8 — Source data Fig. 4 [file 44318_2026_813_MOESM8_ESM.zip › Figure 4/4J/cKO oocyte.jpg]

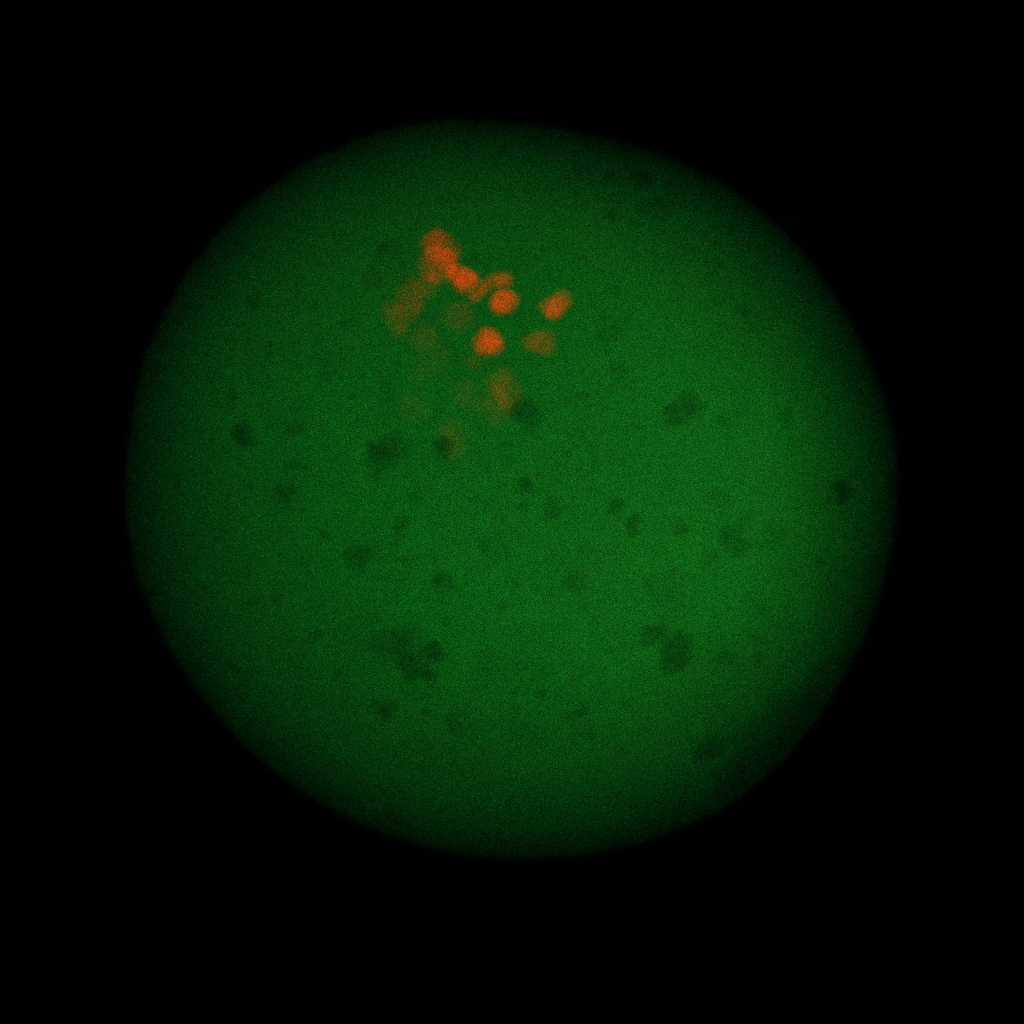

Supplement: Supplementary file 8 — Source data Fig. 4 [file 44318_2026_813_MOESM8_ESM.zip › Figure 4/4J/WT GVBD.jpg]

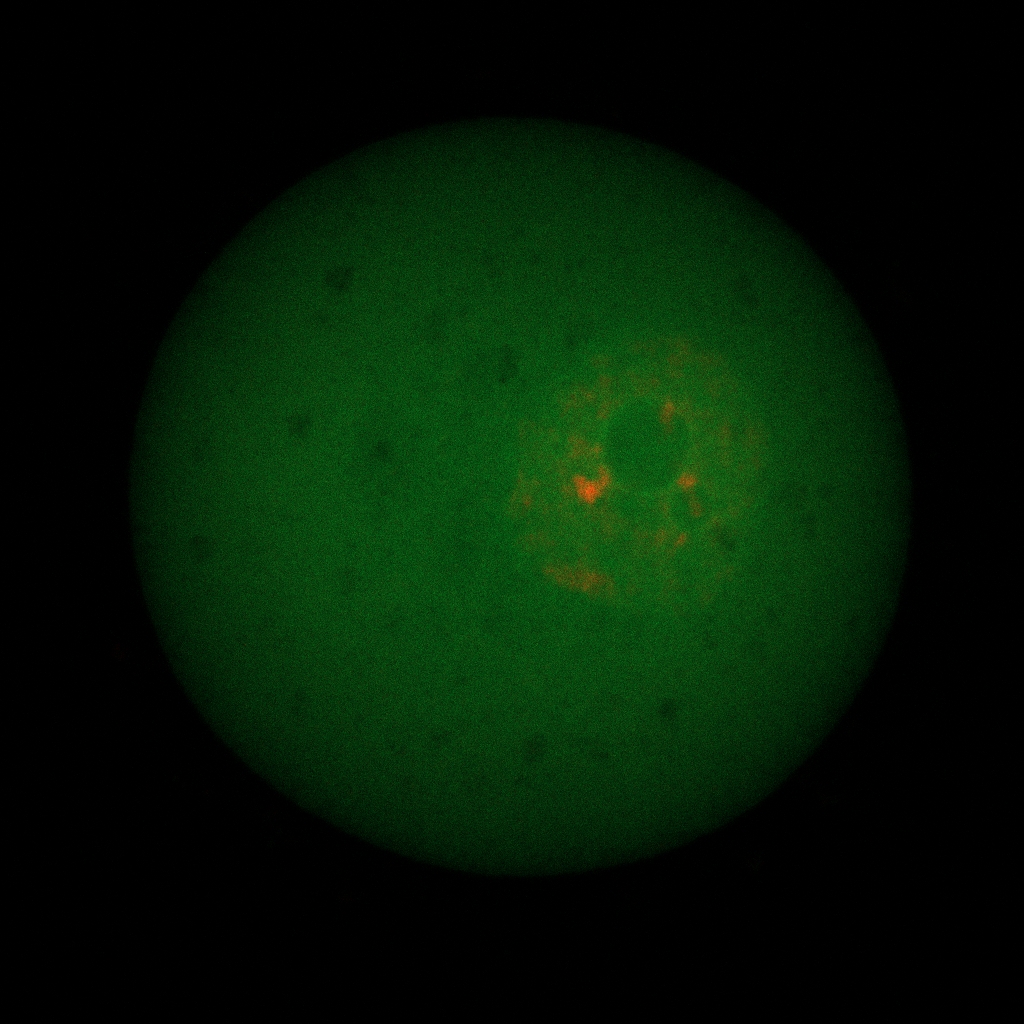

Supplement: Supplementary file 8 — Source data Fig. 4 [file 44318_2026_813_MOESM8_ESM.zip › Figure 4/4J/WT oocyte.jpg]

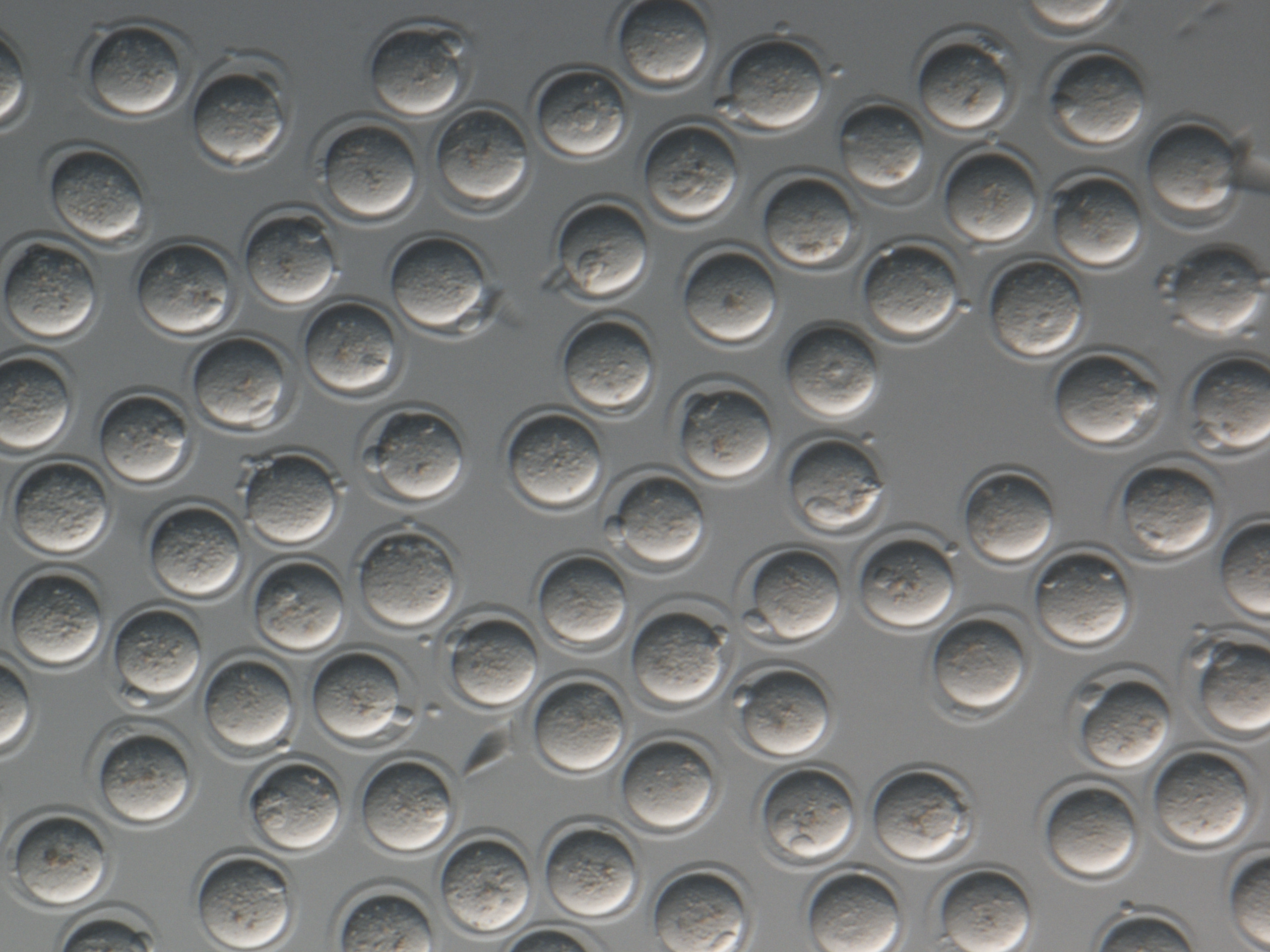

Supplement: Supplementary file 9 — Source data Fig. 5 [file 44318_2026_813_MOESM9_ESM.zip › Figure 5/5C/cKO.tif]

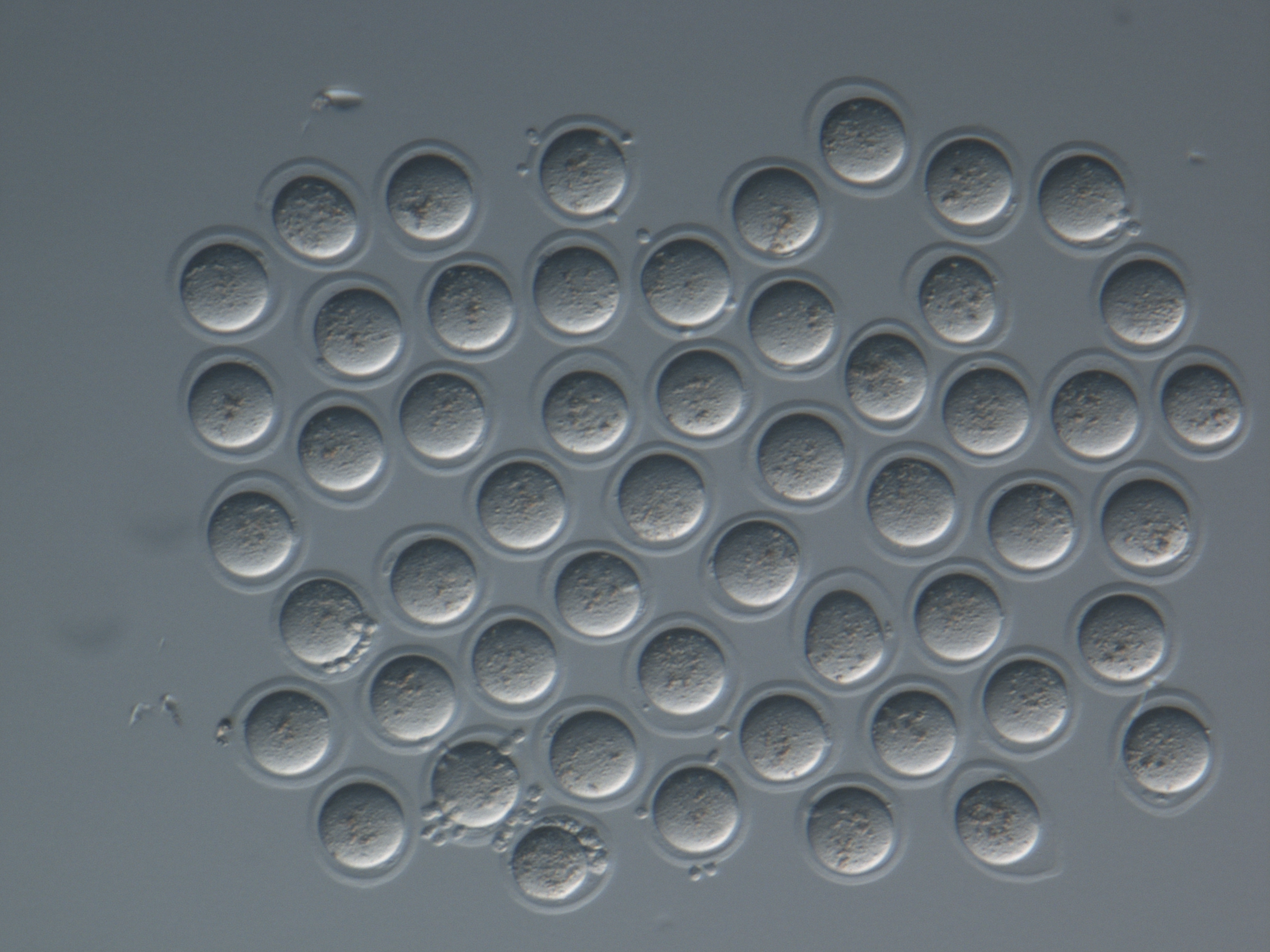

Supplement: Supplementary file 9 — Source data Fig. 5 [file 44318_2026_813_MOESM9_ESM.zip › Figure 5/5C/WT.tif]

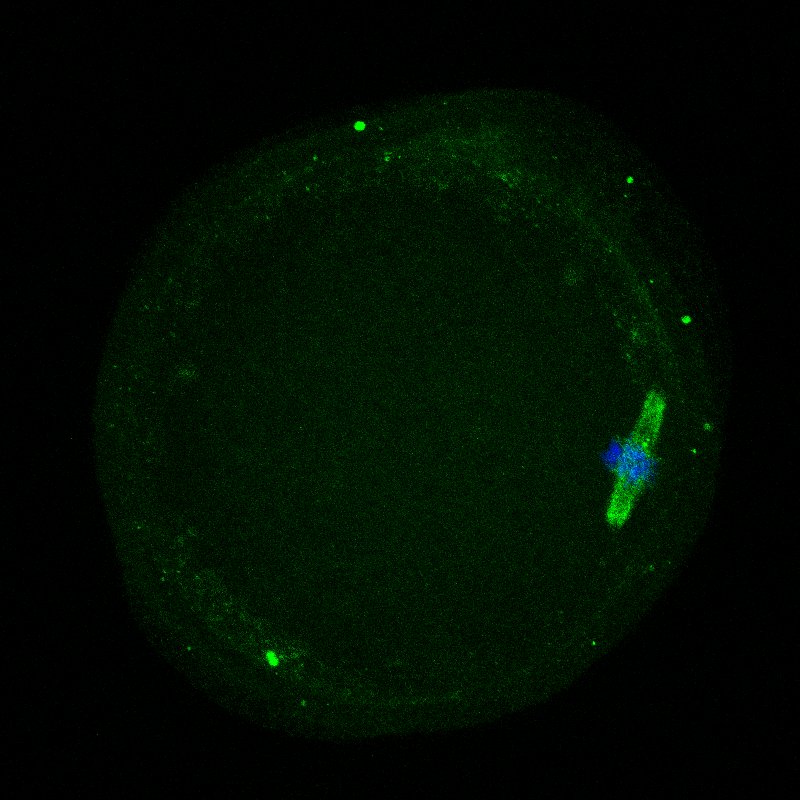

Supplement: Supplementary file 9 — Source data Fig. 5 [file 44318_2026_813_MOESM9_ESM.zip › Figure 5/5E/cKO-1-MII-actub, DNA.jpg]

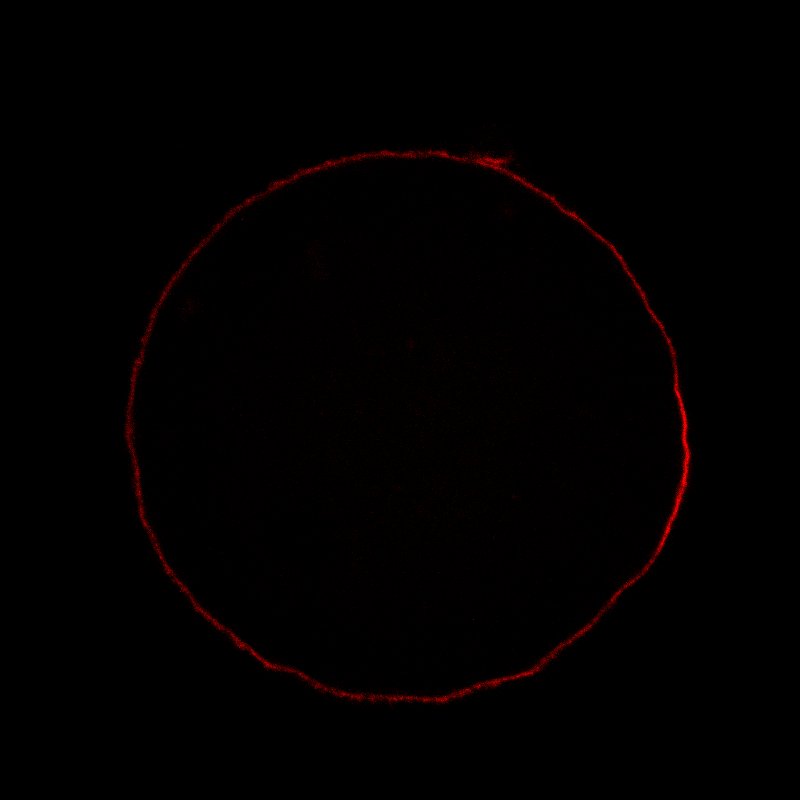

Supplement: Supplementary file 9 — Source data Fig. 5 [file 44318_2026_813_MOESM9_ESM.zip › Figure 5/5E/cKO-1-MII-phalloidin.jpg]

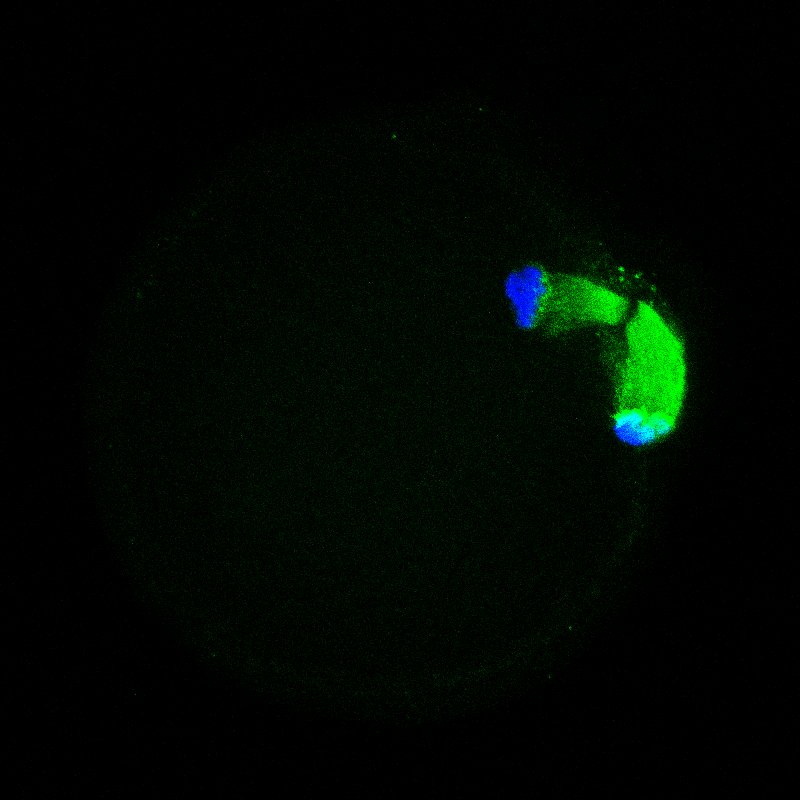

Supplement: Supplementary file 9 — Source data Fig. 5 [file 44318_2026_813_MOESM9_ESM.zip › Figure 5/5E/cKO-2-TI-actub, DNA.jpg]

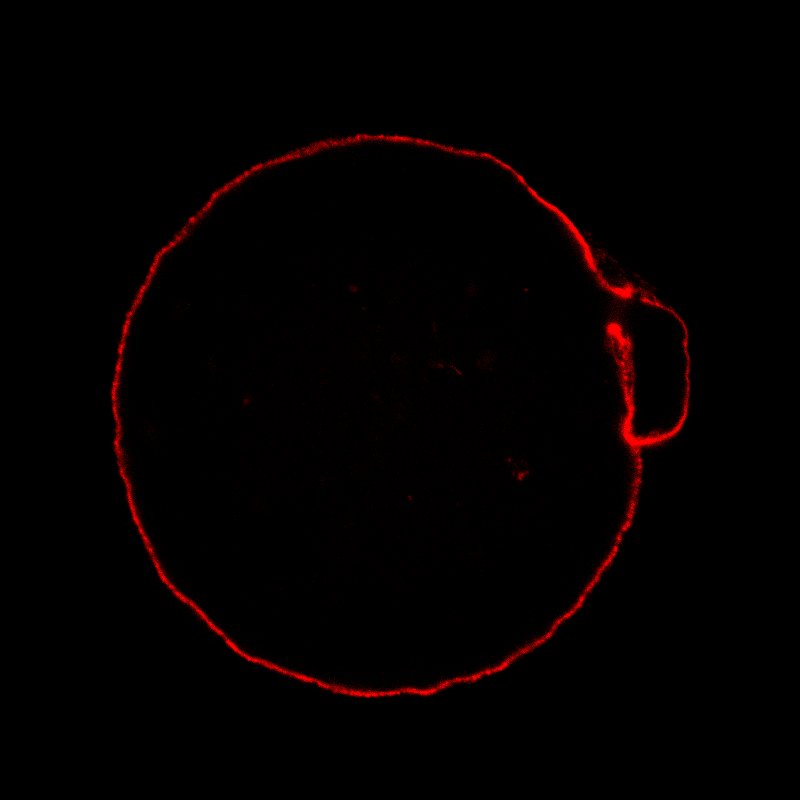

Supplement: Supplementary file 9 — Source data Fig. 5 [file 44318_2026_813_MOESM9_ESM.zip › Figure 5/5E/cKO-2-TI-phalloidin.jpg]

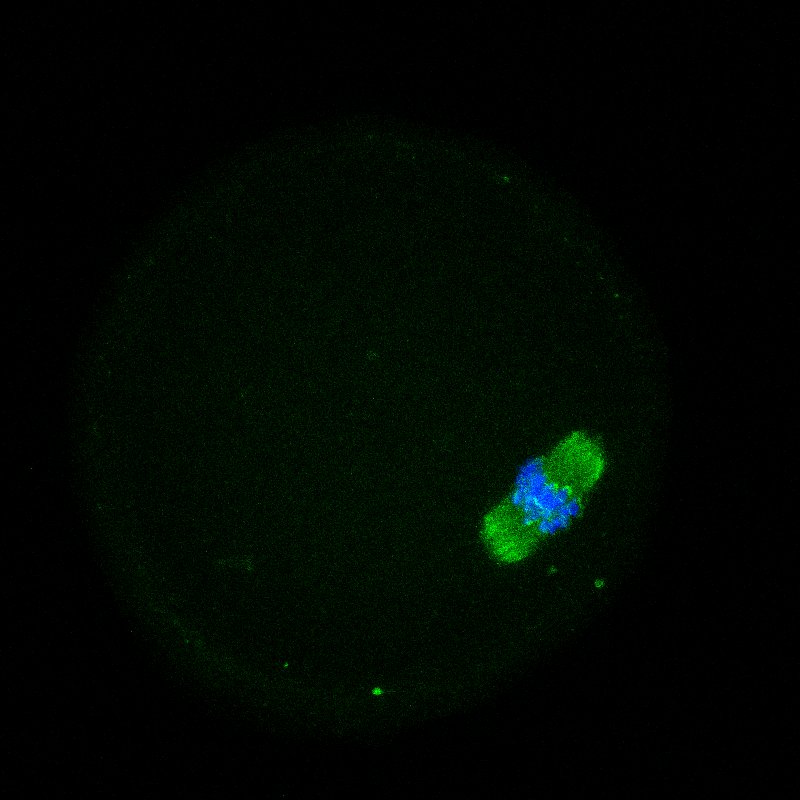

Supplement: Supplementary file 9 — Source data Fig. 5 [file 44318_2026_813_MOESM9_ESM.zip › Figure 5/5E/cKO-3-MI-actub, DNA.jpg]

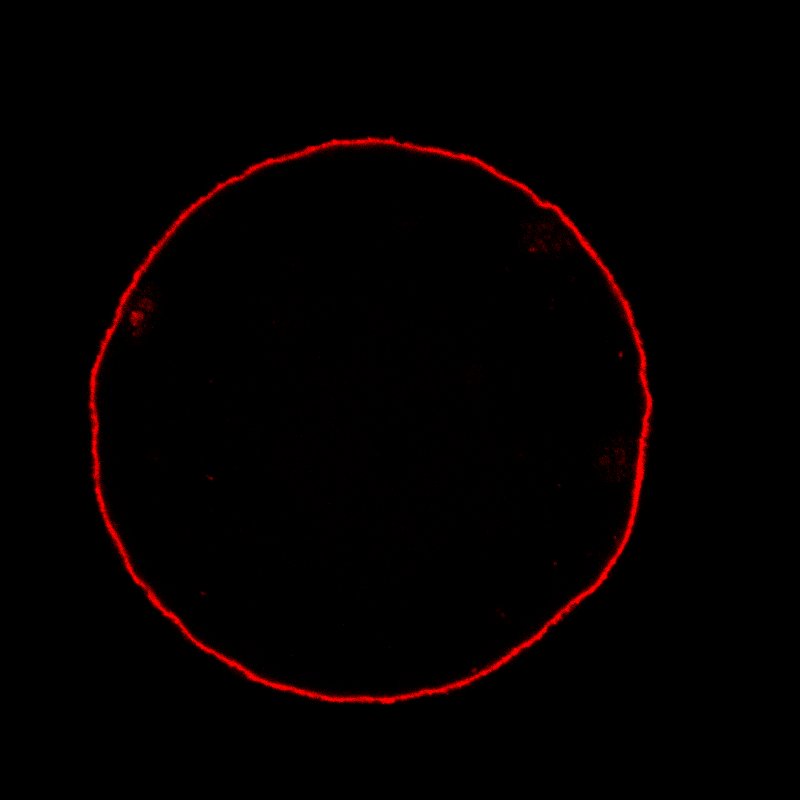

Supplement: Supplementary file 9 — Source data Fig. 5 [file 44318_2026_813_MOESM9_ESM.zip › Figure 5/5E/cKO-3-MI-phalloidin.jpg]

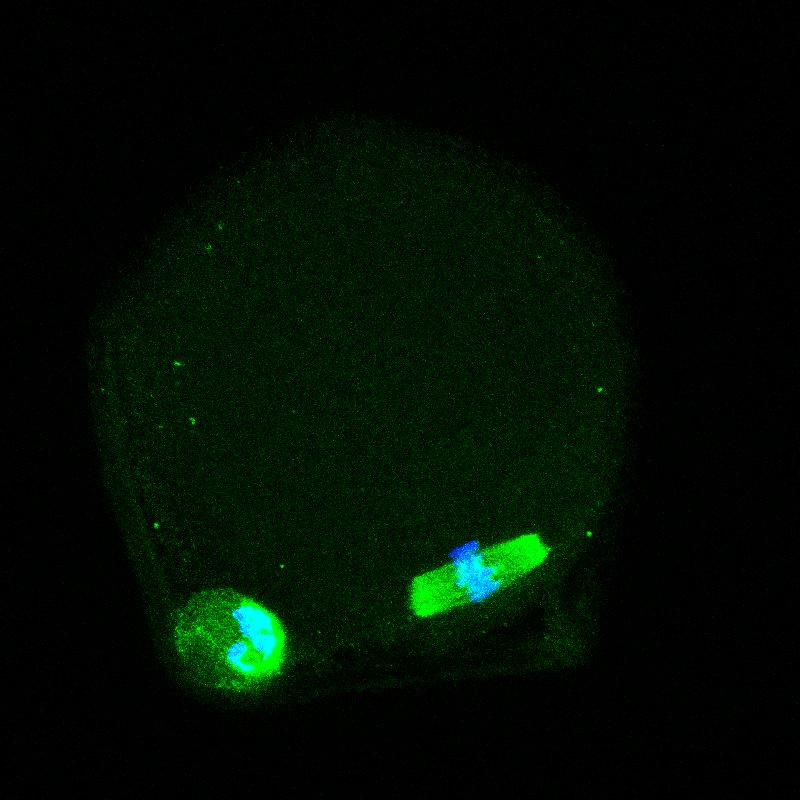

Supplement: Supplementary file 9 — Source data Fig. 5 [file 44318_2026_813_MOESM9_ESM.zip › Figure 5/5E/WT-actub, DNA.jpg]

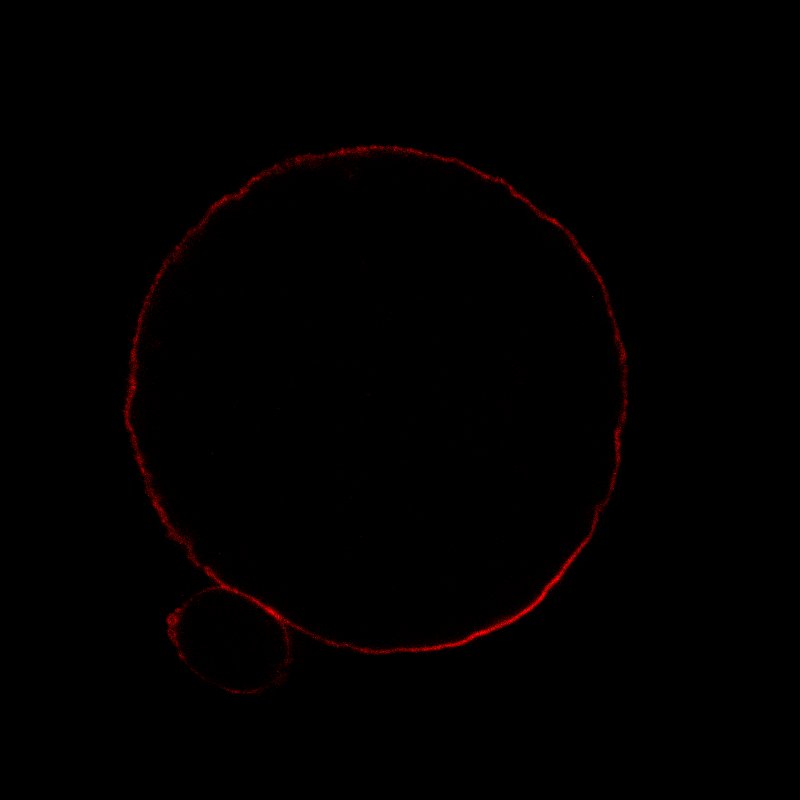

Supplement: Supplementary file 9 — Source data Fig. 5 [file 44318_2026_813_MOESM9_ESM.zip › Figure 5/5E/WT-phalloidin.jpg]

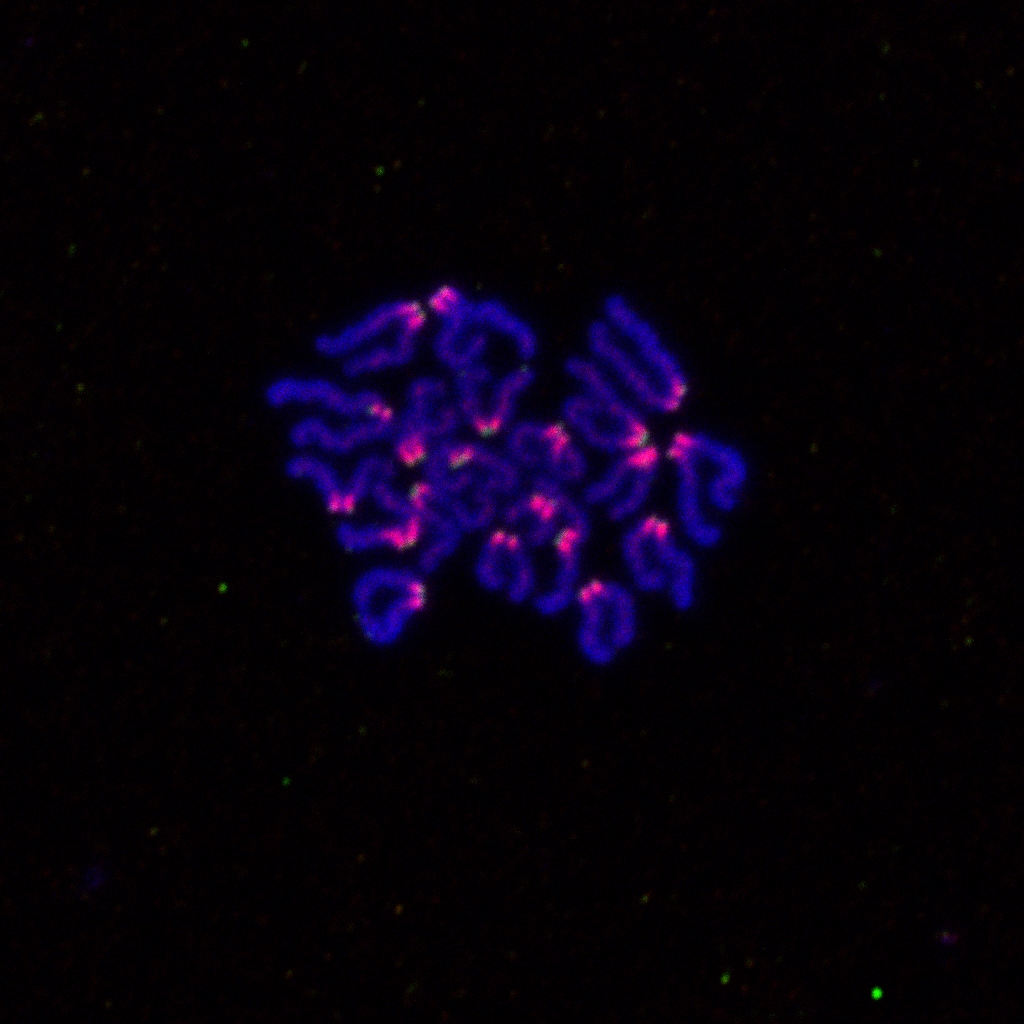

Supplement: Supplementary file 9 — Source data Fig. 5 [file 44318_2026_813_MOESM9_ESM.zip › Figure 5/5G/cKO-1.jpg]

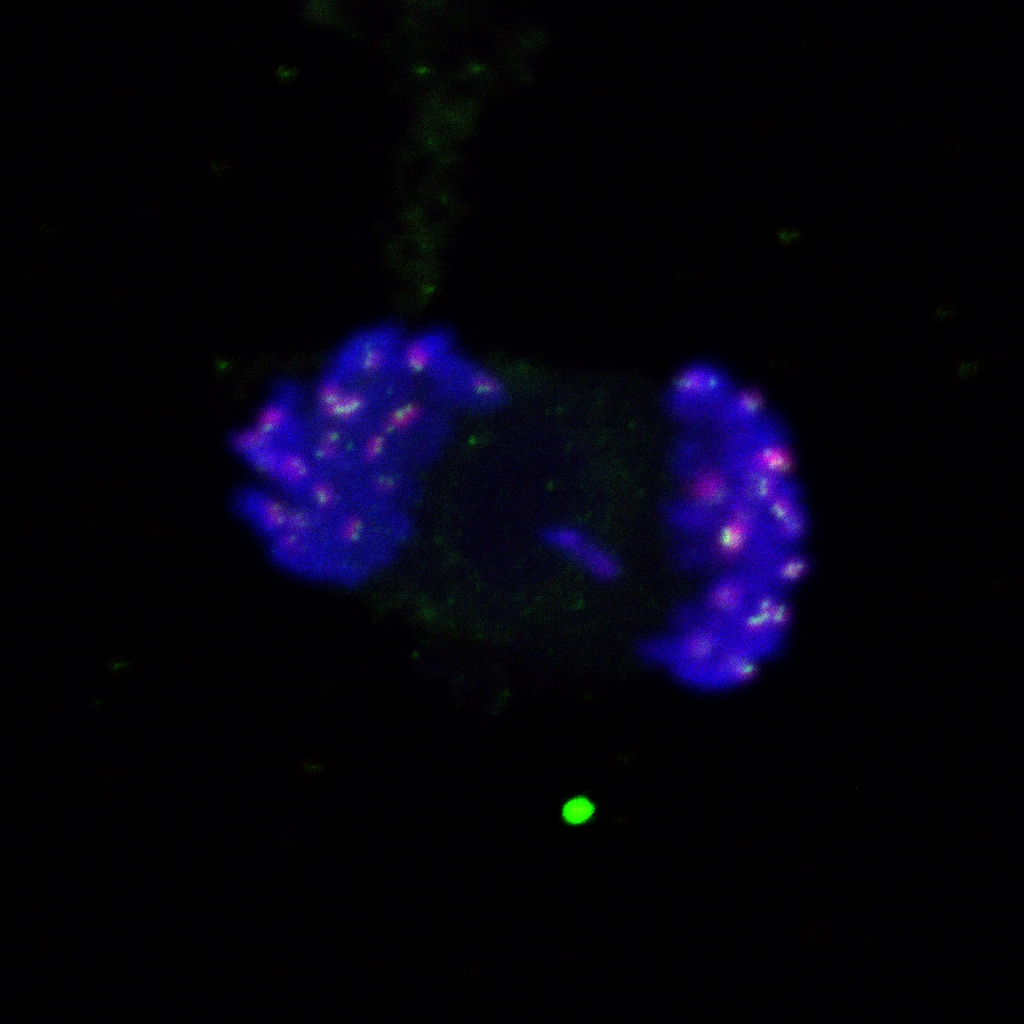

Supplement: Supplementary file 9 — Source data Fig. 5 [file 44318_2026_813_MOESM9_ESM.zip › Figure 5/5G/cKO-2.jpg]

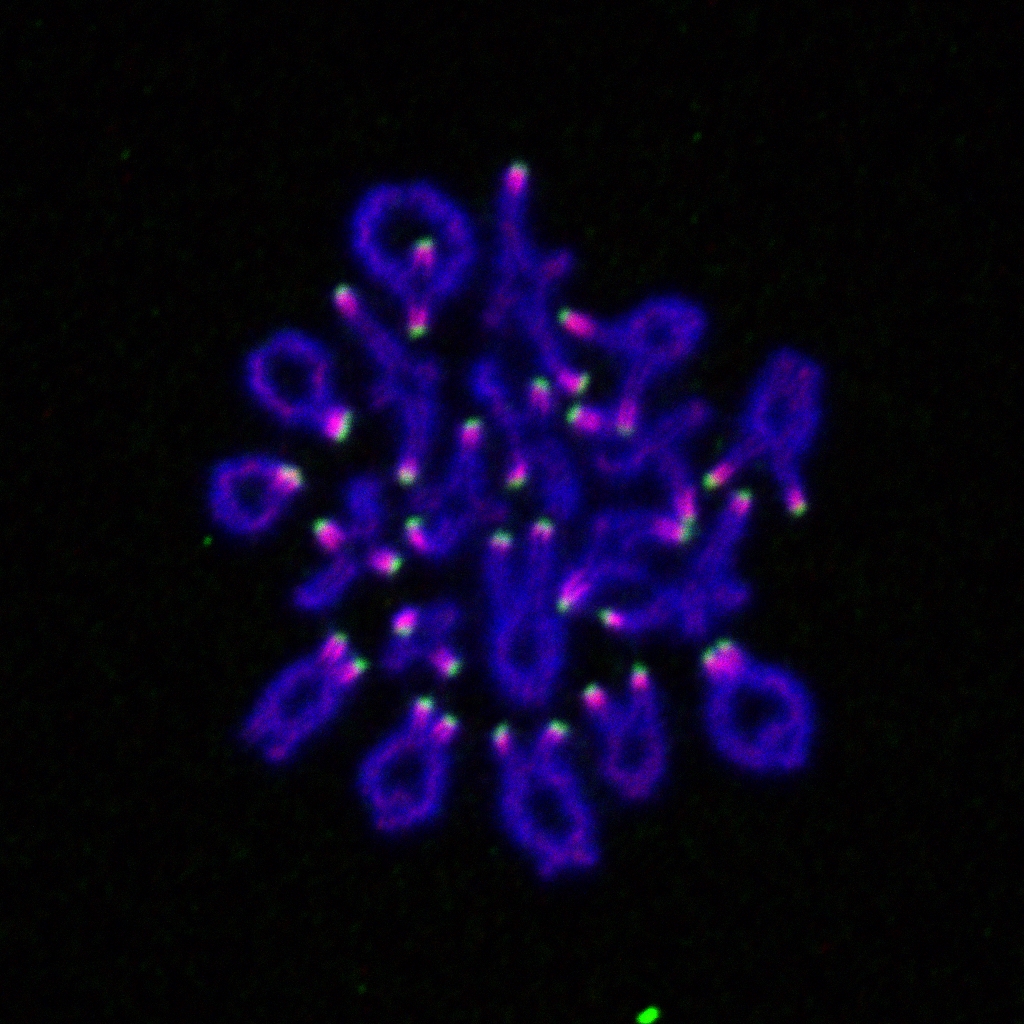

Supplement: Supplementary file 9 — Source data Fig. 5 [file 44318_2026_813_MOESM9_ESM.zip › Figure 5/5G/cKO-3.jpg]

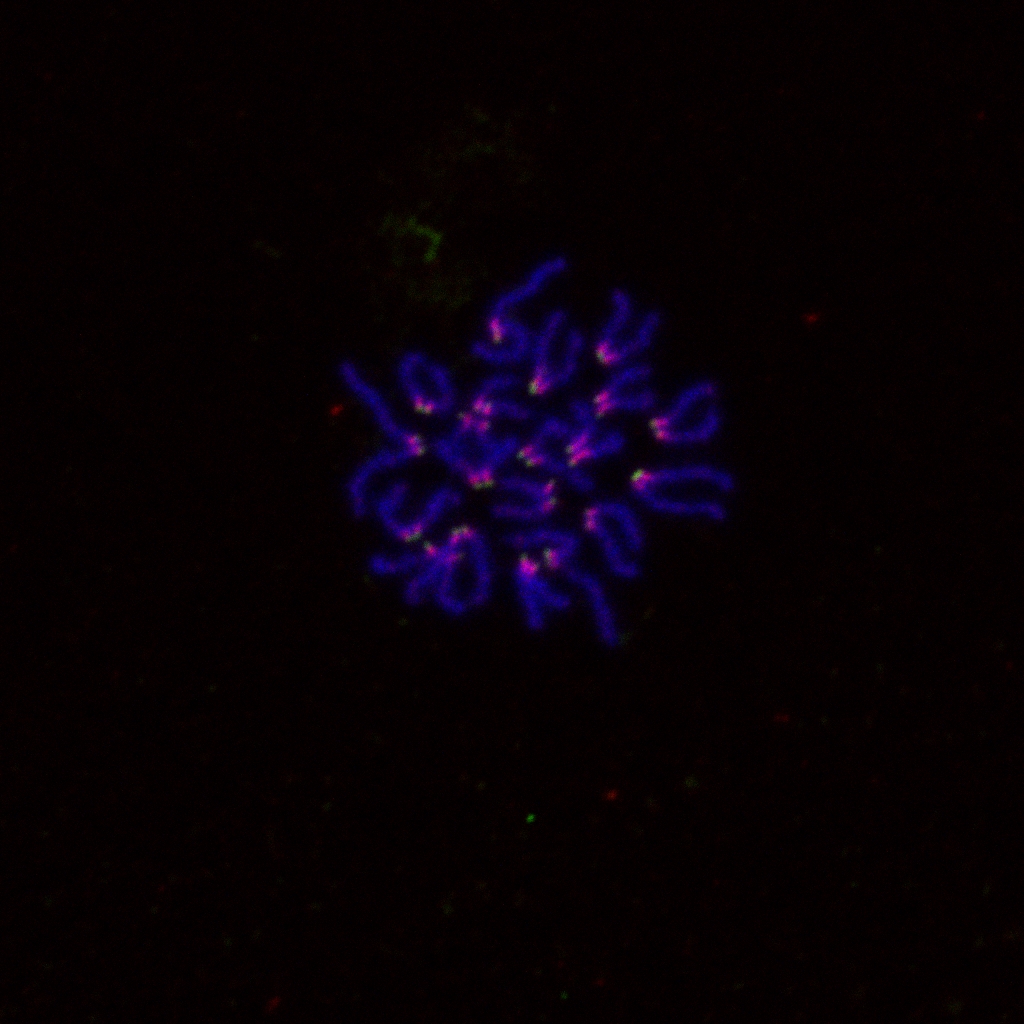

Supplement: Supplementary file 9 — Source data Fig. 5 [file 44318_2026_813_MOESM9_ESM.zip › Figure 5/5G/WT.jpg]

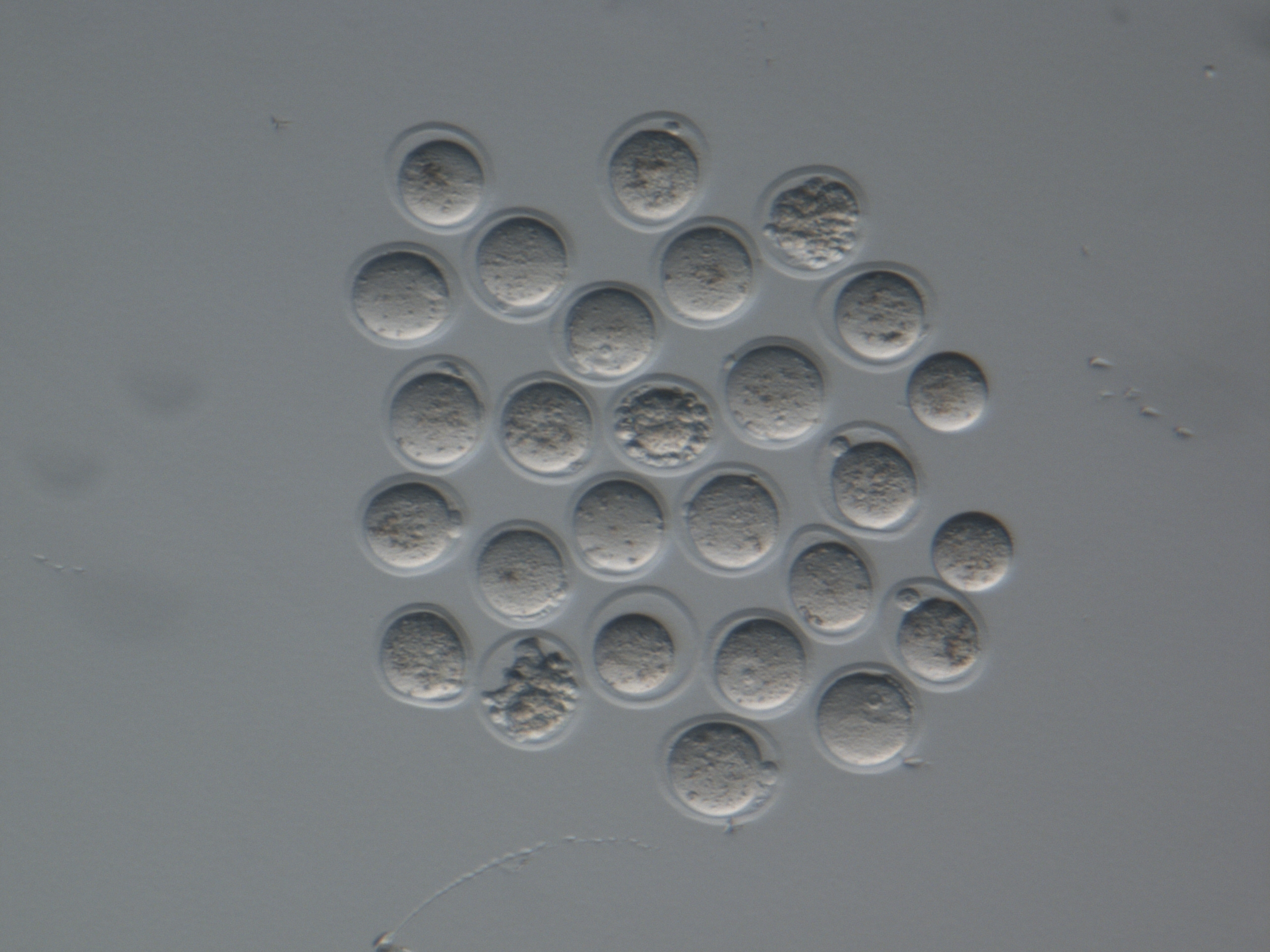

Supplement: Supplementary file 10 — Source data Fig. 6 [file 44318_2026_813_MOESM10_ESM.zip › Figure 6/6A/cKO.jpg]

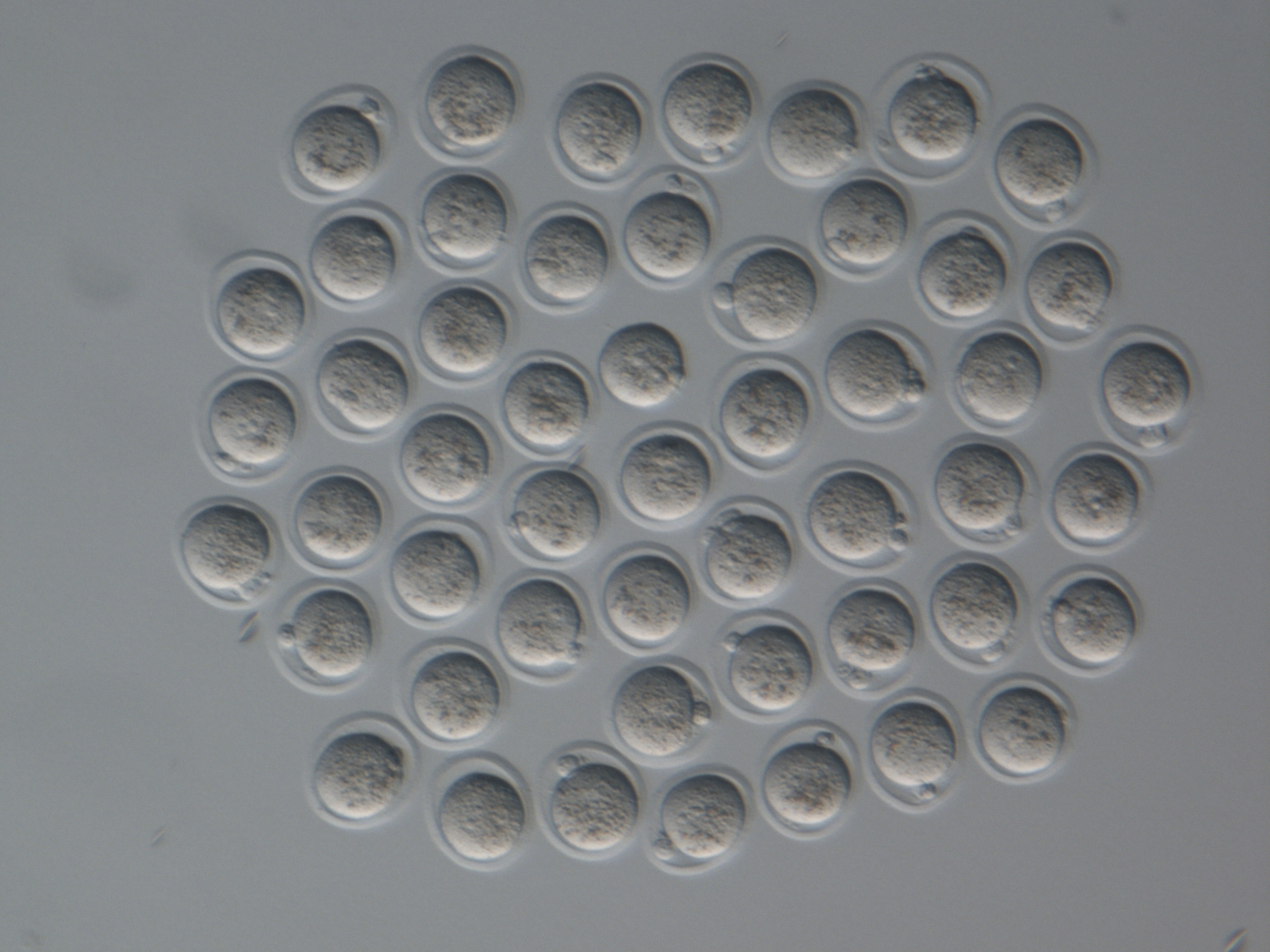

Supplement: Supplementary file 10 — Source data Fig. 6 [file 44318_2026_813_MOESM10_ESM.zip › Figure 6/6A/WT.jpg]

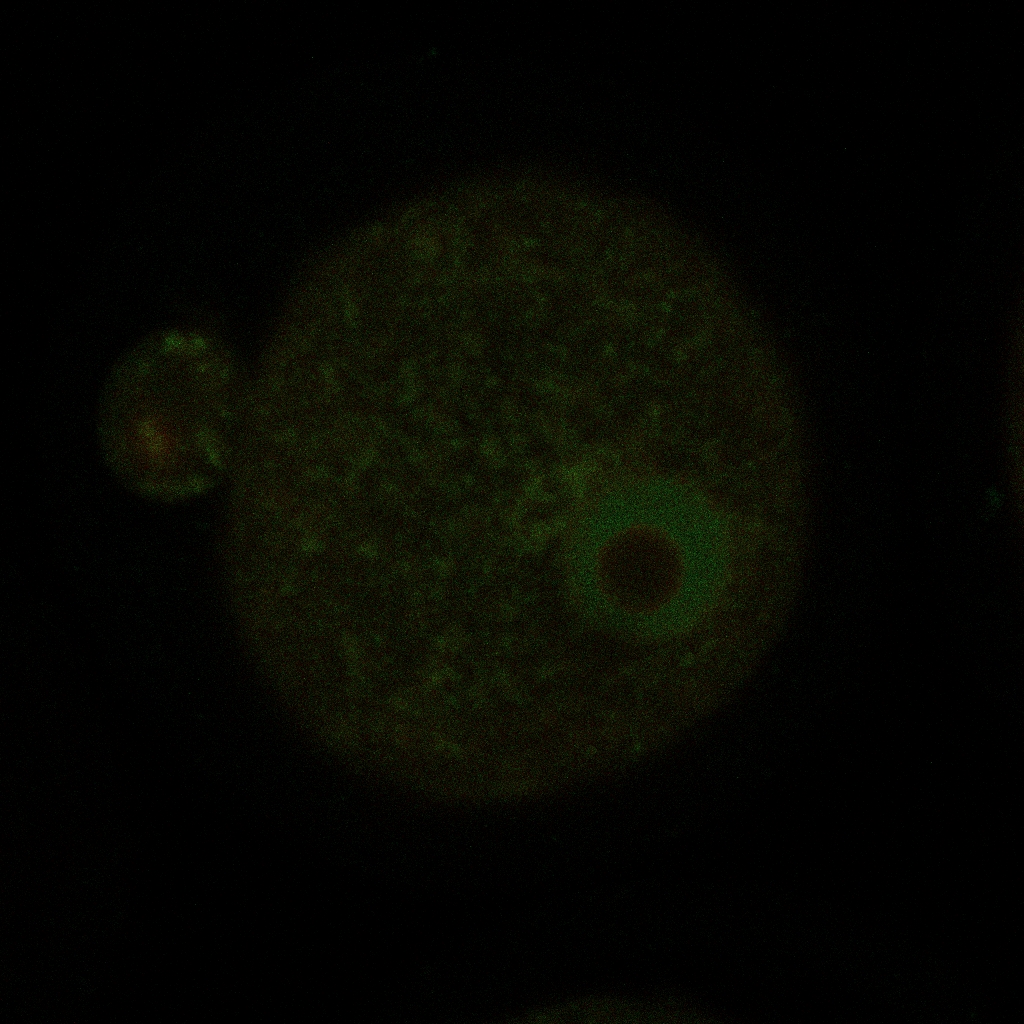

Supplement: Supplementary file 10 — Source data Fig. 6 [file 44318_2026_813_MOESM10_ESM.zip › Figure 6/6D/cKO.jpg]

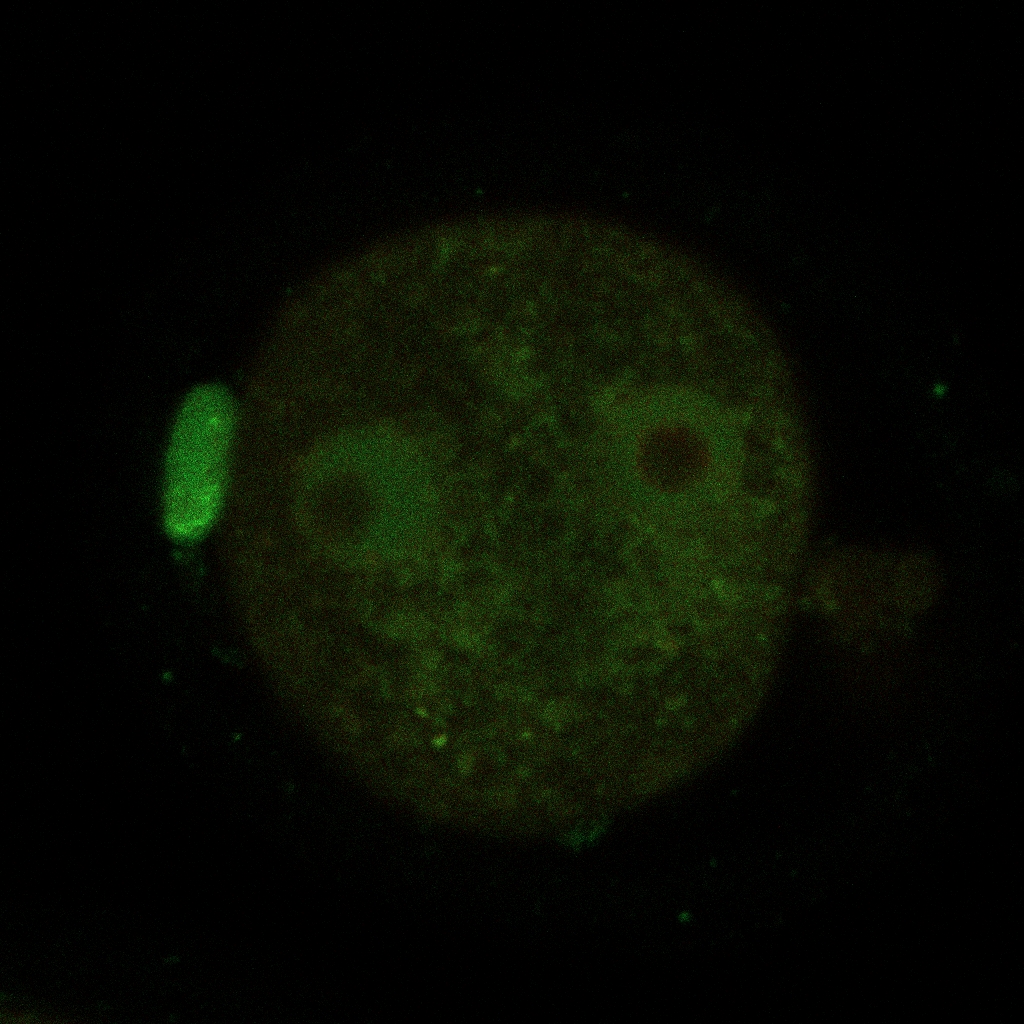

Supplement: Supplementary file 10 — Source data Fig. 6 [file 44318_2026_813_MOESM10_ESM.zip › Figure 6/6D/WT.jpg]

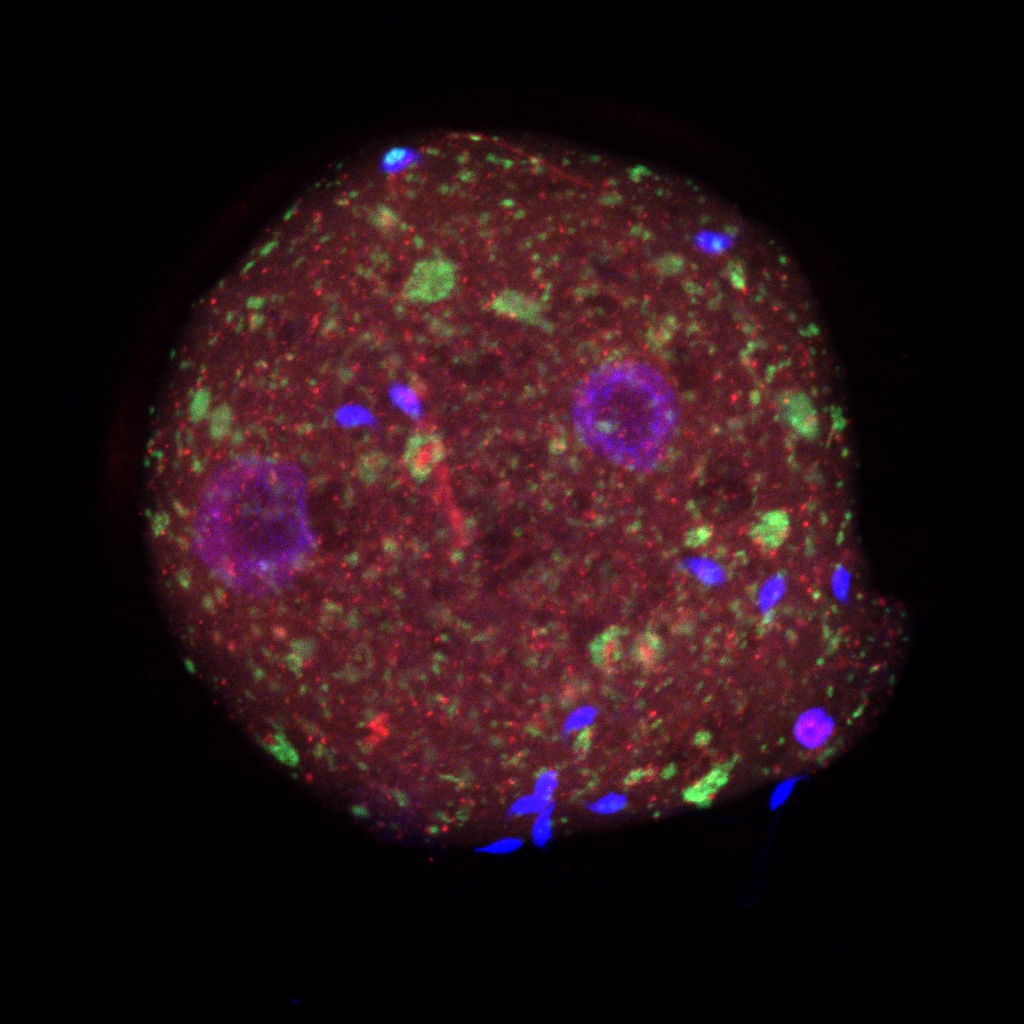

Supplement: Supplementary file 10 — Source data Fig. 6 [file 44318_2026_813_MOESM10_ESM.zip › Figure 6/6F/cKO.jpg]

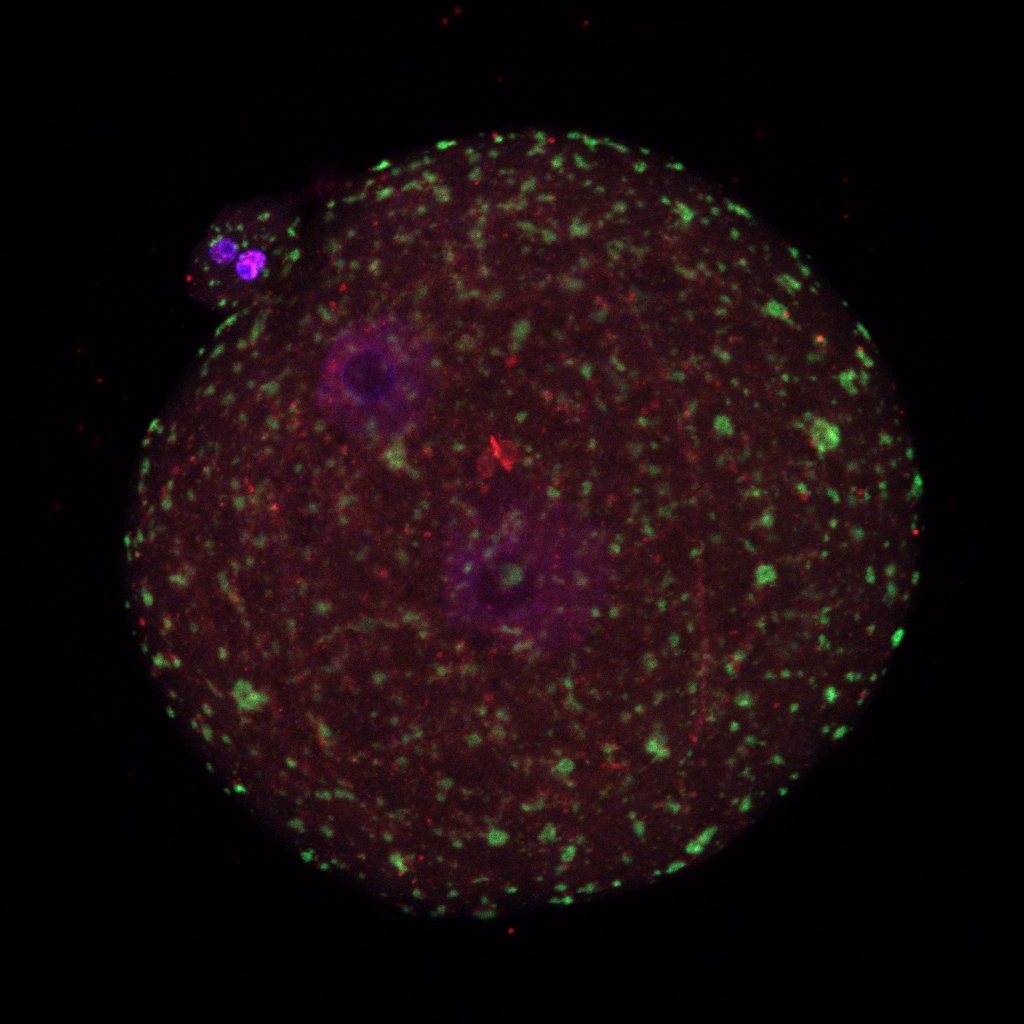

Supplement: Supplementary file 10 — Source data Fig. 6 [file 44318_2026_813_MOESM10_ESM.zip › Figure 6/6F/WT.jpg]

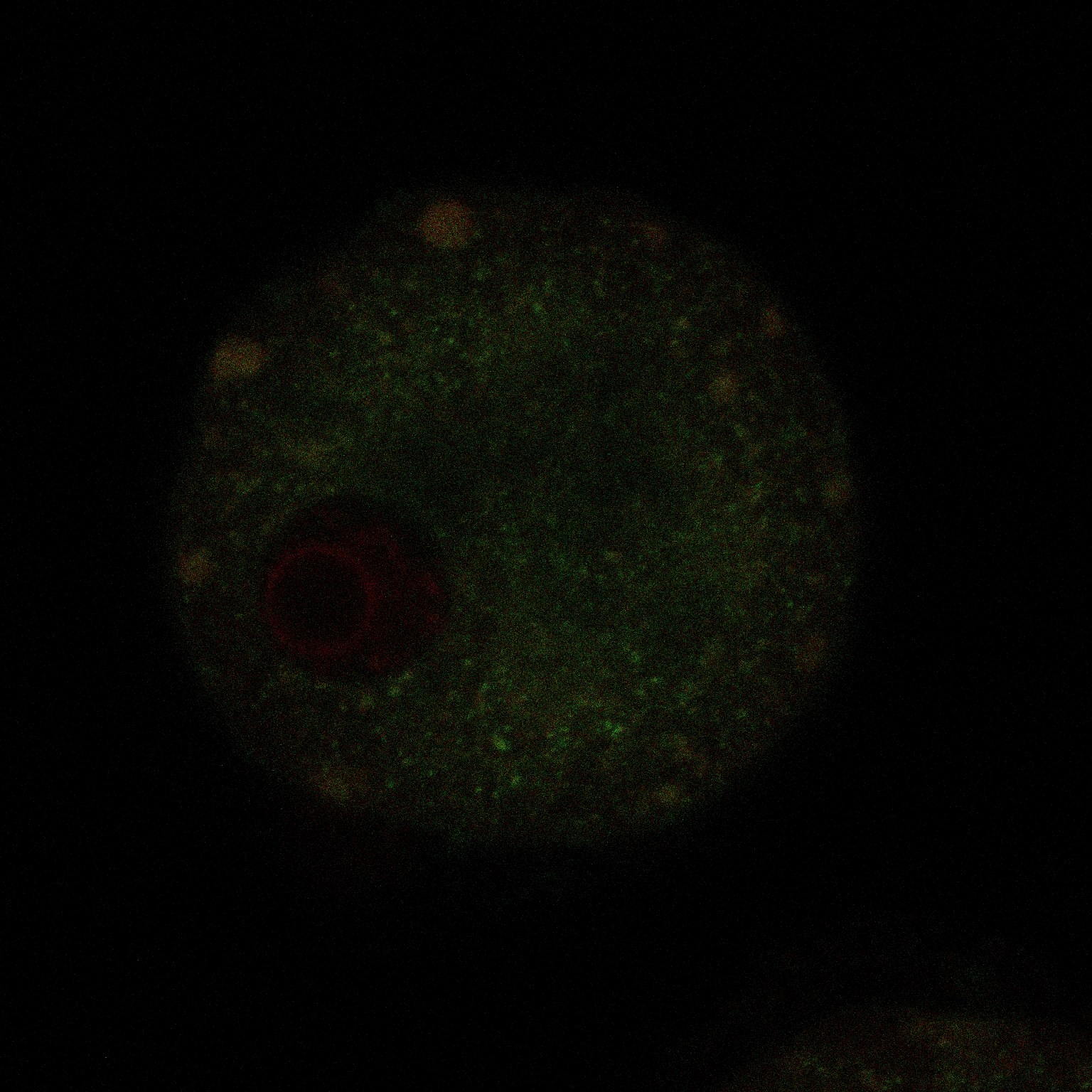

Supplement: Supplementary file 10 — Source data Fig. 6 [file 44318_2026_813_MOESM10_ESM.zip › Figure 6/6I/cKO.jpg]

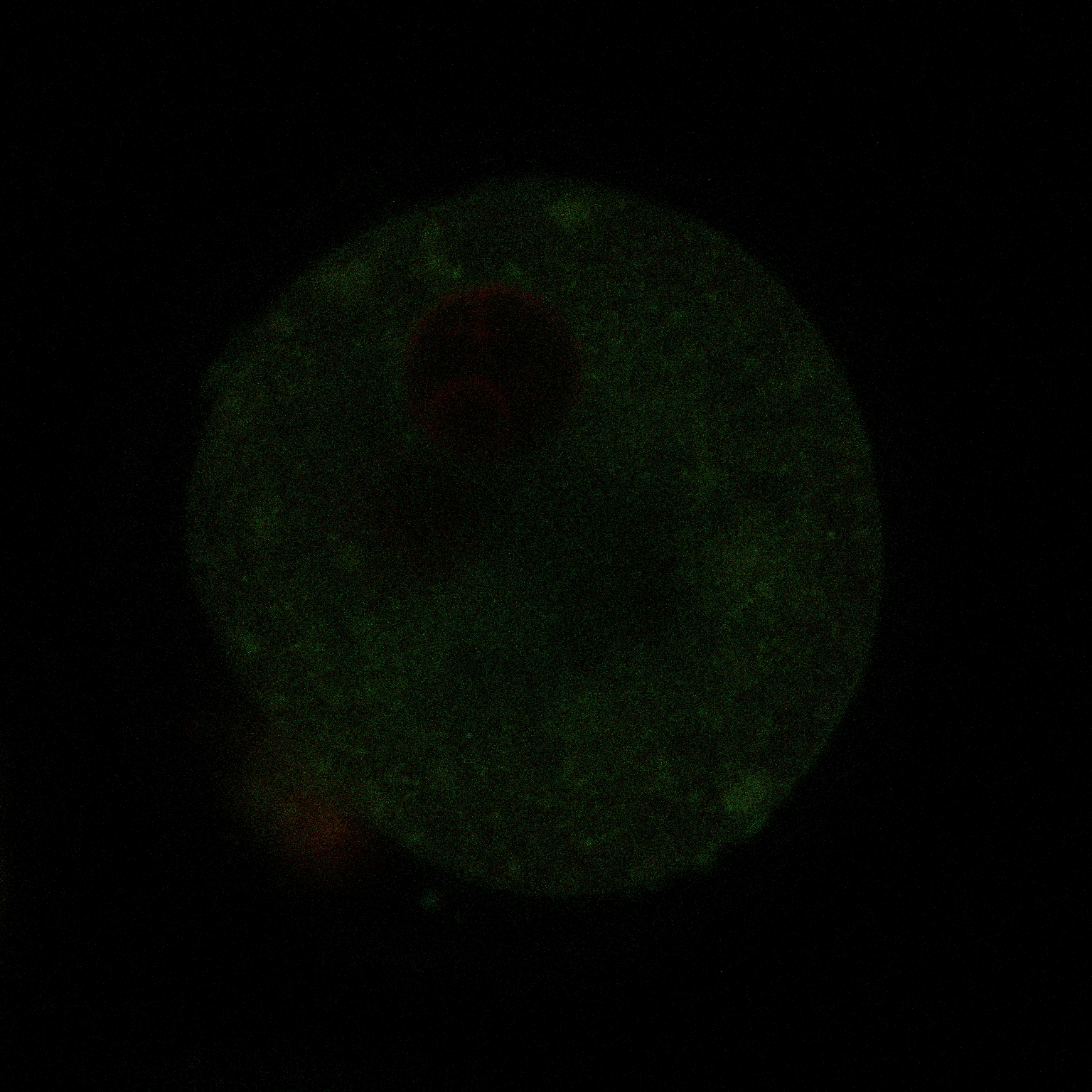

Supplement: Supplementary file 10 — Source data Fig. 6 [file 44318_2026_813_MOESM10_ESM.zip › Figure 6/6I/WT.jpg]
