# Supplementary material for: 20S proteasome-regulated proteostasis in ELVAs is critical for oocyte-to-embryo transition and female fertility
Source: EMBO J. 2026 May 21;45(14):4887–909. doi: 10.1038/s44318-026-00813-0 (PMC13373198; doi:10.1038/s44318-026-00813-0)
Supplement: Supplementary file 6 — Source data Fig. 2 [file 44318_2026_813_MOESM6_ESM.zip › Figure 2/2B/2B.pptx]

## Slide 1
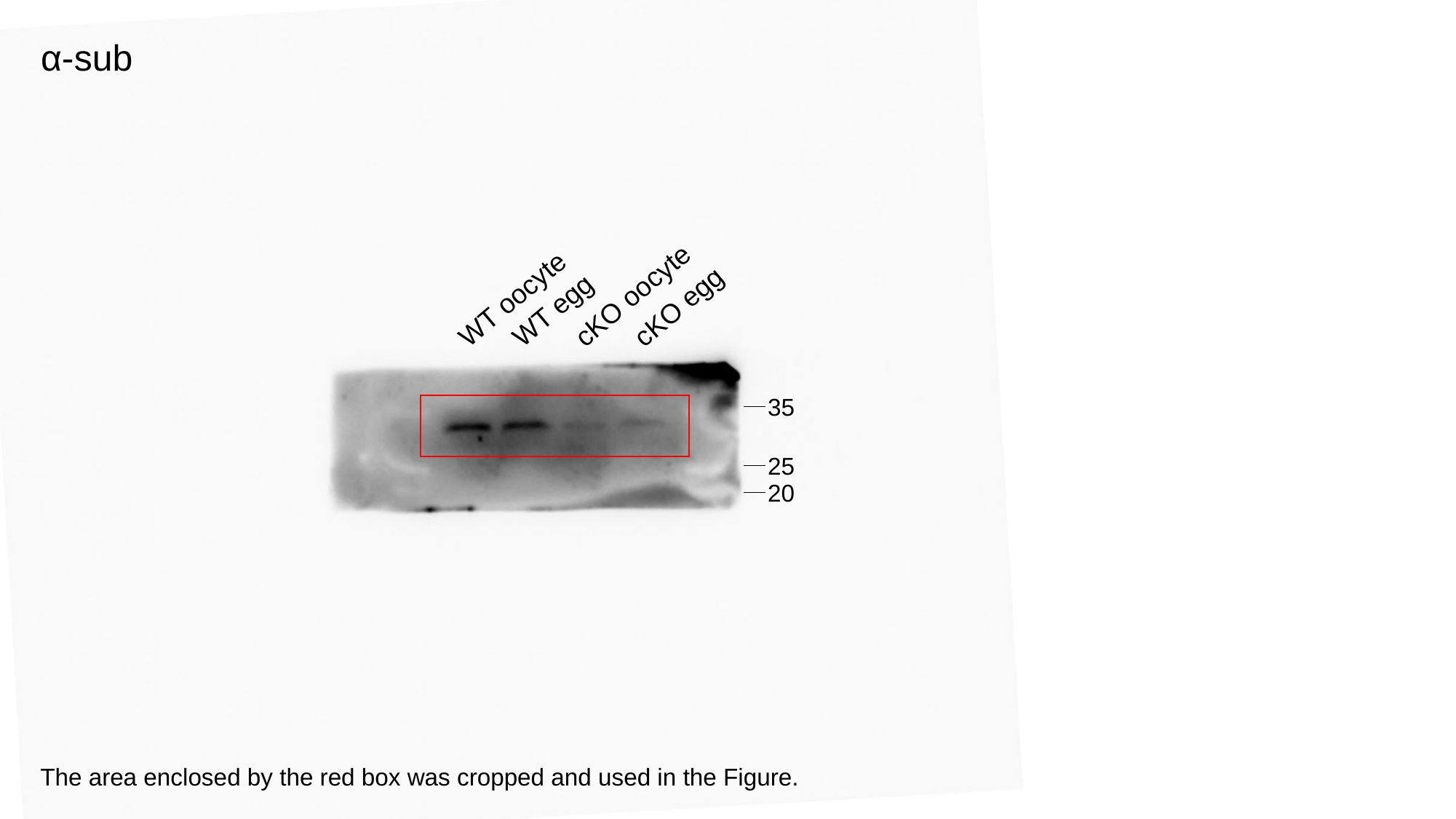

α-sub
cKO oocyte
WT oocyte
cKO egg
WT egg
35
25
20
The area enclosed by the red box was cropped and used in the Figure.

## Slide 2
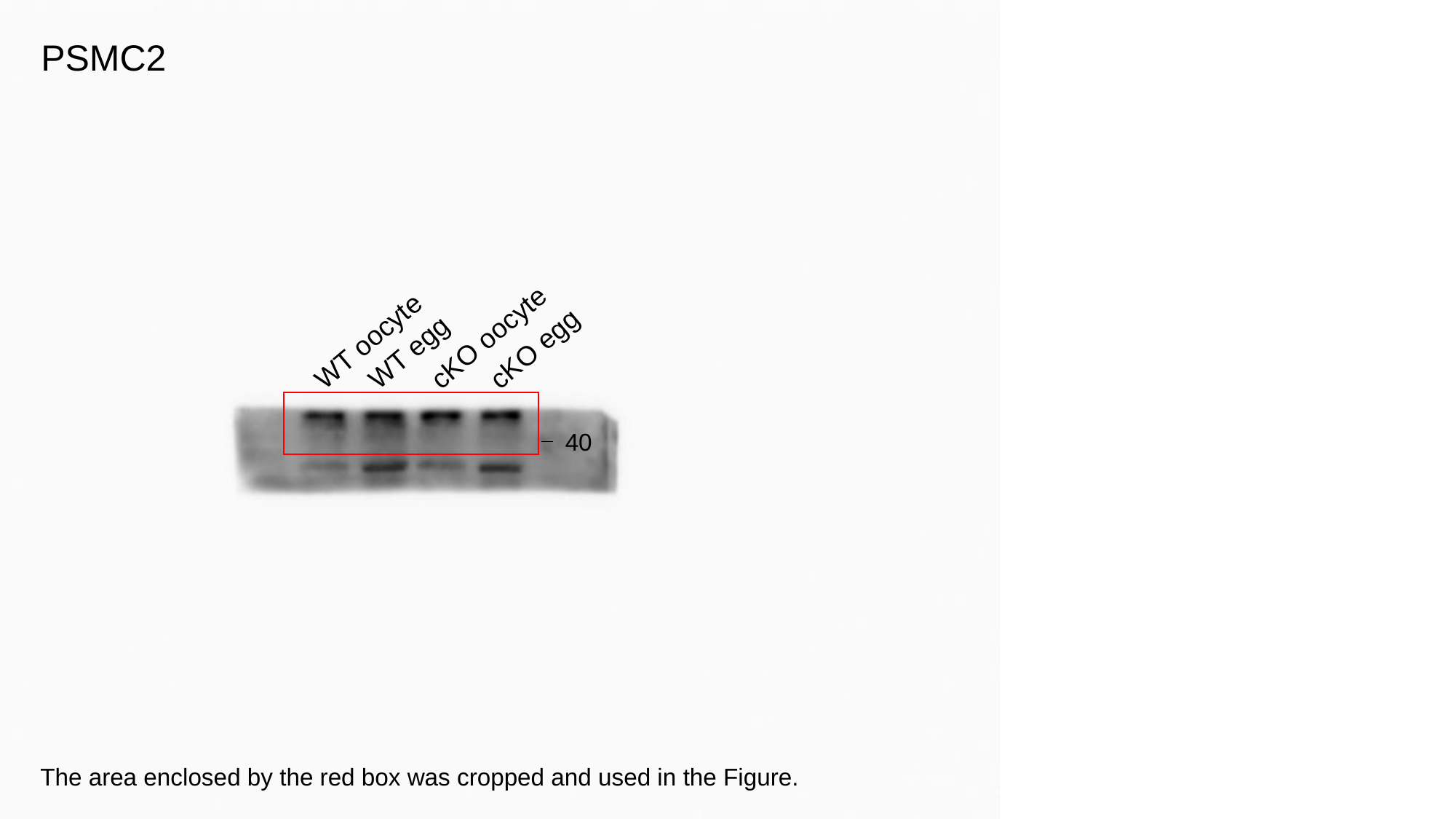

PSMC2
cKO oocyte
WT oocyte
cKO egg
WT egg
40
The area enclosed by the red box was cropped and used in the Figure.

## Slide 3
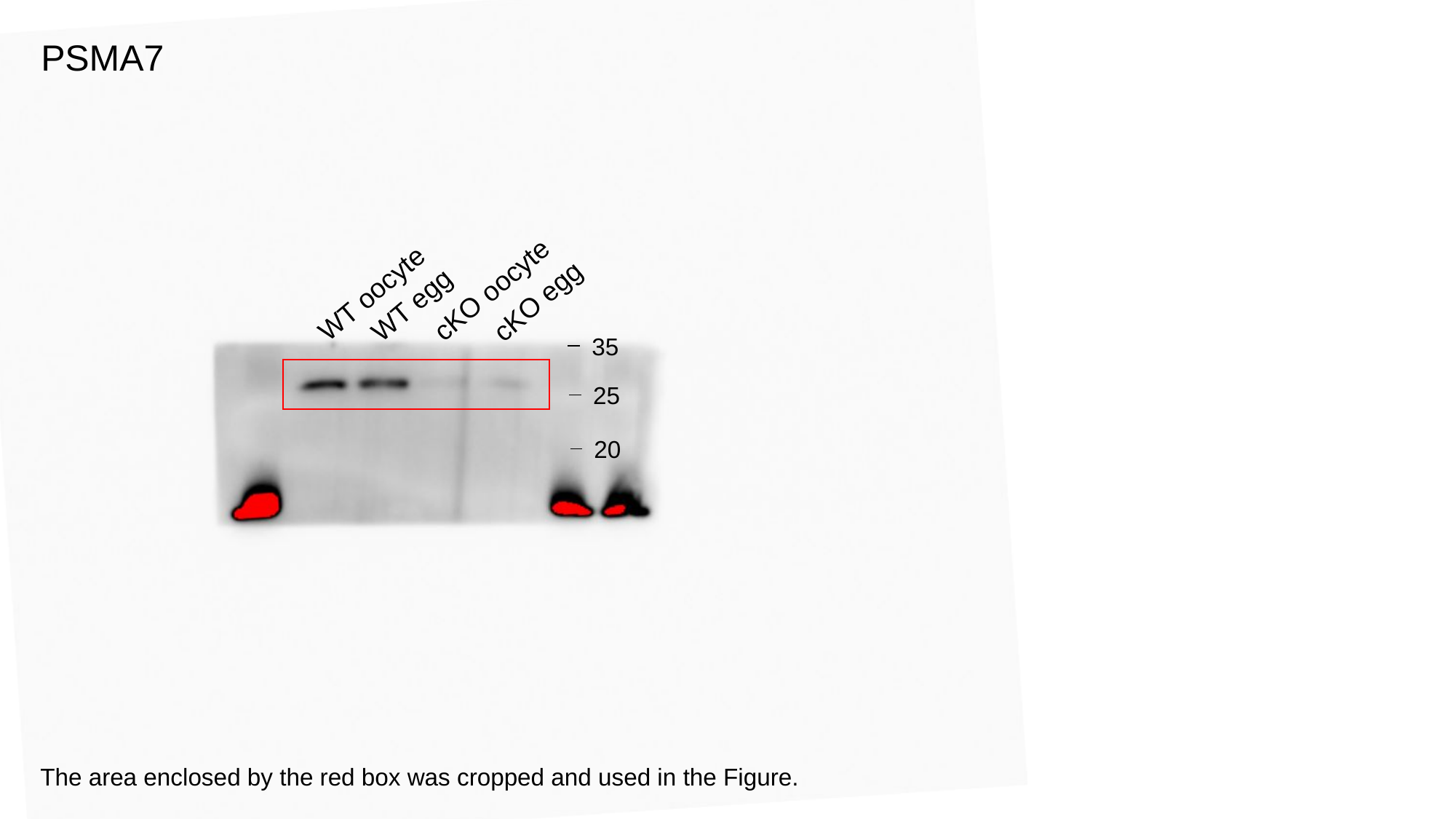

PSMA7
cKO oocyte
WT oocyte
cKO egg
WT egg
35
25
20
The area enclosed by the red box was cropped and used in the Figure.

## Slide 4
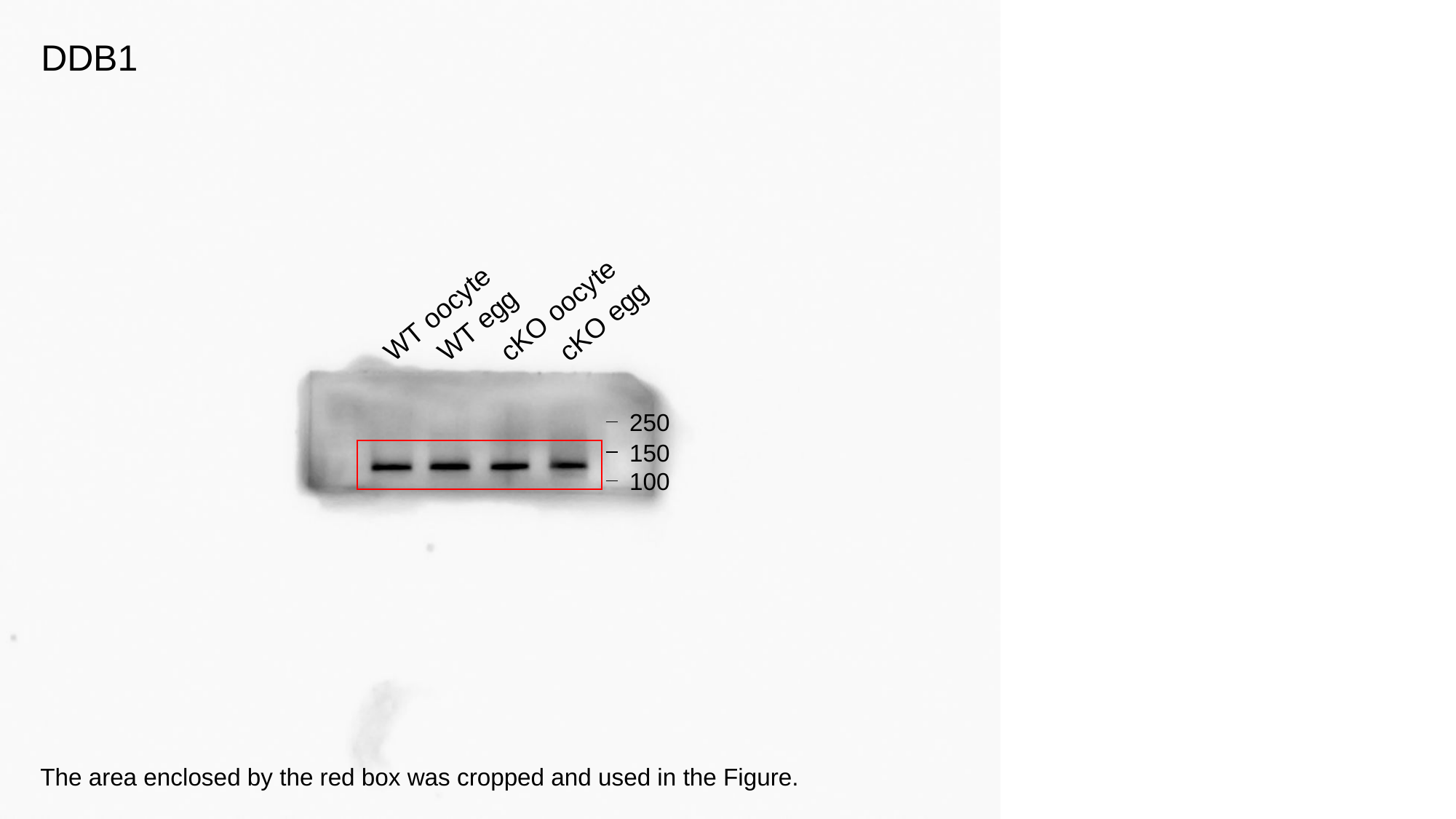

DDB1
cKO oocyte
WT oocyte
cKO egg
WT egg
250
150
100
The area enclosed by the red box was cropped and used in the Figure.
