# Supplementary material for: 20S proteasome-regulated proteostasis in ELVAs is critical for oocyte-to-embryo transition and female fertility
Source: EMBO J. 2026 May 21;45(14):4887–909. doi: 10.1038/s44318-026-00813-0 (PMC13373198; doi:10.1038/s44318-026-00813-0)
Supplement: Supplementary file 7 — Source data Fig. 3 [file 44318_2026_813_MOESM7_ESM.zip › Figure 3/3J/3J.pptx]

## Slide 1
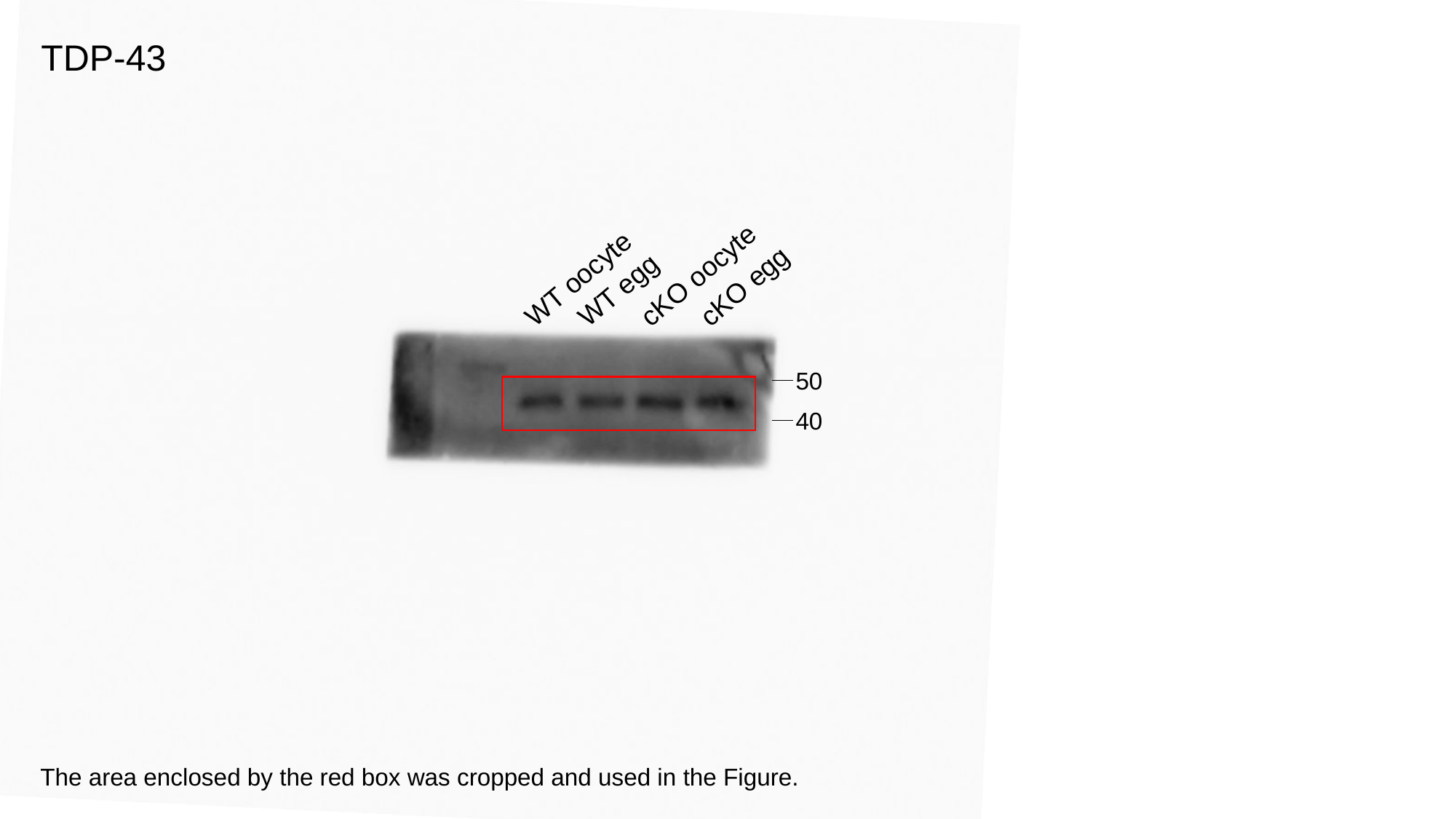

TDP-43
cKO oocyte
WT oocyte
cKO egg
WT egg
50
40
The area enclosed by the red box was cropped and used in the Figure.

## Slide 2
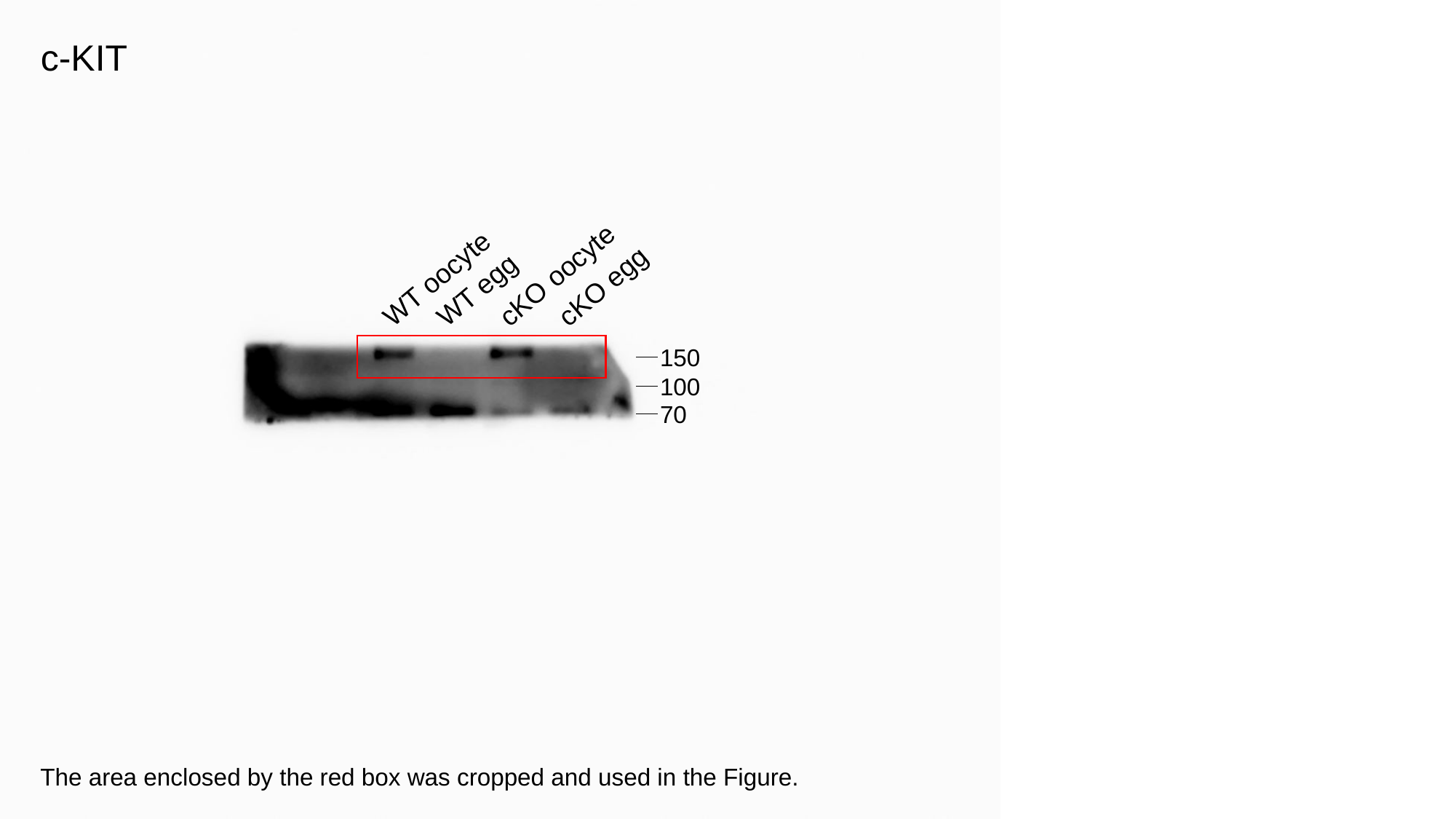

c-KIT
cKO oocyte
WT oocyte
cKO egg
WT egg
150
100
70
The area enclosed by the red box was cropped and used in the Figure.

## Slide 3
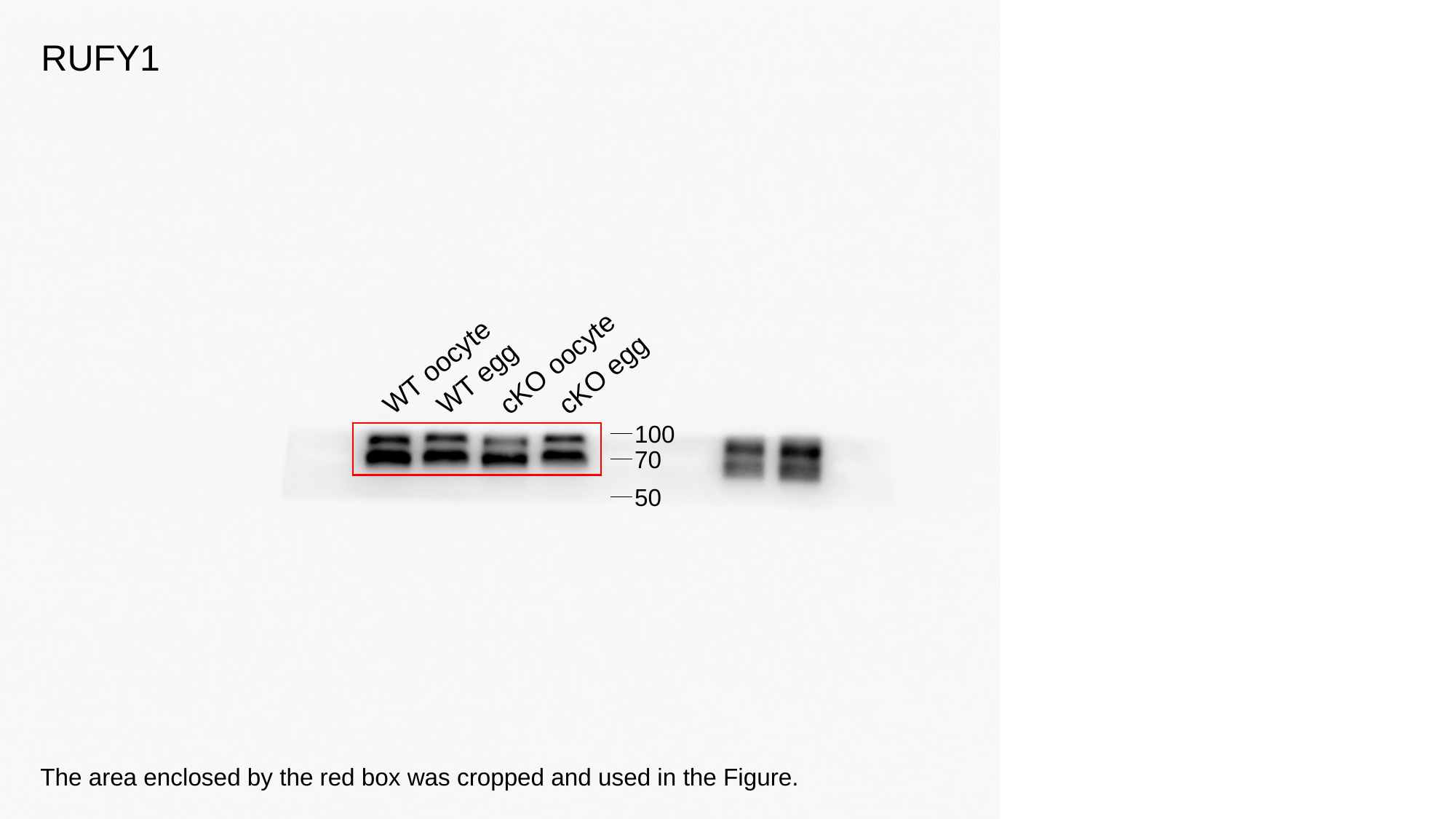

RUFY1
cKO oocyte
WT oocyte
cKO egg
WT egg
100
70
50
The area enclosed by the red box was cropped and used in the Figure.

## Slide 4
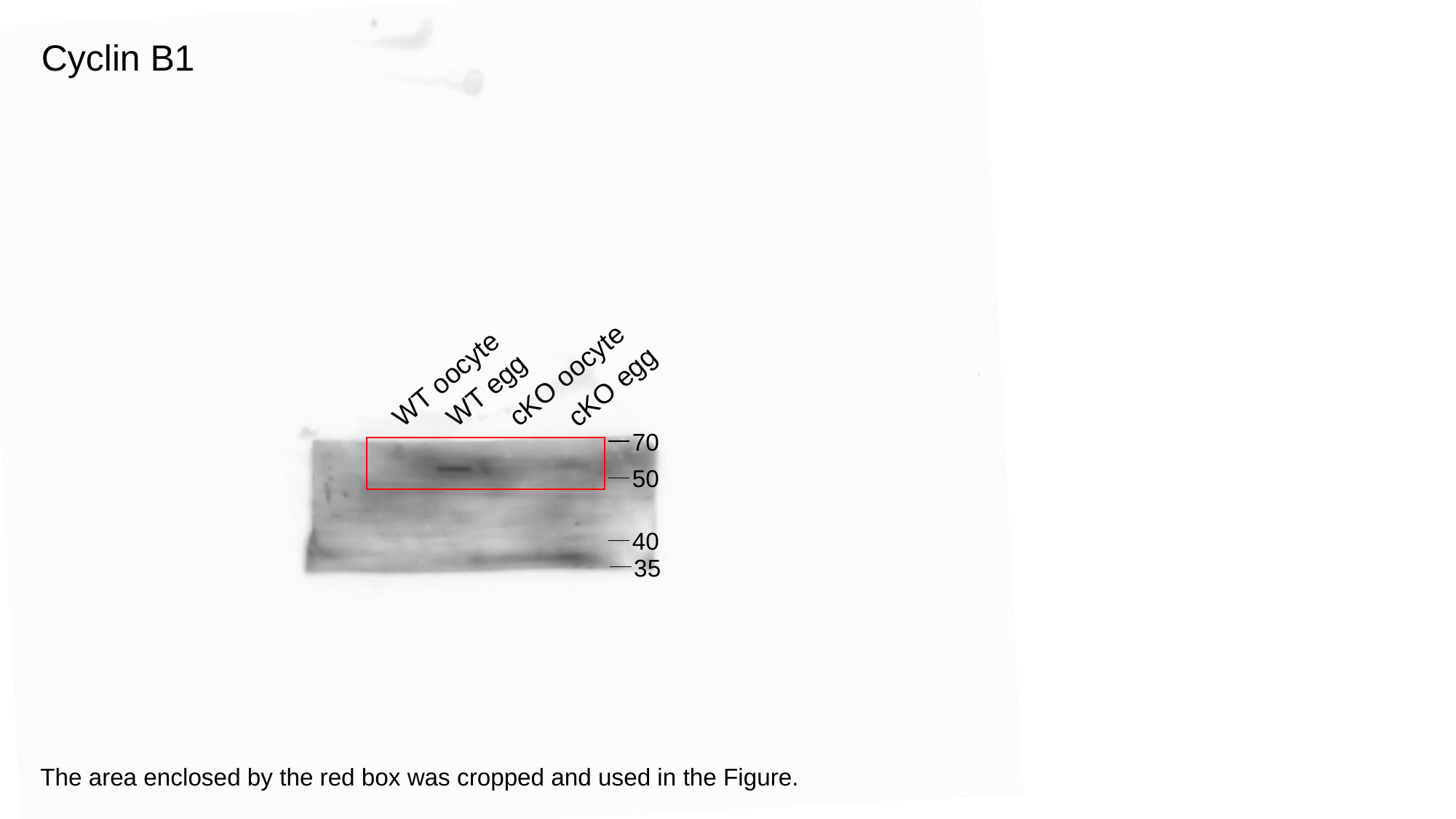

Cyclin B1
cKO oocyte
WT oocyte
cKO egg
WT egg
70
50
40
35
The area enclosed by the red box was cropped and used in the Figure.

## Slide 5
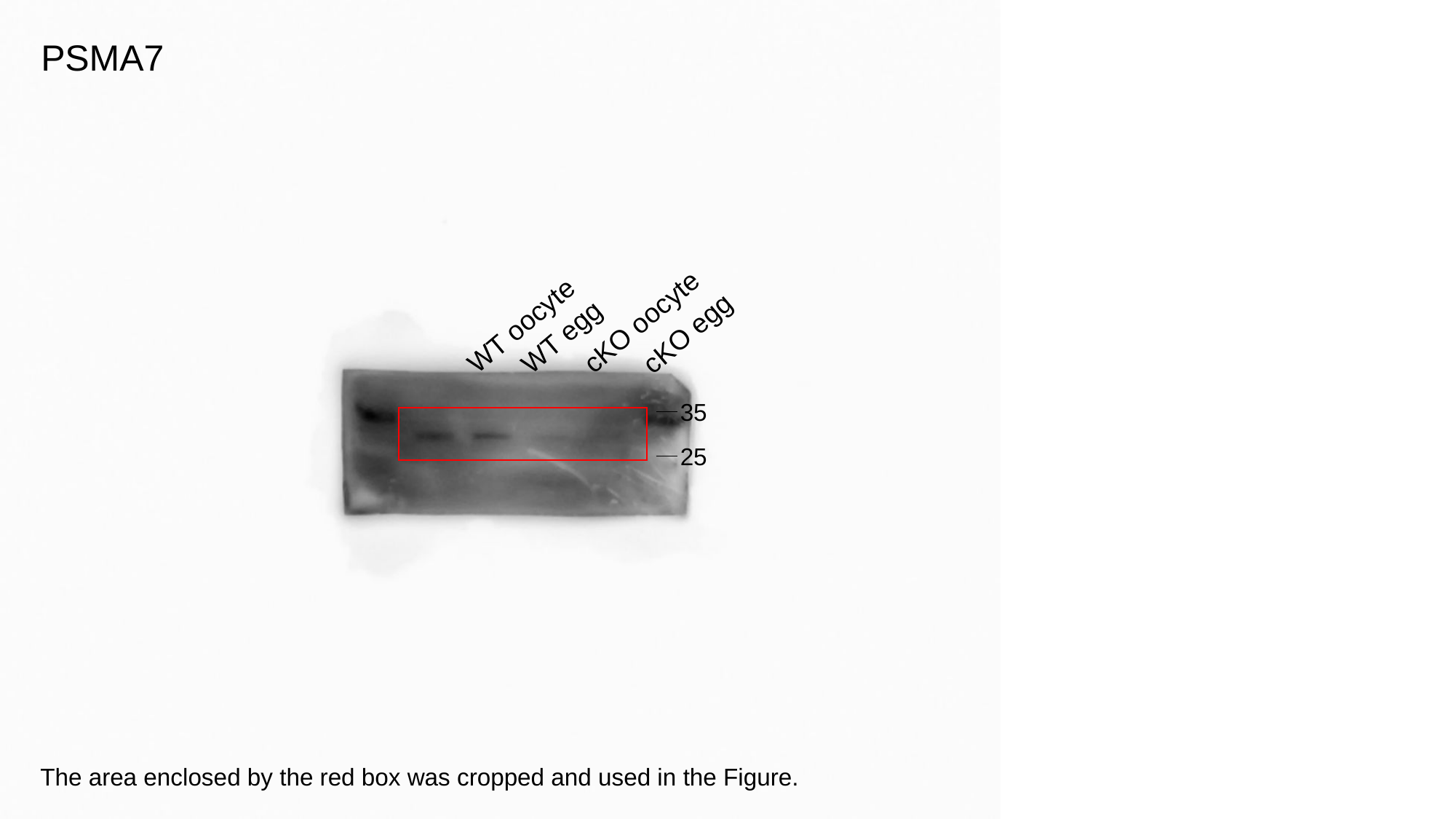

PSMA7
cKO oocyte
WT oocyte
cKO egg
WT egg
35
25
The area enclosed by the red box was cropped and used in the Figure.

## Slide 6
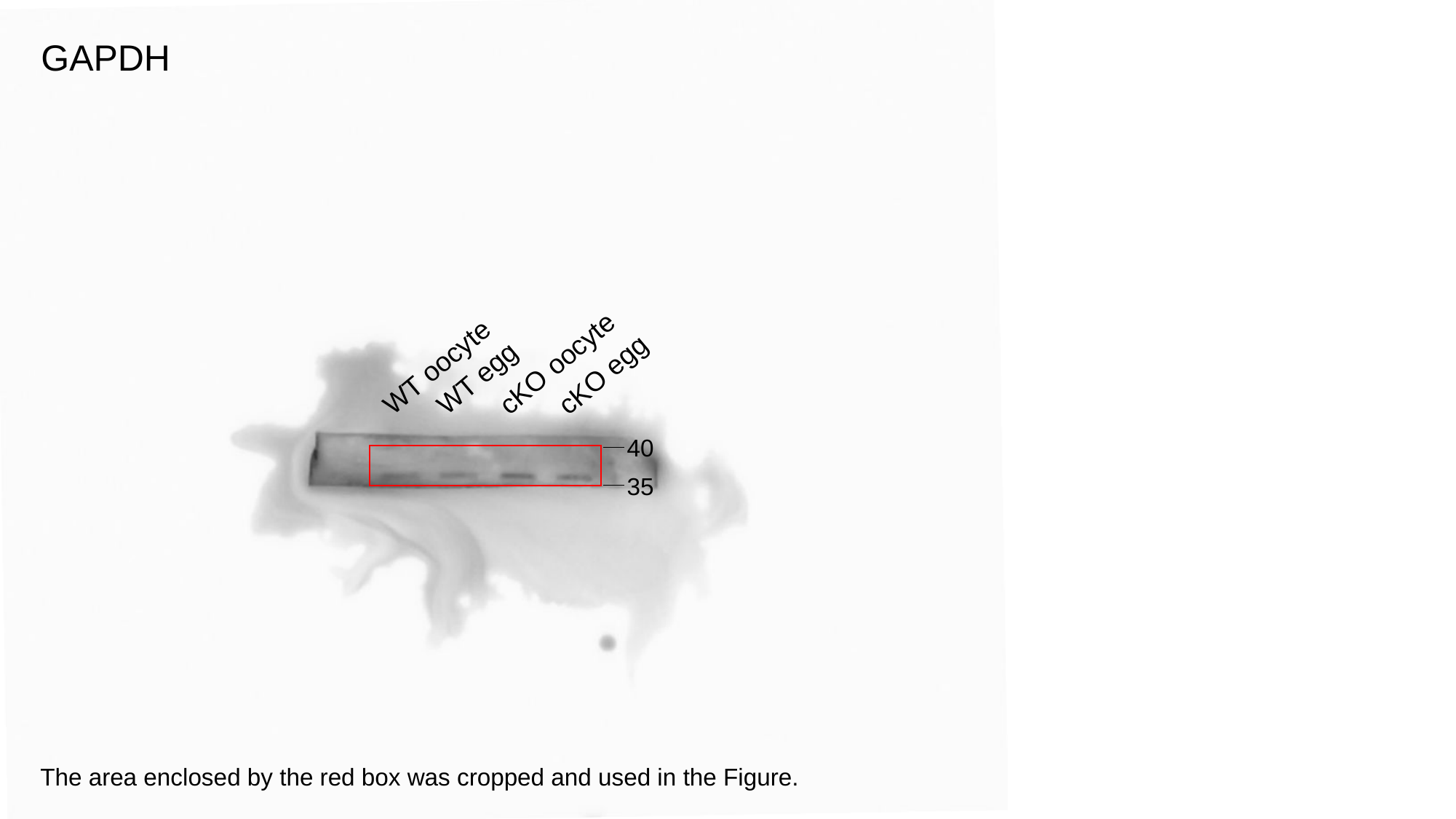

GAPDH
cKO oocyte
WT oocyte
cKO egg
WT egg
40
35
The area enclosed by the red box was cropped and used in the Figure.
