# Supplementary material for: 20S proteasome-regulated proteostasis in ELVAs is critical for oocyte-to-embryo transition and female fertility
Source: EMBO J. 2026 May 21;45(14):4887–909. doi: 10.1038/s44318-026-00813-0 (PMC13373198; doi:10.1038/s44318-026-00813-0)
Supplement: Supplementary file 11 — Expanded View Figures [file 44318_2026_813_MOESM11_ESM.pdf]

Expanded View Figures

Figure EV1. Histological analyses of ovaries and oocytes derived from WT and *Psma7* cKO females.

(**A**, **B**) Immunofluorescent staining of PSMA7 (**A**) and PSMC2 (**B**) in mouse oocytes and early embryos. Scale bar, 20 µm. (**C**) Western blot analysis of PSMA7, PSMC2 and α-subunits levels during mouse oocyte maturation. DDB1 serves as the loading control. Total protein lysates from 50 oocytes were loaded in each lane. Quantification of the band intensities is shown below the blot. (**D**) The line chart illustrating the expression levels of proteasome subunits in mouse and human oocytes. Data were reanalyzed from Wu et al, 2022. (**E**) H&E staining of WT and *Psma7* cKO ovaries at PD21 (Postnatal Day 21). Scale bar, 100 µm. (**F**, **G**) IHC analysis of MVH (**F**) and PSMA7 (**G**) levels in WT and *Psma7* cKO ovaries at PD21. The black and blue arrows denote primordial and primary follicles, respectively. Scale bar, 100 µm. (**H**, **I**) Representative images of GV oocytes (**H**) and quantification of their number (**I**) collected from WT and *Psma7* cKO ovaries. BF brightfield. Scale bar, 100 µm. *n* indicates the number of female mice analyzed. Mean and SD are shown. *P* values: unpaired *t* test.

 © The Author(s)

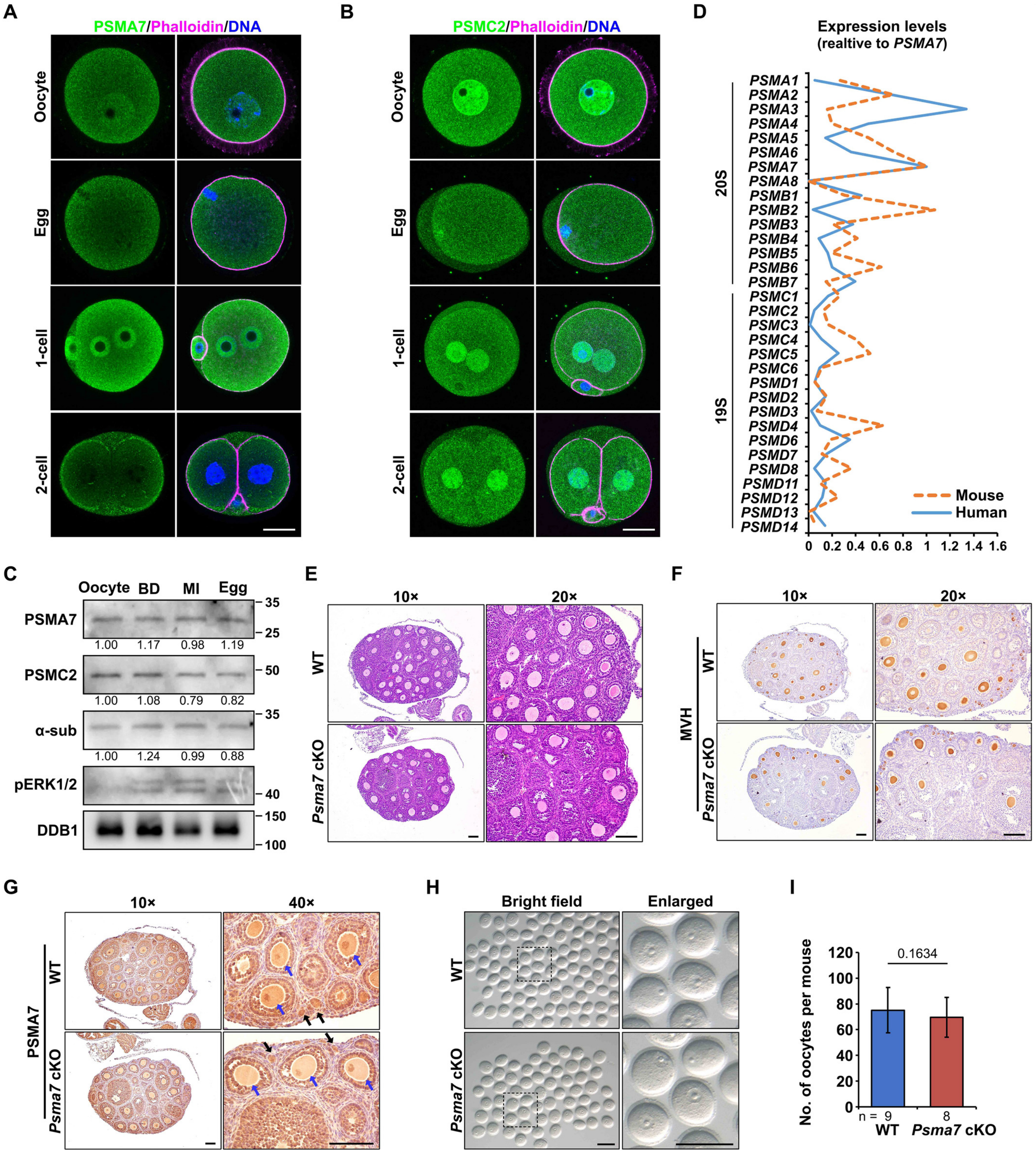
A
PSMA7/Phalloidin/DNA
Oocyte
Egg
1-cell
2-cell
B
PSMC2/Phalloidin/DNA
Oocyte
Egg
1-cell
2-cell
C
Oocyte BD MI Egg
PSMA7
35
25
1.00 1.17 0.98 1.19
PSMC2
50
1.00 1.08 0.79 0.82
α-sub
35
1.00 1.24 0.99 0.88
pERK1/2
40
DDB1
150
100
D
Expression levels
(realtive to PSMA7)
20S
19S
PSMA1
PSMA2
PSMA3
PSMA4
PSMA5
PSMA6
PSMA7
PSMA8
PSMB1
PSMB2
PSMB3
PSMB4
PSMB5
PSMB6
PSMB7
PSMC1
PSMC2
PSMC3
PSMC4
PSMC5
PSMC6
PSMD1
PSMD2
PSMD3
PSMD4
PSMD6
PSMD7
PSMD8
PSMD11
PSMD12
PSMD13
PSMD14
Mouse
Human
0 0.2 0.4 0.6 0.8 1 1.2 1.4 1.6
E
10×
20×
WT
Psma7 cKO
F
10×
20×
MVH
WT
Psma7 cKO
G
10×
40×
PSMA7
WT
Psma7 cKO
H
Bright field
Enlarged
WT
Psma7 cKO
I
No. of oocytes per mouse
120
100
80
60
40
20
0
0.1634
n = 9
8
WT
Psma7 cKO

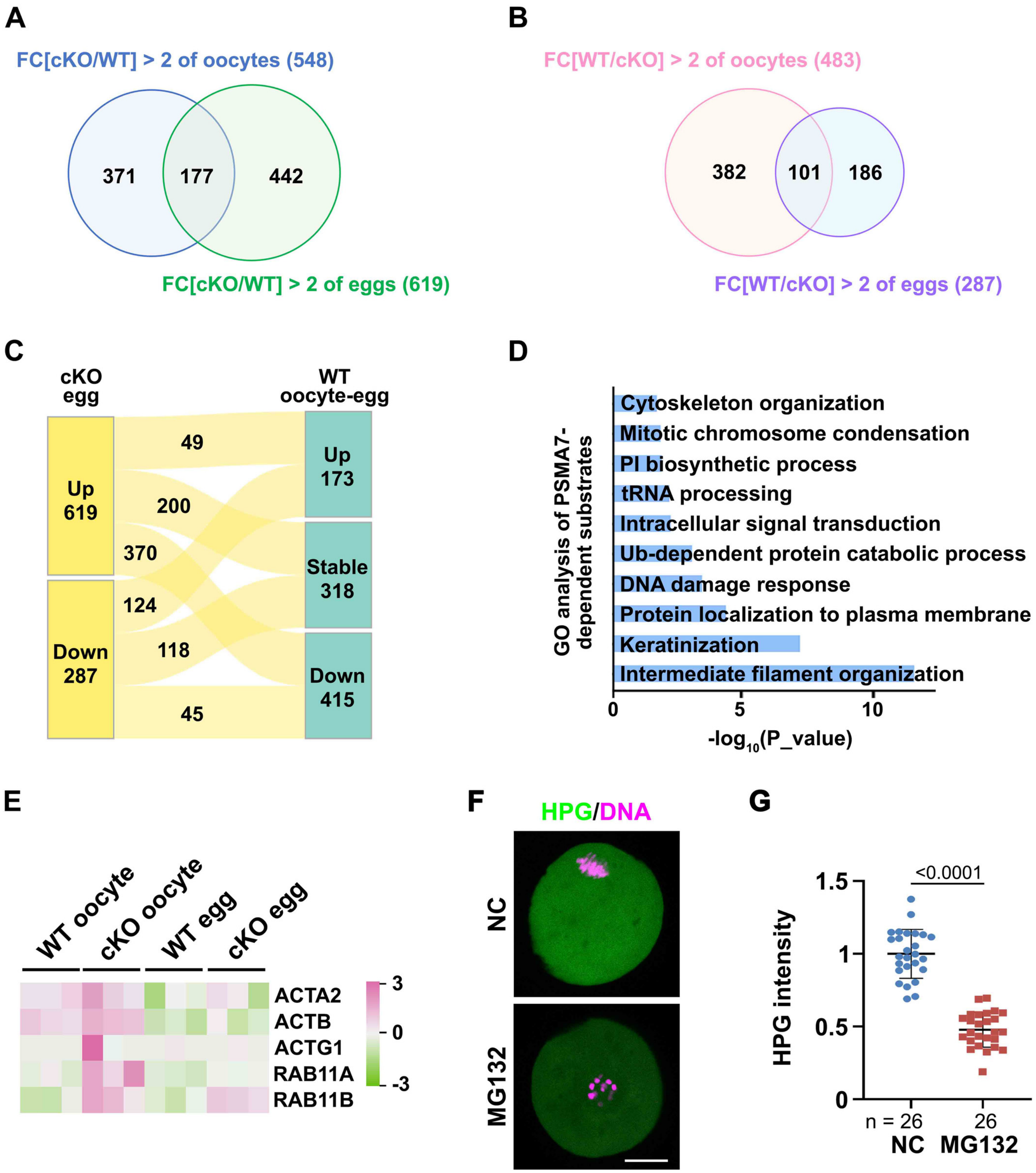

Figure EV2. The dynamic proteomic landscape correlates with oocyte developmental competence and progression.

(**A**, **B**) Venn diagram illustrating the overlap of significantly upregulated (**A**) or downregulated (**B**) proteins in both GV oocytes and eggs upon maternal PSMA7 depletion. (**C**) Sankey diagram showing how PSMA7 deficiency redirects the proteomic landscape, detailing shifts in up- and down-regulated proteins between WT and cKO eggs. (**D**) GO analysis of PSMA7-dependent substrates from Fig. 4H. Results are analyzed using the DAVID web server. (**E**) Heat map of actin-dependent relocation-related component levels in WT and *Psma7* cKO oocytes. (**F**) HPG fluorescent staining indicating protein synthesis activity in MI oocytes treated with or without MG132 treatment. Scale bar, 20 µm. (**G**) Quantification of HPG signal MFI in panel (**F**). *n* indicates number of oocytes. Mean and SD are shown. *P* values: unpaired *t* test.

 © The Author(s)

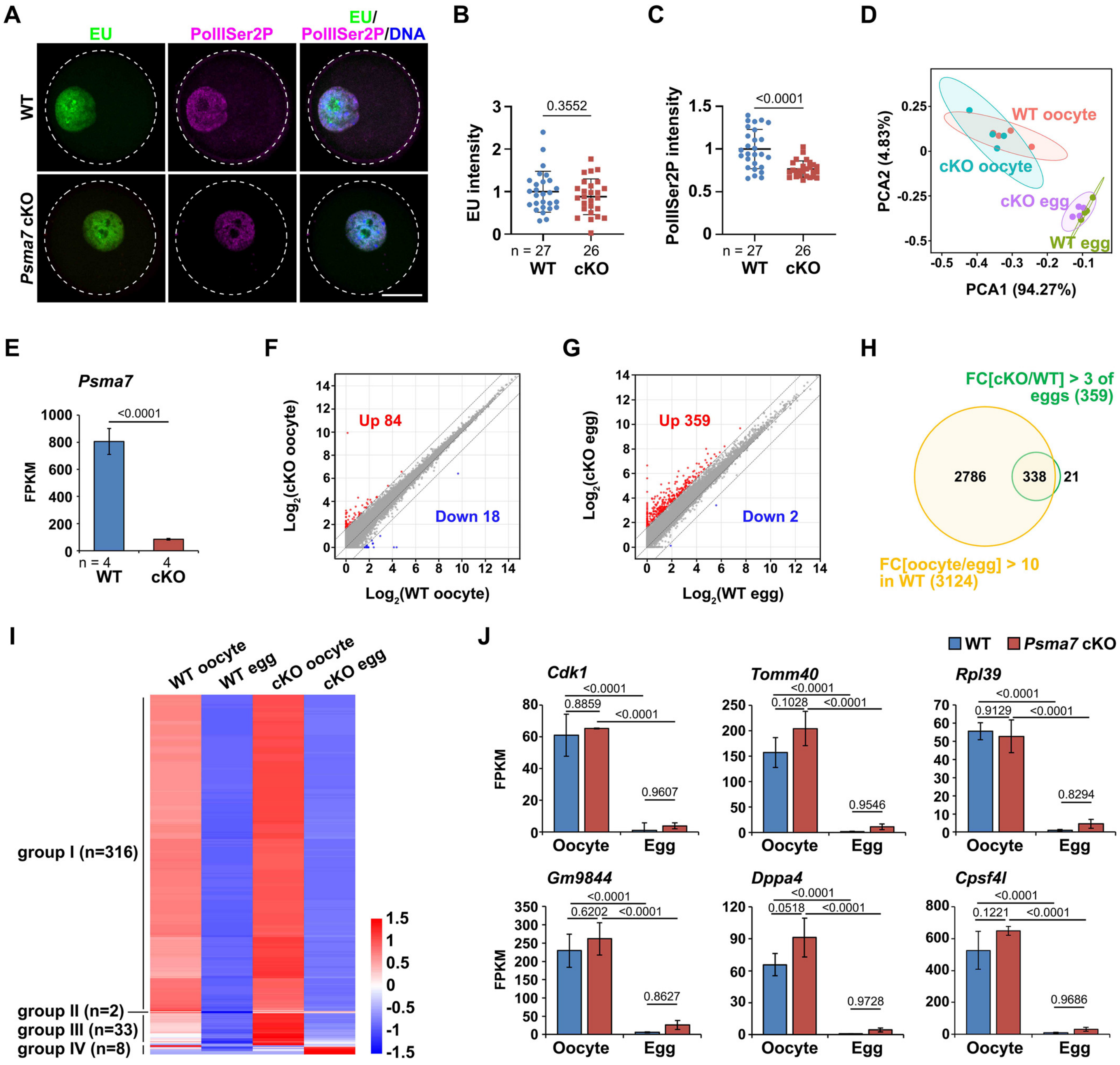

Figure EV3. PSMA7 depletion has no significant effect on oocyte transcriptome.

(**A–C**) Confocal analysis of EU and PolIISer2P signals (**A**), and quantification of relevant MFI (**B**, **C**) in WT and *Psma7* cKO GV oocytes. Scale bar, 20 μm. *n* indicates the number of oocytes analyzed. Mean and SD are shown. *P* values: unpaired *t* test. (**D**) PCA analysis of RNA-seq data from GV oocytes and eggs derived from WT and *Psma7* cKO mice. Results are analyzed using the SRplot web server. (**E**) RNA-seq results showing the relative expression levels of *Psma7* in GV oocytes of WT and *Psma7* cKO females. Analysis was conducted from four replicates of each genotype by RNA-seq. Mean and SD are shown. *P* values: unpaired *t* test. (**F**, **G**) Scatter plot depicting differentially expressed transcripts in *Psma7* cKO GV oocytes (**F**) and eggs (**G**) compared to WT controls. Transcripts with more than threefold change are highlighted in red (upregulated) or blue (downregulated). (**H**) Venn diagram displaying common transcripts that are downregulated during the GV–MII transition of WT groups and upregulated in *Psma7* cKO eggs. (**I**) Heat map displaying the expression of upregulated transcripts identified in panel (**G**) during the GV–MII transition in WT and *Psma7* cKO oocytes. Group I and III: transcripts upregulated in both GV oocytes and eggs after *Psma7* deletion, with Group III showing stronger upregulation in *Psma7* cKO GV oocytes. Group II: transcripts that should be downregulated during maturation remained stable after *Psma7* deletion. Group IV: genes upregulated in *Psma7* cKO eggs. Gene counts for each group are indicated. (**J**) RNA-seq results showing the relative expression levels of selected transcripts in GV oocytes and eggs of WT and *Psma7* cKO females. Four replicate results of each group by RNA-seq were used. Mean and SD are shown. *P* values: one-way ANOVA.

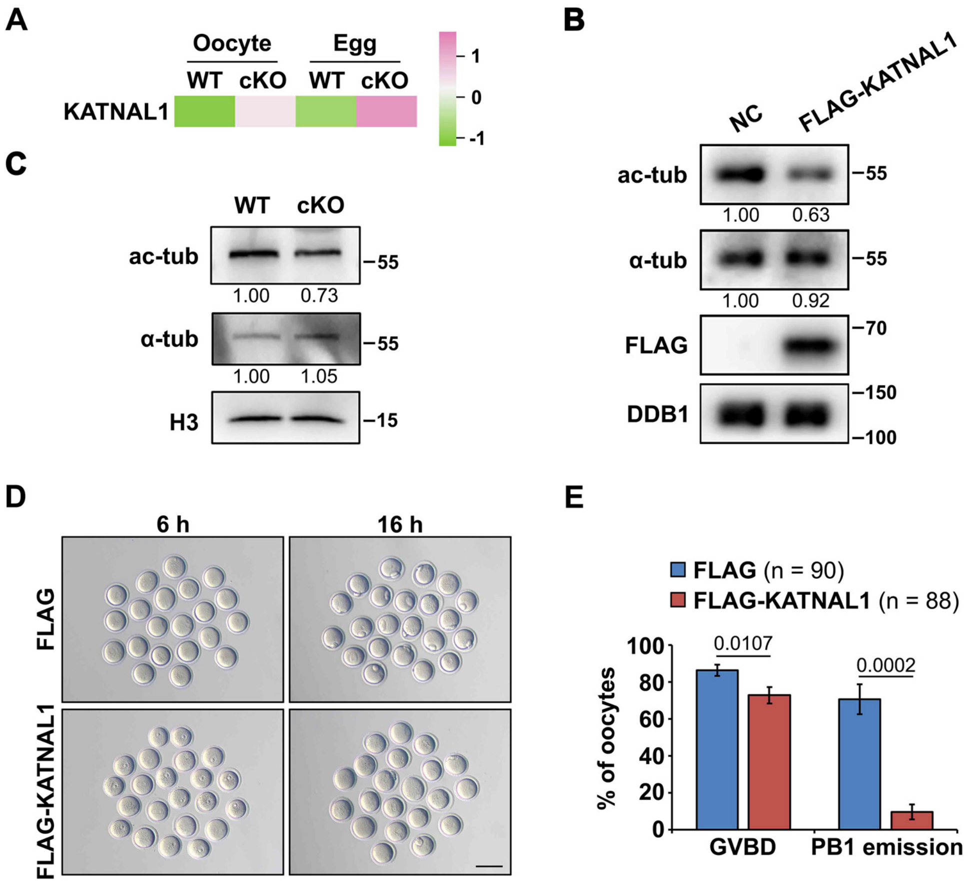

Figure EV4. The accumulation of KATNAL1 leads to meiotic defects in *Psma7* cKO oocytes.

(**A**) Heat map of KATNAL1 protein levels by proteomic analysis in WT and *Psma7* cKO oocytes. (**B**) Western blot analysis of acetylated α-tubulin (ac-tub) and total α-tubulin (α-tub) in control and overexpressing KATNAL1 eggs. (**C**) Western blot analysis of ac-tub and α-tub in WT and *Psma7* cKO eggs. DDB1 and H3 serve as the loading control. (**D**) Representative images of control and overexpressing KATNAL1 oocytes following in vitro culture. Scale bar, 100 μm. (**E**) Rates of GVBD and PB1 extrusion of oocytes cultured in vitro. n indicates the number of oocytes analyzed. Mean and SD are shown. *P* values: unpaired *t* test.

 © The Author(s)

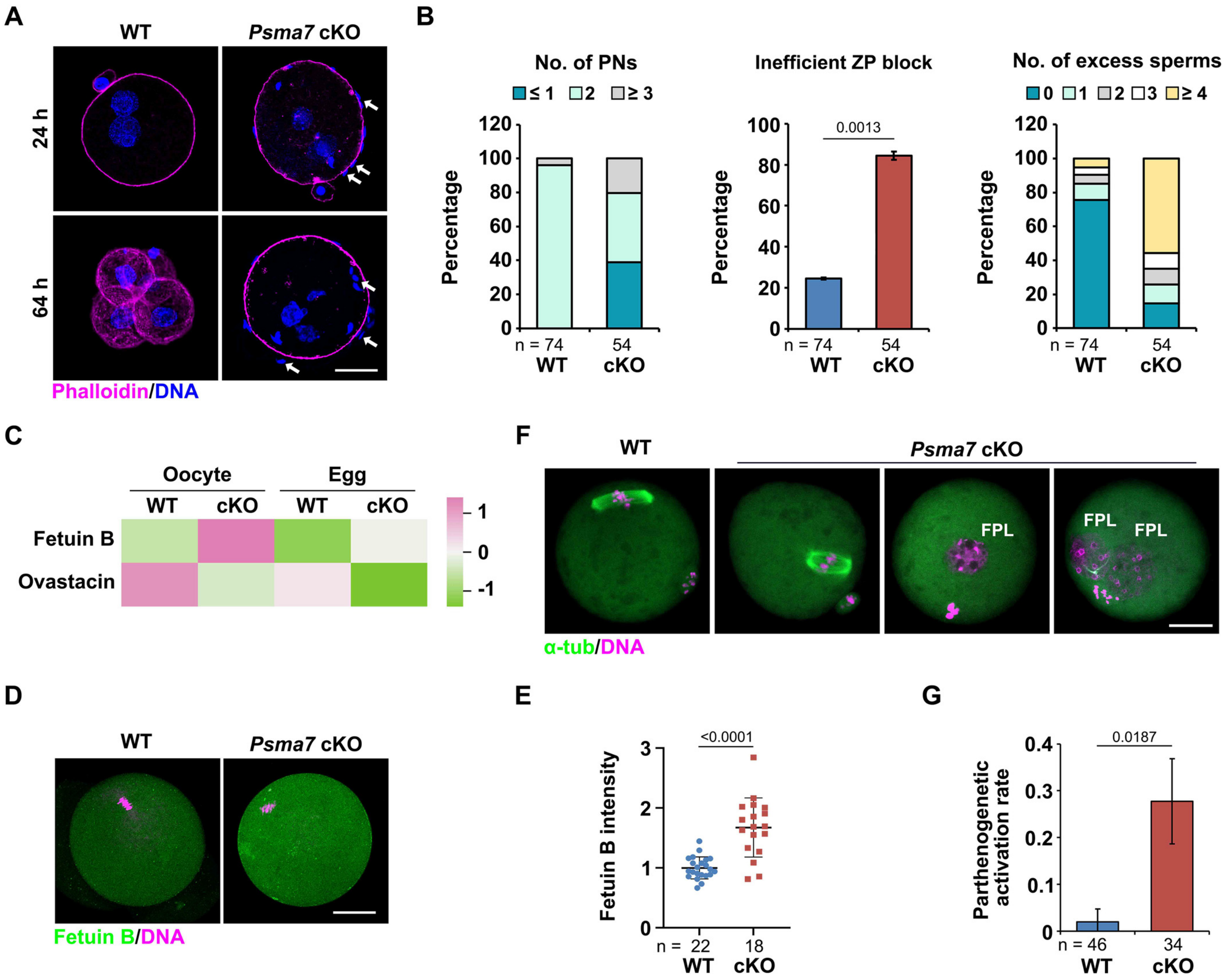

Figure EV5. Maternal PSMA7 deficiency leads to polyspermy and parthenogenetic activation in oocytes.

(**A**) Confocal analysis of embryos following IVF at 24 h and 64 h post-hCG. The arrows indicate excess sperms adhering to the cortex. (**B**) Quantification of pronuclear count, inefficient zona pellucida block rate, and excess sperm count in zygotes 8 h post-IVF. Mean and SD are shown in the middle image. *P* values: unpaired *t* test. (**C**) Heat map of Fetuin B and Ovastacin protein levels in WT and *Psma7* cKO oocytes. (**D**, **E**) Confocal analysis of Fetuin B signal (**D**) and quantification of the MFI (**E**) in WT and *Psma7* cKO eggs. Scale bar, 20 μm. Mean and SD are shown. *P* values: unpaired *t* test. (**F**) Fluorescence images showing normal and female pronucleus-like (FPL) structures in WT and *Psma7* cKO eggs after 48 h of in vitro culture. Scale bar, 20 μm. (**G**) Parthenogenetic activation rates in WT and *Psma7* cKO eggs in (**F**). Mean and SD are shown. *P* values: unpaired *t* test.

© The Author(s)
