# Supplementary material for: 20S proteasome-regulated proteostasis in ELVAs is critical for oocyte-to-embryo transition and female fertility
Source: EMBO J. 2026 May 21;45(14):4887–909. doi: 10.1038/s44318-026-00813-0 (PMC13373198; doi:10.1038/s44318-026-00813-0)
Supplement: Supplementary file 4 — Peer Review File [file 44318_2026_813_MOESM4_ESM.pdf]

THE EMBO JOURNAL

20S Proteasome-Regulated Proteostasis in ELVAs is Critical for Oocyte-to-Embryo Transition and Female Fertility

Yan Rong, Yingyan Chen, Huiwen Cao, Chengkan Liu, Haomang Xu, Xiaomei Tong, Anxuan Fang, Leilei Ai, Yucong Zhu, Yingyi Zhang, Peipei Ren, Xiaoxuan Li, Yufan Gao, Xiaolin Tian, Lugeng He, Songying Zhang, and Chao Yu

Corresponding author(s): Chao Yu (chao_yu@zju.edu.cn) , Songying Zhang (zhangsongying@zju.edu.cn), Lugeng He (fengyuzhe88@zju.edu.cn)

Review Timeline:

Submission Date:	4th Jan 26
Editorial Decision:	28th Jan 26
Revision Received:	11th Mar 26
Editorial Decision:	23rd Mar 26
Revision Received:	30th Apr 26
Accepted:	7th May 26

Editor: Hartmut Vodermaier

Transaction Report:

(Note: With the exception of the correction of typographical or spelling errors that could be a source of ambiguity, letters and reports are not edited. Depending on transfer agreements, referee reports obtained elsewhere may or may not be included in this compilation. Referee reports are anonymous unless the Referee chooses to sign their reports.)

Dr. Chao Yu
Zhejiang University
Hangzhou
China

28th Jan 2026

Re: EMBOJ-2026-123472
20S Proteasome-Regulated Proteostasis in ELVAs is Critical for Oocyte-to-Embryo Transition and Female Fertility

Dear Dr. Yu,

Thank you for submitting your manuscript on proteasome roles in the mammalian oocyte-to-embryo transition for our consideration. I sent it to three expert referees, who have now returned the reports that are copied below. As you will see, all referees consider this work a significant step forward on an important topic; but they also raise a number of substantive experimental and presentational issues that would need to addressed prior to publication. Should you be able to satisfactorily address these concerns, we would be interested in considering a revised version of this manuscript further for EMBO Journal publication.

Please note, however, that our single-major-revision-round policy makes it important to comprehensively respond to each referee point at the time of resubmission. I would therefore encourage you to contact me with a preliminary point-by-point response and revision plan already during the early stages of your revision work, in order to clarify if and how key issues raised in the reports may be solved. We would also be open to extension of the regular three-months revision period if needed; our 'scooping protection' (meaning that competing work appearing elsewhere in the meantime will not affect our considerations of your study) would of course remain valid also throughout such an extension.

Further information on preparing, formatting and uploading a revised manuscript can be found below and in our Guide to Authors. Thank you again for the opportunity to consider this work for The EMBO Journal, and I look forward to hearing back from you.

Yours sincerely,

Hartmut Vodermaier

Hartmut Vodermaier, PhD
Senior Editor, The EMBO Journal
h.vodermaier@embojournal.org

*** PLEASE NOTE: All revised manuscript are subject to initial checks for completeness and adherence to our formatting guidelines. Revisions may be returned to the authors and delayed in their editorial re-evaluation if they fail to comply to the following requirements. As a first step please read our guidelines for revised submissions:
https://link.springer.com/journal/44318/submission-guidelines#cms-Revised-submissions

1) Every manuscript requires a Data Availability section (even if only stating that no deposited datasets are included). Primary datasets or computer code produced in the current study have to be deposited in appropriate public repositories prior to resubmission, and reviewer access details provided in case that public access is not yet allowed.

2) Each figure legend must specify
- size of the scale bars that are mandatory for all micrograph panels
- the statistical test used to generate error bars and P-values
- the type error bars (e.g., S.E.M., S.D.)
- the number (n) and nature (biological or technical replicate) of independent experiments underlying each data point
- Figures may not include error bars for experiments with n<3; scatter plots showing individual data points should be used instead.

3) Revised manuscript text (including main tables, and figure legends for main and EV figures) has to be submitted as editable text file (e.g., .docx format). We encourage highlighting of changes (e.g., via text color) for the referees' reference.

4) Each main and each Expanded View (EV) figure should be uploaded as individual production-quality files (preferably in .eps,

.tif, .jpg formats). For suggestions on figure preparation/layout, please refer to our Figure Preparation Guidelines: https://media.springernature.com/original/springer-cms/rest/v1/content/27825798/data/v1

5) Point-by-point response letters should include the original referee comments in full together with your detailed responses to them (and to specific editor requests if applicable), and also be uploaded as editable (e.g., .docx) text files.

6) Please complete our Author Checklist, and make sure that information entered into the checklist is also reflected in the manuscript; the checklist will be available to readers as part of the Review Process File.

7) All authors listed as (co-)corresponding need to deposit, in their respective author profiles in our submission system, a unique ORCiD identifier linked to their name. Please see our Guide to Authors for detailed instructions.

8) Please note that supplementary information at EMBO Press has been superseded by the 'Expanded View' for inclusion of additional figures, tables, movies or datasets; with up to five EV Figures being typeset and directly accessible in the HTML version of the article.

9) To facilitate reproducibilty and cross-laboratory adoption of methodologies, please structure the Materials & Methods section as outlined in our guide to authors, including a completed Reagents and Tools Table.

10) Digital image enhancement is acceptable practice, as long as it accurately represents the original data and conforms to community standards. If a figure has been subjected to significant electronic manipulation, this must be clearly noted in the figure legend and/or the 'Materials and Methods' section. The editors reserve the right to request original versions of figures and the original images that were used to assemble the figure. Finally, we generally encourage uploading of numerical as well as gel/blot image source data.

At EMBO Press we ask authors to provide source data for the main manuscript figures. You will receive a separate email with instructions for providing source data with your revised manuscript, including how to upload and organize the files.

In the interest of ensuring the conceptual advance provided by the work, we recommend submitting a revision within 3 months (28th Apr 2026). Please discuss the revision progress ahead of this time with the editor if you require more time to complete the revisions. Use the link below to submit your revision:

Link Not Available

Referee #1:

This manuscript by Rong et al, evaluates proteostasis changes with advanced maternal age in mouse and human oocytes. They identify a component of the 20S proteosome, PSMA7, that appears altered in expression with age and they create and evaluate an oocyte-specific knockout mouse strain. Through a battery of tests, they find that KO oocytes have protein turnover defects, translation defects, and many hallmarks of poor egg quality. This manuscript is a strong step forward in understanding how protein translation and turnover are regulated in oocytes and how this impacts gamete quality. However, I have a number of queries and points of concern that I would like the authors to address.

1. The authors present many western blots, however they are all cropped. The authors must present full length blot images in the supplement to demonstrate antibody specificity.
2. The writing, although clear, frequently lacks rationale. It is hard for the reader to understand why experiments were being performed. For example, I where I understand why protein stability would be assessed in a proteosome mutant, I don't understand why they would look at translation. Why would translation be decreased? Is this because the oocytes are not in comparative cell cycle stages or something more direct? What is the rationale to conduct the experiments in Figure 3 with MG132?
3. Some wording is off too. For example, "nature miscarriage" does not make sense. Do the authors mean natural miscarriage; in which case just say miscarriage? The authors should define oocyte/egg in the beginning. The first line of the results is otherwise confusing if a reader does not realize that the first result are prophase I arrested oocytes and that the 2nd result is a MII egg. Even though I am well versed in this field, it took me a few reads to understand the subtlety. Line 252- should read "structural organizer." What is microproteomic analysis? Is this different from proteomic analysis? The use of "entangled chromosome" is not precise. Because these oocytes are in Telophase, they are chromosome bridges.
4. The authors quantify the sizes of ELVA markers using diameter. This is not appropriate as a diameter could easily change depending on which z slice the center of the structure is in. The authors should re-analyze all these experiments using volume, taking into account the entire structure in the z dimension.
5. In figure 3 the authors are describing a difference in ELVA positioning in the oocytes. Again jargon based wording is making

their claim confusing. Instead of saying "ooplasm" state that they fail to migrate to the cortex.
6. The authors spend a whole figure showing no change in transcription. This seems like a control experiment and the result is not surprising- consider moving it to the supplement.
7. The statistical analyses require revisiting. The methods indicate only using a t-Test, however, some graphs evaluate more than 2 groups and should instead use an ANOVA test.
8. I strongly recommend that the authors adjust their image colors to a color combination palette that is red/green color blind friendly

Referee #2:

Rong et al. presented a compelling study that elucidates the crucial role of proteasomal degradation in mouse oocytes, with implications for understanding the oocyte-to-embryo transition and the mechanisms underlying reproductive aging. The work is notable for its innovative insights, particularly in revealing how aging affects proteasome composition and activity across species. The authors first show that key proteasomal subunits-including several α-subunits and PSMA7-are downregulated at both transcript and protein levels in aged human and mouse oocytes, leading to measurably reduced proteasome activity at GV and MII stages. This decline correlates with mis-localized ELVAs and pronounced accumulation of protein aggregates, providing a clear phenotypic link to aging. By generating a Psma7 conditional knockout mouse model, the authors demonstrate that PSMA7 is essential for proteasome stabilization. The resulting defects-including impaired ELVA translocation and disrupted proteostasis-closely mirror those observed in naturally aged oocytes, thereby establishing a direct mechanistic connection. The experimental evidences strongly support the conclusion that dysfunction in proteasomal degradation arrests both oocyte maturation and early embryonic development at the 1-to-2 cell stage.
This study provides physiologically important insights into the regulation of maternal protein clearance and directly implicates dysregulated proteasomal function as a key contributor to oocyte quality decline during aging. These findings open promising avenues for further research into proteostasis mechanisms in reproductive longevity. I have listed several comments below that may assist the authors in further strengthening the manuscript.

Major concerns:
1. Some of the reported claims related to oocyte aging appear to be in contrast. Harasimov et al. (2024) recently reported no decline in proteasomal activity in aged oocytes. Bettina et al. (2018) and the author of this manuscript suggest decreased proteasomal activity. What are the differences?
2. The proteomics data is well processed, but the analysis of "PSMA7-dependent substrates" could go deeper. Running a systematic bioinformatics analysis (GO, KEGG, protein interaction networks) on the 243 substrates from Fig. 4H might reveal key pathways and regulatory nodes more clearly.
3. The proteasome is required for the cell cycle progression, including meiosis, is long known (see Terret et al., 2003, for mouse oocytes). Therefore, it is easily conceivable that any major intervention that alters the proteasome composition, assembly, or activity (such as the KO of a core subunit like PSMA7) would result in severe defects in the cell cycle progression, including in oocytes (e.g. PB1 extrusion). However, to my surprise, ~70% of oocytes from PSMA7 cKO mice did apparently PB1 extrusion (Fig. 6D and S5B). Is that due to the residual proteasomal activity after PSMA7 cKO?
4. Related to the point above, it is surprising that the genetic deletion of PSMA7, a core proteasome subunit, results in quite a substantial residual PSMA7 protein level (Fig. 2B-D, Fig. 4J, and Fig. S2G). Similarly, PSMA7 deletion does not cause a complete absence in proteasomal activity (Fig. 2E). Are oocytes storing PSMA7 mRNA? Or maybe the knockout efficiency of this zp3-cre mouse line is not thorough enough. Has this mouse line been used for the deletion of other genes before? Or maybe PSMA7 is indeed a long-lived protein. Please address the significance of its longevity.
5. Can aged oocytes be rescued by over-expression of PSMA7 or 20S subunit? That will be a potential approach for treating female reproductive aging.
6. Can small molecule activators be developed to enhance proteasome activity in order to improve the quality of aged oocytes? It is suggested to extend this direction in the discussion section.
7. Fig. 5A: Most of the experiments are performed on mice aged 21-23 days. Why are the authors switching to mice aged 13.5 days here? This potentially introduces unnecessary variability between samples, rendering data interpretation difficult.

Minor concerns:
1. Figure 1E is re-plotted from a previously published manuscript. That original paper clearly notes that most of the differentially expressed genes depicted were not statistically significant. I'm worried that using this set of data to support authors' claims in the current manuscript may be problematic.
2. In the proteomics results, the authors mention that "32.3% (177/548) of proteins up-regulated in oocytes remained elevated in eggs." Adding these actual numbers to the Venn diagram in Fig. S4G (and similarly in Figs. 4G, 4H, S4H) would make the data much easier to interpret.
3. Some cited papers are from the early 2000s. If there are more recent studies (within the last 5 years) on related topics, consider updating or supplementing the references to reflect current knowledge.
4. Figure S3C: Could the author clarify the concentration and treatment time for BAFA1 in the Methods? Adding those details will help others reproduce the experiment.
5. Fig. 6E: For WT oocyte image, it appears that the "MII" stage label is missing from the upper-right corner.

6. Fig. S5: The phalloidin immunofluorescent signals are too weak.
7. Fig. 8: Polyspermy occurs as a consequence of sperm entry, so "Further polyspermy" should exist after sperm appearance.
8. Phrases like "slightly reduced" (page 10) are a bit vague. Whenever possible, it's helpful to tie descriptions to actual data-for example, "reduced by roughly 20%, though not statistically significant."

Referee #3:

This manuscript investigates the function of the 20S proteasome in mouse oocytes by characterizing phenotypes in their newly developed oocyte-specific Psma7 knockout (KO) model, and further reports similarities between the KO phenotypes and age-associated phenotypes.

The authors first show that in aged mouse oocytes, the extent of the maturation-associated increase in proteasome activity is limited, and that ELVAs are abnormally enlarged. They report that transcripts encoding 20S proteasome subunits are reduced in aged oocytes, and that this reduction is shared between mouse and human. In addition, they show that protein aggregates increase with age in human oocytes.

Next, the authors generate an oocyte-specific Psma7 KO mouse line and examine its oocyte phenotypes. Mature eggs derived from Psma7 KO oocytes display reduced proteasome activity, resembling aged eggs. ELVAs containing protein aggregates are enlarged, again similar to aged oocytes. Interestingly, ELVAs additionally show an apparent defect in migration toward the cortex. Among these phenotypes, enlargement of ELVAs with elevated lysosomal activity is phenocopied by treatment with the proteasome inhibitor MG132.

The authors then present a mass spectrometry-based characterization of proteome changes in Psma7 KO oocytes. They identify proteins that are down- or up-regulated upon KO and interpret these results as indicating defects in both protein degradation and protein synthesis during oocyte maturation. This proteomics analysis is comprehensive and represents a strength of the study. A defect in protein synthesis is also supported by metabolic labeling experiments, whereas transcript labeling suggests that defects in de novo transcription are relatively modest.

Finally, the authors describe terminal phenotypes of Psma7 KO oocytes: a high frequency of failure to complete maturation, and defective post-fertilization development.

Based on these results, the authors conclude that 20S proteasome-dependent proteostasis in oocytes is critical for female fertility, and that this function declines with aging.

Overall, the manuscript reports cell biological and proteomic phenotyping using a robust genetic model (oocyte-specific Psma7 conditional KO), and it is valuable in demonstrating that several KO phenotypes overlap with those observed in aged oocytes. Assuming that the quantitative image-based analyses underlying the cellular phenotypes are performed appropriately, the major conclusions are supported by substantial experimental evidence. If the authors can adequately address the points below, I believe this work would make an important contribution to the field.

Major comments:
1. A central approach in this study is quantitative analysis of microscopy images to define cellular phenotypes. The resulting data-such as signal intensities of proteostasis-related markers, and the size and subcellular positioning of ELVAs and related structures-directly support the authors' conclusions. However, the manuscript currently provides far too little methodological detail on how these quantifications were performed. For example: How were ROIs defined? Were objective, quantitative rules established for segmentation? If any steps were performed manually, how was objectivity ensured? Was blinding implemented? Were analyses performed in 2D or 3D? How and from where was background estimated and subtracted? Many plots appear to show one data point per oocyte; when multiple relevant structures exist within a single oocyte, how was the reported value selected or calculated (e.g., mean/median across structures, largest structure, closest-to-cortex structure, etc.)? Because the manuscript includes multiple distinct types of quantification across figures, the authors should provide a thorough description for each analysis. Doing so would substantially strengthen confidence in the quantitative phenotypes and, consequently, in the conclusions.

2. The impaired migration of ELVAs toward the cortex in Psma7 KO oocytes is interesting. It is not clear whether an analogous defect is present in aged oocytes. If cortical migration is normal in aged oocytes, this would be worth stating explicitly, as it would help distinguish proteasome-dependent phenotypes that overlap with aging from those that may be specific to Psma7 loss.

3. Related to the point above, the authors report that RAB11, which has been implicated in ELVA cortical translocation, is upregulated in Psma7 KO oocytes. This is somewhat difficult to reconcile with the observation that ELVAs nonetheless exhibit impaired migration toward the cortex in the KO. The manuscript further states that "elevated RAB11 may compensate for the impaired cortical translocation of overloaded ELVAs," but it is not clear what is meant by this. More explicit discussion of why ELVA cortical migration appears defective in Psma7 KO oocytes, and if such defects are not observed in aged oocytes,

discussions of why not in aged oocytes would be helpful (e.g., whether RAB11 upregulation is interpreted as a partial/insufficient compensation, and/or whether additional limiting factors are involved).

Referee #1:

This manuscript by Rong et al, evaluates proteostasis changes with advanced maternal age in mouse and human oocytes. They identify a component of the 20S proteosome, PSMA7, that appears altered in expression with age and they create and evaluate an oocyte-specific knockout mouse strain. Through a battery of tests, they find that KO oocytes have protein turnover defects, translation defects, and many hallmarks of poor egg quality. This manuscript is a strong step forward in understanding how protein translation and turnover are regulated in oocytes and how this impacts gamete quality. However, I have a number of queries and points of concern that I would like the authors to address.

Response: We sincerely thank the reviewer for your constructive suggestions and valuable efforts to enhance the quality of our manuscript. We have carefully addressed each of the issues raised and incorporated corresponding revisions in red into the manuscript.

1. The authors present many western blots, however they are all cropped. The authors must present full length blot images in the supplement to demonstrate antibody specificity.

Response: We thank the reviewer for this constructive suggestion. We fully agree that complete blot images are essential for demonstrating antibody specificity and data integrity. However, due to the precious nature of oocyte and embryo samples, we employed the membrane cropping strategy before antibody incubation to maximize the detection of multiple target proteins from one WB assay. This approach is commonly adopted in reproductive biology studies where sample quantity is severely constrained [1].

Besides, the specificity of most antibodies we used have been validated by published studies, such as PSMA7 [2], α-sub [2], RUFY1 [3], DDB1 [4], Cyclin B1 [4], and pERK1/2 [4]. To ensure the accuracy of our data, all target protein sizes were strictly cross-referenced with theoretical molecular weights and antibody datasheets, and the membrane cutting positions were precisely determined based on visible protein marker bands. Each experiment was independently repeated at 2 to 3 times with consistent results.

Moreover, we have now updated annotated full-scan images of the properly cropped membranes as the Source Data files to editor system, and re-checked the size

of the bands in revised manuscript. We hope this clarification satisfies the reviewer's concerns regarding data reliability while acknowledging the practical constraints of working with limited oocyte materials.

2. The writing, although clear, frequently lacks rationale. It is hard for the reader to understand why experiments were being performed. For example, I where I understand why protein stability would be assessed in a proteosome mutant, I don't understand why they would look at translation. Why would translation be decreased? Is this because the oocytes are not in comparative cell cycle stages or something more direct? What is the rationale to conduct the experiments in Figure 3 with MG132?

Response: We sincerely thank the reviewer for pointing out our deficiencies. The clear rationale is essential for scientific communication, and we acknowledge that our original manuscript lacks sufficient explanation for several experimental designs, creating unnecessary cognitive burden for readers. We have now provided necessary information to describe the rationales in experimental designs. Specifically, responses to the points raised by the reviewer are provided below:

1) Protein degradation and translation are two core links in protein homeostasis, so we examined translation activity to rule out the possibility that accumulated proteins result from increased synthesis. We have supplemented the reason of conducting this experiment in revised manuscript. The results show a decrease in translation activity, suggesting that PSMA7 deficiency may have activated the cellular stress response, or lead to an adaptive energy-saving response of cells when they encounter disruptions (line 423-425 in the revised manuscript).

2) Maternal *Psma7* knockout is a chronic deficiency throughout oocyte development, which may trigger compensatory mechanisms, and the phenotype may be caused by indirect developmental defects. While MG132 is a transient inhibition of the proteasome, eliminating the interference of compensation and directly demonstrating that the decreased translational activity and abnormality of ELVAs is caused by decreased proteasome activity (line 355-358 in the revised manuscript).

3. Some wording is off too. For example, "nature miscarriage" does not make sense. Do the authors mean natural miscarriage; in which case just say miscarriage? The authors should define oocyte/egg in the beginning. The first line of the results is otherwise confusing if a reader does not realize that the first result are prophase I

arrested oocytes and that the 2nd result is a MII egg. Even though I am well versed in this field, it took me a few reads to understand the subtlety. Line 252- should read "structural organizer." What is microproteomic analysis? Is this different from proteomic analysis? The use of "entangled chromosome" is not precise. Because these oocytes are in Telophase, they are chromosome bridges.

Response: We thank the reviewer for the careful line-by-line reading and apologize for these imprecisions. We have revised "nature miscarriage" as "miscarriage" (line 69), "structure organizer" as "structural organizer" (line 283), "entangled chromosome" as "chromosome bridges" (line 469, 477, and Fig. 5G), and used "GV oocytes" to refer to "prophase I arrested oocytes", and "egg" to "MII egg" in the revised text. Microproteomics is a specialized branch of proteomics that focuses on the analysis of protein profiles from extremely limited biological materials, such as oocytes. We have added the explanation behind the word (line 374-375). Besides, we have checked the whole manuscript to avoid similar issues in the revised version.

4. The authors quantify the sizes of ELVA markers using diameter. This is not appropriate as a diameter could easily change depending on which z slice the center of the structure is in. The authors should re-analyze all these experiments using volume, taking into account the entire structure in the z dimension.

Response: We thank the reviewer for raising this point. The methodological detail on how these quantifications were performed are as below, and could also be found in revised "Method" part (line 210-220):

(1) For image acquisition, the maximum cross-section of each oocyte was selected to ensure consistency in section or focal plane selection; therefore, the analysis was performed in 2D.

(2) Relative diameter calculation: The diameter of each ELVA with the maximum cross-section was measured and divided by the diameter of the corresponding oocyte to obtain the relative diameter of that ELVA. The average relative diameter was then calculated for each oocyte, and statistical comparisons were performed across more than ten oocytes.

(3) Relative distance from cortex calculation: The distance from the center of each ELVA to the oocyte cortex was measured and divided by the oocyte radius to obtain the relative cortical distance of that ELVA. The average relative distance from cortex of all ELVAs was calculated for each individual oocyte, followed by

statistical analysis and inter-group comparison across multiple oocytes.

5. In figure 3 the authors are describing a difference in ELVA positioning in the oocytes. Again jargon based wording is making their claim confusing. Instead of saying "ooplasm" state that they fail to migrate to the cortex.

Response: We sincerely thank the reviewer for the constructive input and have corrected the wording in revised manuscript (line 346).

6. The authors spend a whole figure showing no change in transcription. This seems like a control experiment and the result is not surprising- consider moving it to the supplement.

Response: We appreciate the reviewer's suggestion to streamline the main figures. We have moved it to the supplemental part as the revised Fig. S5.

7. The statistical analyses require revisiting. The methods indicate only using a t-Test, however, some graphs evaluate more than 2 groups and should instead use an ANOVA test.

Response: We appreciate the reviewer's careful examination of our statistical methods. Some of our graphs indeed evaluate more than two groups and we have now re-analyzed the multi-group comparisons using one-way ANOVA accordingly, such as Fig. 1B, 2F, 2H, 4F, 4K and S5J. We confirm that the conclusions remain unchanged after re-analysis with appropriate statistical tests. Although Fig. 1D, 1H, 5A, 5D, S6B, S6E and S7E contain more than two groups, each comparison was specifically conducted between pairs of groups rather than across all groups simultaneously; therefore, the Student's T-test is retained. The description of the corresponding method section has been revised (line 267-269), and the statistical test used for data analysis and the error bar definition are shown in the figure legend parts.

8. I strongly recommend that the authors adjust their image colors to a color combination palette that is red/green color blind friendly

Response: We sincerely thank the reviewer for this important suggestion regarding color accessibility. We completely agree that scientific figures should be inclusive and interpretable. We have systematically revised all figures containing red/green color

combinations to magenta/green combination, which remains distinguishable for red/green color-blind and normal vision readers.

Referee #2:

This study provides physiologically important insights into the regulation of maternal protein clearance and directly implicates dysregulated proteasomal function as a key contributor to oocyte quality decline during aging. These findings open promising avenues for further research into proteostasis mechanisms in reproductive longevity. I have listed several comments below that may assist the authors in further strengthening the manuscript.

Response: We sincerely thank the reviewer for the constructive advices and the commitment to improve our manuscript. In response to the reviewer's comments, we have carefully addressed each point one by one, and have incorporated corresponding revisions in red into the manuscript.

Major concerns:

1. Some of the reported claims related to oocyte aging appear to be in contrast. Harasimov et al. (2024) recently reported no decline in proteasomal activity in aged oocytes. Bettina et al. (2018) and the author of this manuscript suggest decreased proteasomal activity. What are the differences?

Response: We appreciate the reviewer's thoughtful suggestions.

We suggest that these differences are dependent on the developmental stage. Harasimov et al. have reported no decline in proteasomal activity in aged GV oocytes. Our study expands on this by examining both GV and MII stages. While our results similarly show comparable proteasomal activity in aged and young GV oocytes (Fig. 1A-B), we observed that upon meiotic maturation, proteasomal activity increases in young oocytes but remains lower in aged eggs (Fig. 1A-B). This suggests that the decline manifests specifically during maturation in aged eggs.

To ensure accuracy, we have carefully revised the description of these findings in the manuscript to more clearly delineate our findings from those of Harasimov et al. We thank the reviewer again for highlighting this important point and have emphasized the novelty of our findings with more nuanced discussion.

2. The proteomics data is well processed, but the analysis of "PSMA7-dependent substrates" could go deeper. Running a systematic bioinformatics analysis (GO, KEGG, protein interaction networks) on the 243 substrates from Fig. 4H might reveal key pathways and regulatory nodes more clearly.

Response: We thank the reviewer for improving our manuscript and have conducted the GO analysis of these proteins in revised Fig. S4J. The GO analysis reveals that 243 PSMA7-dependent substrates are mainly associated with intermediate filament organization, keratinization, protein localization to plasma membrane, cytoskeleton organization and so on. Compromised structural support and stability of cells may impair actin/RAB11A-dependent ELVAs relocation. We have added the content in revised Results (line 411-415) and Discussion (line 595-598) part.

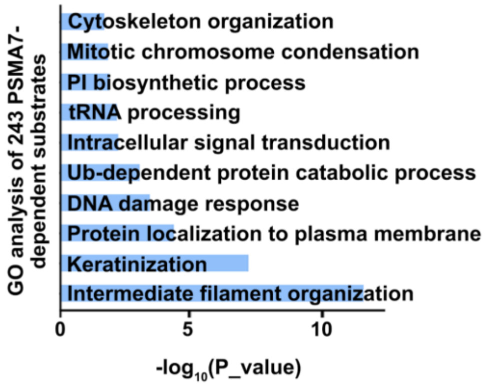

3. The proteasome is required for the cell cycle progression, including meiosis, is long known (see Terret et al., 2003, for mouse oocytes). Therefore, it is easily conceivable that any major intervention that alters the proteasome composition, assembly, or activity (such as the KO of a core subunit like PSMA7) would result in severe defects in the cell cycle progression, including in oocytes (e.g. PB1 extrusion). However, to my surprise, ~70% of oocytes from PSMA7 cKO mice did apparently PB1 extrusion (Fig. 6D and S5B). Is that due to the residual proteasomal activity after PSMA7 cKO?

Response: Thank you for raising this important point. PSMA7 protein exhibits high abundance and stability, proving its importance in oocytes. Even after *Psma7* gene knockout at the primary follicle stage, residual PSMA7 protein persists throughout oocyte growth and maturation. This model is physiologically relevant, as clinically poor-quality oocytes often exhibit reduced, rather than completely absent, proteasomal activity. Thus, we suggest that this is a nuanced model to investigate how declines in proteasomal activity in vivo affect oocyte competence and subsequent embryonic development. This novelty has been further emphasized in our manuscript.

4. Related to the point above, it is surprising that the genetic deletion of PSMA7, a core proteasome subunit, results in quite a substantial residual PSMA7 protein level

(Fig. 2B-D, Fig. 4J, and Fig. S2G). Similarly, PSMA7 deletion does not cause a complete absence in proteasomal activity (Fig. 2E). Are oocytes storing PSMA7 mRNA? Or maybe the knockout efficiency of this zp3-cre mouse line is not thorough enough. Has this mouse line been used for the deletion of other genes before? Or maybe PSMA7 is indeed a long-lived protein. Please address the significance of its longevity.

Response: We thank the reviewer for raising this important point. This ZP3-Cre mouse line has been used in many published works[5-9], proving its effective knockout efficiency, and we have supplemented this information in revised Method part. Besides, we have checked the expression of *Psma7* in WT and *Psma7* cKO GV oocytes from RNA-seq data. *Psma7* is significantly decreased after maternal *Psma7* deletion (see image below, revised Fig. S5E), so the cKO oocytes do not store *Psma7* mRNAs. However, By IHC and IF analyses reveal that residual PSMA7 protein persists throughout oocyte growth and maturation, even after *Psma7* gene knockout at the primary follicle stage. The long-lived longevity of PSMA7 protein implies the functional significance of proteasome-mediated degradation in oocytes, and we have addressed it in the manuscript.

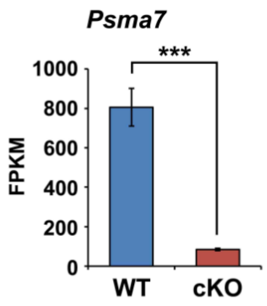

5. Can aged oocytes be rescued by over-expression of PSMA7 or 20S subunit? That will be a potential approach for treating female reproductive aging.

Response: We thank the reviewer for improving our manuscript. This direction has a solid mechanism foundation from our work. We have found that PSMA7 is a key node in 20S assembly and ELVAs assembly and translocation, and its absence leads to a phenotype highly similar to aging, suggesting the theoretical feasibility of the supplementary strategy. However, the decline of oocyte quality in advanced age is a multi-factorial, multi-level, and progressive systemic collapse process, involving decreased transcriptional level, aberrant epigenomics, organelle dysfunction, metabolic decline and impaired protein degradation mentioned here. Besides, oocytes

retrieved generally have typically exited the growth phase and ceased transcription, so the potential for supplementing PSMA7 at this time may be limited. This perspective underscores the importance of preventive rather than restorative interventions for age-related fertility decline. We have added this content in the revised Discussion part (line 630-637).

6. Can small molecule activators be developed to enhance proteasome activity in order to improve the quality of aged oocytes? It is suggested to extend this direction in the discussion section.

Response: We thank the reviewer for raising this point. Based on the answer to the last question, therapeutic activation of proteasome function represents a promising rescue direction arising from our findings. Unlike genetic approaches requiring mRNA microinjection, small-molecule activators offer pharmacological advantages including dose titratability and clinical scalability. We have extended this direction in the Discussion section (line 630-637).

7. Fig. 5A: Most of the experiments are performed on mice aged 21-23 days. Why are the authors switching to mice aged 13.5 days here? This potentially introduces unnecessary variability between samples, rendering data interpretation difficult.

Response: We thank the reviewer for raising this point. Please allow me to explain this experiment. During growth, oocytes undergo active transcription to accumulate maternal mRNAs, which support oocyte maturation, fertilization, and early embryonic development prior to ZGA. Upon reaching the fully-grown stage, oocytes turn to transcriptional quiescence, which persists until the 2-cell stage of embryonic development. Oocytes from 21-23-day-old mice which were treated with PMSG are fully-grown GV oocytes. At this stage, majority of oocytes have already transitioned to the SN nucleus configuration and are transcriptionally silent. In contrast, oocytes from 13.5-day-old mice are characterized by high global transcriptional activity. This stage was specifically chosen for transcriptional assays to capture dynamic transcriptional changes.

Minor concerns:

1. Figure 1E is re-plotted from a previously published manuscript. That original paper clearly notes that most of the differentially expressed genes depicted were not

statistically significant. I'm worried that using this set of data to support authors' claims in the current manuscript may be problematic.

Response: Thank you for raising this important point regarding the presentation of the data. Please allow me to explain this question. (1) Regarding the cited study (Wu YW et al., 2022), we would like to clarify that it did not conclude that the highlighted DEGs were statistically insignificant. Rather, the authors noted that the apparent lack of statistical significance for some transcriptome changes in earlier studies could be attributed to the absence of ERCC spike-in normalization. Without such normalization, fluctuations in transcripts that scale with total RNA content may be masked, potentially obscuring significant aging-related expression patterns. Our re-analysis and presentation of this dataset focus specifically on the comparability of the aging-associated transcriptional trend, which remains informative. (2) To further support our conclusions, we have exhibited our analysis with an independent, replotted dataset from Zhang Y et al. *Nat Aging*, 2023 (revised Fig. S1D). This RNA-seq dataset, although not normalized with ERCC spike-in, also shows a consistent reduction in 20S proteasome subunit transcripts in aged mouse oocytes, thereby reinforcing our original claim regarding age-related declines in proteasomal components. We believe these clarifications and the additional evidence strengthen the validity and transparency of our argument.

2. In the proteomics results, the authors mention that "32.3% (177/548) of proteins up-regulated in oocytes remained elevated in eggs." Adding these actual numbers to the Venn diagram in Fig. S4G (and similarly in Figs. 4G, 4H, S4H) would make the data much easier to interpret.

Response: We sincerely thank the reviewer for this kind suggestion and we have added the total number of each group in the revised images.

3. Some cited papers are from the early 2000s. If there are more recent studies (within the last 5 years) on related topics, consider updating or supplementing the references to reflect current knowledge.

Response: Thank you for pointing out our oversight and we have deleted the outdated references and updated the latest references in the revised manuscript.

4. Figure S3C: Could the author clarify the concentration and treatment time for

BAFA1 in the Methods? Adding those details will help others reproduce the experiment.

Response: Thank you for pointing out our oversight and we have corrected in the revised figure legend part (line 1016).

5. Fig. 6E: For WT oocyte image, it appears that the "MII" stage label is missing from the upper-right corner.

Response: Thank you for pointing out our oversight and we have corrected in the revised manuscript.

6. Fig. S5: The phalloidin immunofluorescent signals are too weak.

Response: Thank you for pointing out our oversight and we have changed the red color to magenta, and up-regulated the fluorescent signal intensity in the revised manuscript to make it more distinguishable for readers.

7. Fig. 8: Polyspermy occurs as a consequence of sperm entry, so "Further polyspermy" should exist after sperm appearance.

Response: Thank you for pointing out our oversight and we have corrected in the revised Fig.7.

8. Phrases like "slightly reduced" (page 10) are a bit vague. Whenever possible, it's helpful to tie descriptions to actual data-for example, "reduced by roughly 20%, though not statistically significant."

Response: Thank you for pointing out our inappropriate description and we have corrected it in the revised manuscript (line 332-333).

Referee #3:

Overall, the manuscript reports cell biological and proteomic phenotyping using a robust genetic model (oocyte-specific Psma7 conditional KO), and it is valuable in demonstrating that several KO phenotypes overlap with those observed in aged oocytes. Assuming that the quantitative image-based analyses underlying the cellular phenotypes are performed appropriately, the major conclusions are supported by substantial experimental evidence. If the authors can adequately address the points below, I believe this work would make an important contribution to the field.

Response: We sincerely thank the reviewer for the efforts in evaluating our work. According to the reviewer's comments, we have now carefully revised our manuscript and hope that the revised version meets your publication standard.

Major comments:

1. A central approach in this study is quantitative analysis of microscopy images to define cellular phenotypes. The resulting data-such as signal intensities of proteostasis-related markers, and the size and subcellular positioning of ELVAs and related structures-directly support the authors' conclusions. However, the manuscript currently provides far too little methodological detail on how these quantifications were performed. For example: How were ROIs defined? Were objective, quantitative rules established for segmentation? If any steps were performed manually, how was objectivity ensured? Was blinding implemented? Were analyses performed in 2D or 3D? How and from where was background estimated and subtracted? Many plots appear to show one data point per oocyte; when multiple relevant structures exist within a single oocyte, how was the reported value selected or calculated (e.g., mean/median across structures, largest structure, closest-to-cortex structure, etc.)? Because the manuscript includes multiple distinct types of quantification across figures, the authors should provide a thorough description for each analysis. Doing so would substantially strengthen confidence in the quantitative phenotypes and, consequently, in the conclusions.

Response: We thank the reviewer for raising this point. The methodological detail on how these quantifications were performed are as below and could also be found in "Method" part in the revised manuscript (line 210-220):

(1) For image acquisition, the maximum cross-section of each oocyte was selected to ensure consistency in section or focal plane selection; therefore, the analysis was

performed in 2D.

(2) Relative diameter calculation: The diameter of each ELVA the maximum cross-section was measured and divided by the diameter of the corresponding oocyte to obtain the relative diameter of that ELVA. The average relative diameter was then calculated for each oocyte, and statistical comparisons were performed across more than ten oocytes.

(3) Relative distance from cortex calculation: The distance from the center of each ELVA to the oocyte cortex was measured and divided by the oocyte radius to obtain the relative cortical distance of that ELVA. The average relative distance from cortex of all ELVAs was calculated for each individual oocyte, followed by statistical analysis and inter-group comparison across multiple oocytes.

2. The impaired migration of ELVAs toward the cortex in Psma7 KO oocytes is interesting. It is not clear whether an analogous defect is present in aged oocytes. If cortical migration is normal in aged oocytes, this would be worth stating explicitly, as it would help distinguish proteasome-dependent phenotypes that overlap with aging from those that may be specific to Psma7 loss.

Response: We sincerely thank the reviewer for improving the quality of our work.

Based on the analysis, advanced age only affects the size of ELVAs in oocytes, with minimal impact on ELVAs cortical translocation (see below, revised Fig. S1A). This may be attributed to the fact that the aged mice used in our study are 10-12 months old, equivalent to humans over 35 years of age. The reduction in PSMA7 and 20S proteasome levels in aged oocytes is less pronounced than that observed in PSMA7 knockout oocytes (Fig. 1F and 2B). More severe phenotypes might become apparent at older ages.

In all, the phenotypes exhibited by aged oocytes and PSMA7-deficient oocytes are not entirely identical; our previous statement is inaccurate. We have revised it in the Result and Discussion part of the manuscript (line 286, 558-563).

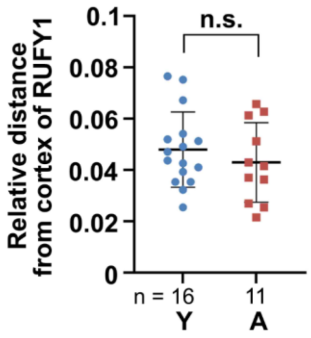

3. Related to the point above, the authors report that RAB11, which has been implicated in ELVA cortical translocation, is upregulated in Psma7 KO oocytes. This is somewhat difficult to reconcile with the observation that ELVAs nonetheless exhibit impaired migration toward the cortex in the KO. The manuscript further states that "elevated RAB11 may compensate for the impaired cortical translocation of overloaded ELVAs," but it is not clear what is meant by this. More explicit discussion of why ELVA cortical migration appears defective in Psma7 KO oocytes, and if such defects are not observed in aged oocytes, discussions of why not in aged oocytes would be helpful (e.g., whether RAB11 upregulation is interpreted as a partial/insufficient compensation, and/or whether additional limiting factors are involved).

Response: We thank the reviewer for the willingness to improve our manuscript. During the revision, we have conducted the GO analysis of the 243 PSMA7-dependent substrates of Fig. 4H and the result is presented as Fig. S4J. The result reveals that these proteins are mainly associated with intermediate filament organization, keratinization, protein localization to plasma membrane, cytoskeleton organization and so on. Compromised structural support and stability of cells may impair actin/RAB11A-dependent ELVAs relocation, so the upregulated RAB11 might be due to the compensation mechanism. As for aged oocytes, the size of ELVAs is enlarged, with minimal impact on ELVAs cortical translocation. This may be attributed to the fact that the reduction in PSMA7 and 20S proteasome levels in aged oocytes is less pronounced than that observed in PSMA7 knockout oocytes (Fig. 1F and 2B). The aged mice used in our study are 10-12 months old, equivalent to humans over 35 years of age. More severe phenotypes might become apparent at older ages. (line 412-416, 596-599)

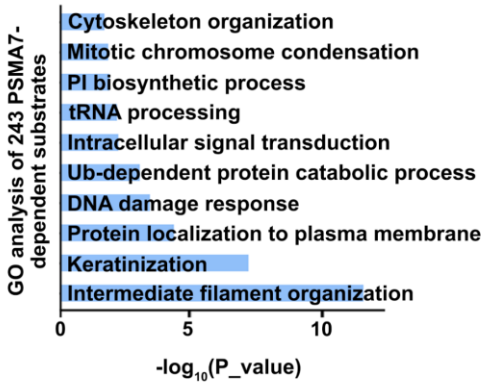

References

1. Zhang YL, Zhao LW, Zhang J, Le R, Ji SY, Chen C, Gao Y, Li D, Gao S, Fan HY: DCAF13 promotes pluripotency by negatively regulating SUV39H1 stability during early embryonic development. *EMBO J* 2018, 37(18).
2. Zhang Q, Ji SY, Busayavalasa K, Shao J, Yu C: Meiosis I progression in spermatogenesis requires a type of testis-specific 20S core proteasome. *Nat Commun* 2019, 10(1):3387.
3. Zaffagnini G, Cheng S, Salzer MC, Pernaute B, Duran JM, Irimia M, Schuh M, Boke E: Mouse oocytes sequester aggregated proteins in degradative super-organelles. *Cell* 2024, 187(5):1109-1126 e1121.
4. Rong Y, Ji SY, Zhu YZ, Wu YW, Shen L, Fan HY: ZAR1 and ZAR2 are required for oocyte meiotic maturation by regulating the maternal transcriptome and mRNA translational activation. *Nucleic Acids Res* 2019, 47(21):11387-11402.
5. Jiao YX, Bu GW, Wu YW, Wu YK, Chen BB, She MT, Zhang YH, Lu YJ, Fan HY: DHX36-mediated G-quadruplexes unwinding is essential for oocyte and early embryo development in mice. *Sci Bull (Beijing)* 2025, 70(8):1295-1310.
6. Liu Y, Zhao LW, Shen JL, Fan HY, Jin Y: Maternal DCAF13 Regulates Chromatin Tightness to Contribute to Embryonic Development. *Sci Rep* 2019, 9(1):6278.
7. Wu YW, Deng ZQ, Rong Y, Bu GW, Wu YK, Wu X, Cheng H, Fan HY: RNA surveillance by the RNA helicase MTR4 determines volume of mouse oocytes. *Dev Cell* 2025, 60(1):85-100 e104.
8. Yu C, Fan X, Sha QQ, Wang HH, Li BT, Dai XX, Shen L, Liu J, Wang L, Liu K *et al*: CFP1 Regulates Histone H3K4 Trimethylation and Developmental

Potential in Mouse Oocytes. *Cell Rep* 2017, 20(5):1161-1172.

9. Yu C, Zhang YL, Pan WW, Li XM, Wang ZW, Ge ZJ, Zhou JJ, Cang Y, Tong C, Sun QY *et al*: CRL4 complex regulates mammalian oocyte survival and reprogramming by activation of TET proteins. *Science* 2013, 342(6165):1518-1521.

1st Revision - Editorial Decision 23rd Mar 2026

Dr. Chao Yu
Zhejiang University
College of Life Sciences
Hangzhou
China

23rd Mar 2026

Re: EMBOJ-2026-123472R
20S Proteasome-Regulated Proteostasis in ELVAs is Critical for Oocyte-to-Embryo Transition and Female Fertility

Dear Dr. Yu,

Thank you for submitting your revised manuscript to The EMBO Journal. Two of the original referees have now reviewed it once more, and their comments are copied below. Both of them appreciate your revisions and improvements to the study, but referee 1 still retains important concerns regarding methodology and reproducibility, as well as a few more specific points. Given the overall improvements already made to the study, I would like to give you an opportunity to address the remaining issues during an exceptional second round of experimental revision, and I am therefore returning the manuscript to you once more with the invitation to revise.

When preparing a re-revised manuscript, I would appreciate if you could also already incorporate the following editorial issues, which would greatly facilitate processing of the manuscript in case we should eventually proceed with its publication:

- Importantly, I noticed that the manuscript lists three equally contributing first authors and four corresponding authors, with one author even assigned to both categories. Although we often see broad-based collaborations between different laboratories leading to shared corresponding authorship on many research papers in The EMBO Journal, it is unusual to include a full four co-corresponding authors all sharing the same affiliation. We certainly understand that authorship position is relevant for research assessment and academic credit, but please remember that an excessively distributive authorship also dilutes and diminishes this credit; therefore, we would consider a detailed description of each author's specific contributions (using CRediT taxonomy as well as the free-text boxes in the submission system) clearly more valuable. We would encourage the author panel to reconsider the authorship status assignments on this particular paper in light of this, and to opt for the most straightforward authorship assignment possible in accordance with journal policy.

- Please adjust the order of the manuscript sections: Title page with complete author information, Abstract, Keywords, Introduction, Results, Discussion, Methods, Data Availability, Acknowledgements, Disclosure and Competing Interests Statement, References, Main Figure Legends, Tables, Expanded Figure Legends.

- Please ensure that externally deposited data become publicly available at the time of resubmission, and adjust the Data Availability section by removing referee access tokens at this point.

- Please move all funding information into the Acknowledgements section.

- Please move the Ethics Statement into the Methods section of the manuscript.

- Please rename the Conflict of Interest section into "Disclosure and Competing Interests Statement", in accordance with our updated Guide to Authors (https://www.embopress.org/competing-interests)

- Please correct the reference list, making sure that for references with more than 10 authors, only the first up to 10 authors should be listed, followed by 'et al.' after that (please refer to our Guide to Authors for additional information on EMBO J reference format). Please also carefully go through the reference list and make sure that each reference is complete with citation year, volume, and page/elocator numbers - this information is currently incomplete for many of the references.

- Please convert the "Supplementary Tables 1 & 2" into Expanded View tables, and upload them as two separate files. Their naming and the references to them in the text should be changed to "Table EV1/2".

- Please remove mentions of "supplementary information" or "supplementary figures" from then manuscript. The EV figure legend section should have the header "EV Figure Legends".

Finally, please provide suggestions for a short 'blurb' text prefacing and summing up the conceptual aspect of the study in two sentences (max. 250 characters), followed by 3-5 one-sentence 'bullet points' with brief factual statements of key results of the paper; they will form the basis of an editor-written 'Synopsis' accompanying the online version of the article. Please also upload

a synopsis image, which can be used as a "visual title" for the synopsis section of your paper. The image (maybe based on a Fig 7 with reduced detail labeling?) should be in PNG or JPG format, and please make sure that it remains in the modest dimensions of (exactly) 550 pixels wide and 300-600 pixels high.

I am therefore returning the manuscript to you for a second, final round of revision, with the link below for eventual resubmission. Should you have any questions regarding the referee comments or this decision, please do not hesitate to contact me directly.

Yours sincerely,

Hartmut Vodermaier

Hartmut Vodermaier, PhD
Senior Editor, The EMBO Journal
h.vodermaier@embojournal.org

*** PLEASE NOTE: All revised manuscript are subject to initial checks for completeness and adherence to our formatting guidelines. Revisions may be returned to the authors and delayed in their editorial re-evaluation if they fail to comply to the following requirements. As a first step please read our guidelines for revised submissions:
https://link.springer.com/journal/44318/submission-guidelines#cms-Revised-submissions

1) Every manuscript requires a Data Availability section (even if only stating that no deposited datasets are included). Primary datasets or computer code produced in the current study have to be deposited in appropriate public repositories prior to resubmission, and reviewer access details provided in case that public access is not yet allowed.

2) Each figure legend must specify
- size of the scale bars that are mandatory for all micrograph panels
- the statistical test used to generate error bars and P-values
- the type error bars (e.g., S.E.M., S.D.)
- the number (n) and nature (biological or technical replicate) of independent experiments underlying each data point
- Figures may not include error bars for experiments with n<3; scatter plots showing individual data points should be used instead.

3) Revised manuscript text (including main tables, and figure legends for main and EV figures) has to be submitted as editable text file (e.g., .docx format). We encourage highlighting of changes (e.g., via text color) for the referees' reference.

4) Each main and each Expanded View (EV) figure should be uploaded as individual production-quality files (preferably in .eps, .tif, .jpg formats). For suggestions on figure preparation/layout, please refer to our Figure Preparation Guidelines:
https://media.springernature.com/original/springer-cms/rest/v1/content/27825798/data/v1

5) Point-by-point response letters should include the original referee comments in full together with your detailed responses to them (and to specific editor requests if applicable), and also be uploaded as editable (e.g., .docx) text files.

6) Please complete our Author Checklist, and make sure that information entered into the checklist is also reflected in the manuscript; the checklist will be available to readers as part of the Review Process File.

7) All authors listed as (co-)corresponding need to deposit, in their respective author profiles in our submission system, a unique ORCiD identifier linked to their name. Please see our Guide to Authors for detailed instructions.

8) Please note that supplementary information at EMBO Press has been superseded by the 'Expanded View' for inclusion of additional figures, tables, movies or datasets; with up to five EV Figures being typeset and directly accessible in the HTML version of the article.

9) To facilitate reproducibilty and cross-laboratory adoption of methodologies, please structure the Materials & Methods section as outlined in our guide to authors, including a completed Reagents and Tools Table.

10) Digital image enhancement is acceptable practice, as long as it accurately represents the original data and conforms to community standards. If a figure has been subjected to significant electronic manipulation, this must be clearly noted in the figure legend and/or the 'Materials and Methods' section. The editors reserve the right to request original versions of figures and the original images that were used to assemble the figure. Finally, we generally encourage uploading of numerical as well as gel/blot image source data.

At EMBO Press we ask authors to provide source data for the main manuscript figures. You will receive a separate email with instructions for providing source data with your revised manuscript, including how to upload and organize the files.

In the interest of ensuring the conceptual advance provided by the work, we recommend submitting a revision within 3 months (21st Jun 2026). Please discuss the revision progress ahead of this time with the editor if you require more time to complete the revisions. Use the link below to submit your revision:

Link Not Available

Referee #1:

The authors have provided a substantial amount of clarification and edits to improve the manuscript. I thank them for tending to many of my concerns and suggestions. I have a few remaining.

1- Size of a 3D organelle cannot be accurately measured in a 2D approach. Furthermore, deciding a "maximum cross section" is subjective and introduces bias. Because the size of the ELVAs is a major point of their study, I find this explanation and method not an improvement. The authors must obtain measures in 3D using volume. This is essential for reproducibility and rigor of the study.

2- The authors should include a reference to micro-proteomics.

3- The authors need a rationale at the start of the results of why they performed the Me4BodipyFL to being with.

4- The authors describe that PMSA7 KO is a chronic situation for why they use acute MG132 treatment. I'm not convinced it is a true "chronic" issue since there is a reasonable amount of protein remaining in the oocyte. Can they authors provide a stronger rationale for the MG132 experiment?

5- "Protein degradation and translation are two core links in protein homeostasis" doesn't make sense to me. Better might be something like "Protein homeostasis is maintained through a balance of protein degradation and translation (citation needed)".

Referee #2:

The authors have fully addressed this reviewers concerns. I recommend its publication.

Referee #1:

The authors have provided a substantial amount of clarification and edits to improve the manuscript. I thank them for tending to many of my concerns and suggestions. I have a few remaining.

Response: We sincerely thank the reviewer for your constructive suggestions and valuable efforts to enhance the quality of our manuscript. We have carefully addressed each of the issues raised and incorporated corresponding revisions in red into the manuscript.

1- Size of a 3D organelle cannot be accurately measured in a 2D approach. Furthermore, deciding a "maximum cross section" is subjective and introduces bias. Because the size of the ELVAs is a major point of their study, I find this explanation and method not an improvement. The authors must obtain measures in 3D using volume. This is essential for reproducibility and rigor of the study.

Response: We fully agree with the reviewer that 3D volume measurement is the appropriate and rigorous approach for quantifying ELVA size, and we thank the reviewer for this critical suggestion.

We have re-acquired the 3D images of oocytes and quantified their 3D volume. Specifically, after immunofluorescence operation, oocytes were placed in droplets on a glass-bottom 35mm dish covered with mineral oil and imaged with the confocal microscope. Z-stack was taken encompassing the entire oocyte with 31 slices. The volume of ELVA was quantified with the reported custom-made FIJI macro (Zaffagnini et al., 2024), available in Zenodo: https://doi.org/10.5281/zenodo.10446149. The top ten largest ELVAs by volume were quantified and averaged to represent the single value per oocyte, followed by statistical analysis and inter-group comparison across multiple oocytes. Following this flow, we have obtained a more intuitive observation of ELVAs and supplemented the volume results of RUFY1 and LAMP1 in young v.s. aged eggs (Fig. 1D, E) and WT v.s. *Psma7* cKO eggs (Fig. 3C, G). The overall conclusions remain unchanged: aged oocytes and *Psma7* cKO oocytes exhibit significantly enlarged RUFY1- and LAMP1-positive compartments.

We have included corresponding descriptions in Results and Methods parts (line 143-145, 206, 578-585) and provided the related Source Data files to the editor system to ensure full reproducibility. We believe these revisions substantially strengthen the rigor and reproducibility of our study, and we are grateful to the reviewer for insisting on this essential methodological improvement.

2- The authors should include a reference to micro-proteomics.

Response: We appreciate the reviewer's thoughtful suggestions. We have supplemented the reference related to this micro-proteomic approach (Zhang H, Genome Biol, 2023) in the revised Methods part (line 604). They have described this approach procedure in detail, and demonstrated the superior quantification ability of this highly sensitive and quantitative MS approach in fully grown oocytes with

varying cell numbers (10, 100, 200, and 500). They then used 100 oocytes/embryos at each stage to successfully obtain high-quality proteome data from fully grown oocyte to blastocyst stages.

3- The authors need a rationale at the start of the results of why they performed the Me4BodipyFL to being with.
Response: We appreciate the reviewer's thoughtful suggestions. We fully agree that explicitly stating the rationale at the start of the Results section improves the logical flow and readability of our manuscript. Specifically, we have inserted the following sentences in revised manuscript (line 128-132):
"The proteasome is a primary machinery for targeted protein degradation, and its functional integrity is essential for maintaining proteostasis. Given that the loss of protein homeostasis is the common feature of cellular aging (Labbadia and Morimoto, 2015), we examined whether proteasomal activity of oocytes is altered during maternal aging using the cell-permeable fluorescent probe Me4BodipyFL."

4- The authors describe that PMSA7 KO is a chronic situation for why they use acute MG132 treatment. I'm not convinced it is a true "chronic" issue since there is a reasonable amount of protein remaining in the oocyte. Can they authors provide a stronger rationale for the MG132 experiment?
Response: We sincerely thank the reviewer for this insightful comment. We would like to clarify that by "chronic," we refer to the sustained enzymatic deficiency arising from genetic ablation throughout oocyte development, as distinct from acute pharmacological inhibition during MZT. We acknowledge that residual PSMA7 protein persists in cKO oocytes, resulting in incomplete decrease of 20S and proteasomal inhibition. Therefore, to further investigate the consequences of total proteasome inhibition on ELVA formation, we employed MG132 treatment as a complementary approach. We have revised the text accordingly to clarify this rationale (line 215-218).

5- "Protein degradation and translation are two core links in protein homeostasis" doesn't make sense to me. Better might be something like "Protein homeostasis is maintained through a balance of protein degradation and translation (citation needed)".
Response: We appreciate the reviewer's valuable suggestion. We have corrected the description and added corresponding citations (Balch et al., 2008, Labbadia and Morimoto, 2015), and it makes the text and experiment design more logical (line 280-281).

Referee #2:

The authors have fully addressed this reviewers concerns. I recommend its publication.
Response: We are sincerely grateful to the reviewer for the constructive advice and the commitment to improve our manuscript.

EMBO Press Author Checklist

Corresponding Author Name: Chao Yu	
Journal Submitted to: The EMBO Journal	
Manuscript Number: EMBOJ-2026-123472	

USEFUL LINKS FOR COMPLETING THIS FORM
The EMBO Journal - Author Guidelines
EMBO Reports - Author Guidelines
Molecular Systems Biology - Author Guidelines
EMBO Molecular Medicine - Author Guidelines

Reporting Checklist for Life Science Articles (updated January
This checklist is adapted from Materials Design Analysis Reporting (MDAR) Checklist for Authors. MDAR establishes a minimum set of requirements in transparent reporting in the life sciences (see Statement of Task: 10.31222/osf.io/9sm4x). Please follow the journal's guidelines in preparing your
Please note that a copy of this checklist will be published alongside your article.

Abridged guidelines for figures
1. Data
The data shown in figures should satisfy the following conditions:
- the data were obtained and processed according to the field's best practice and are presented to reflect the results of the experiments in an accurate and unbiased manner.
- ideally, figure panels should include only measurements that are directly comparable to each other and obtained with the same assay.
- plots include clearly labeled error bars for independent experiments and sample sizes. Unless justified, error bars should not be shown for technical
- if n<5, the individual data points from each experiment should be plotted. Any statistical test employed should be justified.
- Source Data should be included to report the data underlying figures according to the guidelines set out in the authorship guidelines on Data

2. Captions
Each figure caption should contain the following information, for each panel where they are relevant:
- a specification of the experimental system investigated (eg cell line, species name).
- the assay(s) and method(s) used to carry out the reported observations and measurements.
- an explicit mention of the biological and chemical entity(ies) that are being measured.
- an explicit mention of the biological and chemical entity(ies) that are altered/varied/perturbed in a controlled manner.
- the exact sample size (n) for each experimental group/condition, given as a number, not a range;
- a description of the sample collection allowing the reader to understand whether the samples represent technical or biological replicates (including how many animals, litters, cultures, etc.).
- a statement of how many times the experiment shown was independently replicated in the laboratory.
- definitions of statistical methods and measures:
  - common tests, such as t-test (please specify whether paired vs. unpaired), simple χ2 tests, Wilcoxon and Mann-Whitney tests, can be unambiguously identified by name only, but more complex techniques should be described in the methods section;
  - are tests one-sided or two-sided?
  - are there adjustments for multiple comparisons?
  - exact statistical test results, e.g., P values = x but not P values < x;
  - definition of 'center values' as median or average;
  - definition of error bars as s.d. or s.e.m.

Please complete ALL of the questions below.
Select "Not Applicable" only when the requested information is not relevant for your study.

Materials

Newly Created Materials	Information included in the manuscript?	In which section is the information available? (Reagents and Tools Table, Materials and Methods, Figures, Data Availability Section)
New materials and reagents need to be available; do any restrictions apply?	Not Applicable	

Antibodies	Information included in the manuscript?	In which section is the information available? (Reagents and Tools Table, Materials and Methods, Figures, Data Availability Section)
For **antibodies** provide the following information:<br>- Commercial antibodies: RRID (if possible) or supplier name, catalogue number and or/clone number<br>- Non-commercial: RRID or citation	Yes	Supplementary Table 1. Antibody information

DNA and RNA sequences	Information included in the manuscript?	In which section is the information available? (Reagents and Tools Table, Materials and Methods, Figures, Data Availability Section)
**Short novel DNA or RNA including primers, probes**: provide the sequences.	Yes	Supplementary Table 2. Primer sequences

Cell materials	Information included in the manuscript?	In which section is the information available? (Reagents and Tools Table, Materials and Methods, Figures, Data Availability Section)
**Cell lines:** Provide species information, strain. Provide accession number in repository **OR** supplier name, catalog number, clone number, and/**OR** RRID.	Not Applicable	
**Primary cultures:** Provide species, strain, sex of origin, genetic modification status.	Yes	Materials and Methods
Report if the cell lines were recently **authenticated** (e.g., by STR profiling) and tested for mycoplasma contamination.	Not Applicable	

Experimental animals	Information included in the manuscript?	In which section is the information available? (Reagents and Tools Table, Materials and Methods, Figures, Data Availability Section)
**Laboratory animals or Model organisms:** Provide species, strain, sex, age, genetic modification status. Provide accession number in repository **OR** supplier name, catalog number, clone number, **OR** RRID.	Yes	Materials and Methods
**Animal observed in or captured from the field:** Provide species, sex, and age where possible.	Yes	Materials and Methods
Please detail **housing and husbandry conditions**.	Yes	Materials and Methods

Plants and microbes	Information included in the manuscript?	In which section is the information available? (Reagents and Tools Table, Materials and Methods, Figures, Data Availability Section)
**Plants:** provide species and strain, ecotype and cultivar where relevant, unique accession number if available, and source (including location for collected wild specimens).	Not Applicable	
**Microbes:** provide species and strain, unique accession number if available, and source.	Not Applicable	

Human research participants	Information included in the manuscript?	In which section is the information available? (Reagents and Tools Table, Materials and Methods, Figures, Data Availability Section)
If collected and within the bounds of privacy constraints report on age, sex and gender or ethnicity for all study participants.	Yes	Materials and Methods

Core facilities	Information included in the manuscript?	In which section is the information available? (Reagents and Tools Table, Materials and Methods, Figures, Data Availability Section)
If your work benefited from core facilities, was their service mentioned in the acknowledgments section?	Not Applicable	

Design

Study protocol	Information included in the manuscript?	In which section is the information available? (Reagents and Tools Table, Materials and Methods, Figures, Data Availability Section)
If study protocol has been **pre-registered, provide DOI in the manuscript**. For clinical trials, provide the trial registration number **OR** cite DOI.	Not Applicable	
Report the **clinical trial registration number** (at ClinicalTrials.gov or equivalent), where applicable.	Not Applicable	

Laboratory protocol	Information included in the manuscript?	In which section is the information available? (Reagents and Tools Table, Materials and Methods, Figures, Data Availability Section)
Provide DOI OR other citation details if **external detailed step-by-step protocols** are available.	Yes	Materials and Methods

Experimental study design and statistics	Information included in the manuscript?	In which section is the information available? (Reagents and Tools Table, Materials and Methods, Figures, Data Availability Section)
Include a statement about **sample size** estimate even if no statistical methods were used.	Yes	Materials and Methods
Were any steps taken to minimize the effects of subjective bias when allocating animals/samples to treatment (e.g. **randomization procedure**)? If yes, have they been described?	Not Applicable	
Include a statement about **blinding** even if no blinding was done.	Yes	Materials and Methods
Describe **inclusion/exclusion criteria** if samples or animals were excluded from the analysis. Were the criteria pre-established?<br><br>If sample or data points were omitted from analysis, report if this was due to attrition or intentional exclusion and provide justification.	Yes	Materials and Methods
For every figure, are **statistical tests** justified as appropriate? Do the data meet the assumptions of the tests (e.g., normal distribution)? Describe any methods used to assess it. Is there an estimate of variation within each group of data? Is the variance similar between the groups that are being statistically compared?	Yes	Materials and Methods

Sample definition and in-laboratory replication	Information included in the manuscript?	In which section is the information available? (Reagents and Tools Table, Materials and Methods, Figures, Data Availability Section)
In the figure legends: state number of times the experiment was **replicated** in laboratory.	Yes	Figures
In the figure legends: define whether data describe **technical or biological replicates**.	Yes	Figures

Ethics

Ethics	Information included in the manuscript?	In which section is the information available? (Reagents and Tools Table, Materials and Methods, Figures, Data Availability Section)
Studies involving **human participants**: State details of **authority granting ethics approval** (IRB or equivalent committee(s), provide reference number for approval.	Yes	Ethics Statements
Studies involving **human participants**: Include a statement confirming that **informed consent** was obtained from all subjects and that the experiments conformed to the principles set out in the WMA Declaration of Helsinki and the Department of Health and Human Services Belmont Report.	Yes	Ethics Statements
Studies involving **human participants:** For publication of **patient photos**, include a statement confirming that consent to publish was obtained.	Not Applicable	
Studies involving experimental **animals**: State details of **authority granting ethics approval** (IRB or equivalent committee(s), provide reference number for approval. Include a statement of compliance with ethical regulations.	Yes	Ethics Statements
Studies involving **specimen and field samples:** State if relevant **permits** obtained, provide details of authority approving study; if none were required, explain why.	Yes	Ethics Statements

Dual Use Research of Concern (DURC)	Information included in the manuscript?	In which section is the information available? (Reagents and Tools Table, Materials and Methods, Figures, Data Availability Section)
Could your study fall under dual use research restrictions? Please check biosecurity documents and list of **select agents and toxins** (CDC): https://www.selectagents.gov/sat/list.htm	Not Applicable	
If you used a select agent, is the security level of the lab appropriate and reported in the manuscript?	Not Applicable	
If a study is subject to dual use research of concern regulations, is the name of the **authority granting approval and reference number** for the regulatory approval provided in the manuscript?	Not Applicable	

Reporting

The MDAR framework recommends adoption of discipline-specific guidelines, established and endorsed through community initiatives. Journals have their own policy about requiring specific guidelines and recommendations to complement MDAR.

Adherence to community standards	Information included in the manuscript?	In which section is the information available? (Reagents and Tools Table, Materials and Methods, Figures, Data Availability Section)
State if relevant guidelines or checklists (e.g., **ICMJE, MIBBI, ARRIVE, PRISMA**) have been followed or provided.	Not Applicable	
For **tumor marker prognostic studies,** we recommend that you follow the **REMARK** reporting guidelines (see link list at top right). See author guidelines, under 'Reporting Guidelines'. Please confirm you have followed these guidelines.	Not Applicable	
For **phase II and III randomized controlled trials**, please refer to the **CONSORT** flow diagram (see link list at top right) and submit the CONSORT checklist (see link list at top right) with your submission. See author guidelines, under 'Reporting Guidelines'. Please confirm you have submitted this list.	Not Applicable	

Data Availability

Data availability	Information included in the manuscript?	In which section is the information available? (Reagents and Tools Table, Materials and Methods, Figures, Data Availability Section)
Have **primary datasets** been deposited according to the journal's guidelines (see 'Data Deposition' section) and the respective accession numbers provided in the Data Availability Section?	Yes	Data Avaliability
Were **human clinical and genomic datasets** deposited in a public access-controlled repository in accordance to ethical obligations to the patients and to the applicable consent agreement?	Not Applicable	
Are **computational models** that are central and integral to a study available without restrictions in a machine-readable form? Were the relevant accession numbers or links provided?	Not Applicable	
If publicly available data were reused, provide the respective **data citations in the reference list**.	Yes	Figures
